# Supplementary material for: In situ SEM/EDS compositional characterization of osteocytes and blood vessels in fossil and extant turtles on untreated bone surfaces; different preservational pathways microns away
Source: PeerJ. 2020 Aug 27;8:e9833. doi: 10.7717/peerj.9833 (PMC7456530; doi:10.7717/peerj.9833)

Data S1. SEM/EDS raw analyses of fossil and extant turtles.

Mongolemys elegans, IGM-90/42 specimen

Osteocytes embedded in bone 01

1. map

Combined map

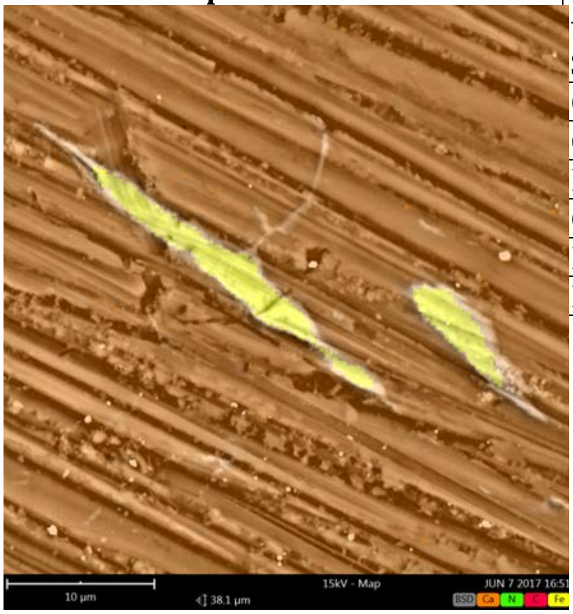

Element Symbol	Atomic Conc.	Weight Conc.	Oxide Symbol	Stoich. Conc.
O	52.77	44.67		
Ca	11.24	23.83	Ca	23.79
N	16.64	12.33	N	35.22
C	13.44	8.54	C	28.46
P	5.21	8.53	P	11.02
Fe	0.71	2.10	Fe	1.50

FOV: 38.1 μm, Mode: 15kV - Map, Detector: BSD Full, Time: JUN 7 2017 16:51

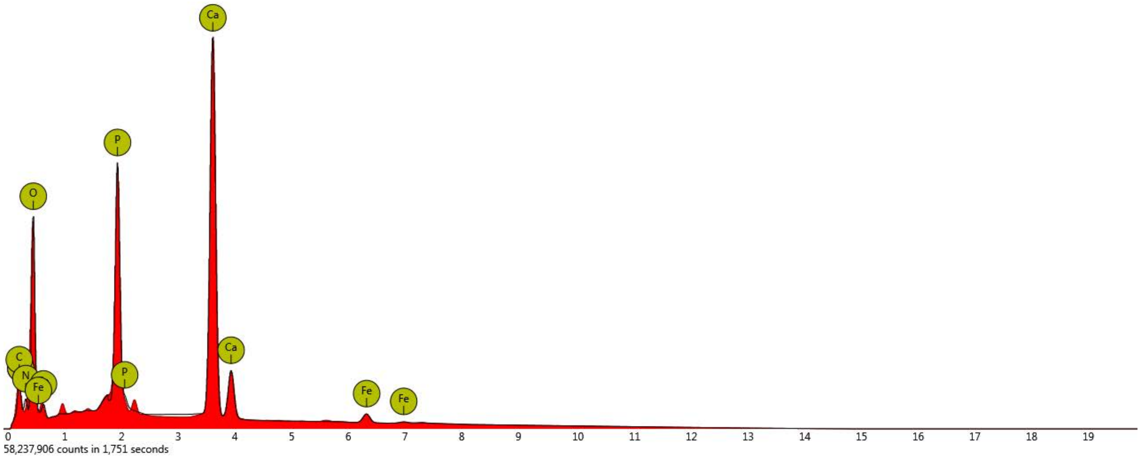

Disabled elements: B

Cut out of map (resolution: 128x128 pixels)

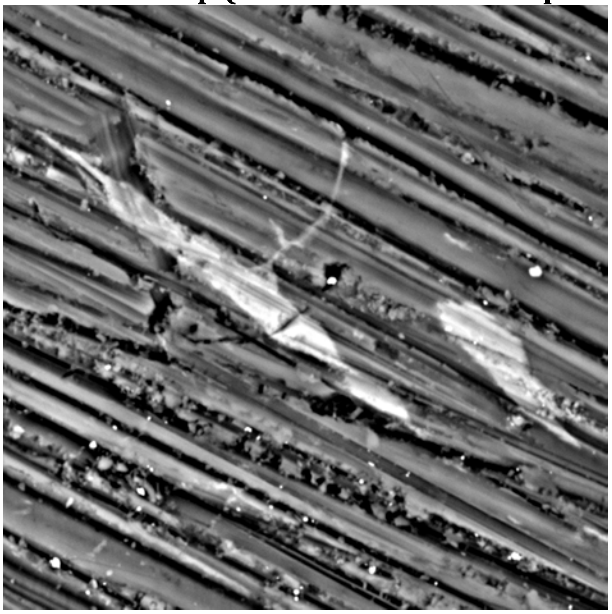

Oxygen

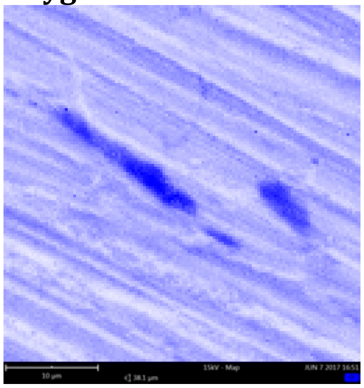

Calcium

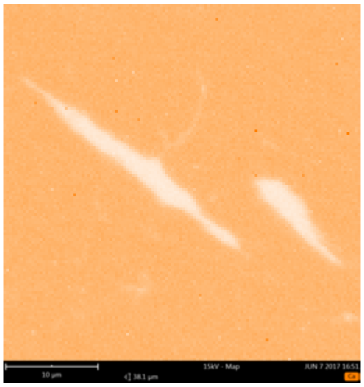

Nitrogen

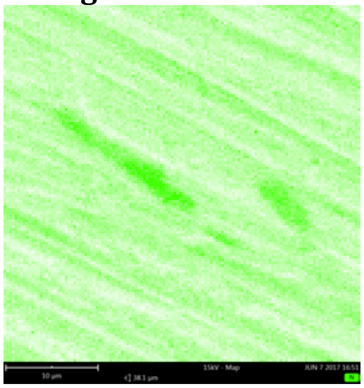

Carbon

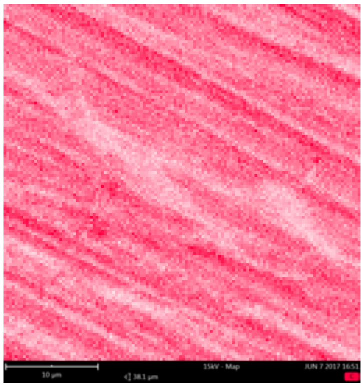

Phosphorus

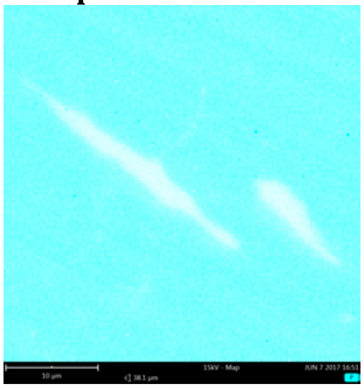

Iron

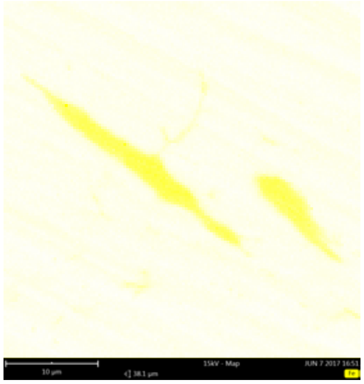

Osteocytes embedded in bone 02

1. map

Combined map

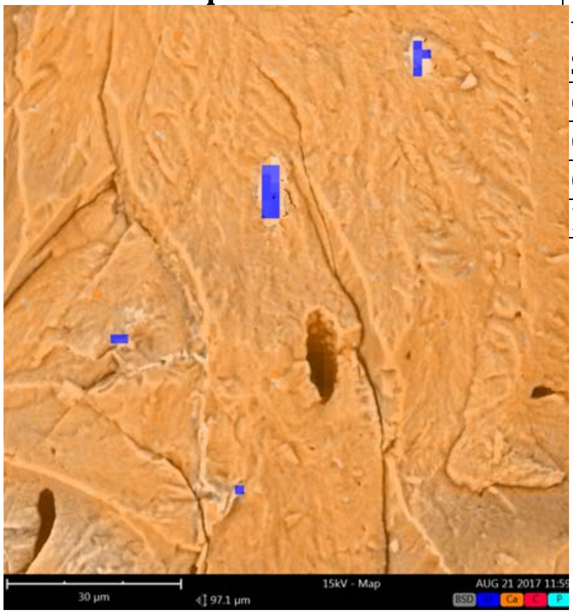

Element Symbol	Atomic Conc.	Weight Conc.	Oxide Symbol	Stoich. Conc.
O	62.69	50.61		
Ca	14.66	29.64	Ca	39.28
C	16.37	9.92	C	43.86
P	6.29	9.83	P	16.86

FOV: 97.1 μm, Mode: 15kV - Map, Detector: BSD Full, Time: AUG 21 2017 11:59

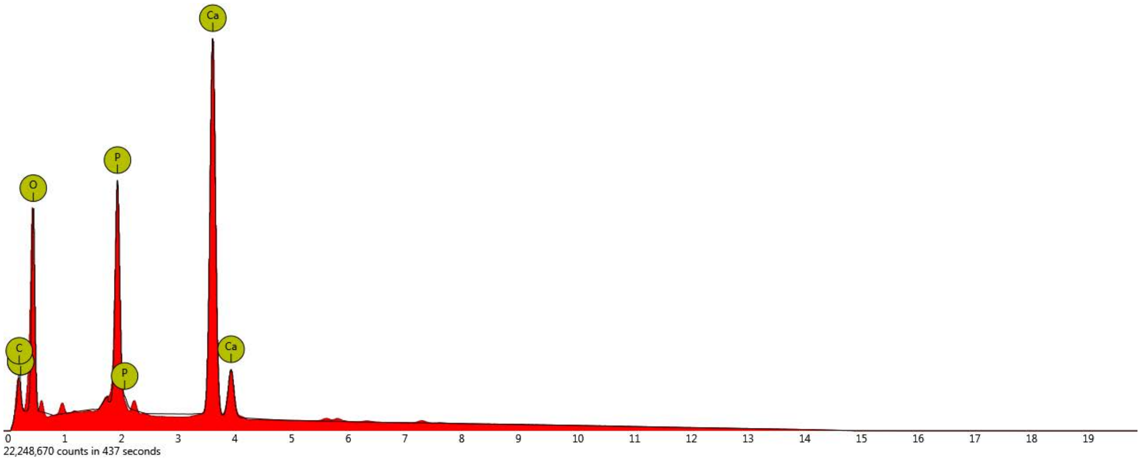

Disabled elements: B, Y

Cut out of map (resolution: 64x64 pixels)

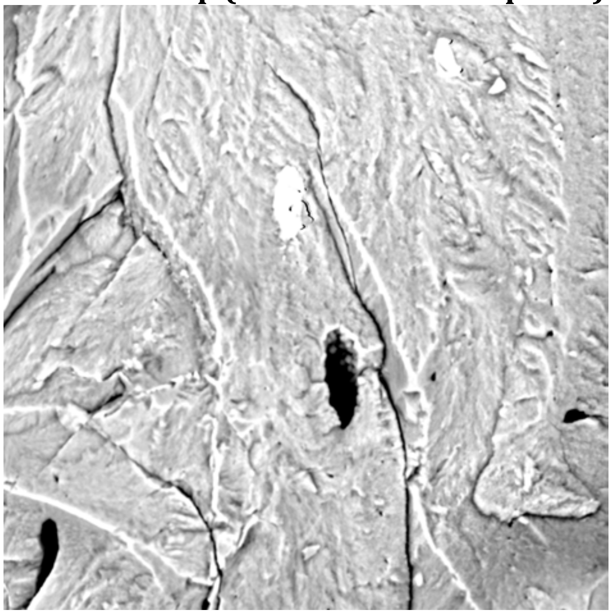

Oxygen

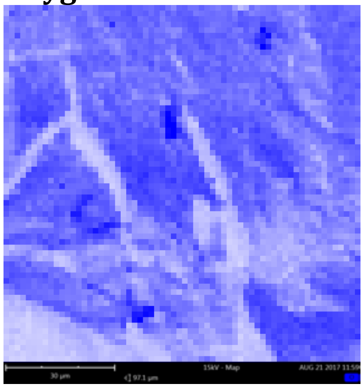

Calcium

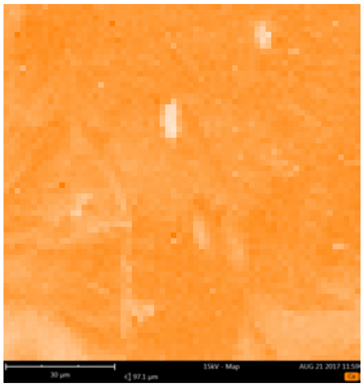

Carbon

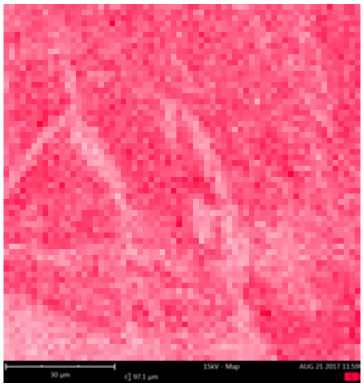

Phosphorus

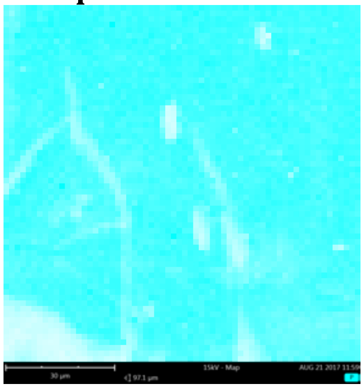

2. spot

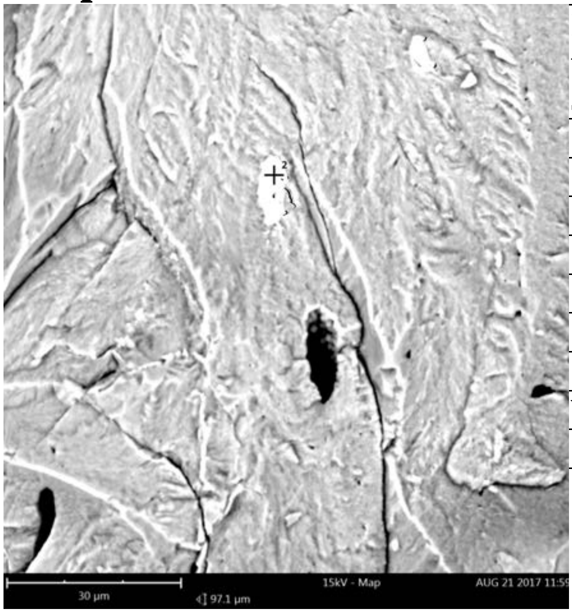

Element Symbol	Atomic Conc.	Weight Conc.	Oxide Symbol	Stoich. Conc.
O	66.68	34.38		
Mn	15.40	27.27	Mn	46.22
Ba	2.74	12.11	Ba	8.21
Ca	7.85	10.14	Ca	23.55
Pb	1.00	6.69	Pb	3.01
P	3.81	3.80	P	11.43
Fe	1.49	2.68	Fe	4.47
Y	0.54	1.54	Y	1.61
Sr	0.49	1.40	Sr	1.48

FOV: 97.1 µm, Mode: 15kV - Map, Detector: BSD Full, Time: AUG 21 2017 11:59

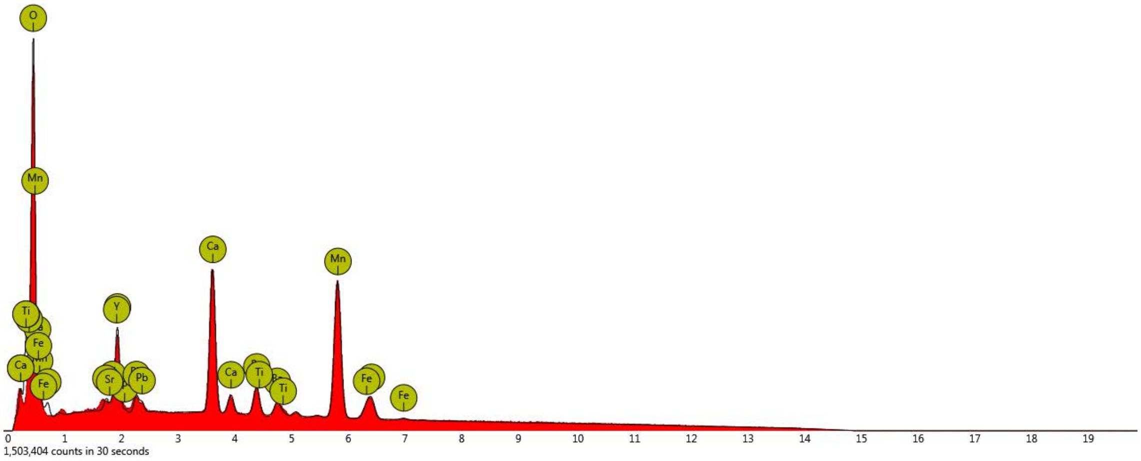

Disabled elements: B

3. spot

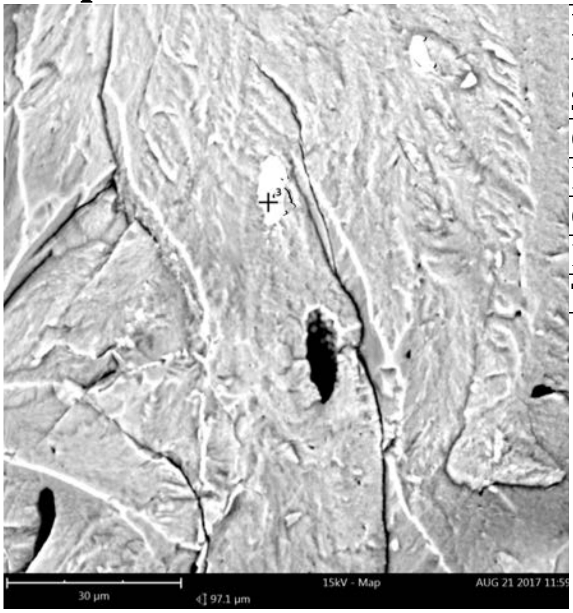

Element Symbol	Atomic Conc.	Weight Conc.	Oxide Symbol	Stoich. Conc.
O	81.92	61.20		
Mn	8.43	21.64	Mn	46.64
Ca	5.89	11.02	Ca	32.57
P	2.87	4.15	P	15.86
Ti	0.89	1.99	Ti	4.93

FOV: 97.1 µm, Mode: 15kV - Map, Detector: BSD Full, Time: AUG 21 2017 11:59

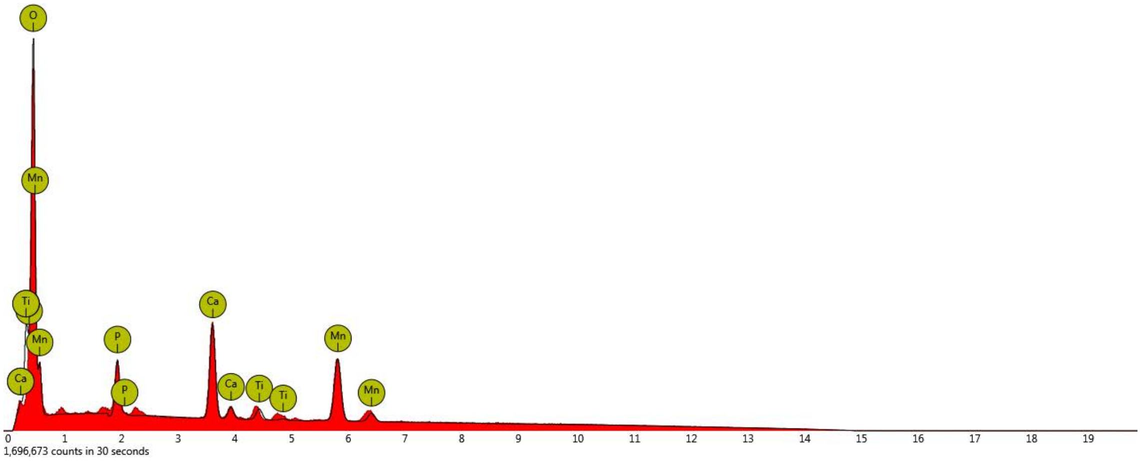

Disabled elements: B

4. spot

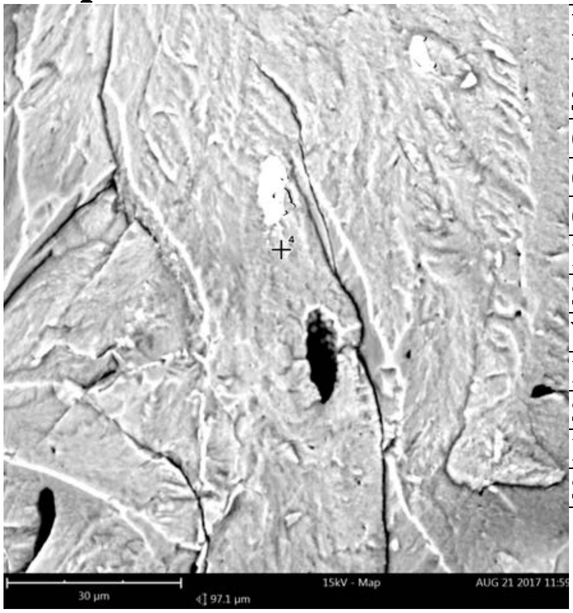

Element Symbol	Atomic Conc.	Weight Conc.	Oxide Symbol	Stoich. Conc.
O	49.96	35.54		
Ca	14.48	25.81	Ca	28.94
C	20.39	10.89	C	40.75
P	7.38	10.17	P	14.75
Sb	1.39	7.53	Sb	2.78
Y	0.76	2.99	Y	1.51
F	3.53	2.98	F	7.05
Sr	0.60	2.33	Sr	1.20
Na	0.95	0.97	Na	1.89
S	0.56	0.80	S	1.12

FOV: 97.1 µm, Mode: 15kV - Map, Detector: BSD Full, Time: AUG 21 2017 11:59

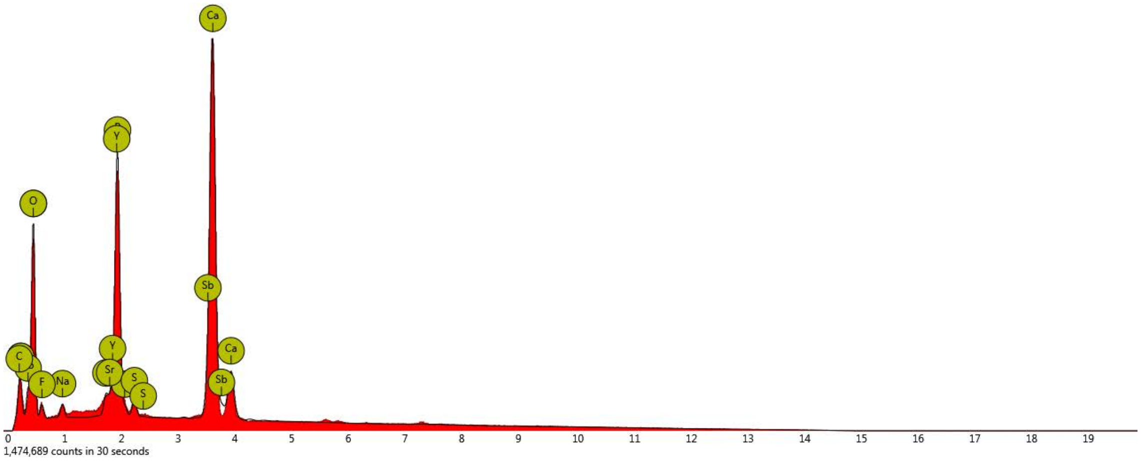

Disabled elements: B

5. spot

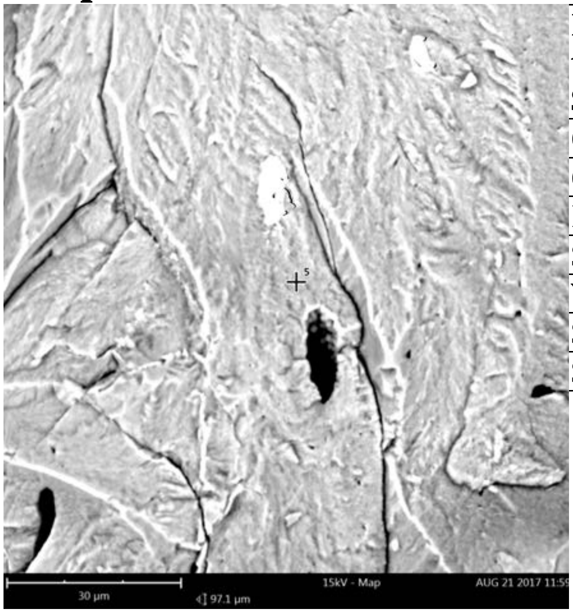

Element Symbol	Atomic Conc.	Weight Conc.	Oxide Symbol	Stoich. Conc.
O	67.82	43.34		
Ca	18.66	29.87	Ca	57.97
P	9.55	11.82	P	29.68
Sb	1.87	9.12	Sb	5.82
Y	0.81	2.86	Y	2.50
Sr	0.60	2.11	Sr	1.87
S	0.69	0.89	S	2.15

FOV: 97.1 µm, Mode: 15kV - Map, Detector: BSD Full, Time: AUG 21 2017 11:59

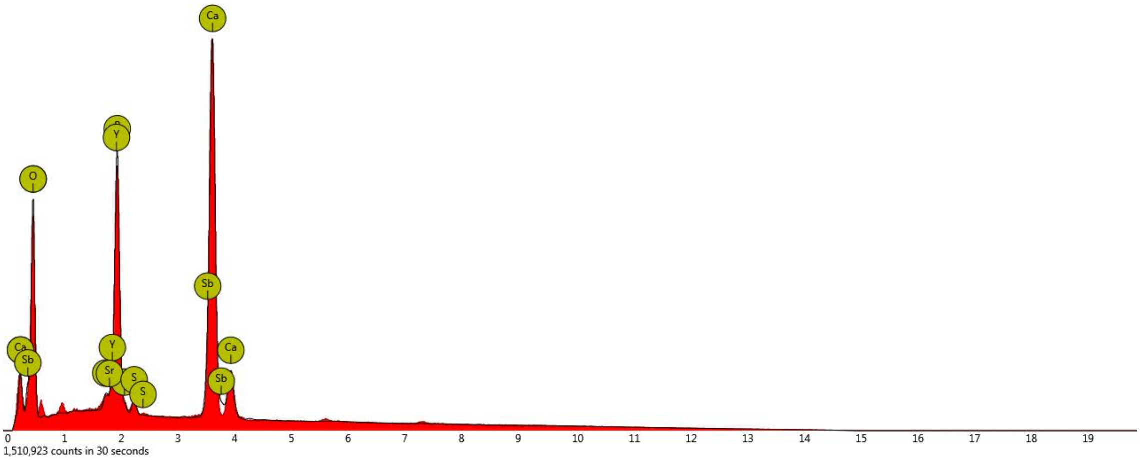

Disabled elements: B, F

6. spot

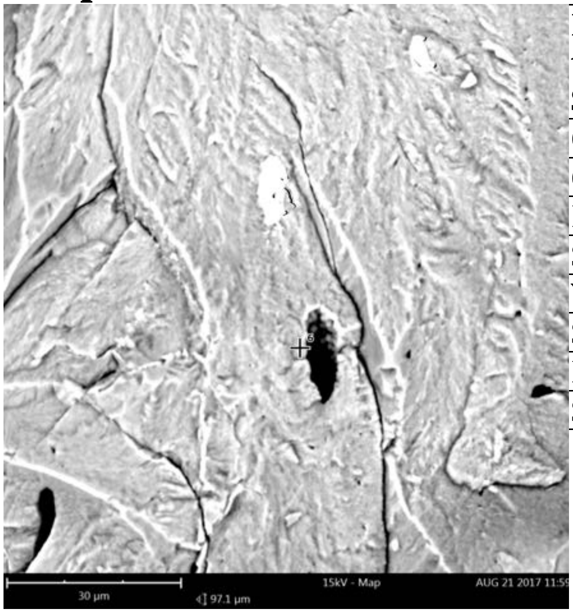

Element Symbol	Atomic Conc.	Weight Conc.	Oxide Symbol	Stoich. Conc.
O	69.50	45.75		
Ca	17.24	28.43	Ca	56.52
P	8.69	11.08	P	28.50
Sb	1.66	8.34	Sb	5.46
Y	0.73	2.66	Y	2.38
Sr	0.54	1.94	Sr	1.77
Na	0.96	0.90	Na	3.13
S	0.68	0.90	S	2.23

FOV: 97.1 µm, Mode: 15kV - Map, Detector: BSD Full, Time: AUG 21 2017 11:59

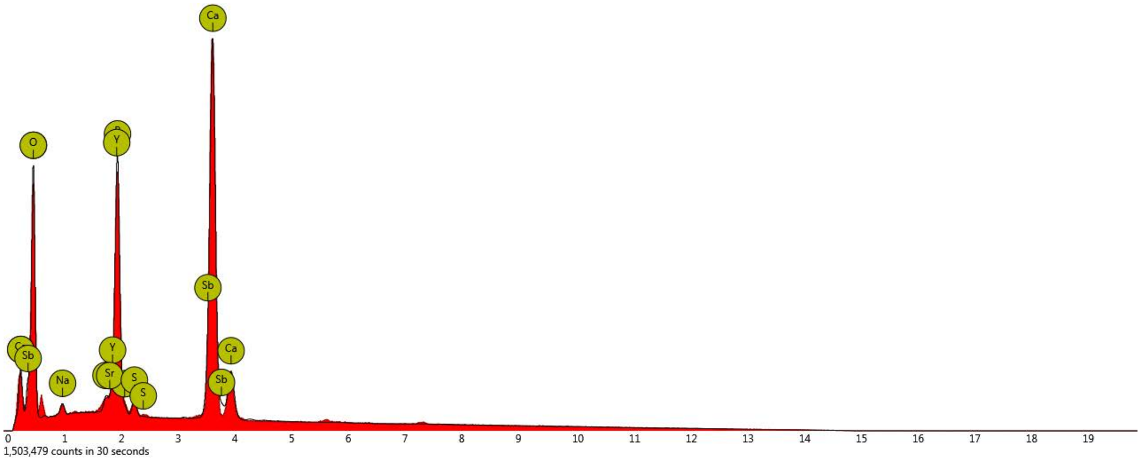

Disabled elements: B, F

7. spot

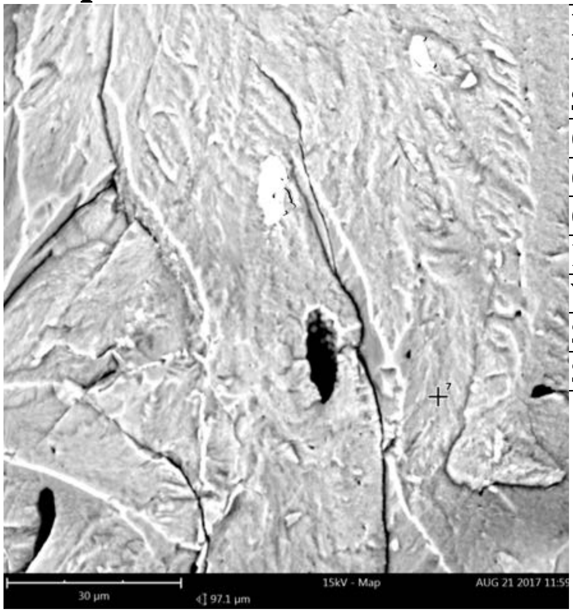

Element Symbol	Atomic Conc.	Weight Conc.	Oxide Symbol	Stoich. Conc.
O	51.24	38.23		
Ca	19.58	36.60	Ca	40.16
C	22.10	12.38	C	45.33
P	5.73	8.27	P	11.74
Y	0.57	2.36	Y	1.17
Sr	0.38	1.56	Sr	0.78
S	0.40	0.59	S	0.82

FOV: 97.1 µm, Mode: 15kV - Map, Detector: BSD Full, Time: AUG 21 2017 11:59

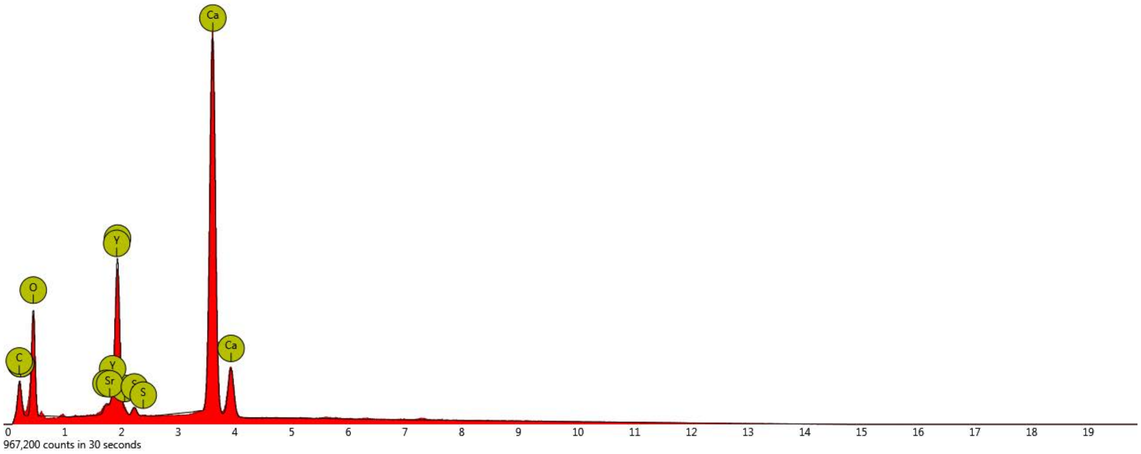

Disabled elements: B

8. spot

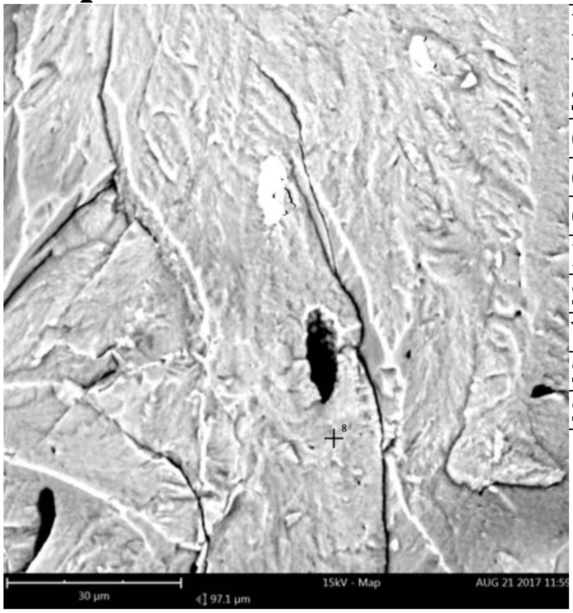

Element Symbol	Atomic Conc.	Weight Conc.	Oxide Symbol	Stoich. Conc.
O	49.15	33.92		
Ca	18.25	31.54	Ca	35.88
C	21.97	11.38	C	43.21
P	7.39	9.88	P	14.54
Sb	1.51	7.92	Sb	2.96
Y	0.71	2.74	Y	1.40
Sr	0.51	1.92	Sr	1.00
S	0.51	0.71	S	1.01

FOV: 97.1 µm, Mode: 15kV - Map, Detector: BSD Full, Time: AUG 21 2017 11:59

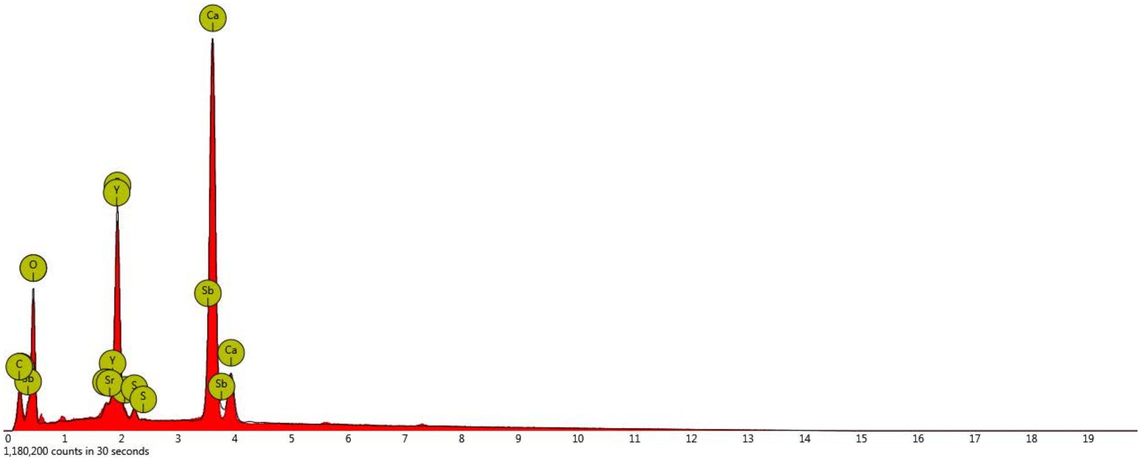

Disabled elements: B

Osteocytes embedded in bone 03

1. map

Combined map

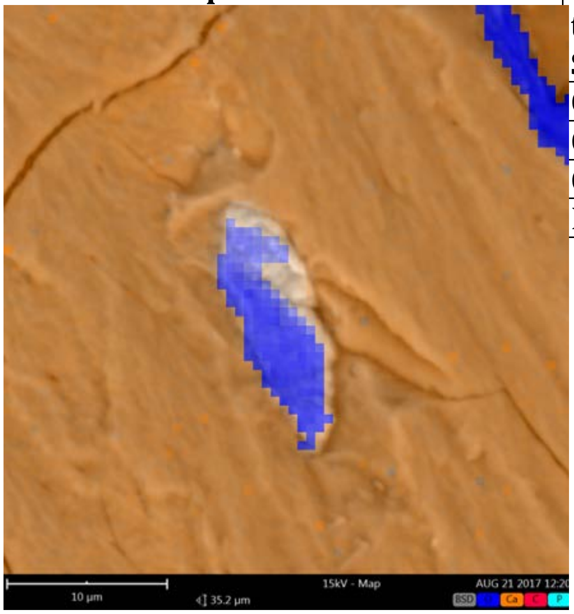

Element Symbol	Atomic Conc.	Weight Conc.	Oxide Symbol	Stoich. Conc.
O	63.79	54.27		
Ca	11.49	24.48	Ca	31.73
C	19.30	12.33	C	53.31
P	5.42	8.92	P	14.96

FOV: 35.2 µm, Mode: 15kV - Map, Detector: BSD Full, Time: AUG 21 2017 12:20

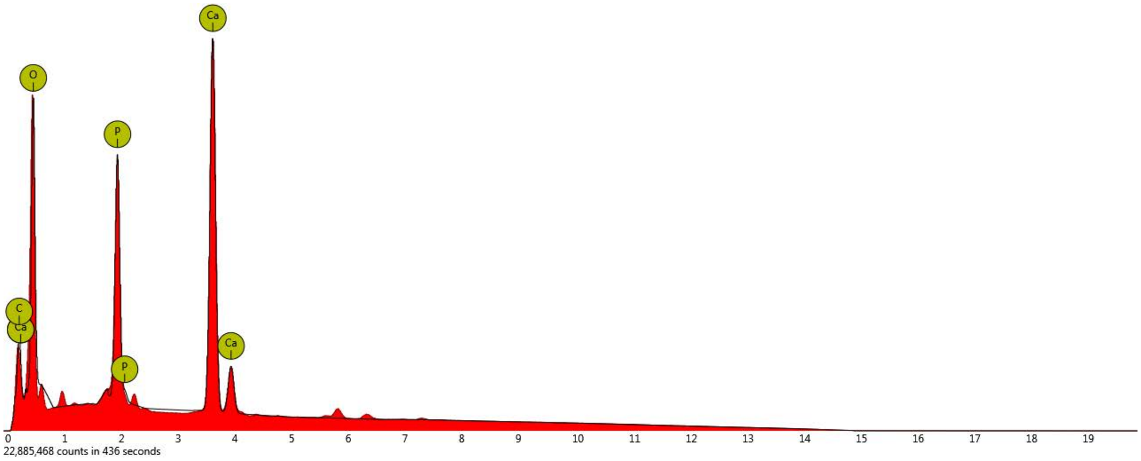

Disabled elements: B

Cut out of map (resolution: 64x64 pixels)

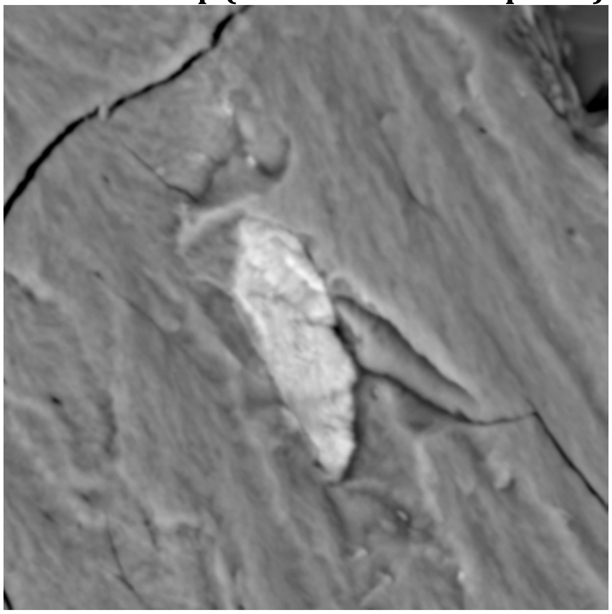

Oxygen

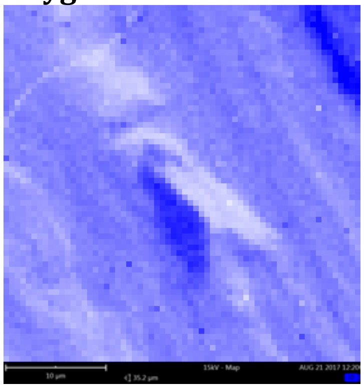

Calcium

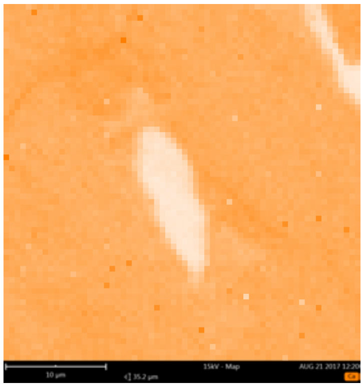

Carbon

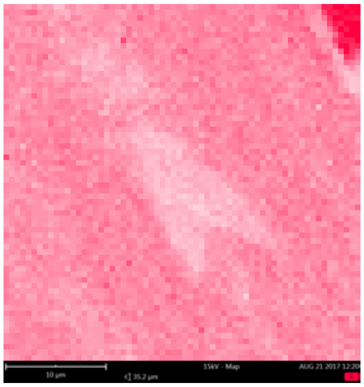

Phosphorus

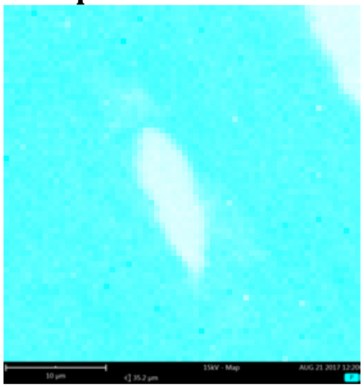

2. spot

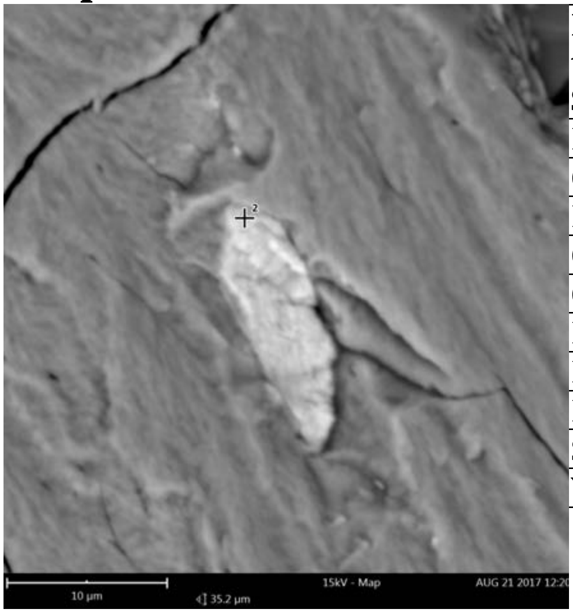

Element Symbol	Atomic Conc.	Weight Conc.	Oxide Symbol	Stoich. Conc.
Mn	18.60	33.23	Mn	27.48
O	32.33	16.82		
Ba	3.21	14.33	Ba	4.74
C	31.78	12.41	C	46.96
Ca	7.34	9.57	Ca	10.85
Pb	0.69	4.63	Pb	1.02
P	3.67	3.70	P	5.43
Fe	1.47	2.68	Fe	2.18
Sr	0.55	1.57	Sr	0.82
Y	0.36	1.05	Y	0.54

FOV: 35.2 µm, Mode: 15kV - Map, Detector: BSD Full, Time: AUG 21 2017 12:20

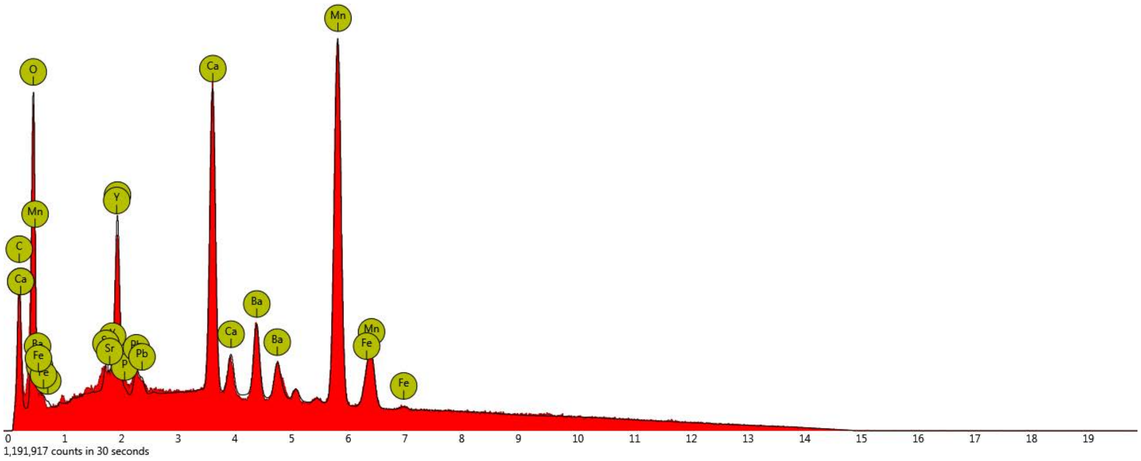

Disabled elements: B

3. spot

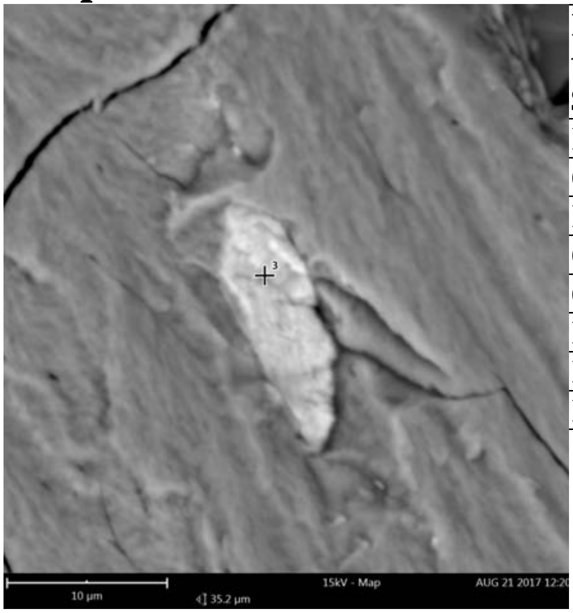

Element Symbol	Atomic Conc.	Weight Conc.	Oxide Symbol	Stoich. Conc.
Mn	14.40	30.83	Mn	24.66
O	41.60	25.94		
Ba	2.71	14.48	Ba	4.63
C	21.07	9.86	C	36.08
Ca	4.52	7.06	Ca	7.74
N	12.58	6.87	N	21.54
Fe	1.22	2.66	Fe	2.10
P	1.91	2.30	P	3.26

FOV: 35.2 µm, Mode: 15kV - Map, Detector: BSD Full, Time: AUG 21 2017 12:20

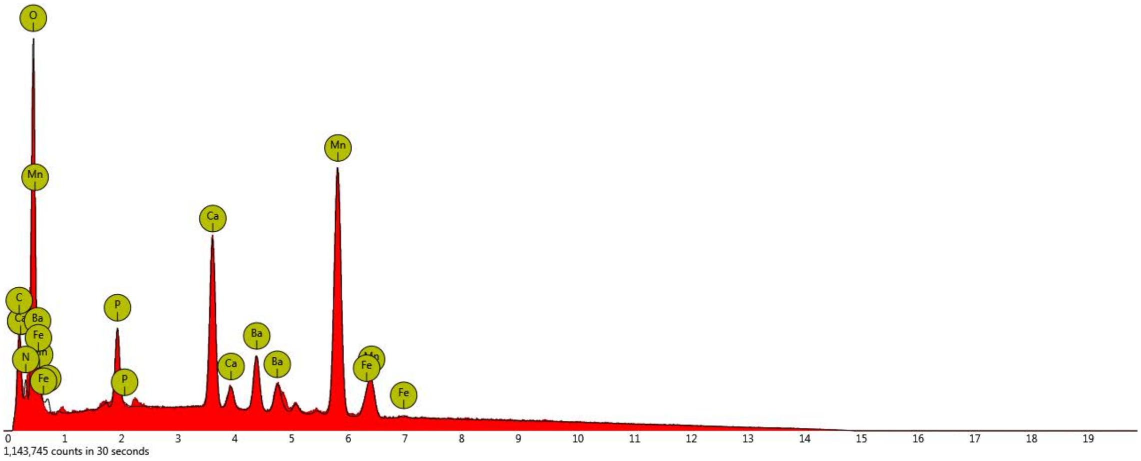

Disabled elements: B

4. spot

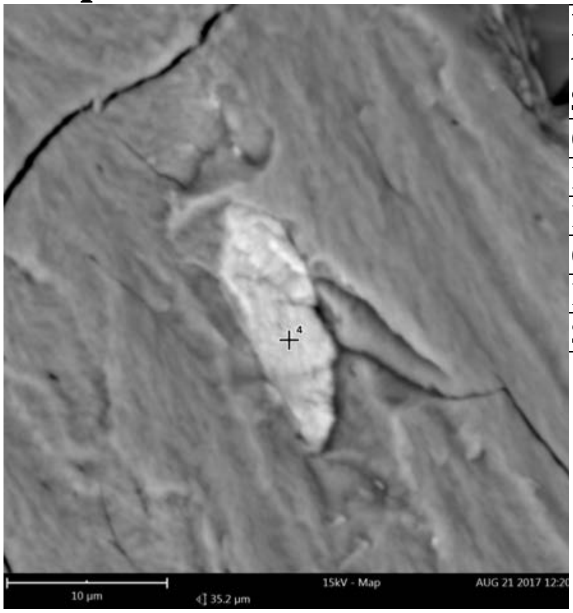

Element Symbol	Atomic Conc.	Weight Conc.	Oxide Symbol	Stoich. Conc.
O	76.04	45.03		
Mn	14.72	29.94	Mn	61.46
Ba	2.93	14.90	Ba	12.24
Ca	3.95	5.86	Ca	16.49
P	1.88	2.16	P	7.85
Sb	0.47	2.11	Sb	1.96

FOV: 35.2 μm, Mode: 15kV - Map, Detector: BSD Full, Time: AUG 21 2017 12:20

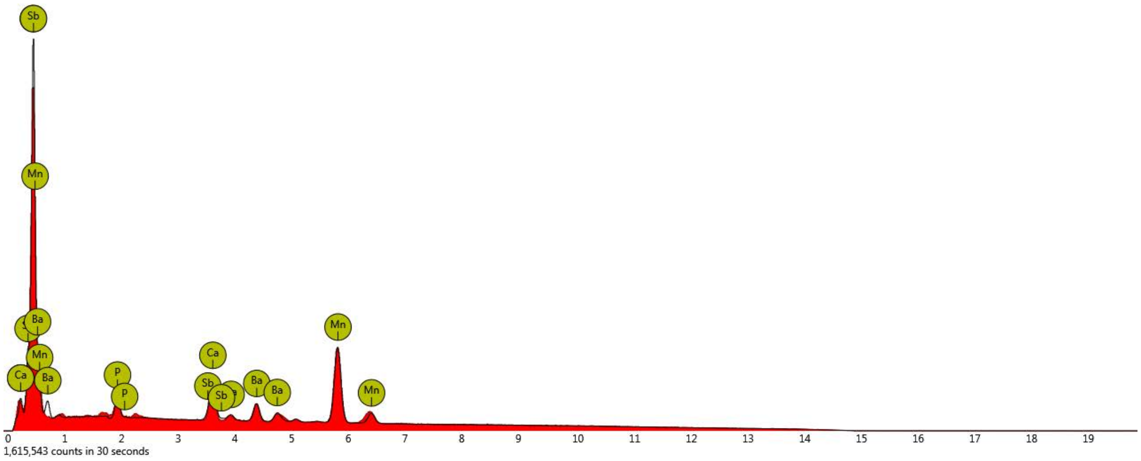

Disabled elements: B

5. spot

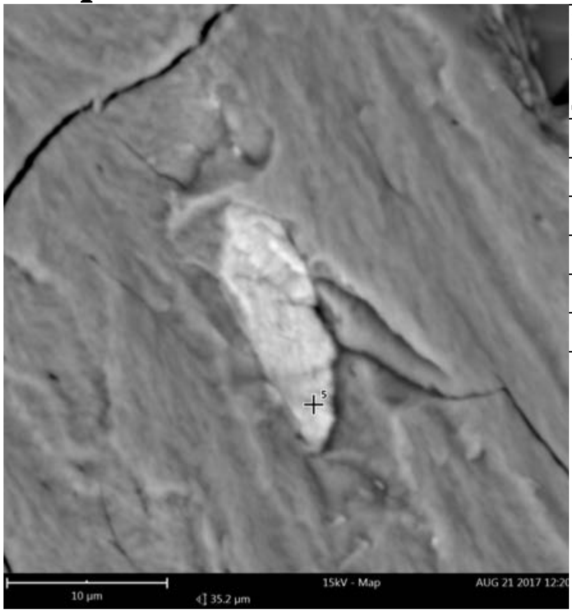

**Element Symbol**	**Atomic Conc.**	**Weight Conc.**	**Oxide Symbol**	**Stoich. Conc.**
O	78.79	50.31		
Mn	10.86	23.81	Mn	51.20
Ba	2.10	11.52	Ba	9.91
Ca	5.06	8.09	Ca	23.85
P	2.55	3.15	P	12.02
Sb	0.64	3.12	Sb	3.03

FOV: 35.2 µm, Mode: 15kV - Map, Detector: BSD Full, Time: AUG 21 2017 12:20

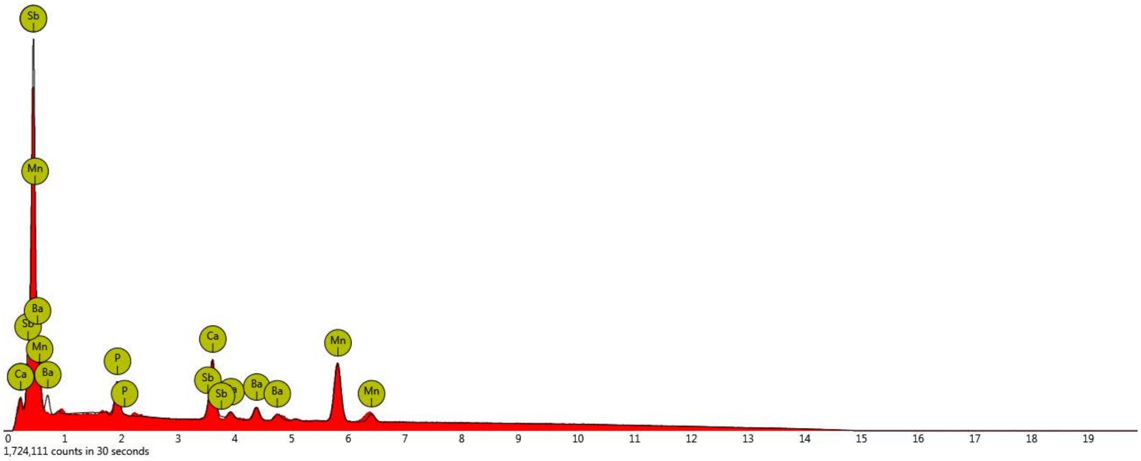

Disabled elements: B

6. spot

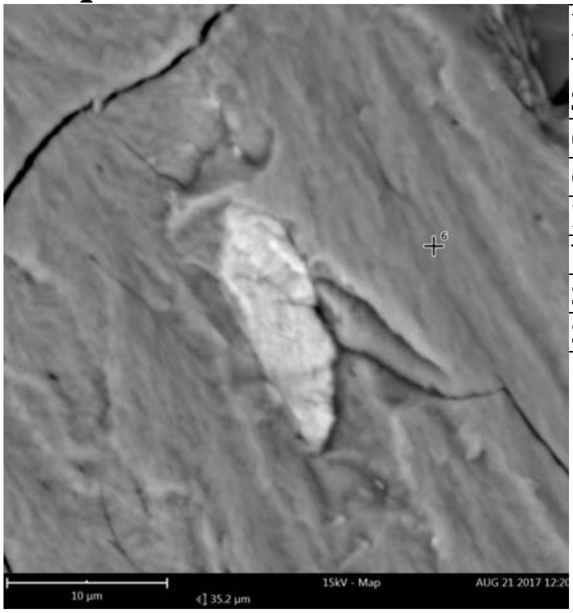

Element Symbol	Atomic Conc.	Weight Conc.	Oxide Symbol	Stoich. Conc.
O	74.15	53.97		
Ca	16.01	29.18	Ca	61.91
P	8.10	11.41	P	31.33
Y	0.62	2.49	Y	2.38
Sr	0.51	2.04	Sr	1.98
S	0.62	0.90	S	2.40

FOV: 35.2 µm, Mode: 15kV - Map, Detector: BSD Full, Time: AUG 21 2017 12:20

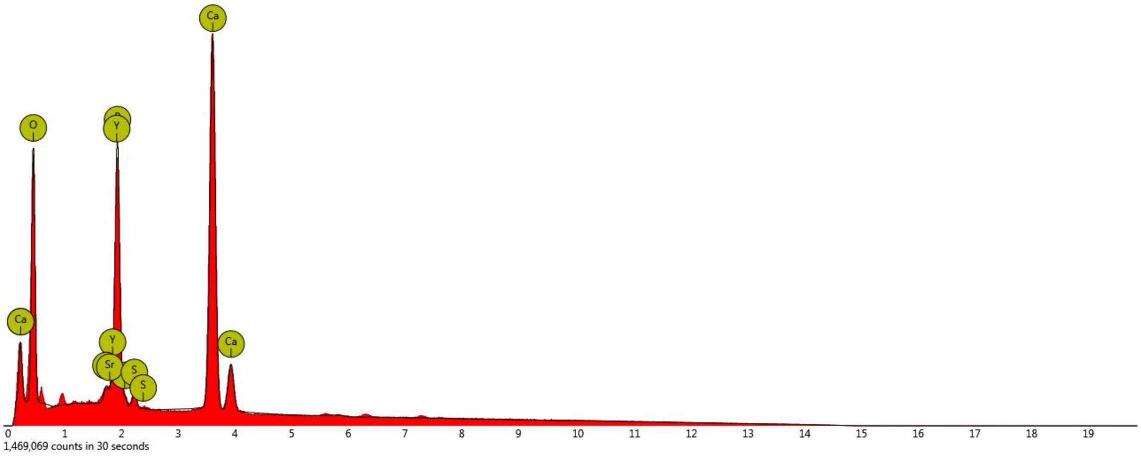

Disabled elements: B

7. spot

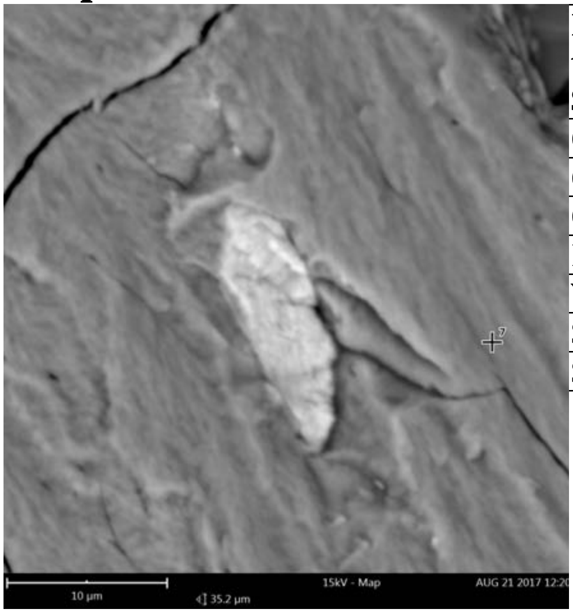

Element Symbol	Atomic Conc.	Weight Conc.	Oxide Symbol	Stoich. Conc.
O	56.93	46.64		
Ca	11.83	24.28	Ca	27.47
C	24.01	14.77	C	55.74
P	5.84	9.26	P	13.55
Y	0.58	2.66	Y	1.35
Sr	0.37	1.67	Sr	0.87
S	0.44	0.72	S	1.02

FOV: 35.2 µm, Mode: 15kV - Map, Detector: BSD Full, Time: AUG 21 2017 12:20

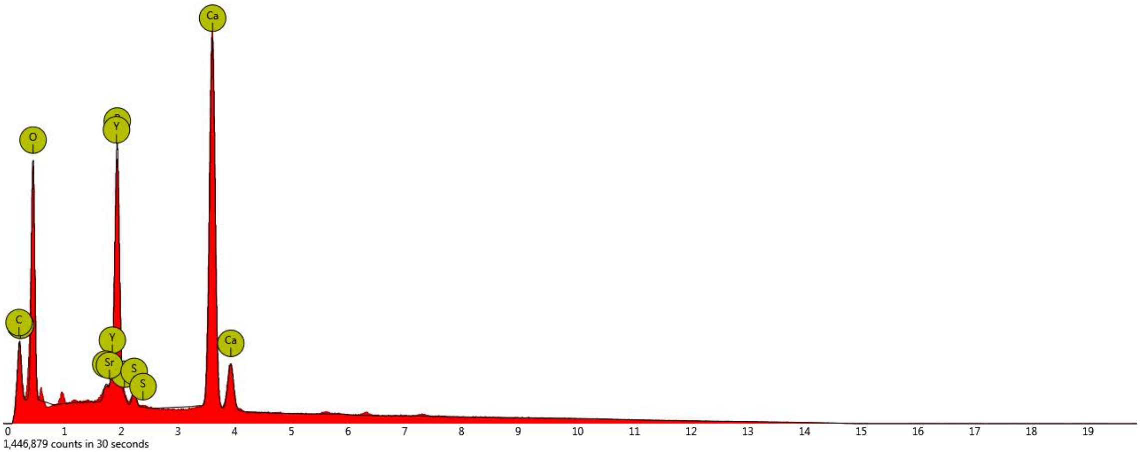

Disabled elements: B

Osteocytes embedded in bone 04

1. map

Combined map

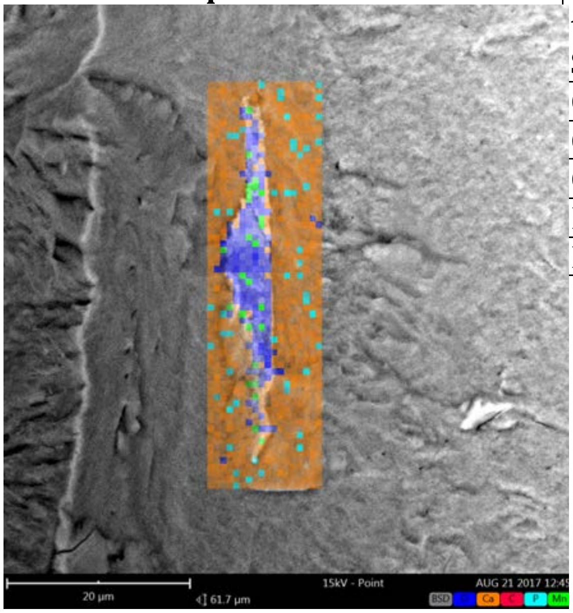

Element Symbol	Atomic Conc.	Weight Conc.	Oxide Symbol	Stoich. Conc.
O	61.00	50.74		
Ca	10.39	21.64	Ca	26.63
C	21.37	13.34	C	54.79
P	5.16	8.31	P	13.23
Mn	2.09	5.96	Mn	5.35

FOV: 61.7 µm, Mode: 15kV - Point, Detector: BSD Full, Time: AUG 21 2017 12:45

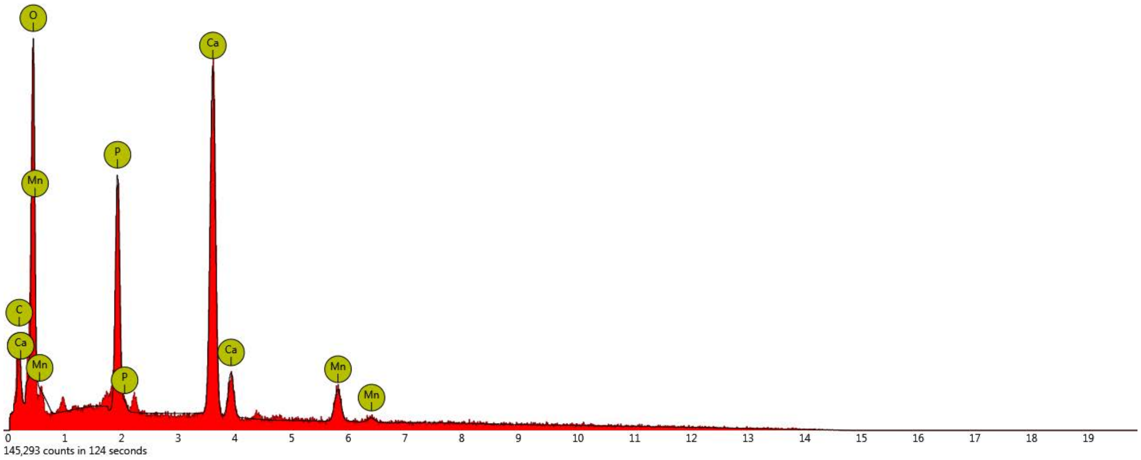

Disabled elements: B, Mo, S, Sr, Y

Cut out of map (resolution: 18x64 pixels)

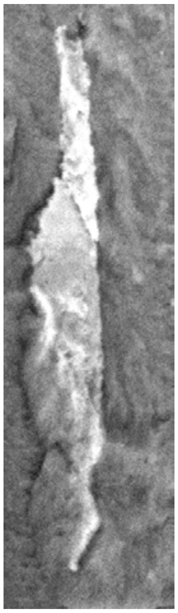

Oxygen

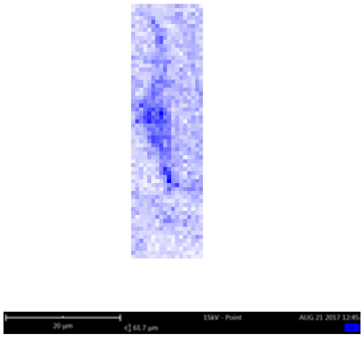

Calcium

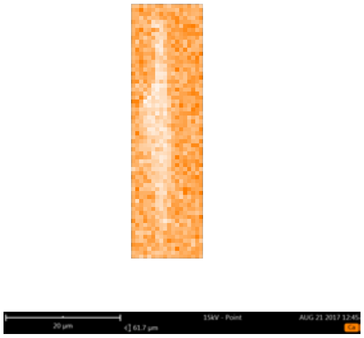

Carbon

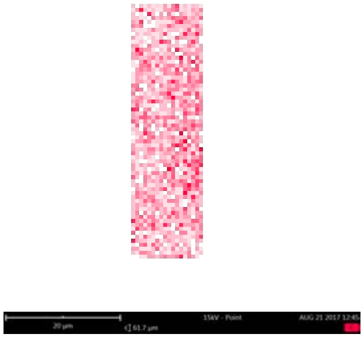

Phosphorus

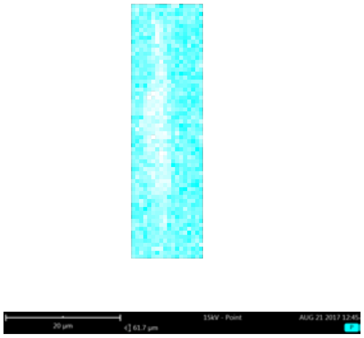

Manganese

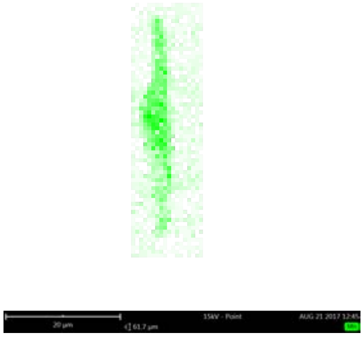

2. spot

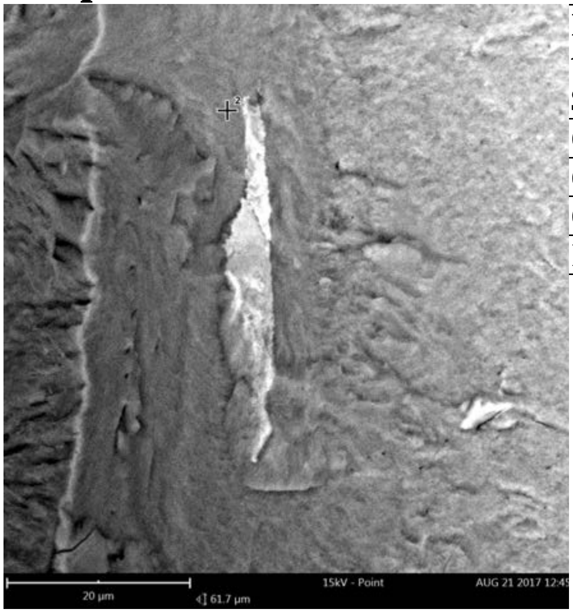

Element Symbol	Atomic Conc.	Weight Conc.	Oxide Symbol	Stoich. Conc.
O	57.33	48.73		
Ca	12.17	25.92	Ca	28.52
C	24.66	15.74	C	57.78
P	5.84	9.62	P	13.70

FOV: 61.7 µm, Mode: 15kV - Point, Detector: BSD Full, Time: AUG 21 2017 12:45

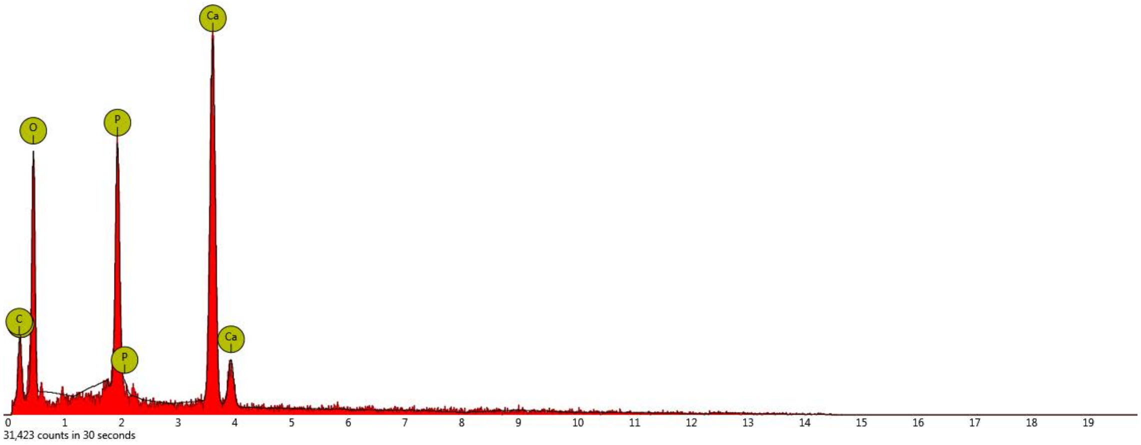

Disabled elements: B

3. spot

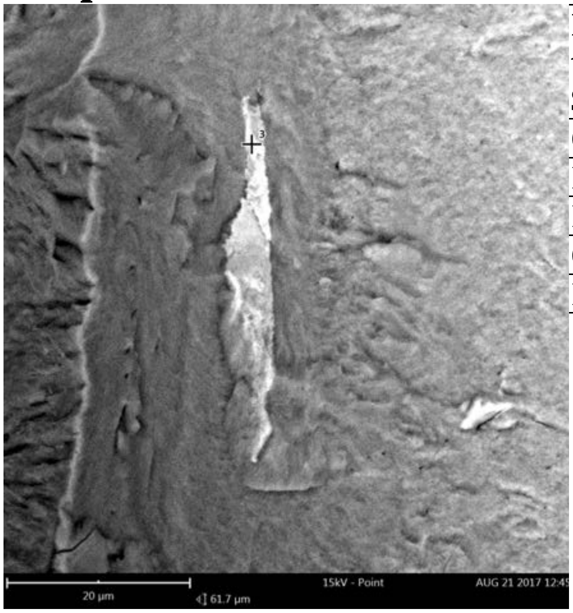

Element Symbol	Atomic Conc.	Weight Conc.	Oxide Symbol	Stoich. Conc.
O	72.80	42.45		
Mn	16.17	32.37	Mn	59.46
Ba	2.79	13.95	Ba	10.25
Ca	5.81	8.49	Ca	21.37
P	2.43	2.74	P	8.92

FOV: 61.7 µm, Mode: 15kV - Point, Detector: BSD Full, Time: AUG 21 2017 12:45

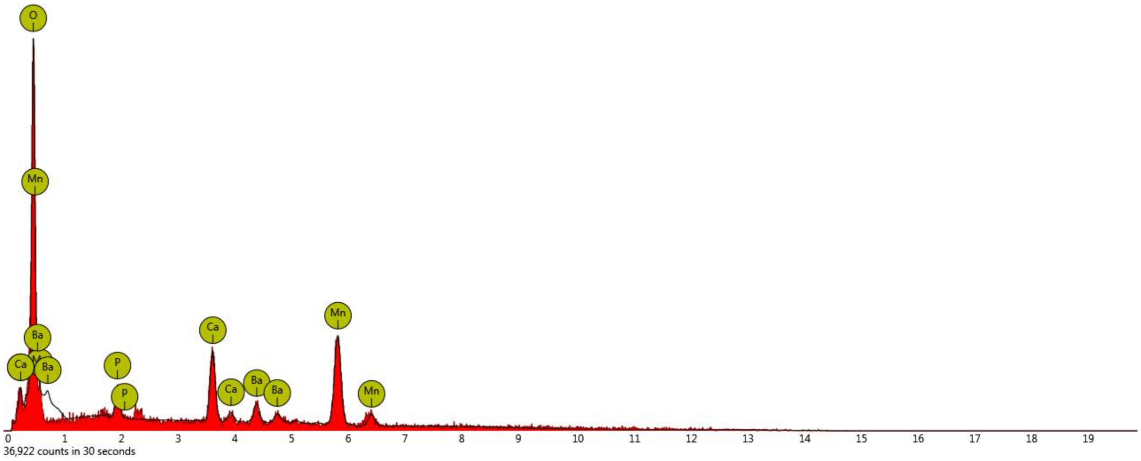

Disabled elements: B

4. spot

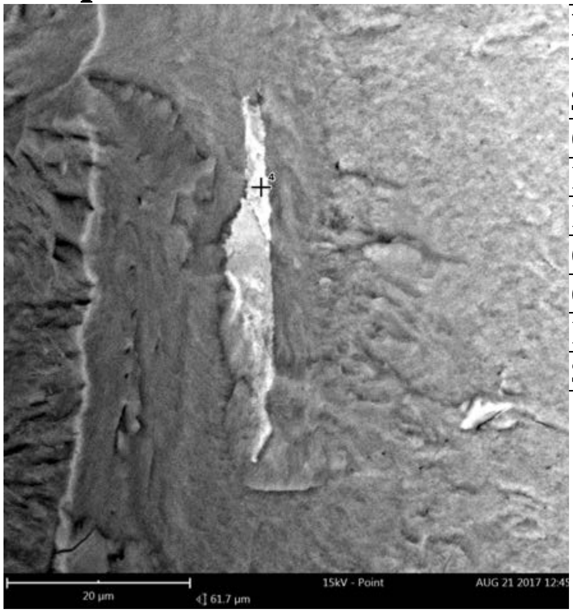

Element Symbol	Atomic Conc.	Weight Conc.	Oxide Symbol	Stoich. Conc.
O	61.97	42.84		
Mn	10.24	24.30	Mn	26.93
Ba	1.75	10.41	Ba	4.61
C	19.26	10.00	C	50.66
Ca	4.25	7.35	Ca	11.16
P	2.08	2.79	P	5.48
Sb	0.44	2.31	Sb	1.15

FOV: 61.7 µm, Mode: 15kV - Point, Detector: BSD Full, Time: AUG 21 2017 12:45

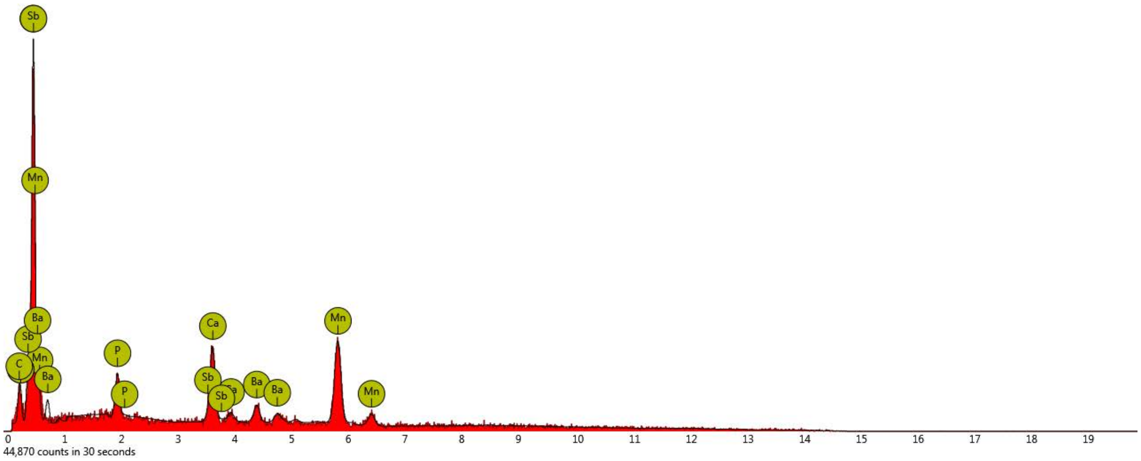

Disabled elements: B

5. spot

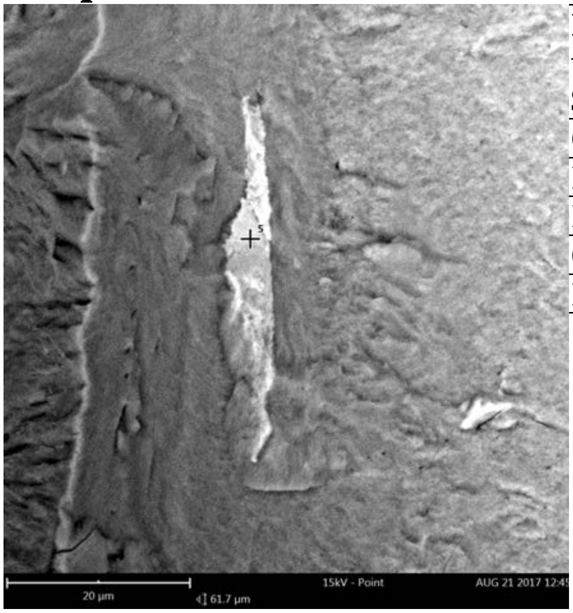

Element Symbol	Atomic Conc.	Weight Conc.	Oxide Symbol	Stoich. Conc.
O	72.19	40.65		
Mn	16.97	32.80	Mn	61.01
Ba	3.49	16.89	Ba	12.56
Ca	5.14	7.25	Ca	18.49
P	2.21	2.41	P	7.94

FOV: 61.7 µm, Mode: 15kV - Point, Detector: BSD Full, Time: AUG 21 2017 12:45

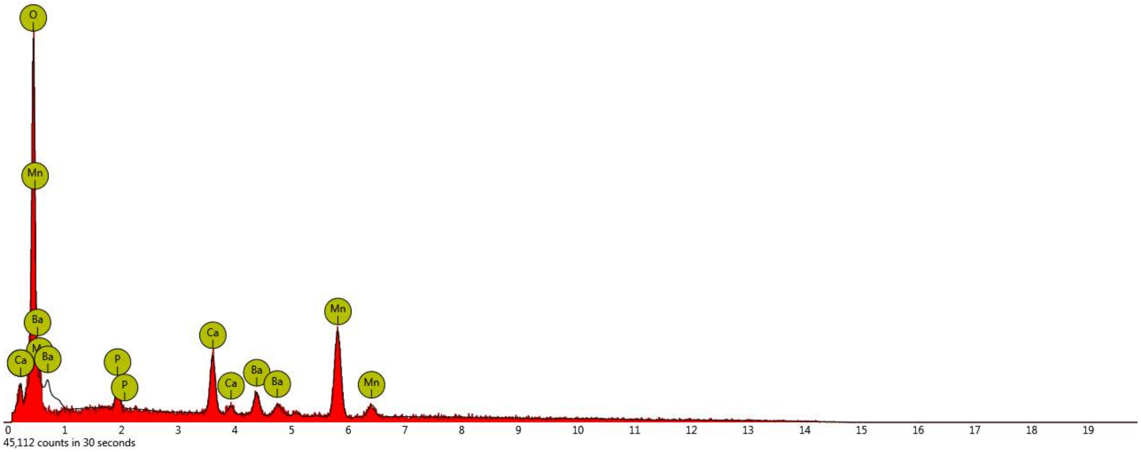

Disabled elements: B

6. spot

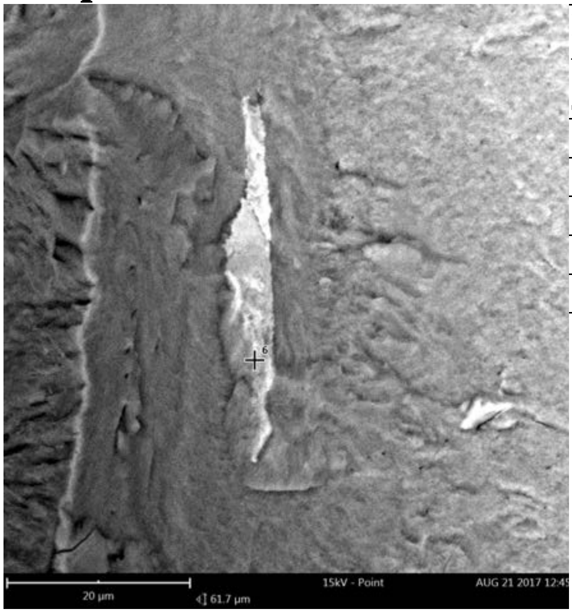

Element Symbol	Atomic Conc.	Weight Conc.	Oxide Symbol	Stoich. Conc.
O	59.36	44.41		
Mn	11.06	28.42	Mn	27.22
Ca	6.24	11.69	Ca	15.34
C	20.67	11.61	C	50.85
P	2.68	3.88	P	6.58

FOV: 61.7 µm, Mode: 15kV - Point, Detector: BSD Full, Time: AUG 21 2017 12:45

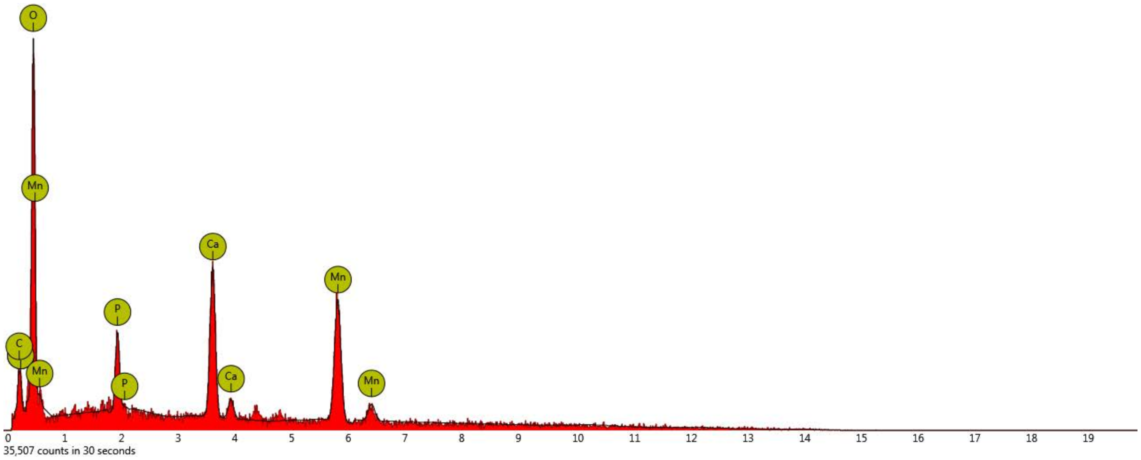

Disabled elements: B

7. spot

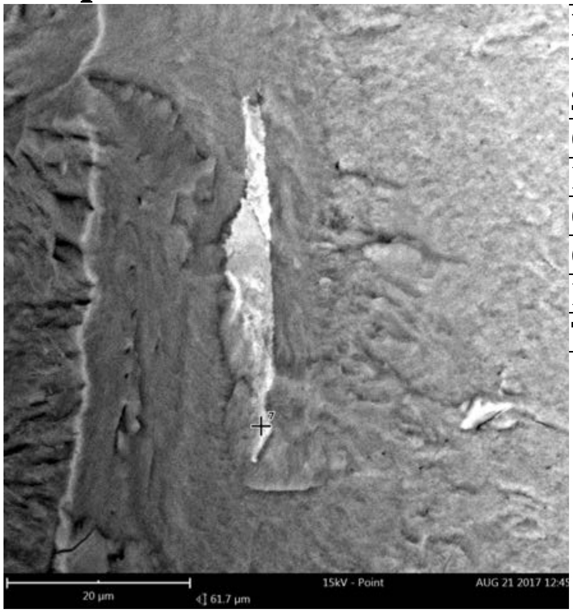

Element Symbol	Atomic Conc.	Weight Conc.	Oxide Symbol	Stoich. Conc.
O	62.24	50.34		
Mn	7.24	20.11	Mn	19.18
C	22.25	13.51	C	58.93
Ca	5.34	10.81	Ca	14.14
P	2.48	3.89	P	6.58
Ti	0.38	0.92	Ti	1.01

FOV: 61.7 µm, Mode: 15kV - Point, Detector: BSD Full, Time: AUG 21 2017 12:45

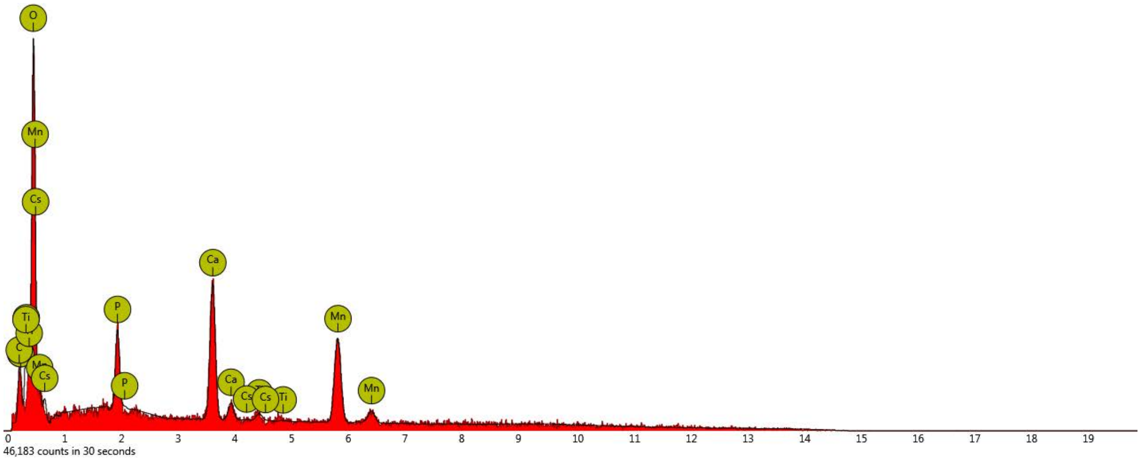

Disabled elements: B

8. spot

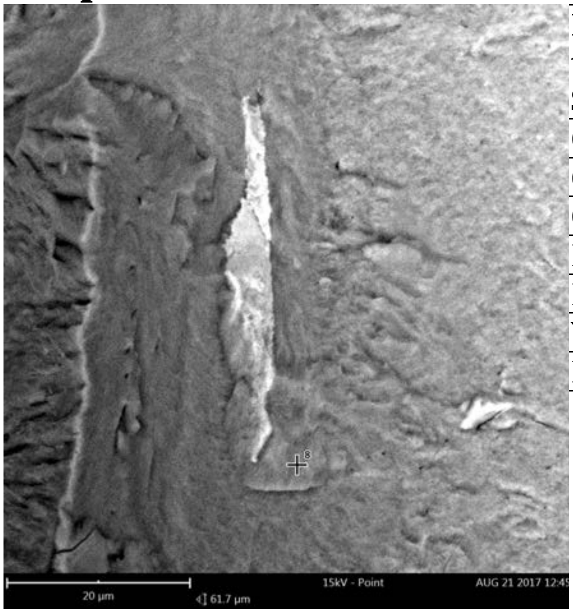

Element Symbol	Atomic Conc.	Weight Conc.	Oxide Symbol	Stoich. Conc.
O	46.47	39.86		
Ca	11.20	24.06	Ca	20.92
C	20.84	13.42	C	38.93
N	15.28	11.48	N	28.55
P	5.24	8.71	P	9.79
Y	0.33	1.58	Y	0.62
Na	0.57	0.71	Na	1.07

FOV: 61.7 µm, Mode: 15kV - Point, Detector: BSD Full, Time: AUG 21 2017 12:45

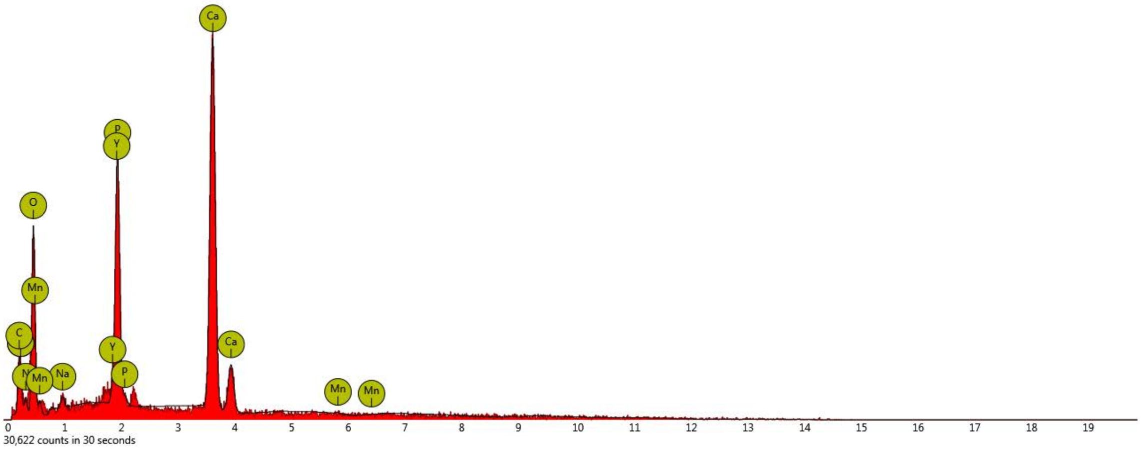

Disabled elements: B

Osteocytes embedded in bone 05

1. map

Combined map

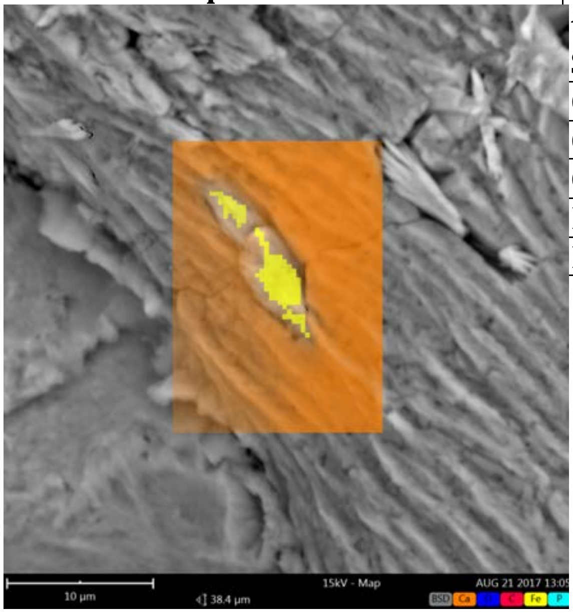

Element Symbol	Atomic Conc.	Weight Conc.	Oxide Symbol	Stoich. Conc.
Ca	22.64	40.82	Ca	49.18
O	53.97	38.86		
C	18.52	10.01	C	40.25
Fe	3.15	7.91	Fe	6.84
P	1.72	2.40	P	3.74

FOV: 38.4 µm, Mode: 15kV - Map, Detector: BSD Full, Time: AUG 21 2017 13:05

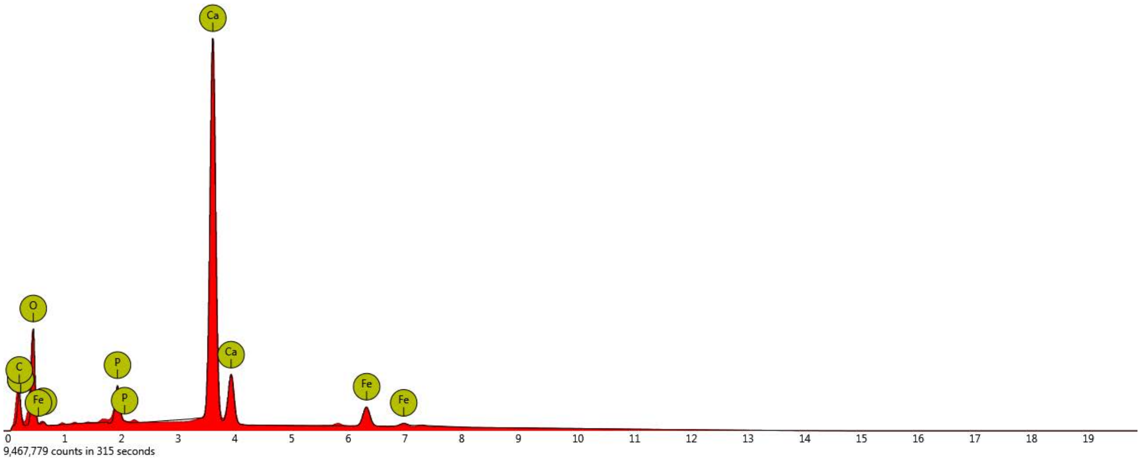

Disabled elements: B

Cut out of map (resolution: 46x64 pixels)

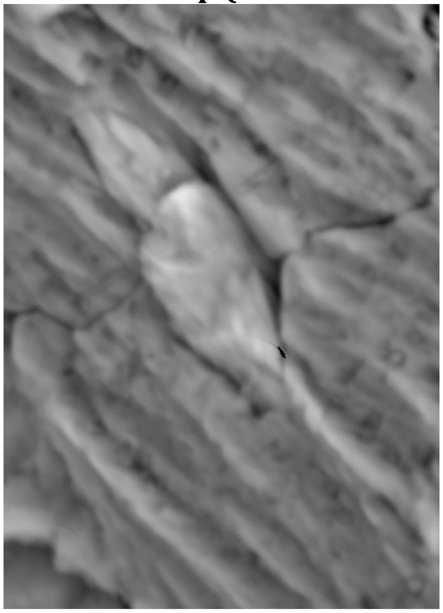

Calcium

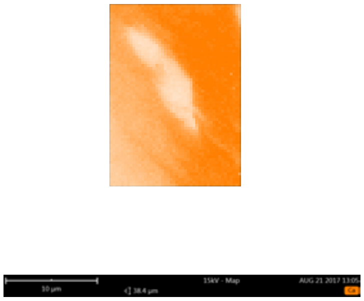

Oxygen

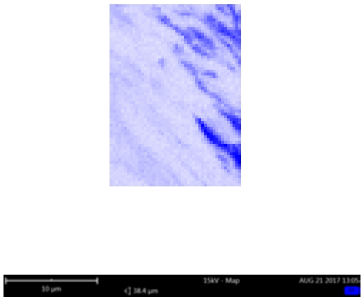

Carbon

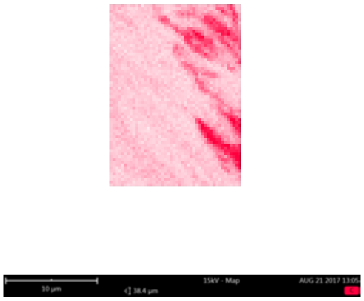

Iron

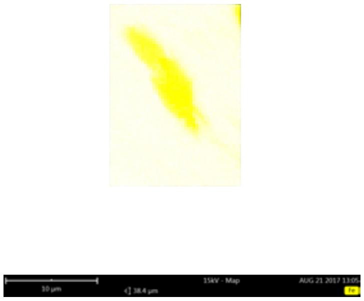

Phosphorus

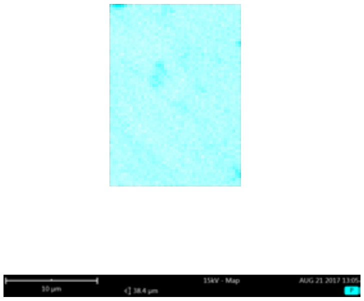

2. spot

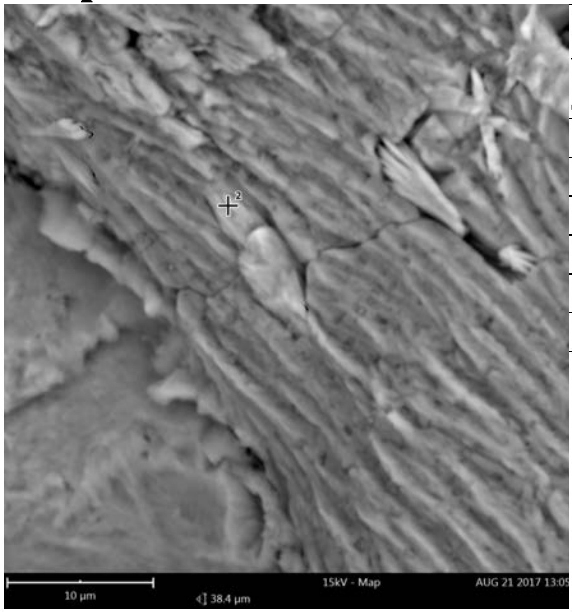

Element Symbol	Atomic Conc.	Weight Conc.	Oxide Symbol	Stoich. Conc.
Fe	19.62	42.55	Fe	28.84
O	31.99	19.88		
Ca	11.51	17.91	Ca	16.92
C	23.54	10.98	C	34.61
N	11.20	6.09	N	16.47
P	2.15	2.59	P	3.16

FOV: 38.4 µm, Mode: 15kV - Map, Detector: BSD Full, Time: AUG 21 2017 13:05

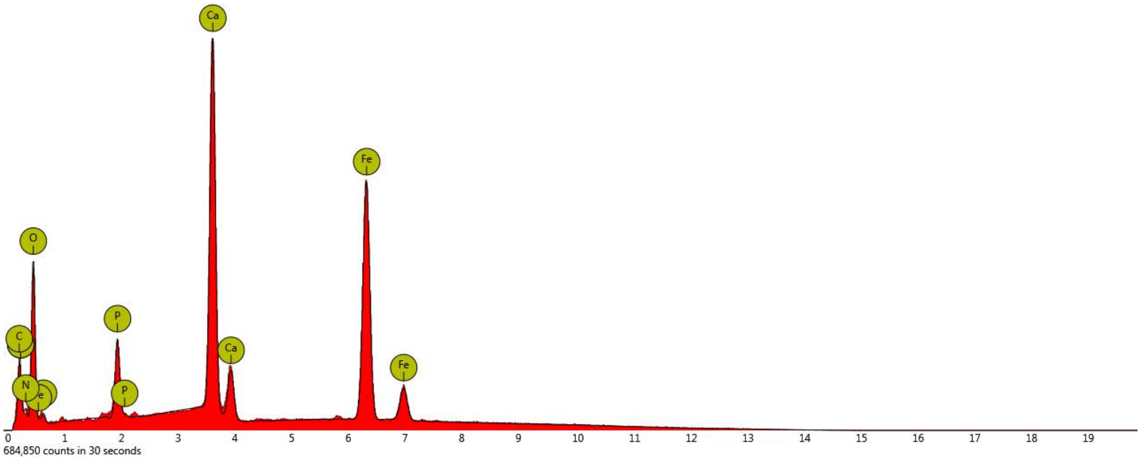

Disabled elements: B

3. spot

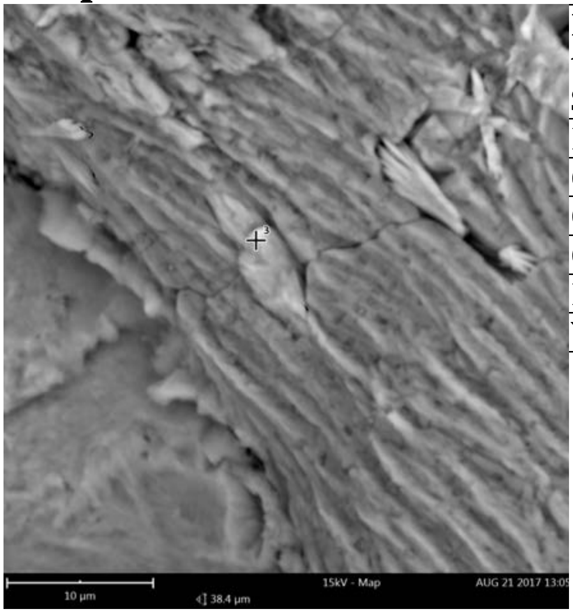

Element Symbol	Atomic Conc.	Weight Conc.	Oxide Symbol	Stoich. Conc.
Fe	18.23	37.48	Fe	28.82
Ca	17.29	25.52	Ca	27.34
O	36.77	21.67		
C	24.18	10.69	C	38.24
P	2.89	3.29	P	4.57
Y	0.30	0.98	Y	0.48

FOV: 38.4 µm, Mode: 15kV - Map, Detector: BSD Full, Time: AUG 21 2017 13:05

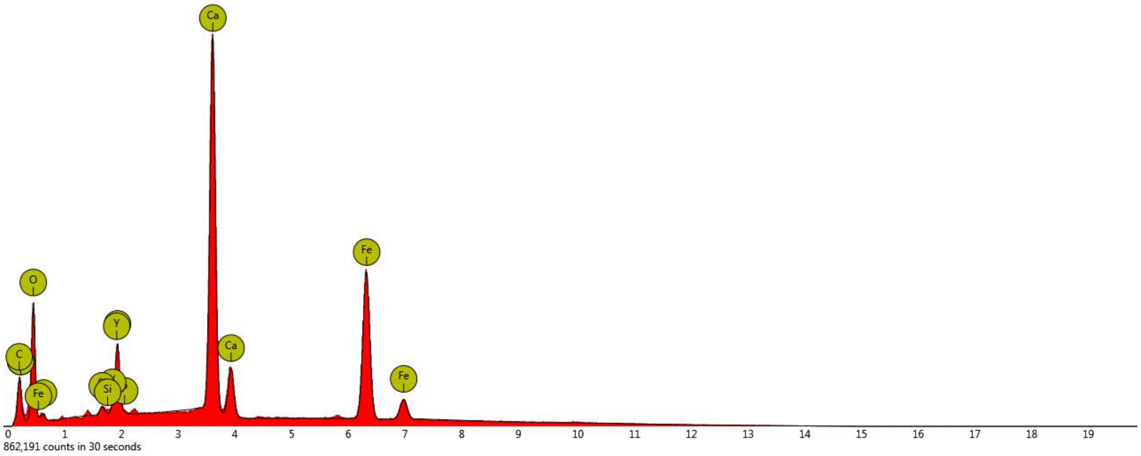

Disabled elements: B

4. spot

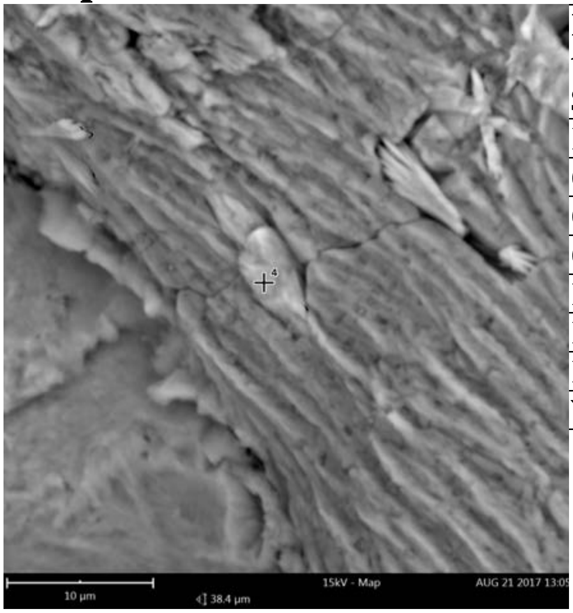

Element Symbol	Atomic Conc.	Weight Conc.	Oxide Symbol	Stoich. Conc.
Fe	22.15	45.77	Fe	31.77
O	30.28	17.93		
Ca	10.74	15.93	Ca	15.41
C	24.03	10.68	C	34.47
N	10.15	5.26	N	14.56
P	2.18	2.49	P	3.12
I	0.28	1.31	I	0.40
Y	0.19	0.62	Y	0.27

FOV: 38.4 µm, Mode: 15kV - Map, Detector: BSD Full, Time: AUG 21 2017 13:05

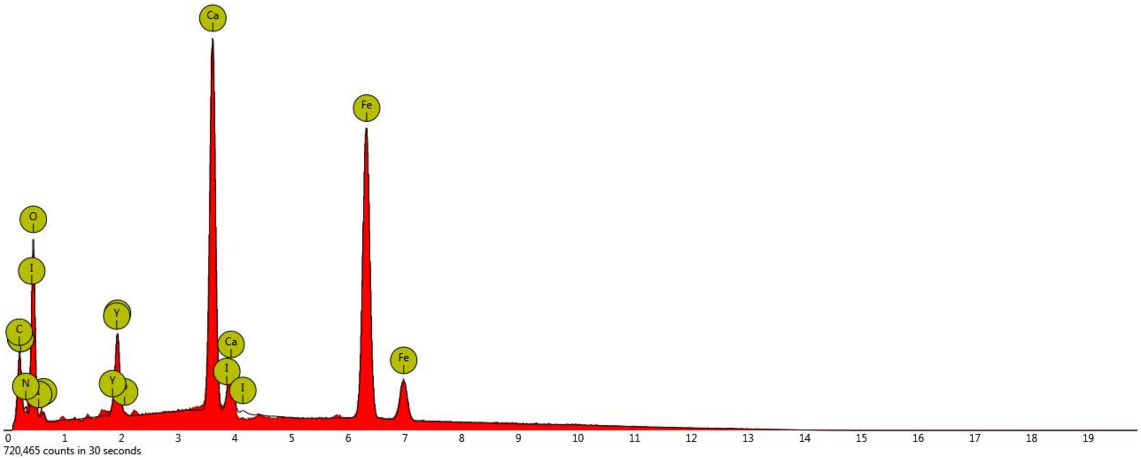

Disabled elements: B

5. spot

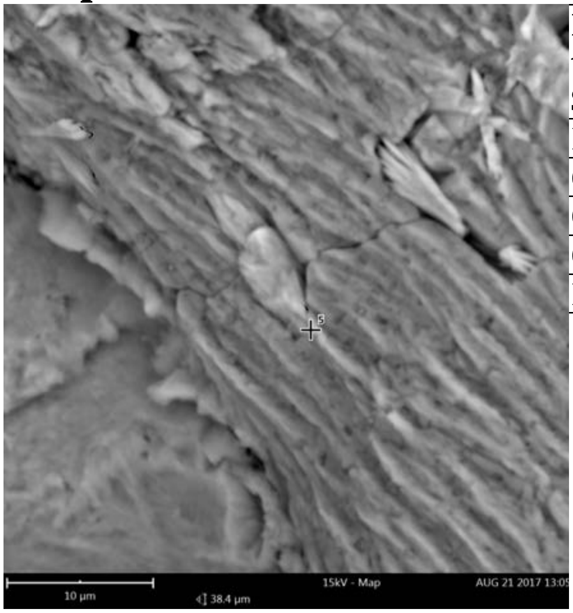

Element Symbol	Atomic Conc.	Weight Conc.	Oxide Symbol	Stoich. Conc.
Fe	13.43	30.32	Fe	23.16
Ca	17.19	27.85	Ca	29.64
O	42.02	27.18		
C	25.59	12.43	C	44.13
P	1.78	2.22	P	3.06

FOV: 38.4 µm, Mode: 15kV - Map, Detector: BSD Full, Time: AUG 21 2017 13:05

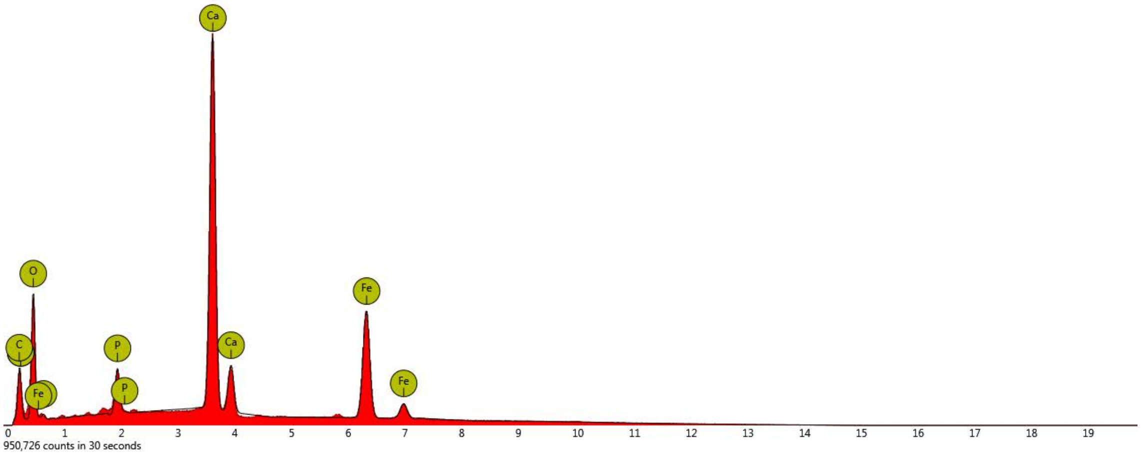

Disabled elements: B

6. spot

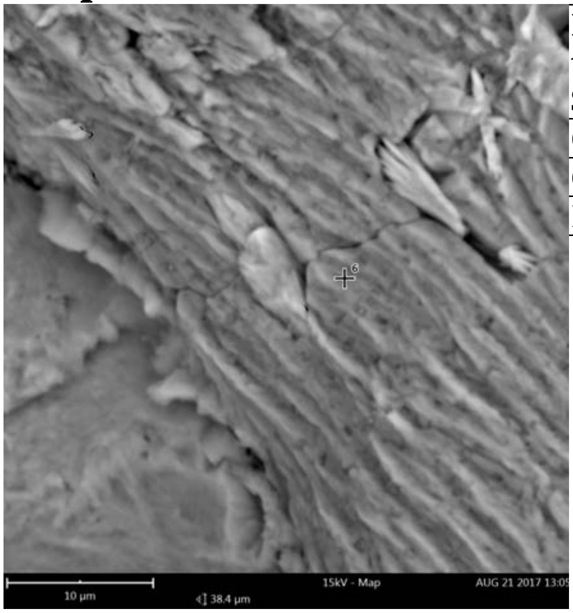

Element Symbol	Atomic Conc.	Weight Conc.	Oxide Symbol	Stoich. Conc.
Ca	36.87	58.78	Ca	95.43
O	61.36	39.05		
P	1.76	2.17	P	4.57

FOV: 38.4 µm, Mode: 15kV - Map, Detector: BSD Full, Time: AUG 21 2017 13:05

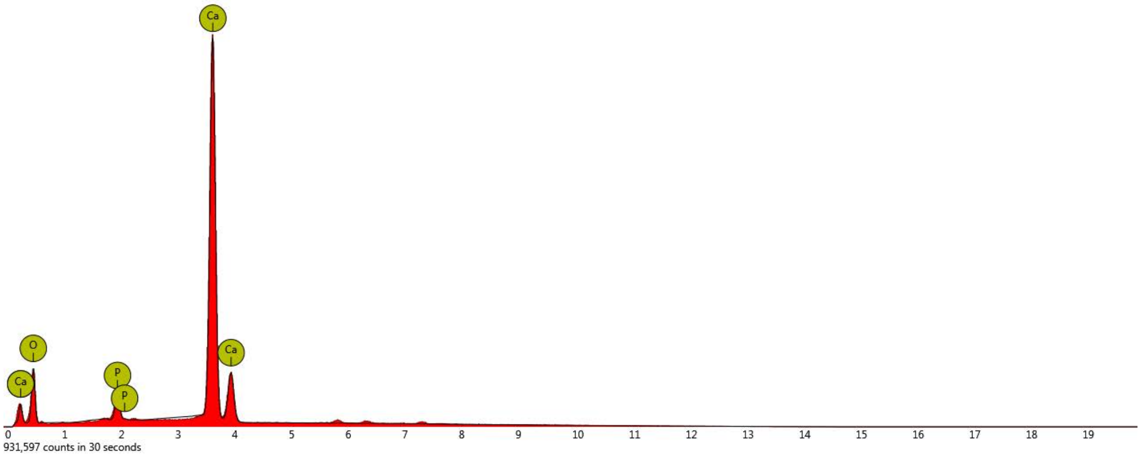

Disabled elements: B

7. spot

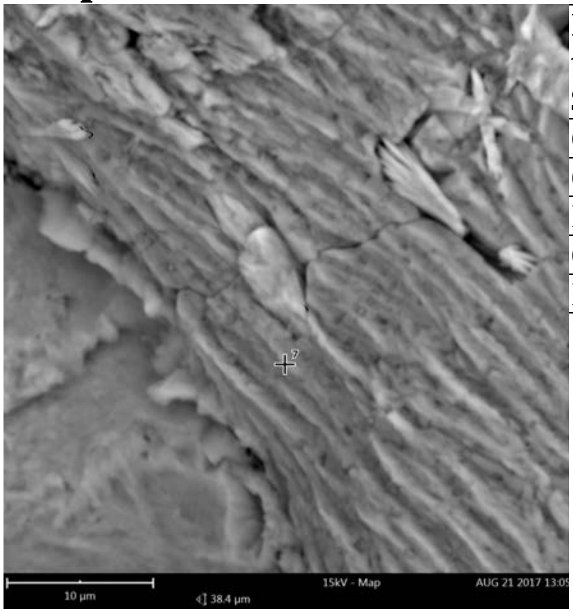

Element Symbol	Atomic Conc.	Weight Conc.	Oxide Symbol	Stoich. Conc.
Ca	24.10	41.66	Ca	48.70
O	50.51	34.86		
Fe	4.68	11.27	Fe	9.46
C	18.90	9.79	C	38.19
P	1.81	2.42	P	3.66

FOV: 38.4 µm, Mode: 15kV - Map, Detector: BSD Full, Time: AUG 21 2017 13:05

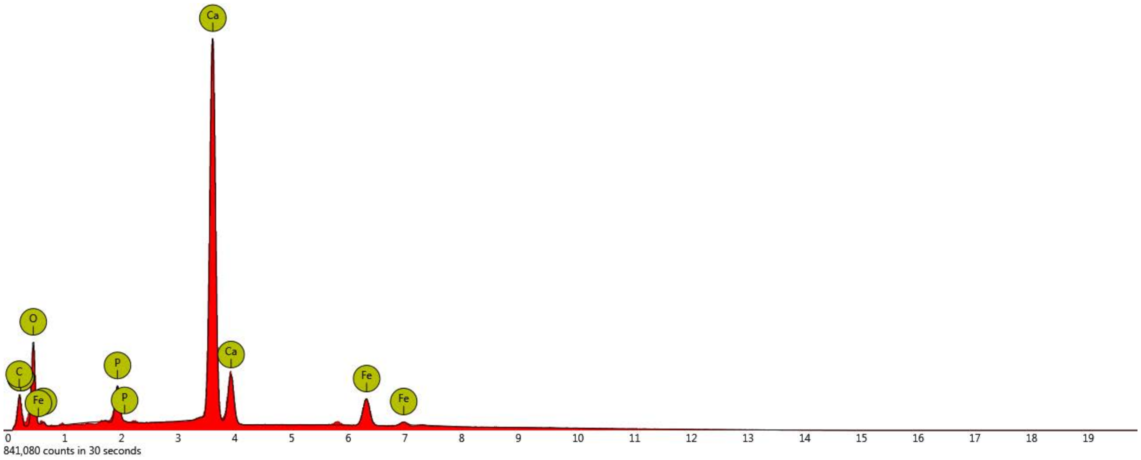

Disabled elements: B

Osteocytes embedded in bone 06

1. map

Combined map

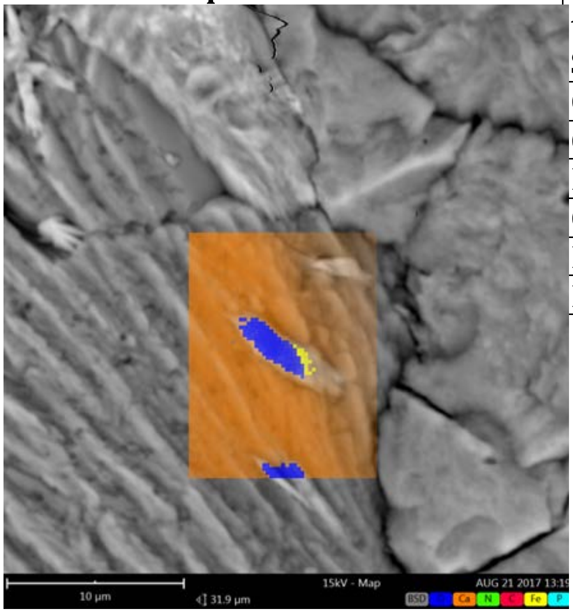

Element Symbol	Atomic Conc.	Weight Conc.	Oxide Symbol	Stoich. Conc.
O	54.56	47.98		
Ca	10.76	23.69	Ca	23.67
N	17.92	13.80	N	39.44
C	14.87	9.82	C	32.73
Fe	1.09	3.36	Fe	2.41
P	0.79	1.35	P	1.75

FOV: 31.9 µm, Mode: 15kV - Map, Detector: BSD Full, Time: AUG 21 2017 13:19

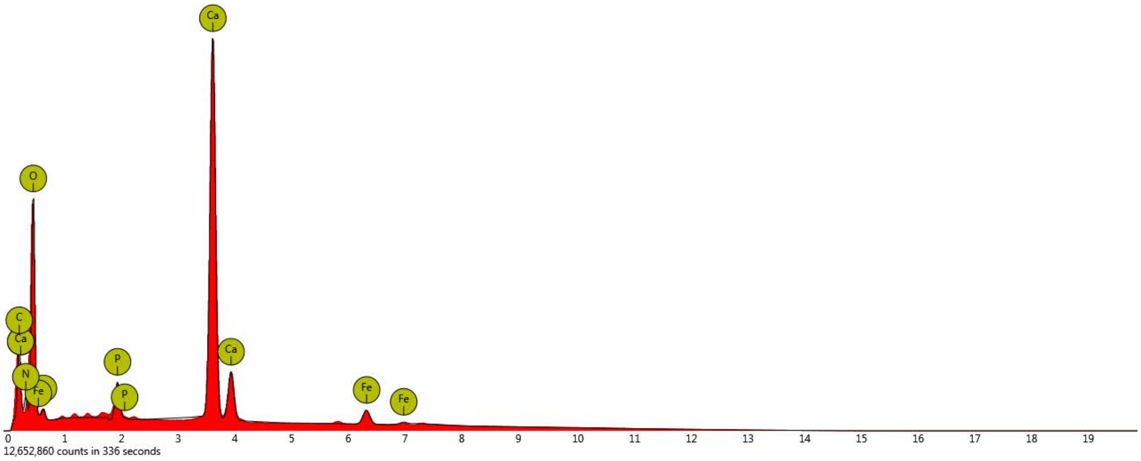

Disabled elements: B

Cut out of map (resolution: 49x64 pixels)

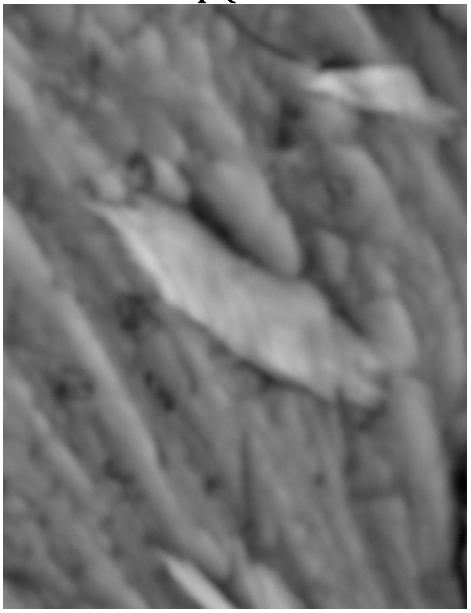

Oxygen

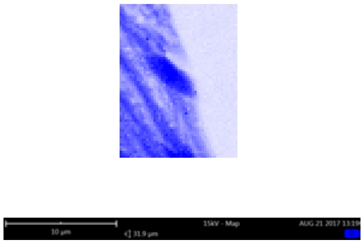

Calcium

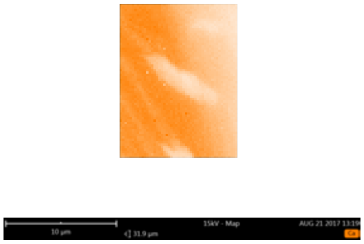

Nitrogen

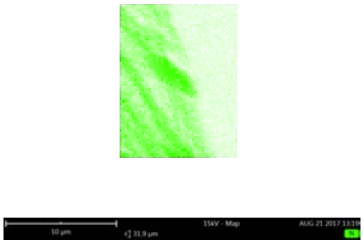

Carbon

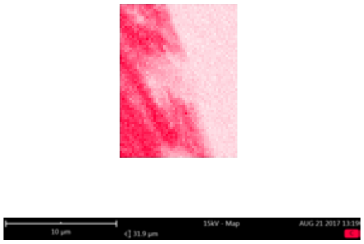

Iron

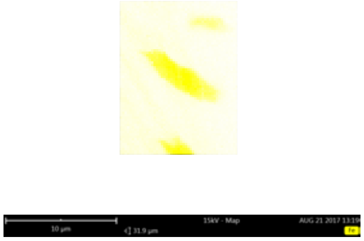

Phosphorus

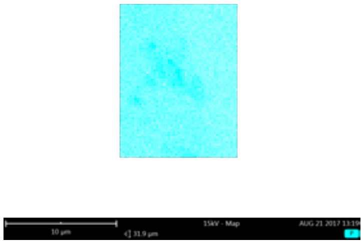

2. spot

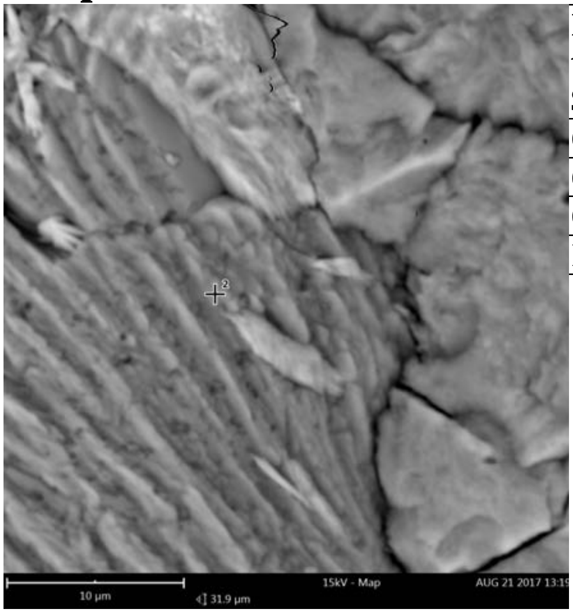

Element Symbol	Atomic Conc.	Weight Conc.	Oxide Symbol	Stoich. Conc.
O	58.90	49.44		
Ca	16.30	34.26	Ca	39.65
C	24.14	15.21	C	58.73
P	0.67	1.08	P	1.62

FOV: 31.9 µm, Mode: 15kV - Map, Detector: BSD Full, Time: AUG 21 2017 13:19

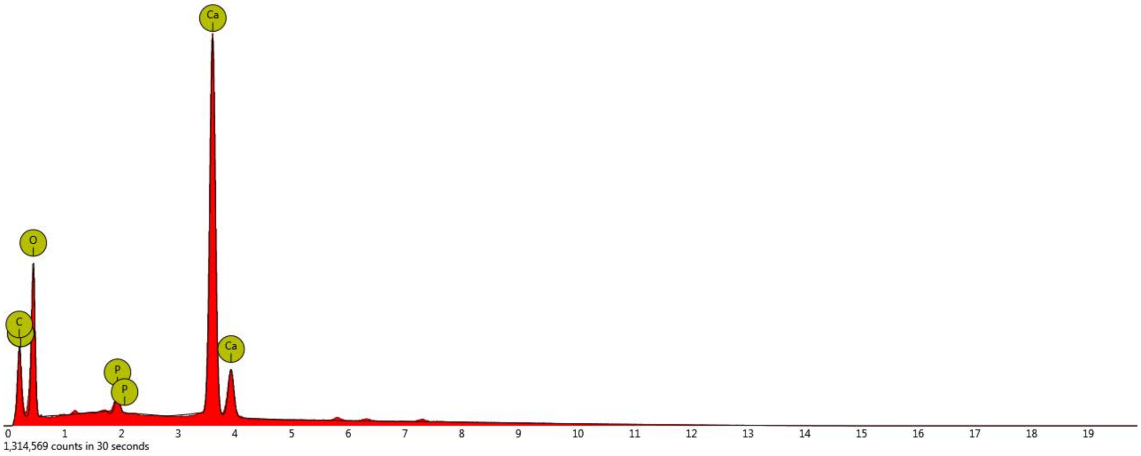

Disabled elements: B

3. spot

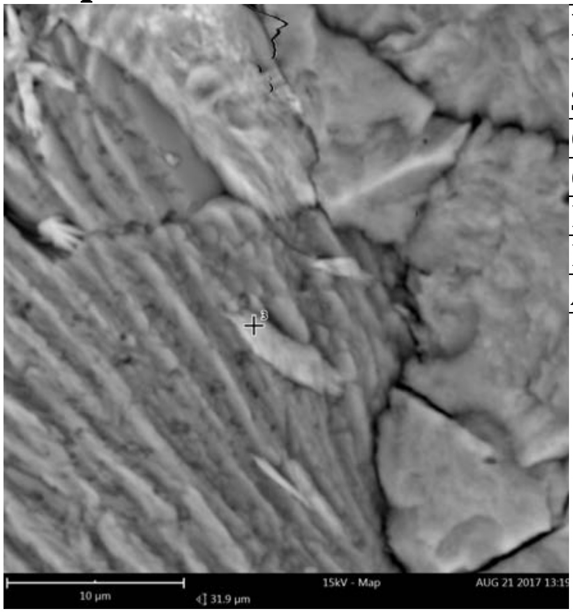

Element Symbol	Atomic Conc.	Weight Conc.	Oxide Symbol	Stoich. Conc.
O	81.03	60.63		
Ca	10.08	18.89	Ca	53.15
Fe	6.68	17.46	Fe	35.24
P	1.26	1.83	P	6.66
Al	0.94	1.18	Al	4.94

FOV: 31.9 µm, Mode: 15kV - Map, Detector: BSD Full, Time: AUG 21 2017 13:19

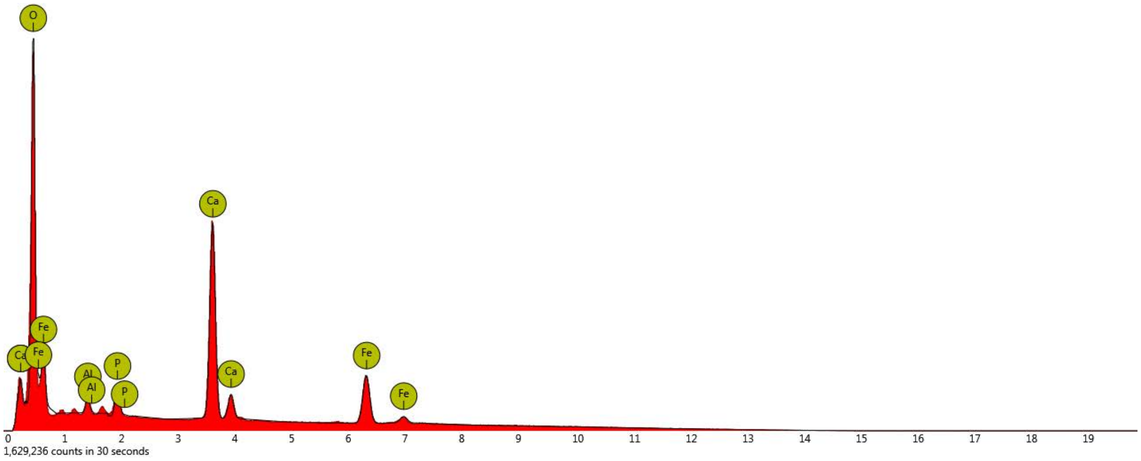

Disabled elements: B, Sb

4. spot

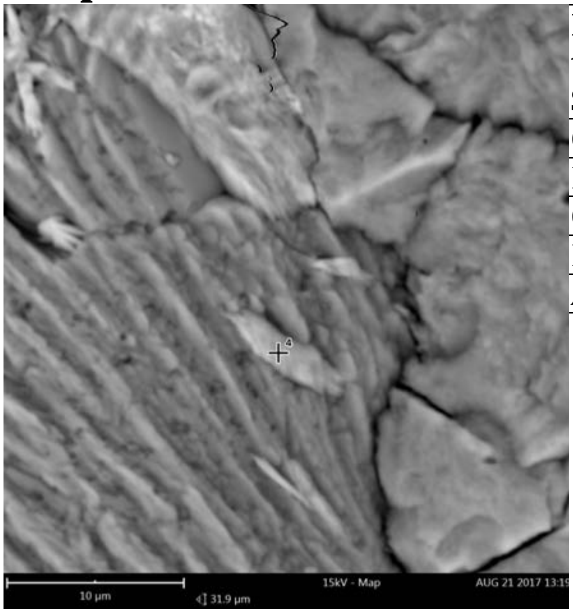

Element Symbol	Atomic Conc.	Weight Conc.	Oxide Symbol	Stoich. Conc.
O	79.13	57.19		
Fe	8.91	22.47	Fe	42.68
Ca	9.30	16.84	Ca	44.57
P	1.42	1.99	P	6.81
Al	1.24	1.51	Al	5.94

FOV: 31.9 µm, Mode: 15kV - Map, Detector: BSD Full, Time: AUG 21 2017 13:19

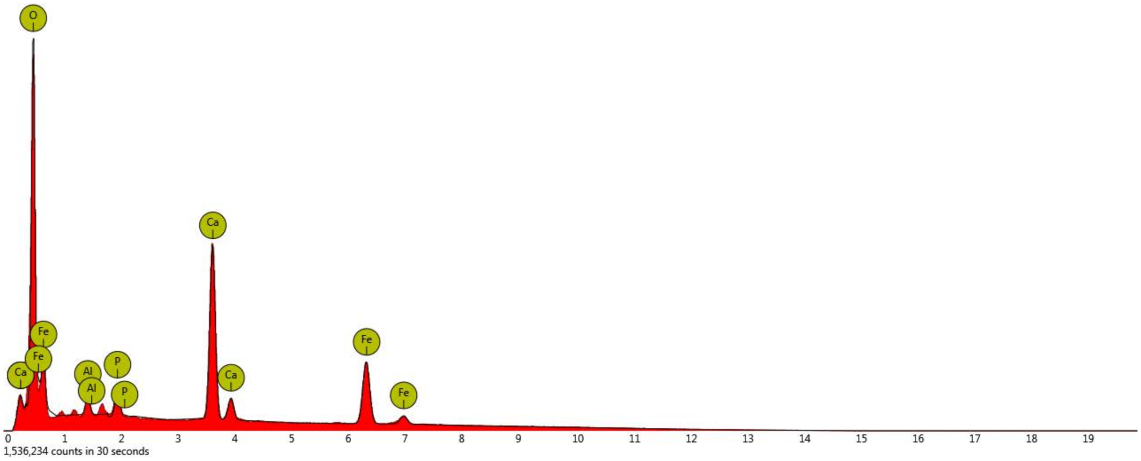

Disabled elements: B

5. spot

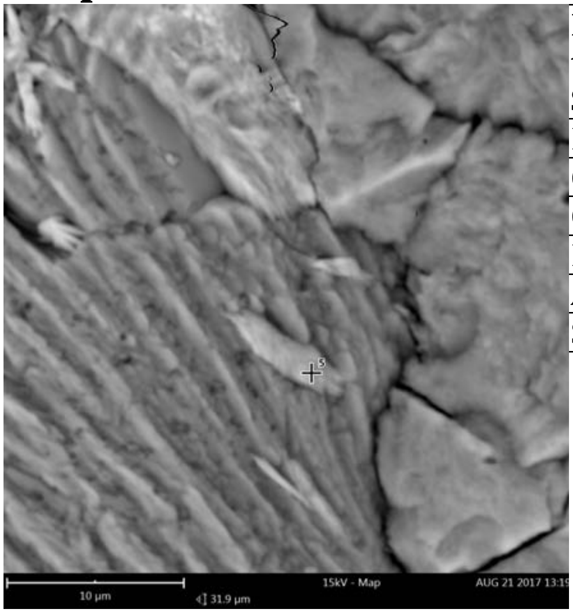

Element Symbol	Atomic Conc.	Weight Conc.	Oxide Symbol	Stoich. Conc.
Fe	19.94	39.09	Fe	48.32
O	58.72	32.98		
Ca	15.72	22.11	Ca	38.07
P	3.33	3.62	P	8.08
Al	1.41	1.33	Al	3.41
Si	0.88	0.86	Si	2.12

FOV: 31.9 μm, Mode: 15kV - Map, Detector: BSD Full, Time: AUG 21 2017 13:19

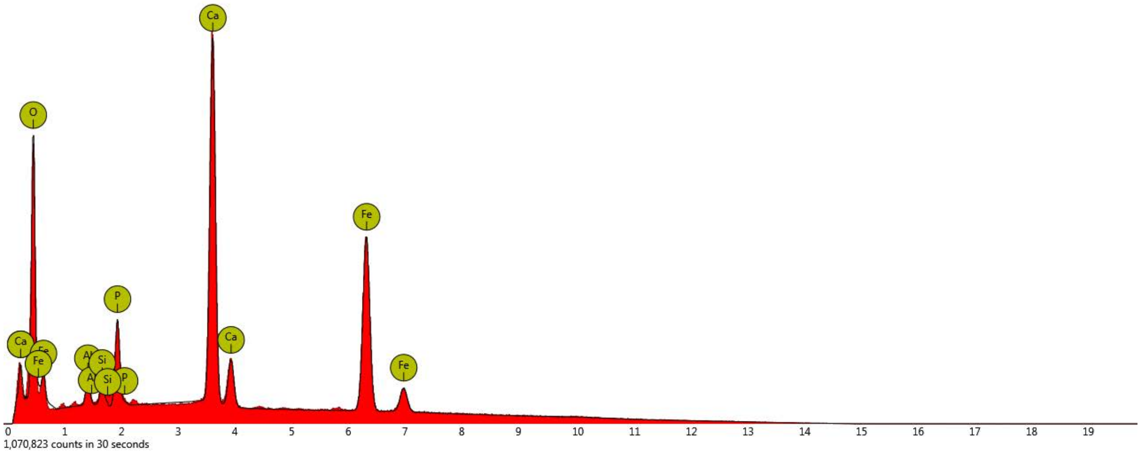

Disabled elements: B, Br, Sr, Y

6. spot

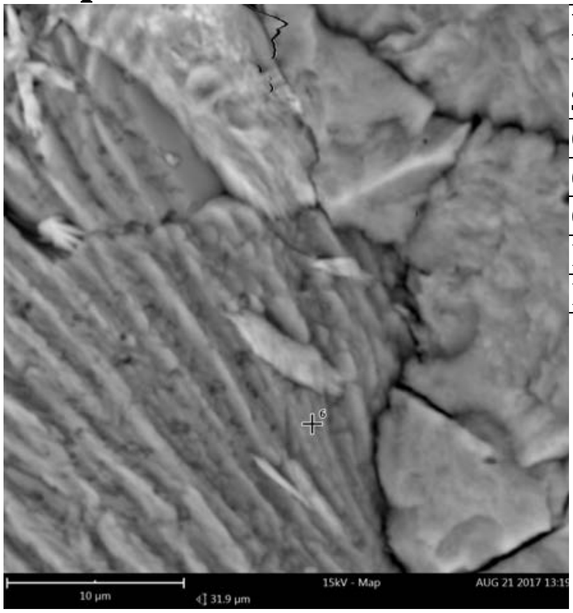

Element Symbol	Atomic Conc.	Weight Conc.	Oxide Symbol	Stoich. Conc.
O	56.52	42.08		
Ca	21.92	40.88	Ca	50.42
C	18.29	10.22	C	42.06
Fe	1.83	4.75	Fe	4.21
P	1.44	2.08	P	3.31

FOV: 31.9 µm, Mode: 15kV - Map, Detector: BSD Full, Time: AUG 21 2017 13:19

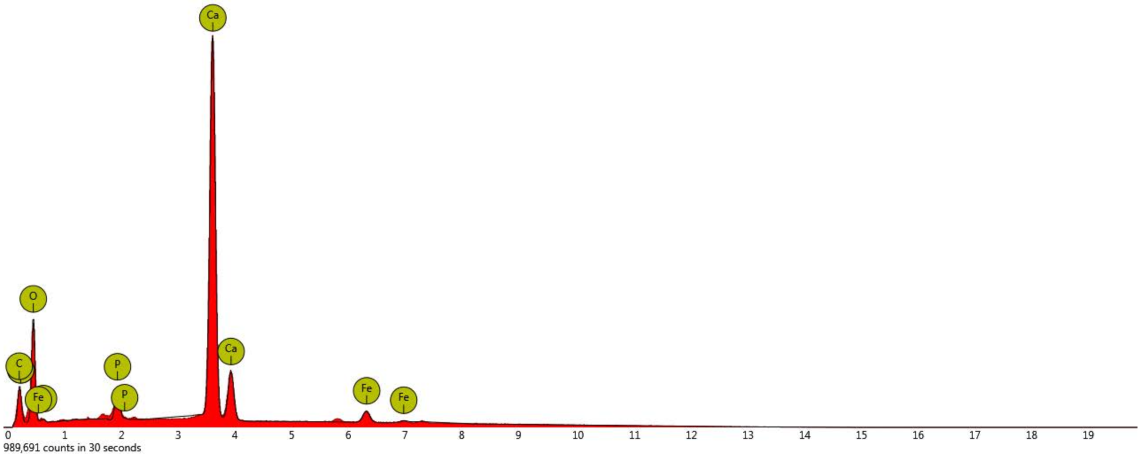

Disabled elements: B, Y

Osteocytes embedded in bone 07

1. map

Combined map

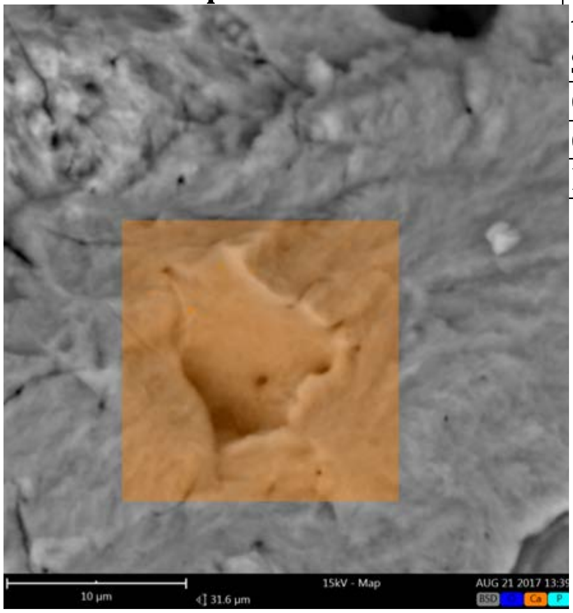

Element Symbol	Atomic Conc.	Weight Conc.	Oxide Symbol	Stoich. Conc.
O	76.70	58.68		
Ca	15.67	30.02	Ca	67.25
P	7.63	11.30	P	32.75

FOV: 31.6 µm, Mode: 15kV - Map, Detector: BSD Full, Time: AUG 21 2017 13:39

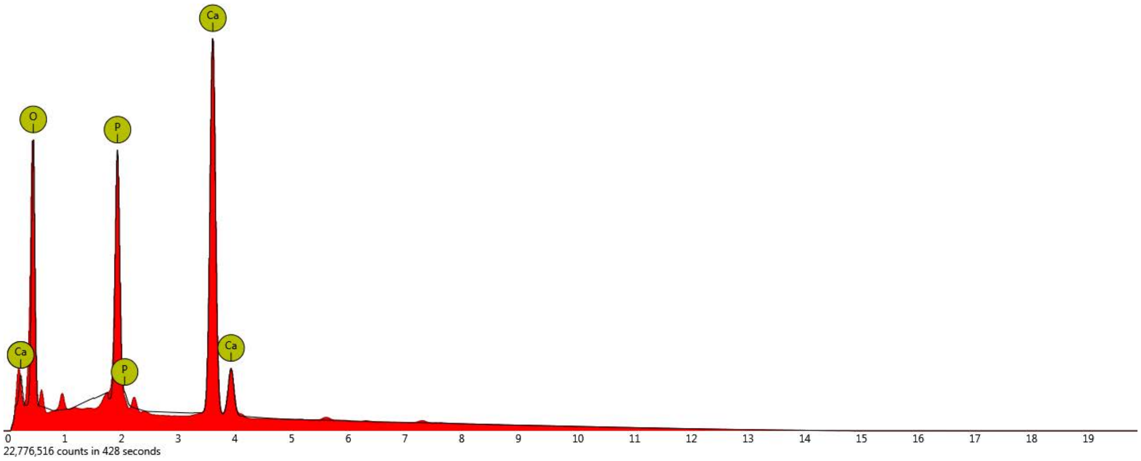

Disabled elements: B

Cut out of map (resolution: 63x64 pixels)

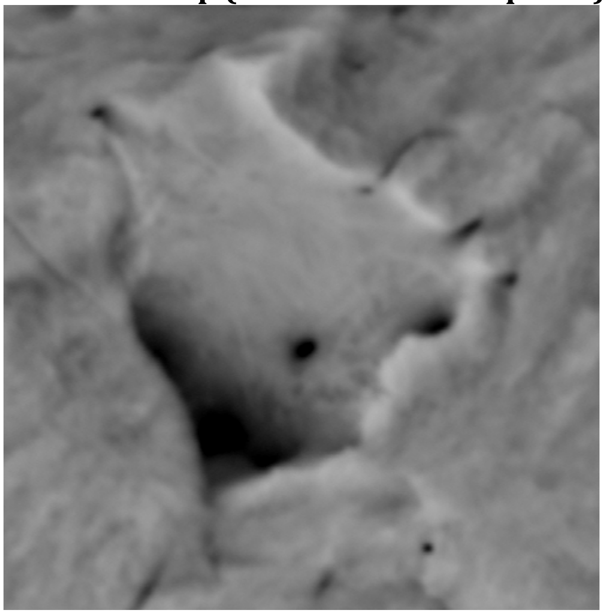

Oxygen

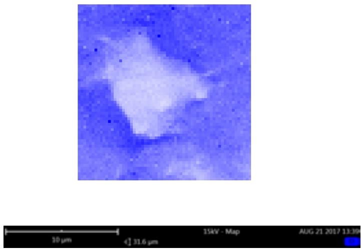

Calcium

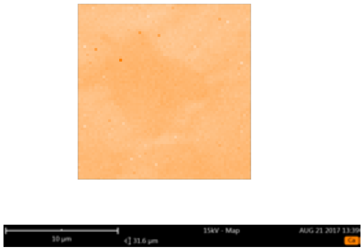

Phosphorus

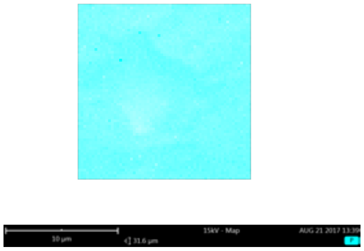

2. spot

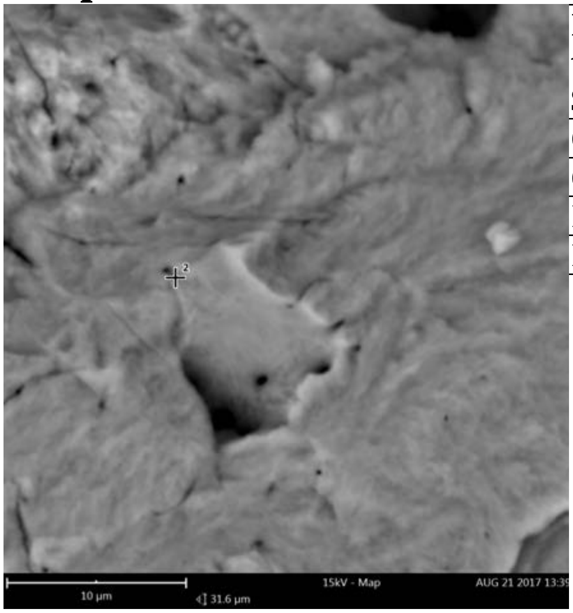

Element Symbol	Atomic Conc.	Weight Conc.	Oxide Symbol	Stoich. Conc.
O	68.95	49.30		
Ca	20.16	36.12	Ca	64.93
P	9.51	13.17	P	30.63
Na	1.38	1.42	Na	4.44

FOV: 31.6 μm, Mode: 15kV - Map, Detector: BSD Full, Time: AUG 21 2017 13:39

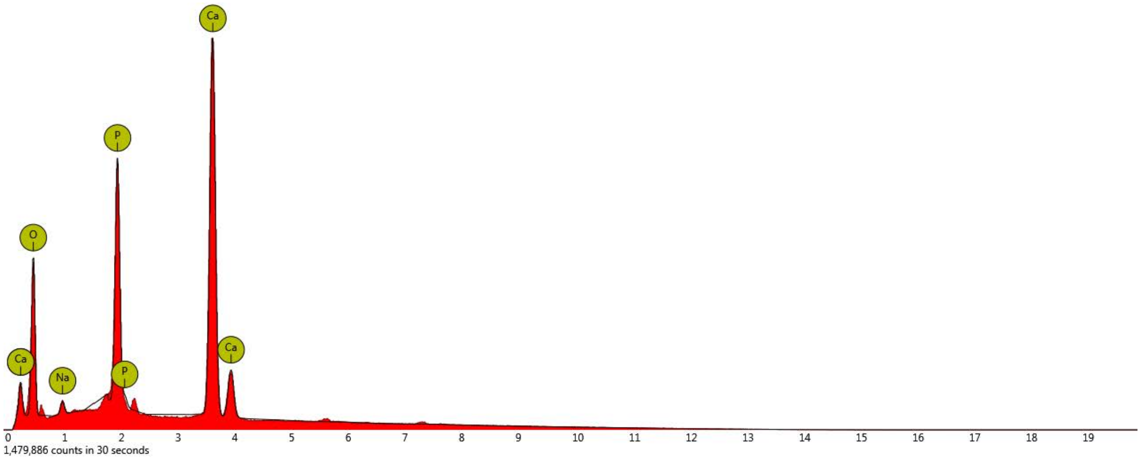

Disabled elements: B, Sb, Y

3. spot

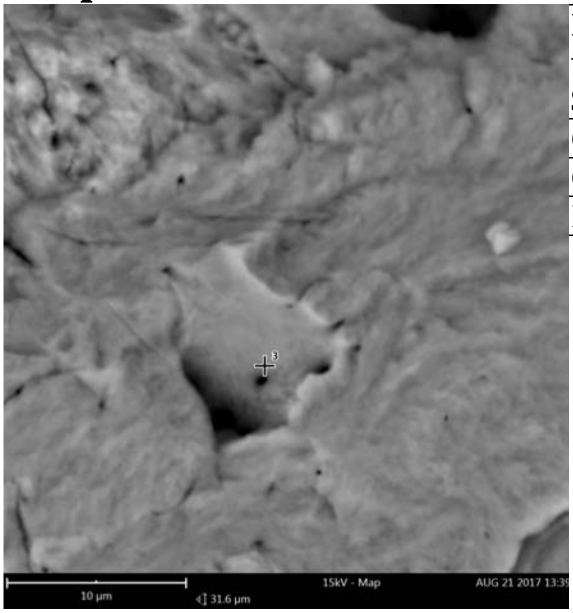

Element Symbol	Atomic Conc.	Weight Conc.	Oxide Symbol	Stoich. Conc.
Ca	28.30	46.51	Ca	72.97
O	61.22	40.17		
P	10.48	13.31	P	27.03

FOV: 31.6 µm, Mode: 15kV - Map, Detector: BSD Full, Time: AUG 21 2017 13:39

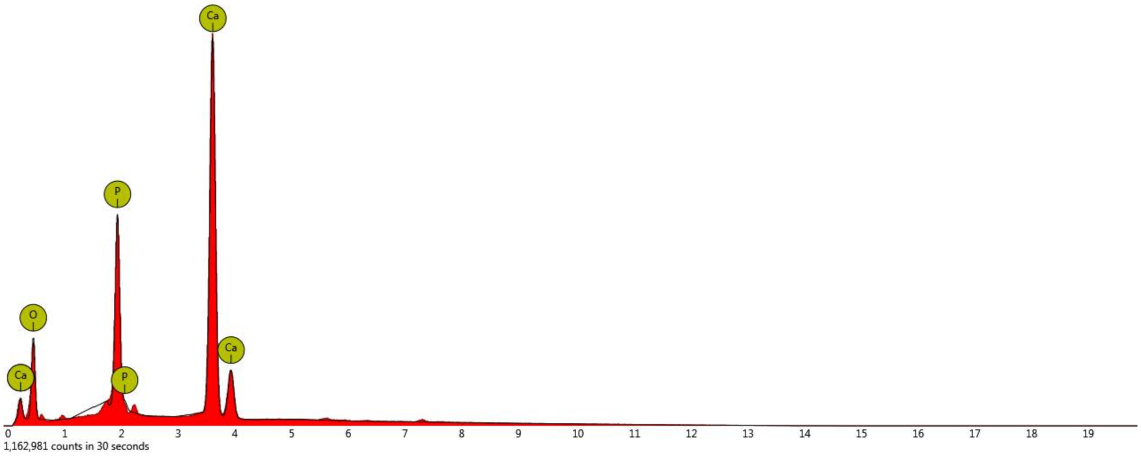

Disabled elements: B, Sr, Y

4. spot

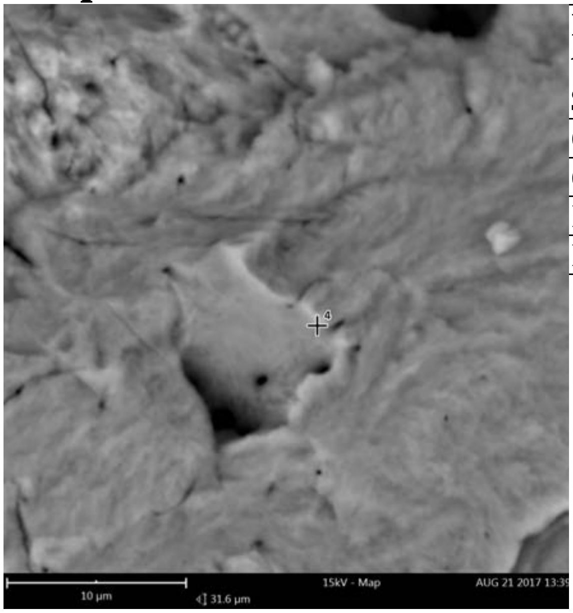

Element Symbol	Atomic Conc.	Weight Conc.	Oxide Symbol	Stoich. Conc.
O	70.70	51.40		
Ca	18.96	34.53	Ca	64.71
P	9.03	12.71	P	30.82
Na	1.31	1.37	Na	4.48

FOV: 31.6 µm, Mode: 15kV - Map, Detector: BSD Full, Time: AUG 21 2017 13:39

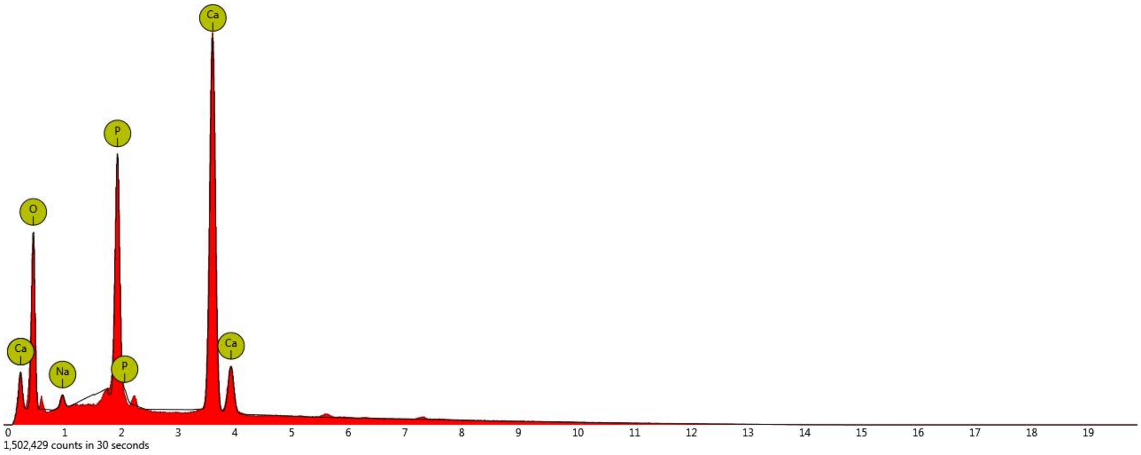

Disabled elements: B, Y

5. spot

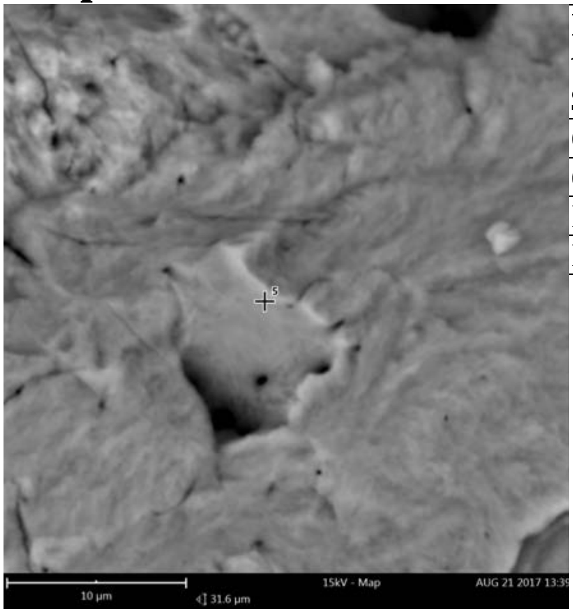

Element Symbol	Atomic Conc.	Weight Conc.	Oxide Symbol	Stoich. Conc.
O	71.15	51.97		
Ca	18.60	34.04	Ca	64.48
P	8.87	12.54	P	30.74
Na	1.38	1.45	Na	4.78

FOV: 31.6 µm, Mode: 15kV - Map, Detector: BSD Full, Time: AUG 21 2017 13:39

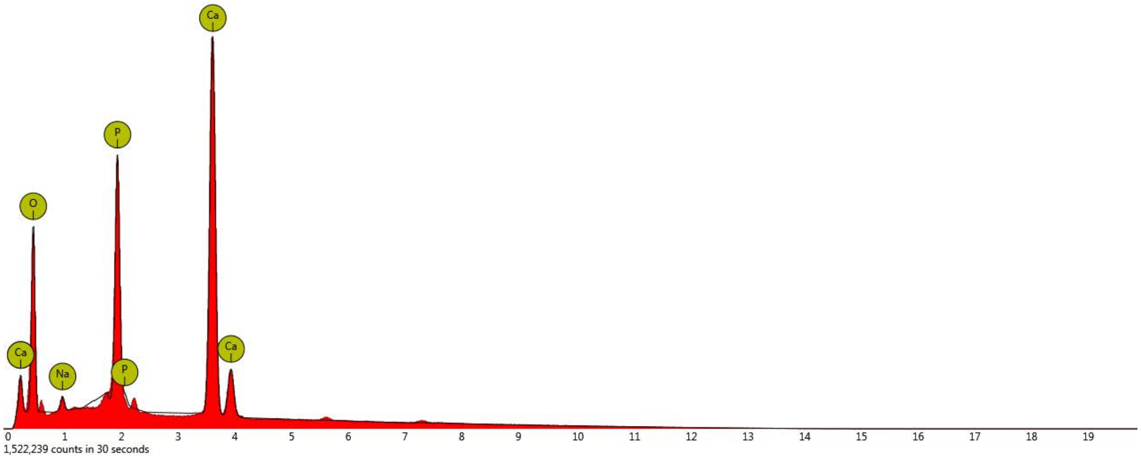

Disabled elements: B, Sb, Y

6. spot

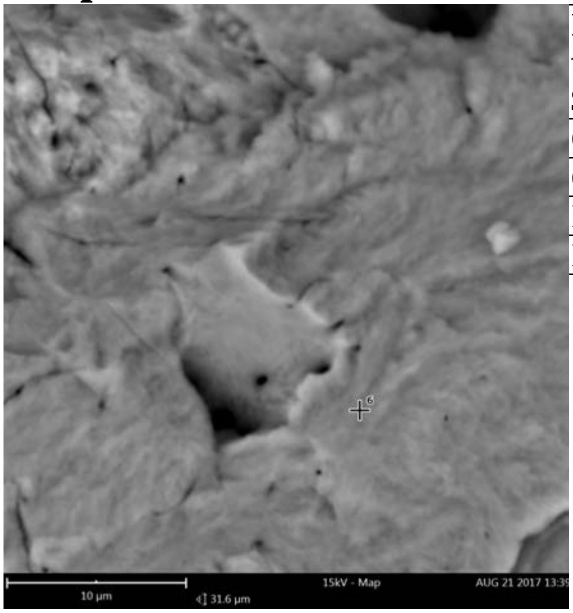

Element Symbol	Atomic Conc.	Weight Conc.	Oxide Symbol	Stoich. Conc.
O	69.74	50.22		
Ca	19.68	35.50	Ca	65.04
P	9.27	12.92	P	30.62
Na	1.32	1.36	Na	4.35

FOV: 31.6 μm, Mode: 15kV - Map, Detector: BSD Full, Time: AUG 21 2017 13:39

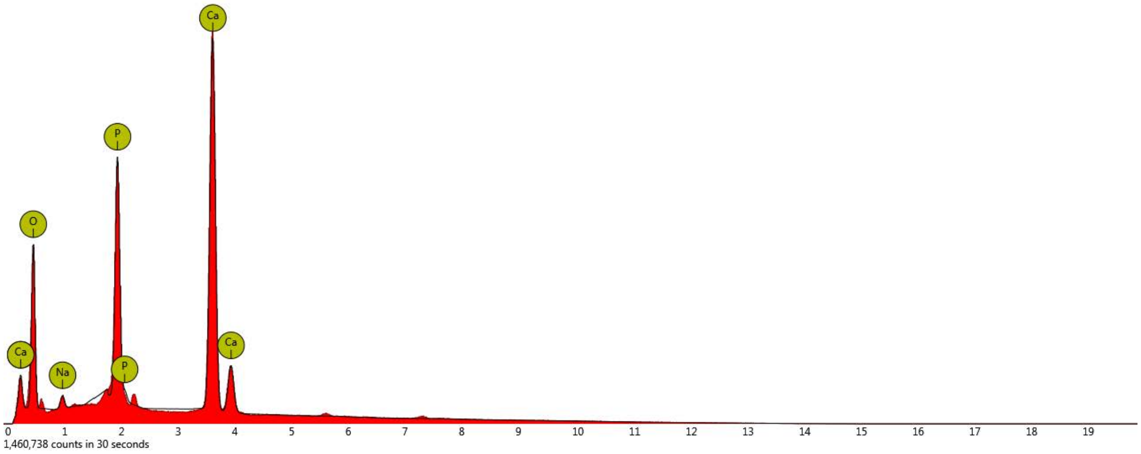

Disabled elements: B, Sr, Y

Isolated osteocytes 01

1. map

Combined map

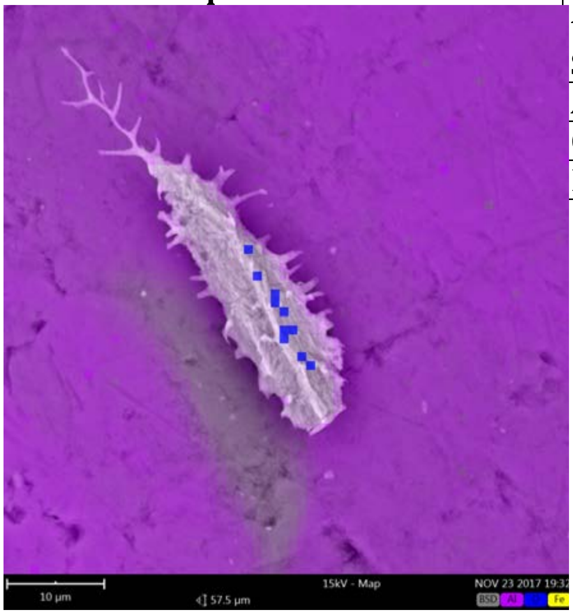

Element Symbol	Atomic Conc.	Weight Conc.	Oxide Symbol	Stoich. Conc.
Al	78.07	83.14	Al	97.61
O	20.02	12.64		
Fe	1.91	4.22	Fe	2.39

FOV: 57.5 µm, Mode: 15kV - Map, Detector: BSD Full, Time: NOV 23 2017 19:32

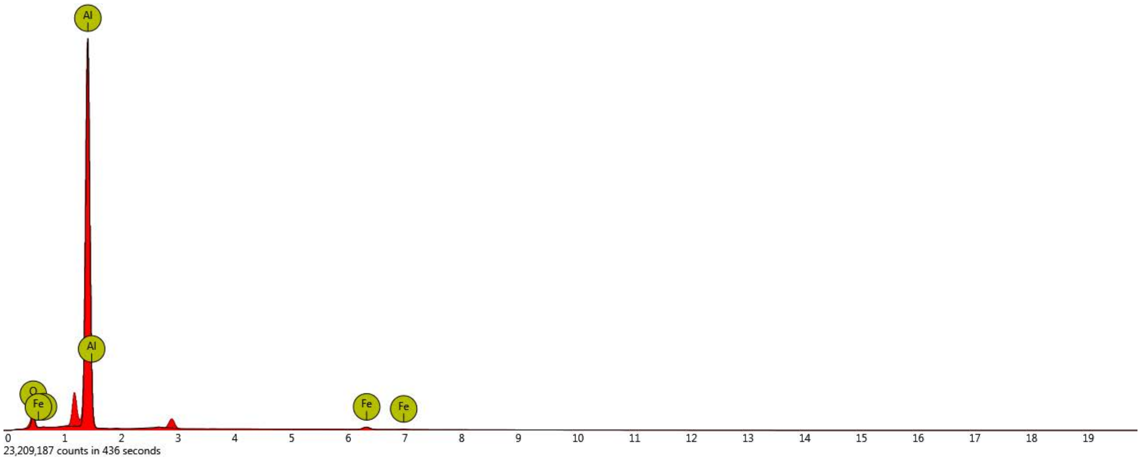

Disabled elements: Ac, Ag, As, B, Mg, Se, Th

Cut out of map (resolution: 64x64 pixels)

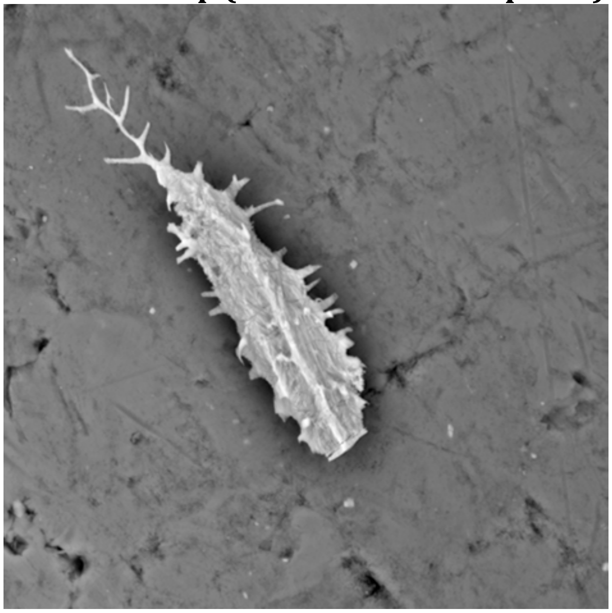

Aluminium

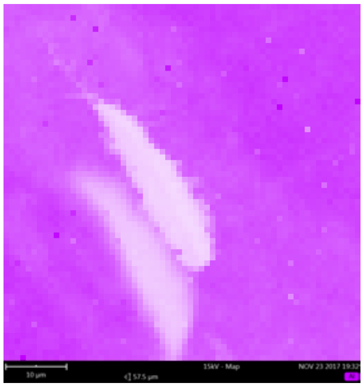

Oxygen

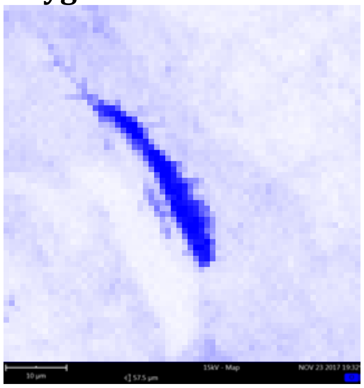

Iron

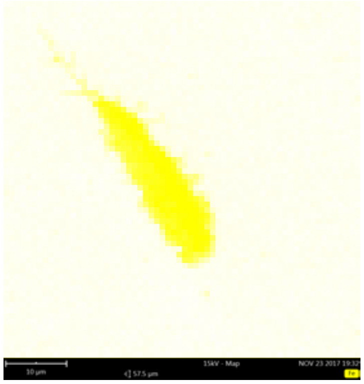

Image 4
1. spot

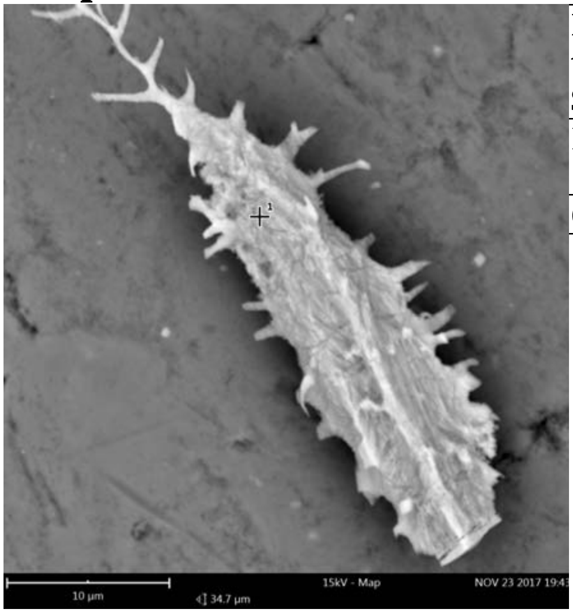

Element Symbol	Atomic Conc.	Weight Conc.	Oxide Symbol	Stoich. Conc.
Fe	69.66	88.91	Fe	100.00
O	30.34	11.09		

FOV: 34.7 µm, Mode: 15kV - Map, Detector: BSD Full, Time: NOV 23 2017 19:43

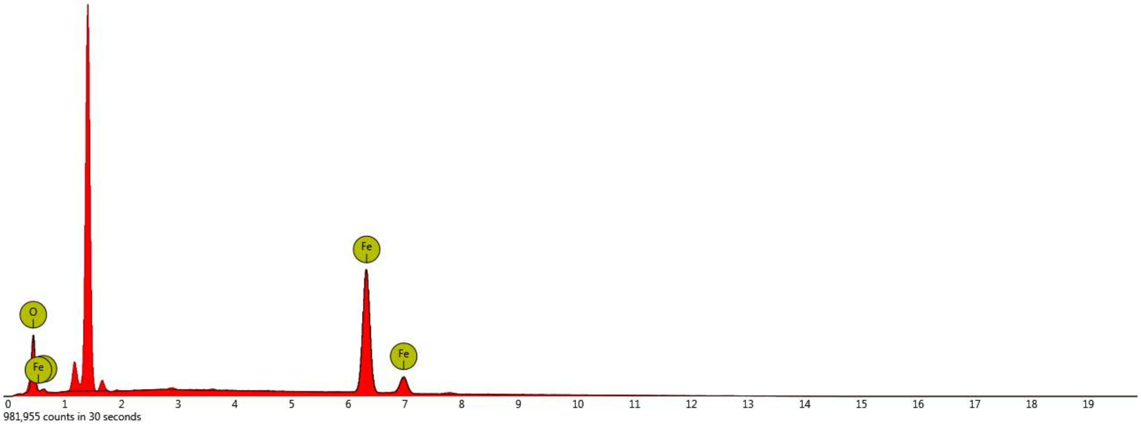

Disabled elements: Al, B, Br, Er, Se, Tm

2. spot

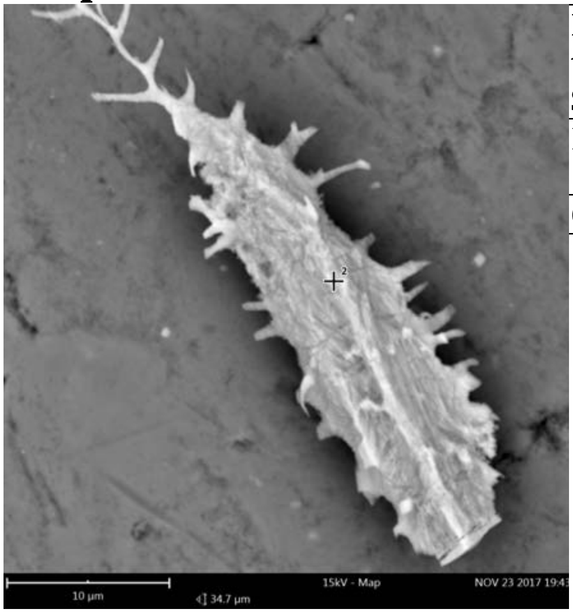

Element Symbol	Atomic Conc.	Weight Conc.	Oxide Symbol	Stoich. Conc.
Fe	30.51	60.52	Fe	100.00
O	69.49	39.48		

FOV: 34.7 µm, Mode: 15kV - Map, Detector: BSD Full, Time: NOV 23 2017 19:43

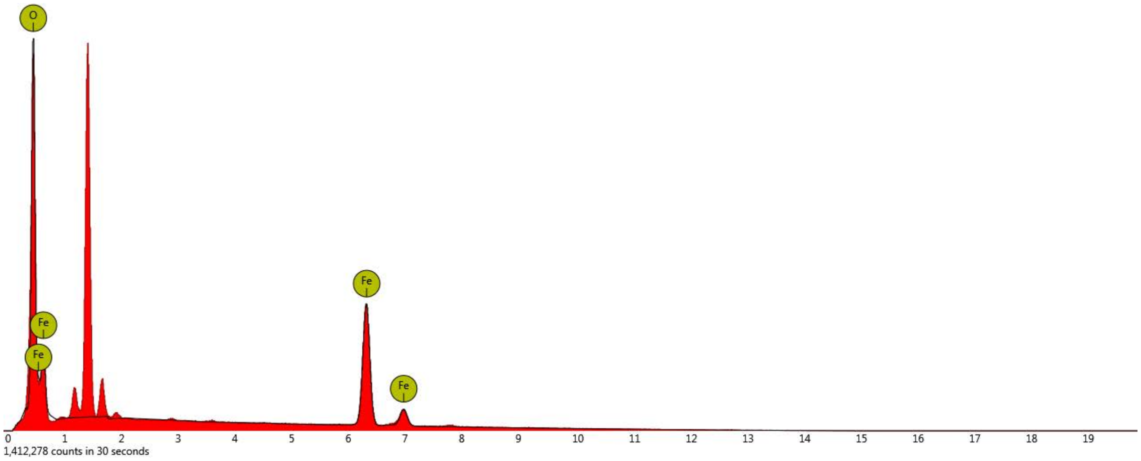

Disabled elements: Al, B, Br, Er, Se, Tm

3. spot

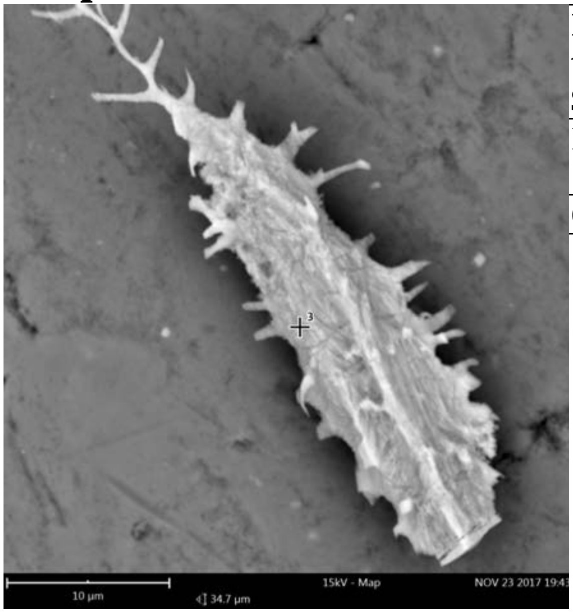

Element Symbol	Atomic Conc.	Weight Conc.	Oxide Symbol	Stoich. Conc.
Fe	83.05	94.48	Fe	100.00
O	16.95	5.52		

FOV: 34.7 µm, Mode: 15kV - Map, Detector: BSD Full, Time: NOV 23 2017 19:43

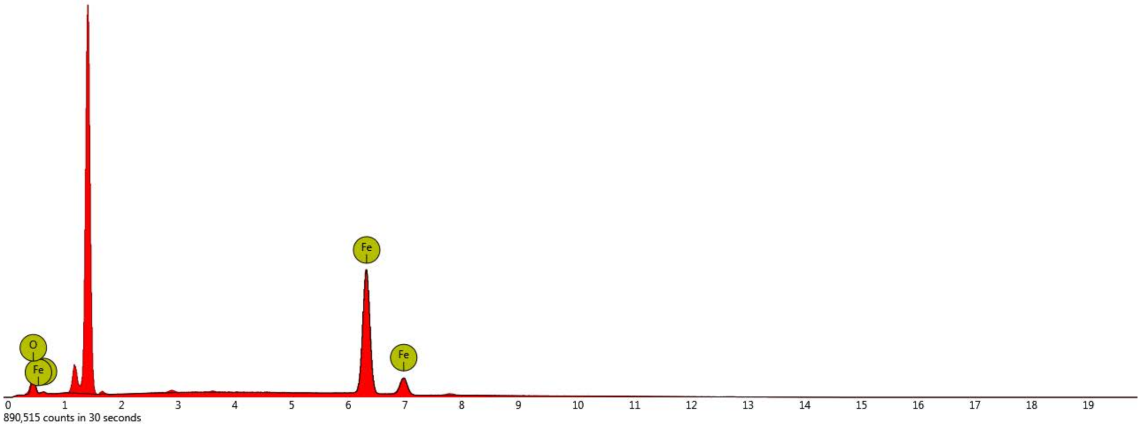

Disabled elements: Ag, Al, B, Br, Er, Se, Tm

4. spot

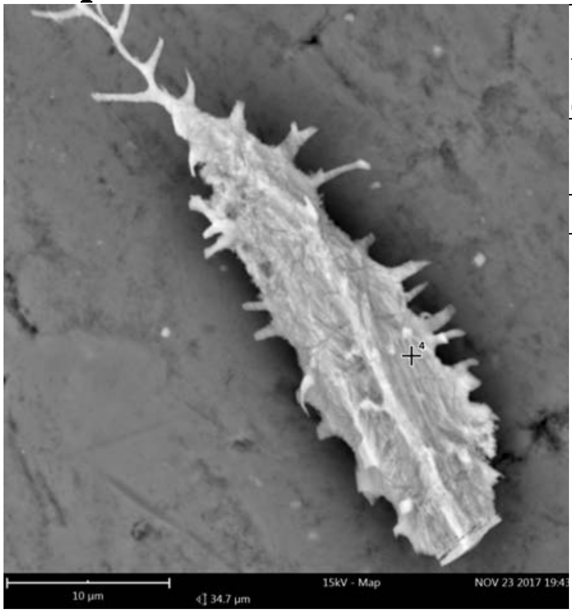

Element Symbol	Atomic Conc.	Weight Conc.	Oxide Symbol	Stoich. Conc.
Fe	30.46	60.46	Fe	100.00
O	69.54	39.54		

FOV: 34.7 µm, Mode: 15kV - Map, Detector: BSD Full, Time: NOV 23 2017 19:43

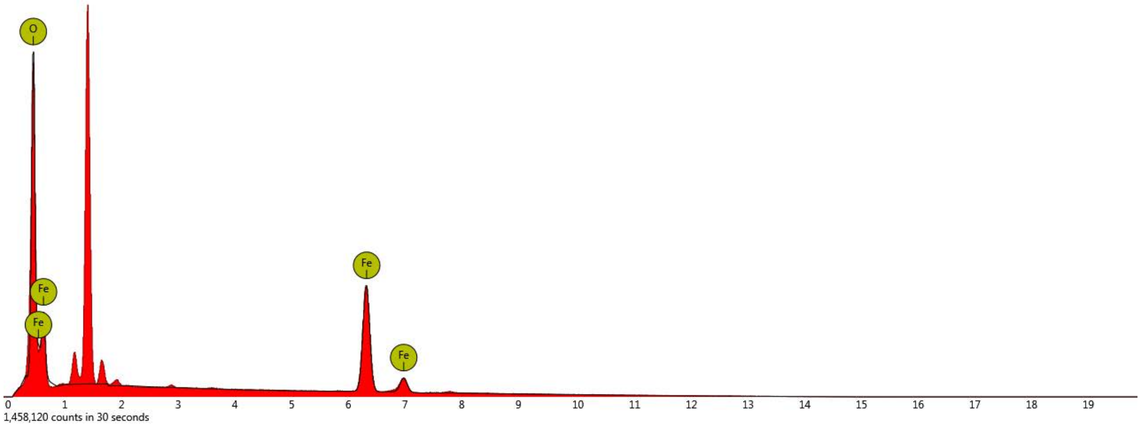

Disabled elements: Al, B, Br, Er, Tm

5. spot

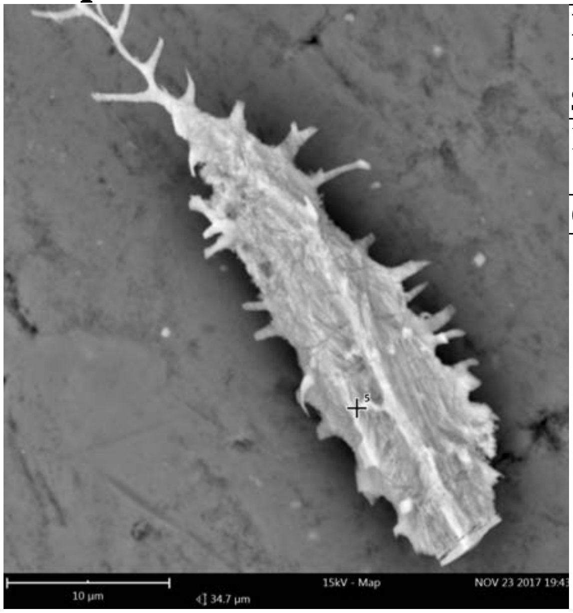

Element Symbol	Atomic Conc.	Weight Conc.	Oxide Symbol	Stoich. Conc.
Fe	44.06	73.32	Fe	100.00
O	55.94	26.68		

FOV: 34.7 µm, Mode: 15kV - Map, Detector: BSD Full, Time: NOV 23 2017 19:43

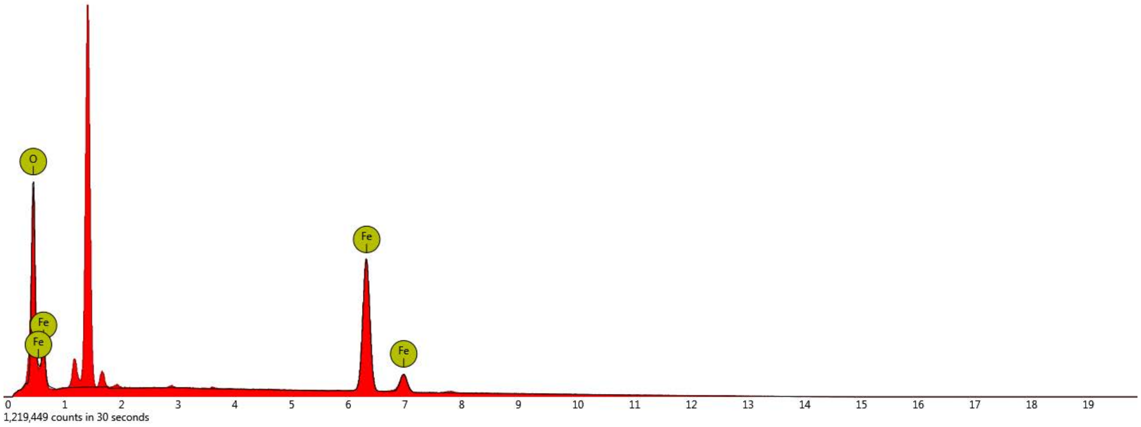

Disabled elements: Al, B, Br, Er, Se, Tm

6. spot

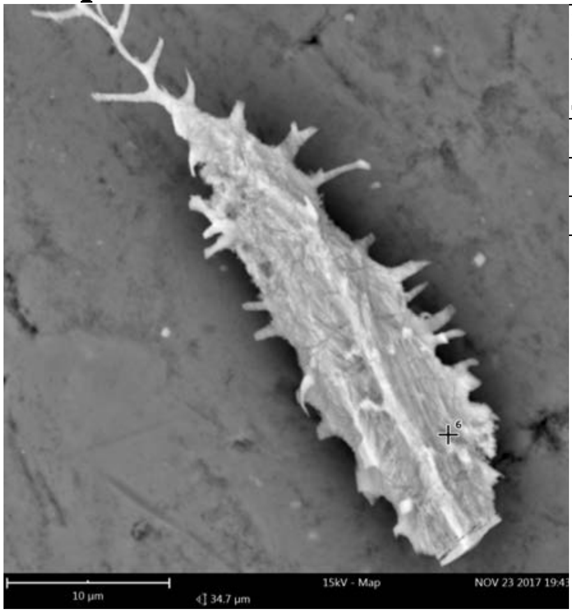

Element Symbol	Atomic Conc.	Weight Conc.	Oxide Symbol	Stoich. Conc.
Fe	31.08	60.56	Fe	93.14
O	66.63	37.20		
Si	2.29	2.24	Si	6.86

FOV: 34.7 μm, Mode: 15kV - Map, Detector: BSD Full, Time: NOV 23 2017 19:43

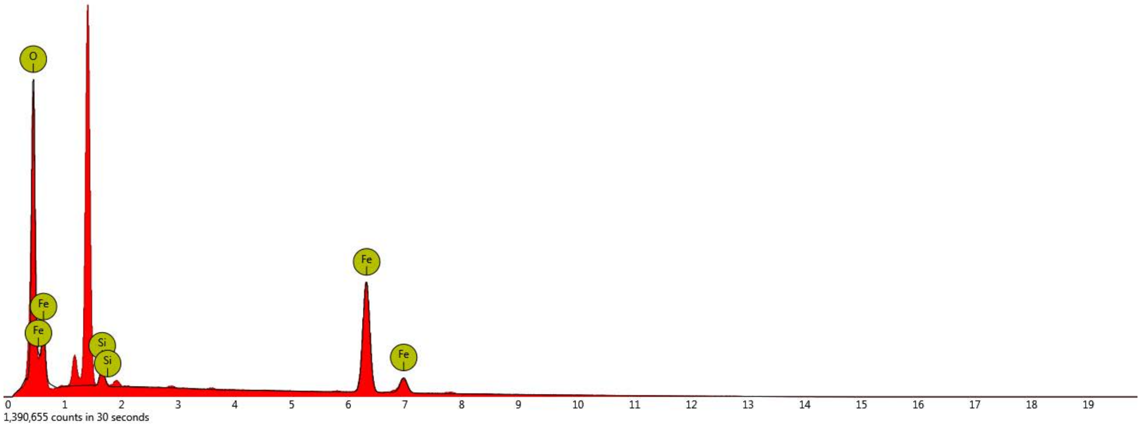

Disabled elements: Al, B, Br, Er, Tm

7. spot

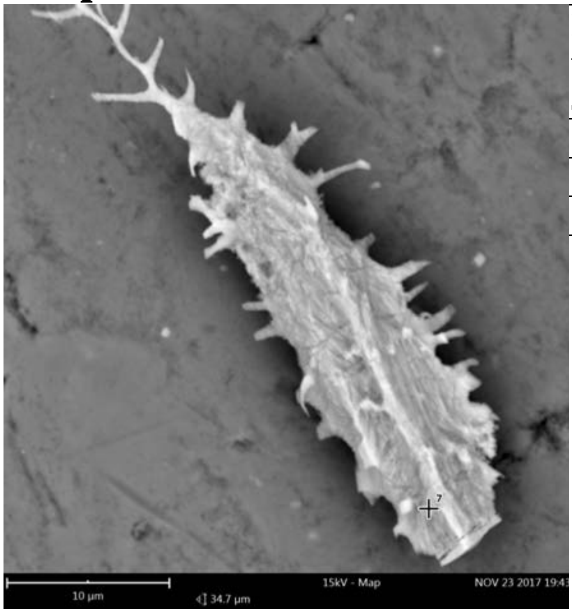

Element Symbol	Atomic Conc.	Weight Conc.	Oxide Symbol	Stoich. Conc.
Fe	62.86	82.49	Fe	94.19
O	33.27	12.51		
Mn	3.88	5.00	Mn	5.81

FOV: 34.7 µm, Mode: 15kV - Map, Detector: BSD Full, Time: NOV 23 2017 19:43

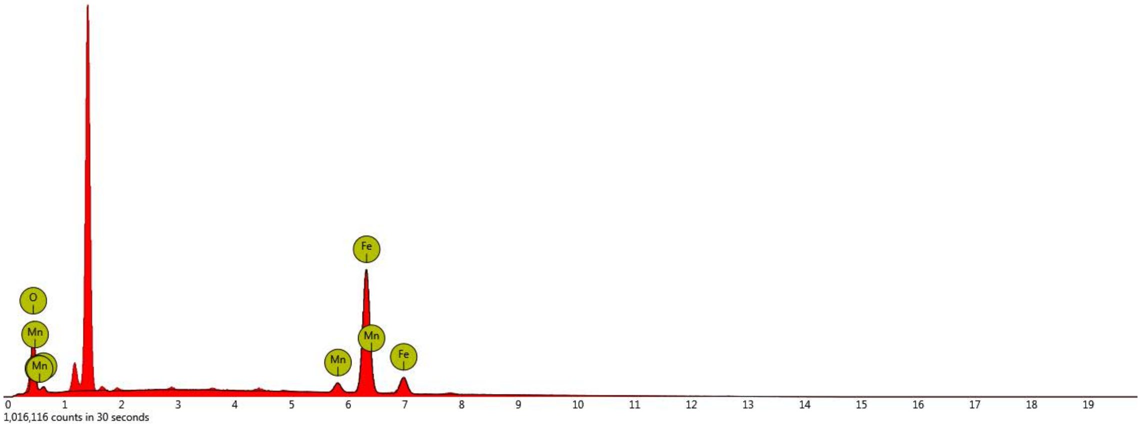

Disabled elements: Al, As, B, Br, Er, Mg, Se, Ti, Tm

Allaeochelys crassesculpta SMF ME 2449

Blood vessels embedded in bone 01

1. map

Combined map

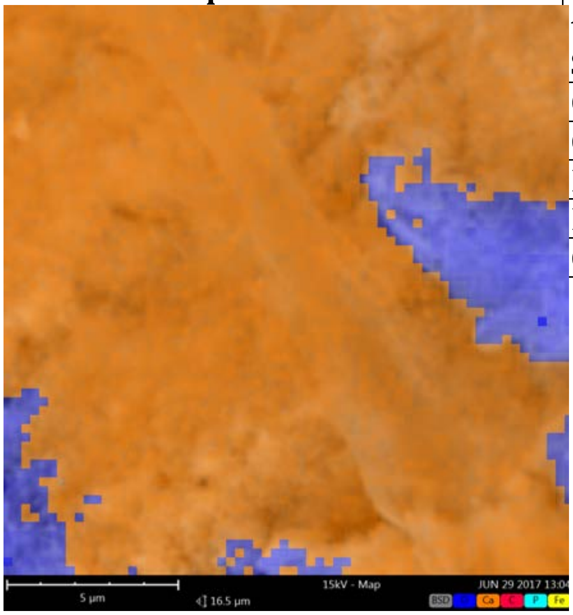

Element Symbol	Atomic Conc.	Weight Conc.	Oxide Symbol	Stoich. Conc.
O	61.82	48.05		
Ca	11.45	22.28	Ca	29.98
P	6.83	10.28	P	17.89
Fe	3.65	9.91	Fe	9.56
C	16.25	9.48	C	42.57

FOV: 16.5 µm, Mode: 15kV - Map, Detector: BSD Full, Time: JUN 29 2017 13:04

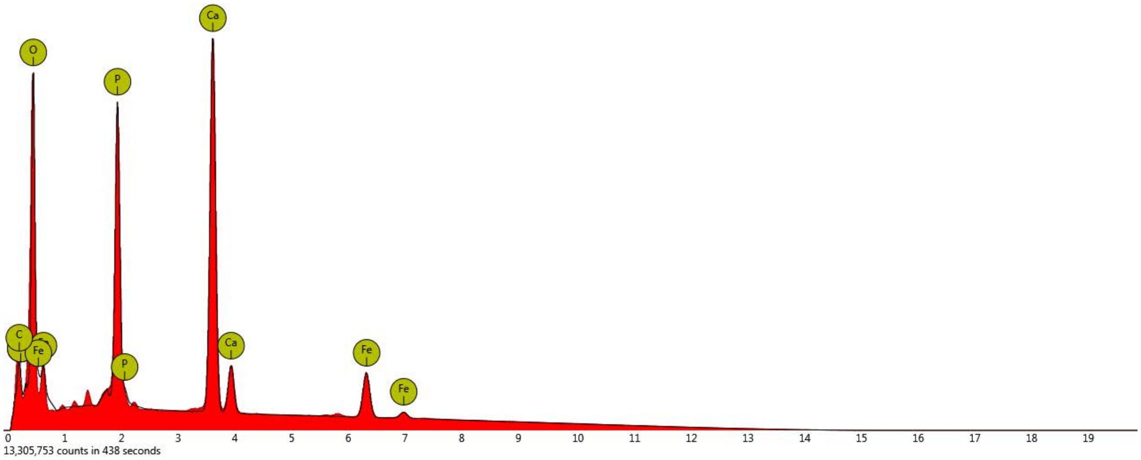

Disabled elements: B

Cut out of map (resolution: 64x64 pixels)

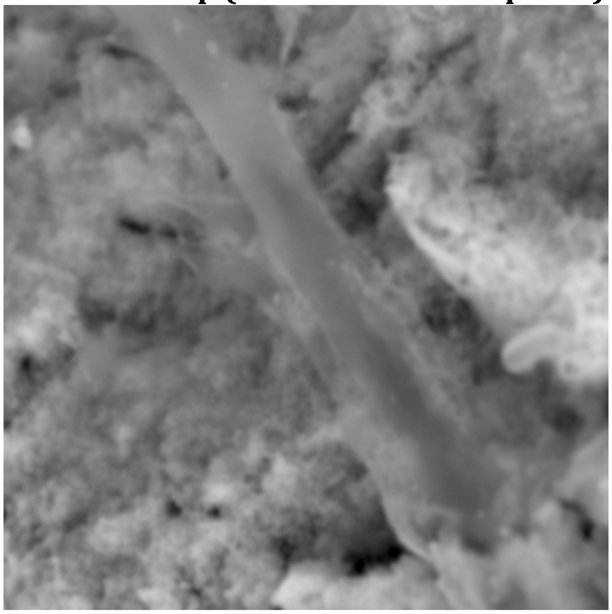

Oxygen

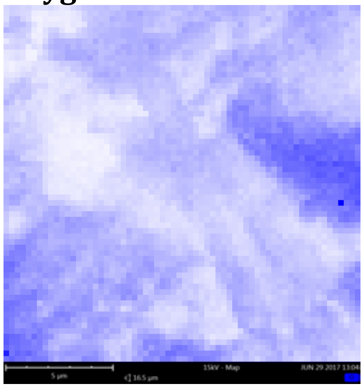

Calcium

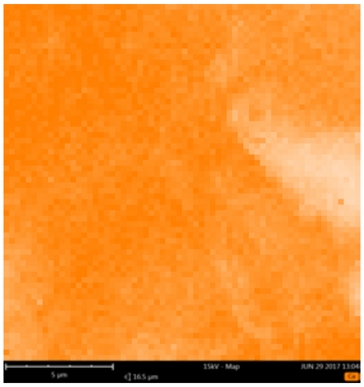

Phosphorus

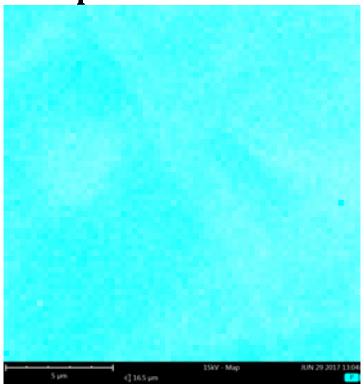

Iron

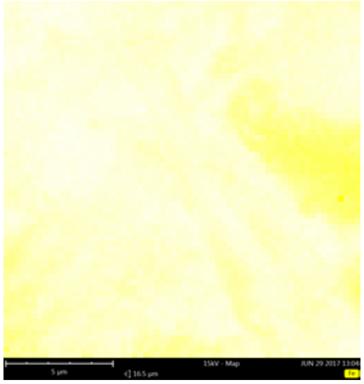

Carbon

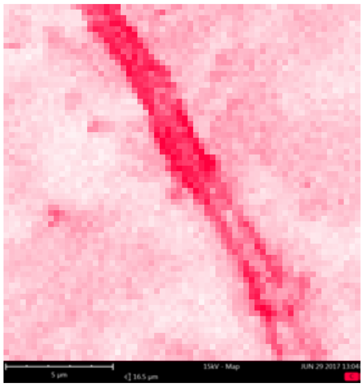

2. spot

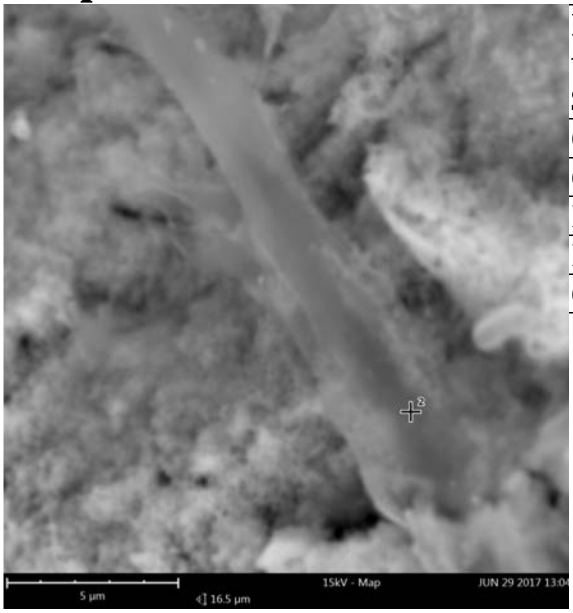

Element Symbol	Atomic Conc.	Weight Conc.	Oxide Symbol	Stoich. Conc.
O	56.27	41.86		
Ca	14.11	26.29	Ca	32.26
P	7.62	10.97	P	17.43
Fe	4.22	10.96	Fe	9.65
C	17.77	9.92	C	40.65

FOV: 16.5 µm, Mode: 15kV - Map, Detector: BSD Full, Time: JUN 29 2017 13:04

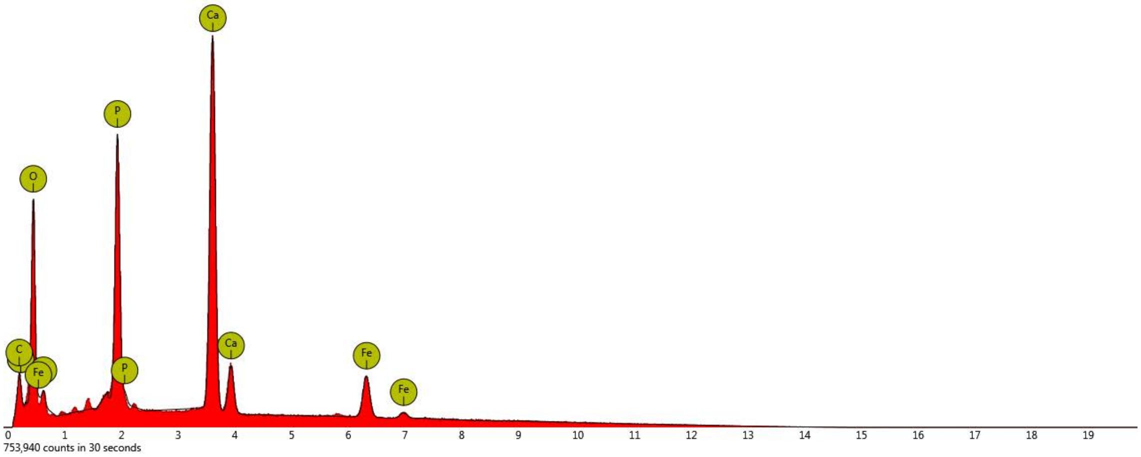

Disabled elements: B

3. spot

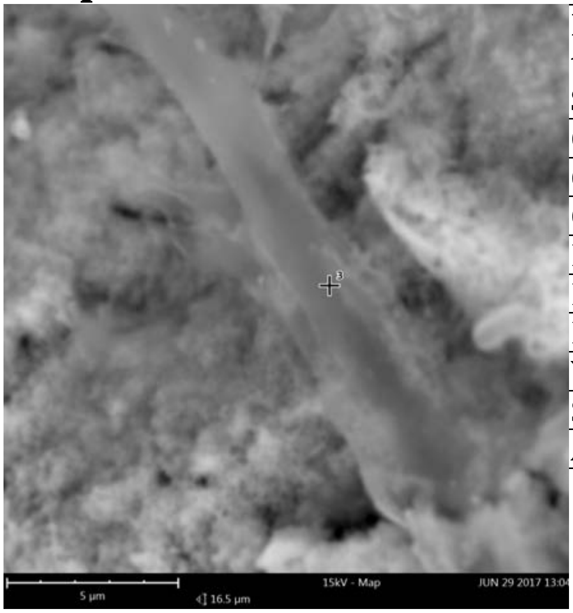

Element Symbol	Atomic Conc.	Weight Conc.	Oxide Symbol	Stoich. Conc.
O	50.69	43.04		
Ca	8.26	17.57	Ca	16.75
C	18.40	11.73	C	37.32
N	14.98	11.14	N	30.39
P	5.12	8.41	P	10.38
Fe	1.55	4.59	Fe	3.14
Y	0.36	1.72	Y	0.74
Sr	0.28	1.31	Sr	0.57
Al	0.35	0.50	Al	0.71

FOV: 16.5 µm, Mode: 15kV - Map, Detector: BSD Full, Time: JUN 29 2017 13:04

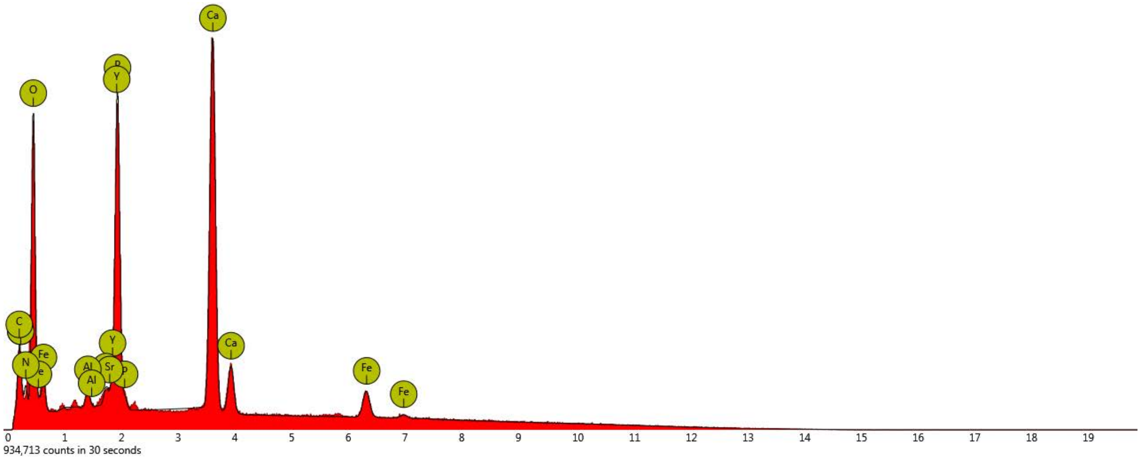

Disabled elements: B

4. spot

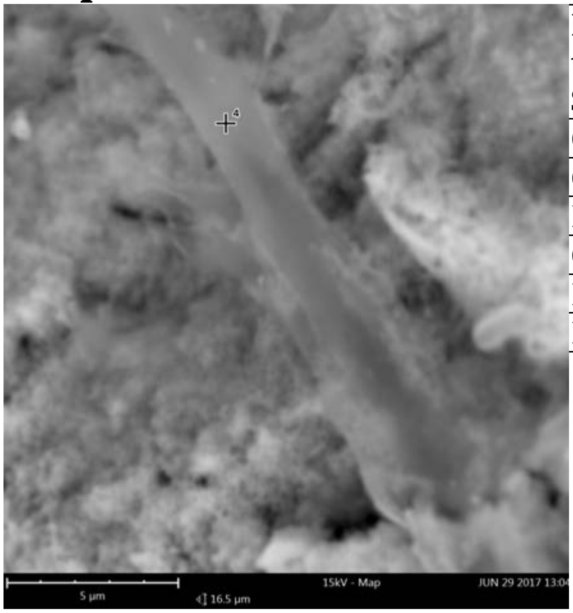

Element Symbol	Atomic Conc.	Weight Conc.	Oxide Symbol	Stoich. Conc.
O	52.82	47.02		
Ca	7.53	16.79	Ca	15.96
N	15.94	12.42	N	33.79
C	17.95	12.00	C	38.04
P	4.43	7.64	P	9.39
Fe	1.33	4.14	Fe	2.82

FOV: 16.5 μm, Mode: 15kV - Map, Detector: BSD Full, Time: JUN 29 2017 13:04

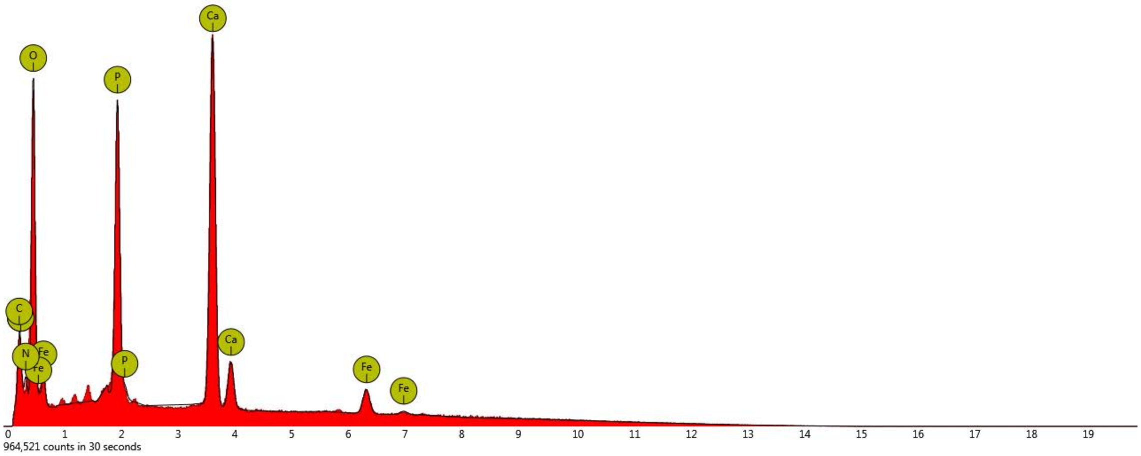

Disabled elements: B

5. spot

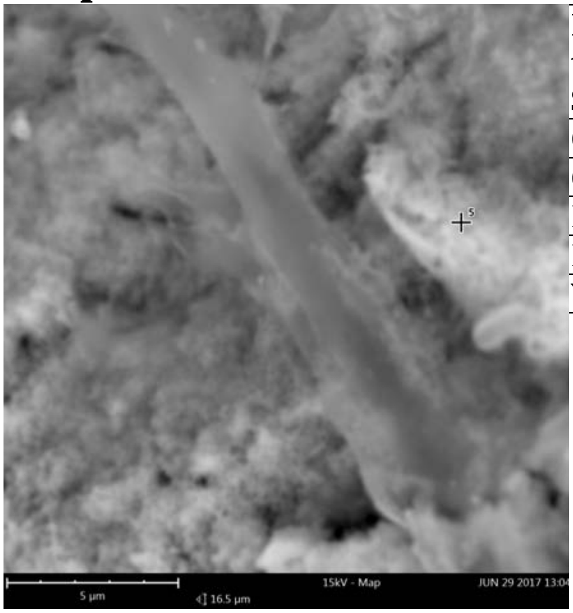

Element Symbol	Atomic Conc.	Weight Conc.	Oxide Symbol	Stoich. Conc.
O	74.31	52.30		
Ca	11.42	20.14	Ca	44.46
Fe	6.11	15.00	Fe	23.77
P	7.60	10.36	P	29.58
Y	0.56	2.20	Y	2.19

FOV: 16.5 µm, Mode: 15kV - Map, Detector: BSD Full, Time: JUN 29 2017 13:04

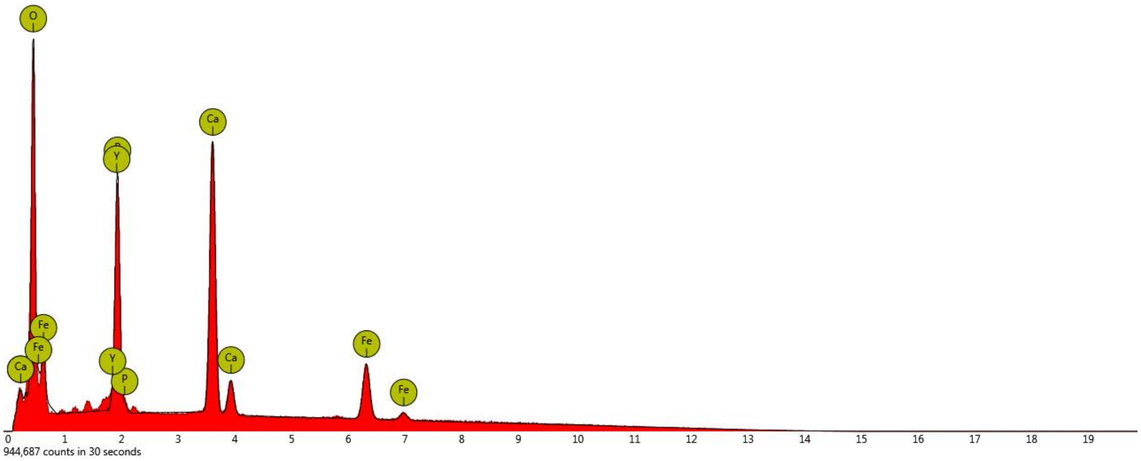

Disabled elements: B

6. spot

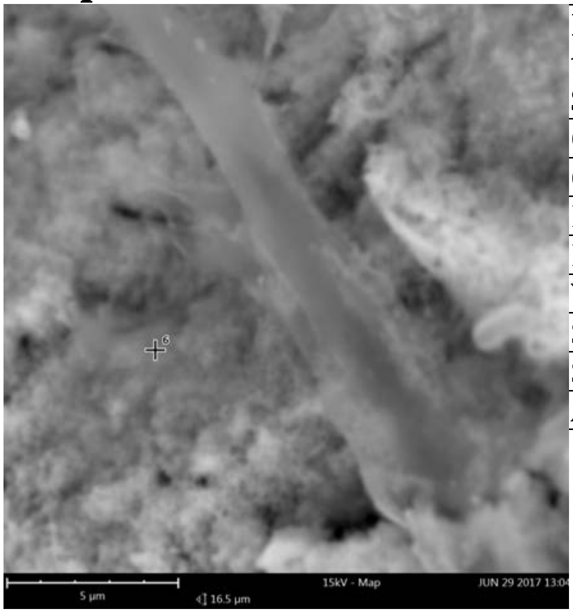

Element Symbol	Atomic Conc.	Weight Conc.	Oxide Symbol	Stoich. Conc.
O	73.83	52.39		
Ca	13.50	24.00	Ca	51.59
P	8.36	11.49	P	31.94
Fe	2.52	6.24	Fe	9.63
Y	0.58	2.30	Y	2.23
Sr	0.44	1.72	Sr	1.70
Sn	0.23	1.22	Sn	0.89
Al	0.53	0.64	Al	2.03

FOV: 16.5 µm, Mode: 15kV - Map, Detector: BSD Full, Time: JUN 29 2017 13:04

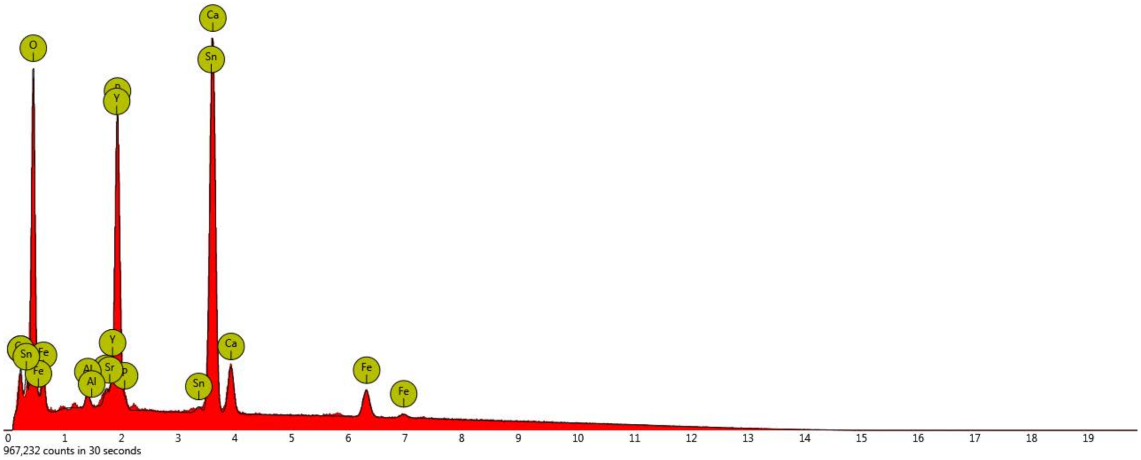

Disabled elements: B

Bone close to rock matrix

1. map

Combined map

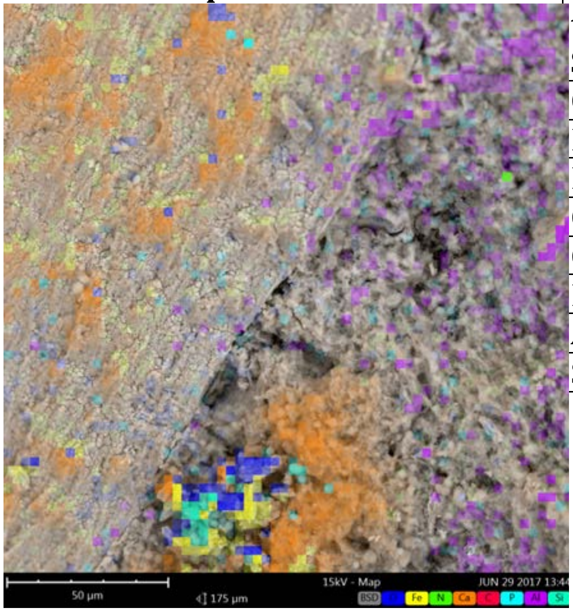

Element Symbol	Atomic Conc.	Weight Conc.	Oxide Symbol	Stoich. Conc.
O	42.80	34.47		
Fe	6.64	18.66	Fe	11.61
N	23.69	16.70	N	41.40
Ca	5.88	11.87	Ca	10.28
C	14.40	8.71	C	25.17
P	2.77	4.32	P	4.84
Al	2.52	3.42	Al	4.41
Si	1.31	1.85	Si	2.28

FOV: 175 µm, Mode: 15kV - Map, Detector: BSD Full, Time: JUN 29 2017 13:44

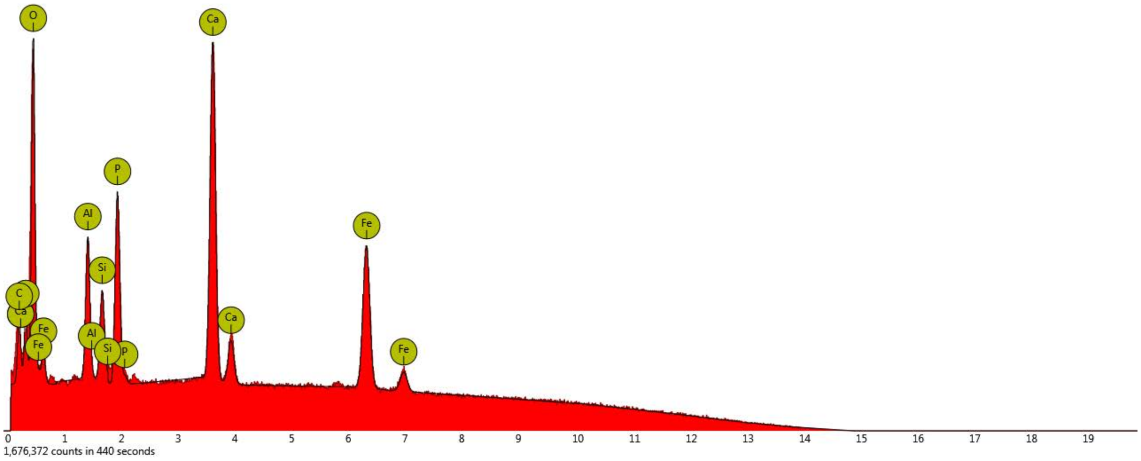

Disabled elements: B

Cut out of map (resolution: 64x64 pixels)

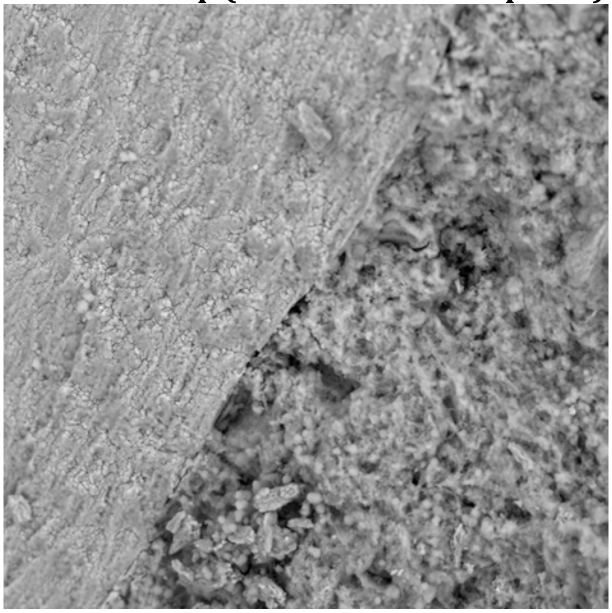

Oxygen

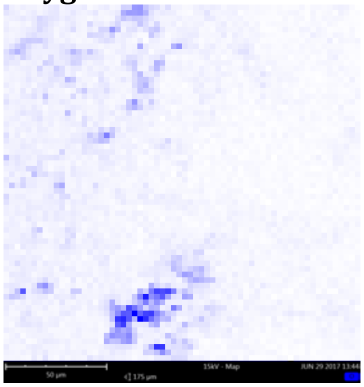

Iron

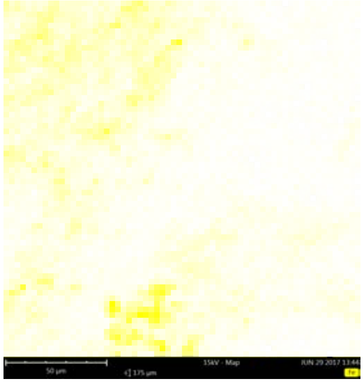

Nitrogen

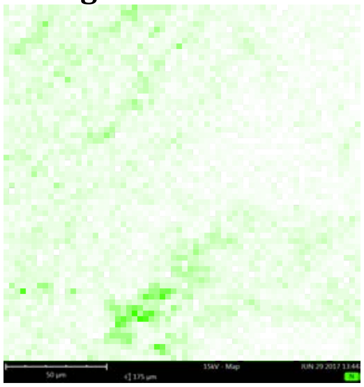

Calcium

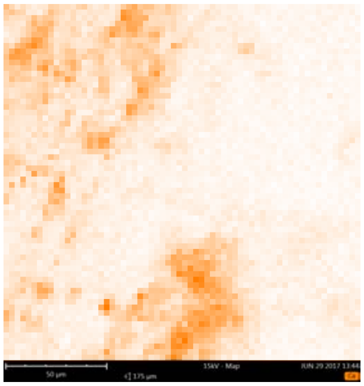

Carbon

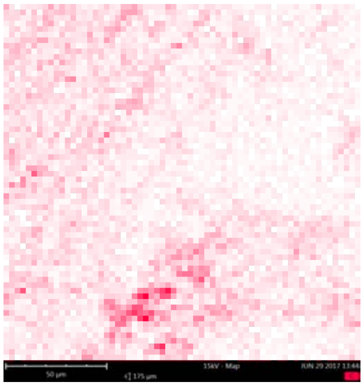

Phosphorus

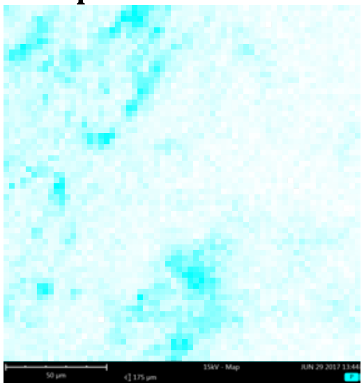

Aluminium

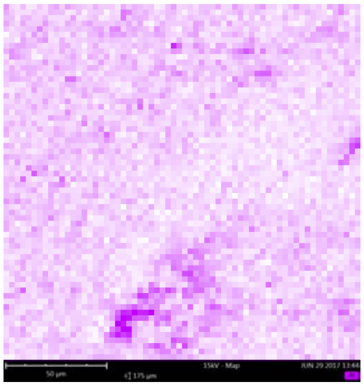

Silicon

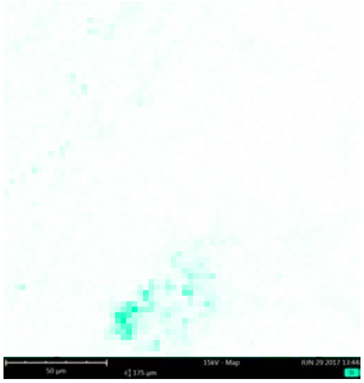

Blood vessel embedded in bone 2

Combined map

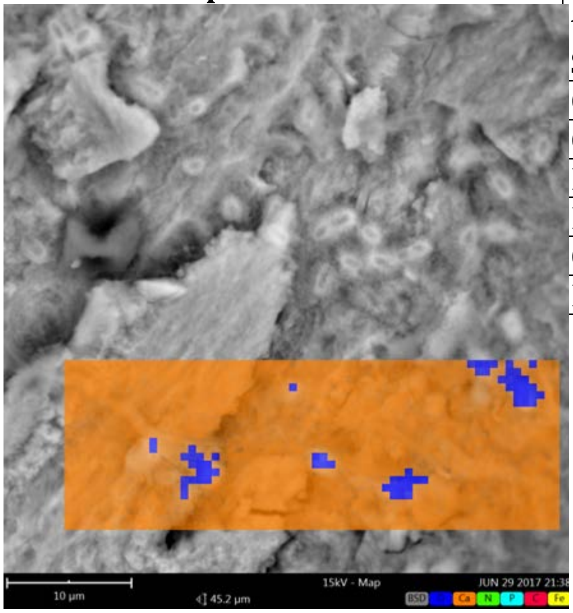

Element Symbol	Atomic Conc.	Weight Conc.	Oxide Symbol	Stoich. Conc.
O	52.70	45.51		
Ca	8.75	18.93	Ca	18.50
N	18.85	14.25	N	39.85
P	5.27	8.81	P	11.14
C	13.10	8.49	C	27.70
Fe	1.33	4.01	Fe	2.81

FOV: 45.2 µm, Mode: 15kV - Map, Detector: BSD Full, Time: JUN 29 2017 21:38

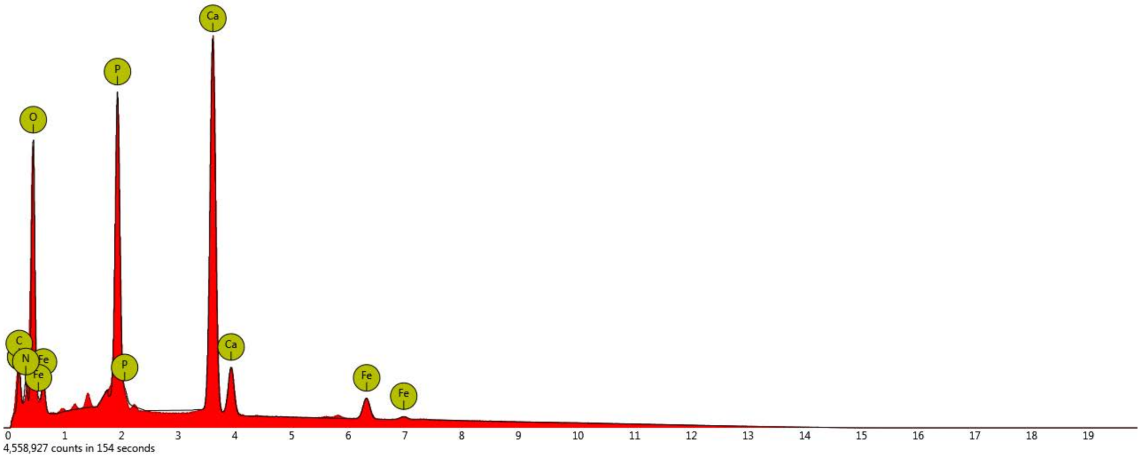

Disabled elements: B

Cut out of map (resolution: 64x22 pixels)

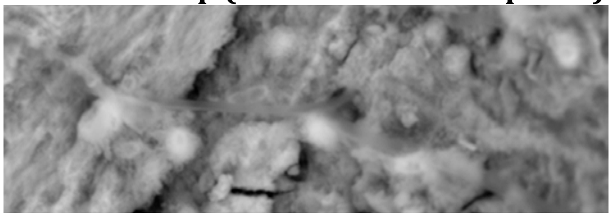

Oxygen

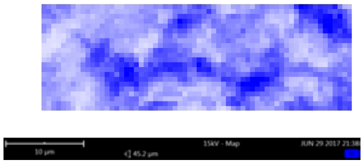

Calcium

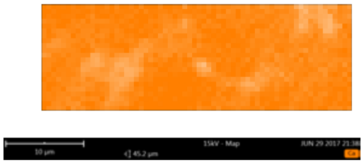

Nitrogen

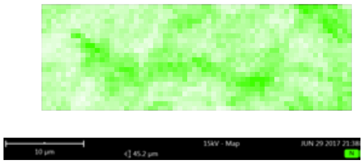

Phosphorus

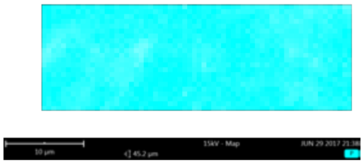

Carbon

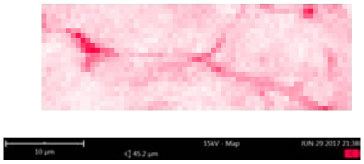

Iron

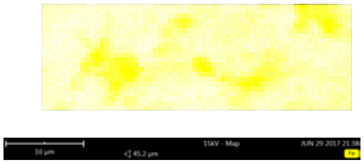

3. spot

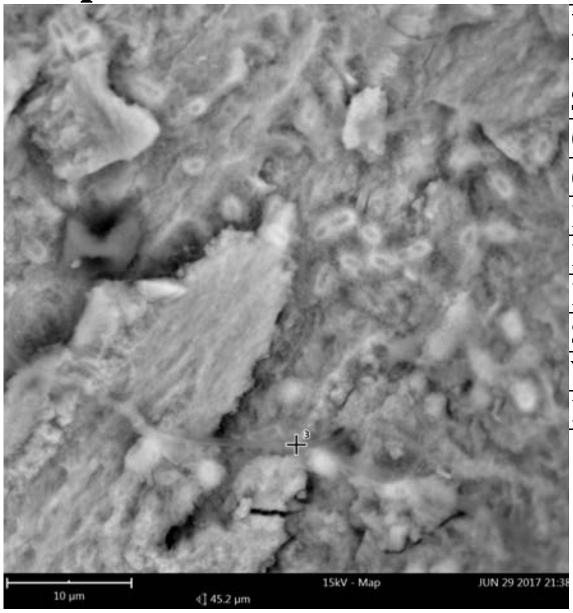

Element Symbol	Atomic Conc.	Weight Conc.	Oxide Symbol	Stoich. Conc.
O	58.54	43.42		
Ca	13.30	24.71	Ca	32.07
P	8.62	12.37	P	20.78
N	16.99	11.03	N	40.97
Fe	1.24	3.20	Fe	2.98
Sr	0.52	2.09	Sr	1.24
Y	0.40	1.65	Y	0.97
Br	0.41	1.52	Br	0.99

FOV: 45.2 µm, Mode: 15kV - Map, Detector: BSD Full, Time: JUN 29 2017 21:38

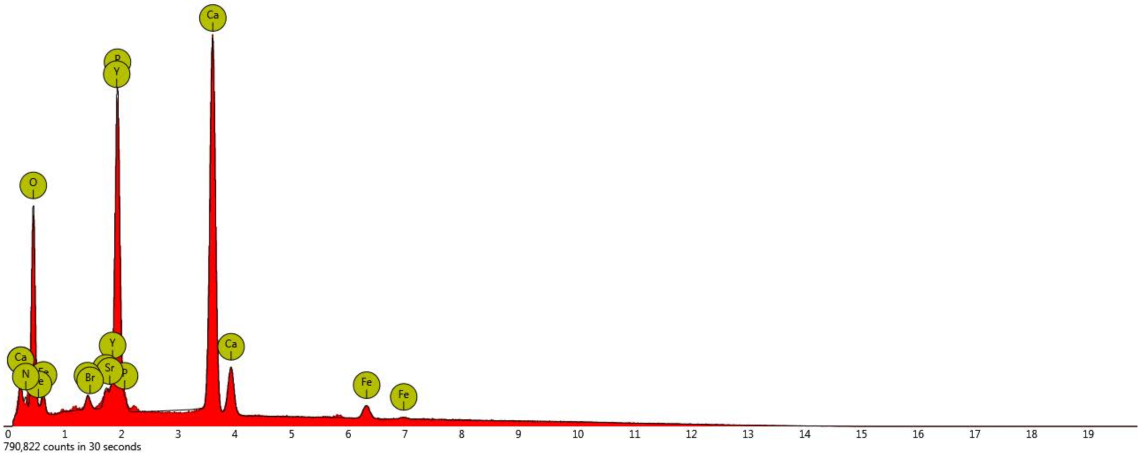

Disabled elements: B

4. spot

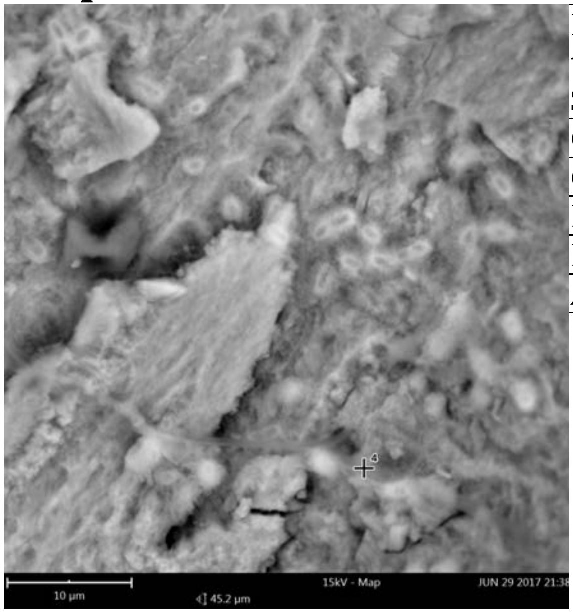

Element Symbol	Atomic Conc.	Weight Conc.	Oxide Symbol	Stoich. Conc.
O	72.57	53.11		
Ca	15.54	28.50	Ca	56.66
P	9.97	14.13	P	36.36
Fe	1.43	3.66	Fe	5.23
Al	0.48	0.59	Al	1.75

FOV: 45.2 µm, Mode: 15kV - Map, Detector: BSD Full, Time: JUN 29 2017 21:38

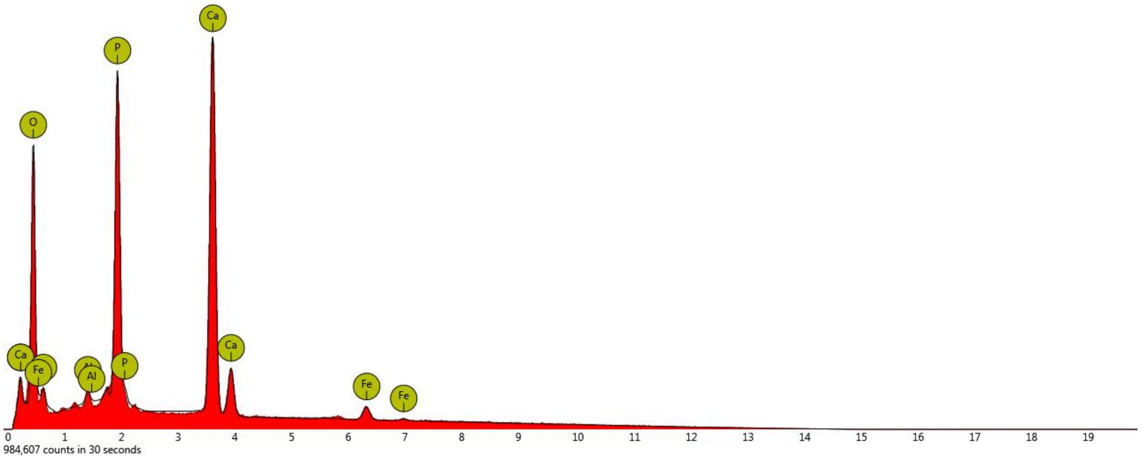

Disabled elements: B

5. spot

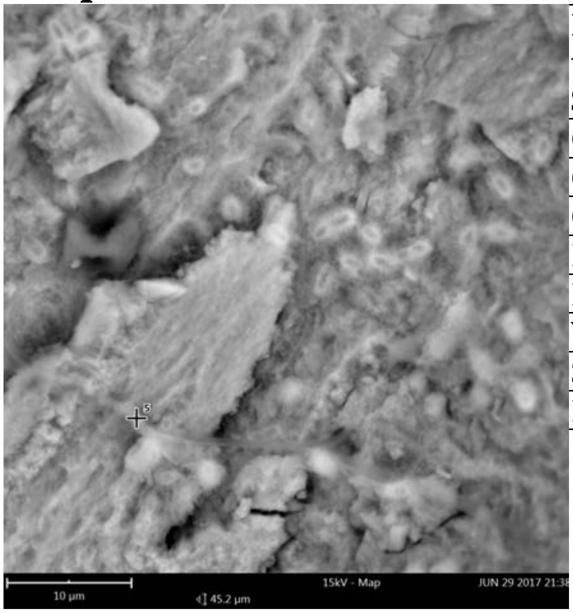

Element Symbol	Atomic Conc.	Weight Conc.	Oxide Symbol	Stoich. Conc.
O	54.84	44.54		
Ca	9.67	19.68	Ca	21.42
C	26.27	16.02	C	58.17
P	6.23	9.79	P	13.79
Fe	2.02	5.73	Fe	4.48
Y	0.36	1.64	Y	0.81
Sr	0.34	1.52	Sr	0.76
Br	0.26	1.07	Br	0.58

FOV: 45.2 µm, Mode: 15kV - Map, Detector: BSD Full, Time: JUN 29 2017 21:38

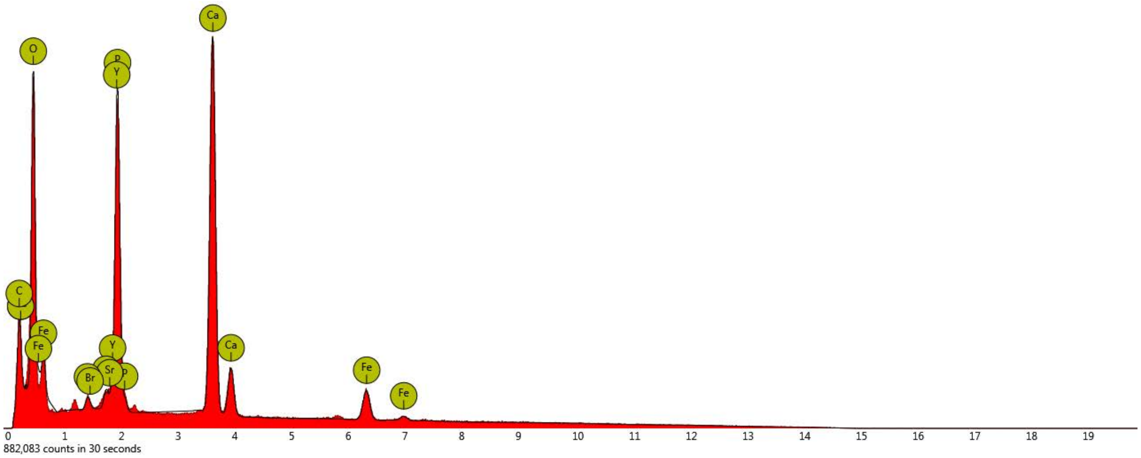

Disabled elements: B

Blood vessel embedded in bone 3

Combined map

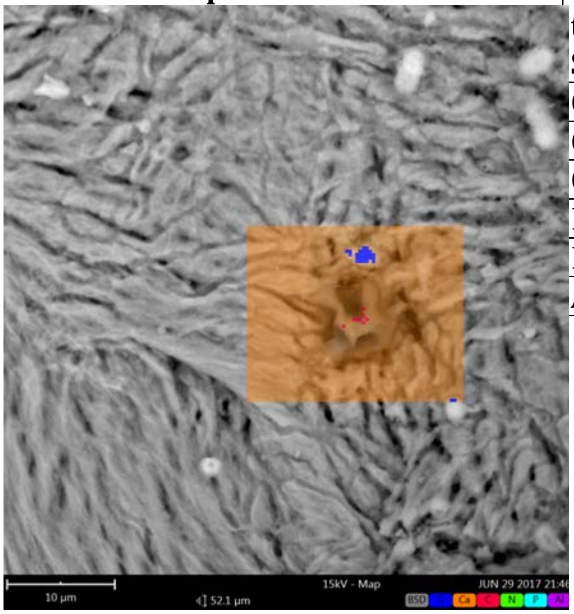

Element Symbol	Atomic Conc.	Weight Conc.	Oxide Symbol	Stoich. Conc.
O	49.23	44.04		
Ca	8.92	19.98	Ca	17.56
C	20.49	13.76	C	40.36
N	15.49	12.13	N	30.50
P	5.47	9.47	P	10.76
Al	0.41	0.62	Al	0.81

FOV: 52.1 µm, Mode: 15kV - Map, Detector: BSD Full, Time: JUN 29 2017 21:46

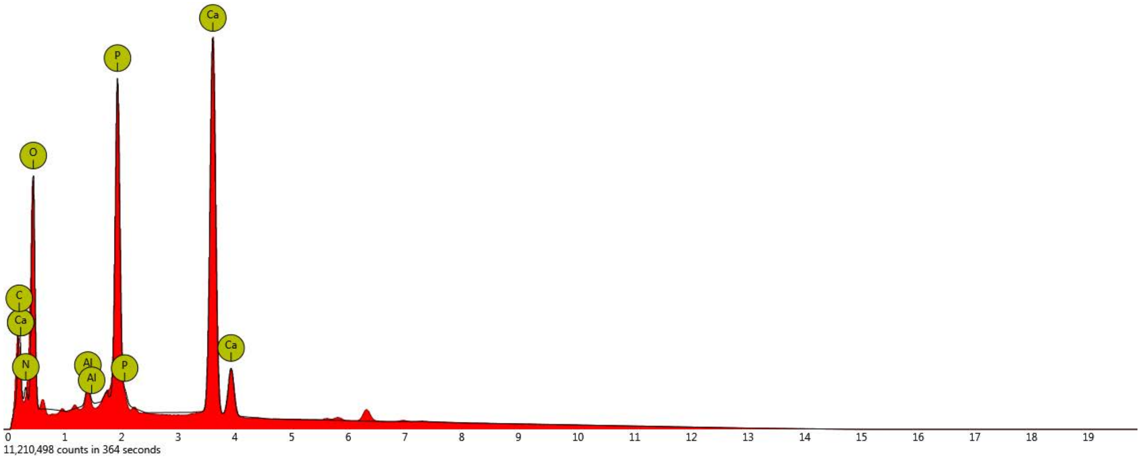

Disabled elements: B

Cut out of map (resolution: 64x52 pixels)

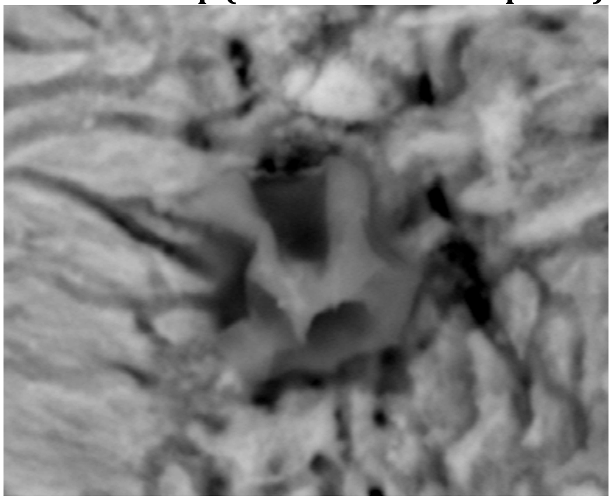

Oxygen

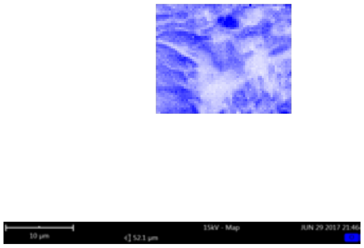

Calcium

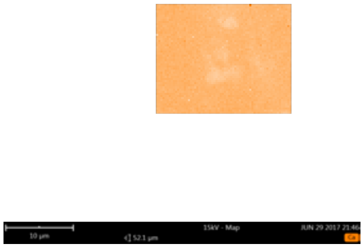

Carbon

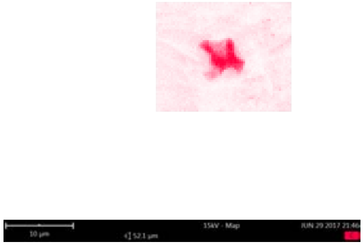

Nitrogen

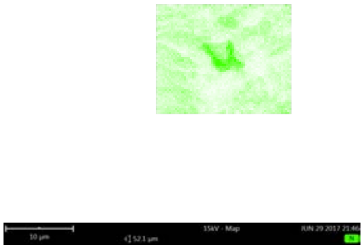

Phosphorus

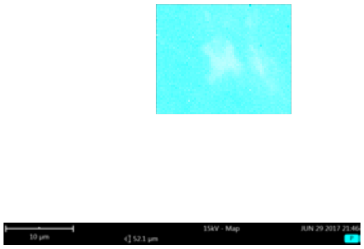

Aluminium

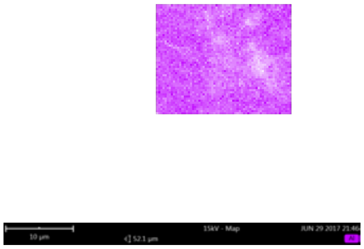

2. spot

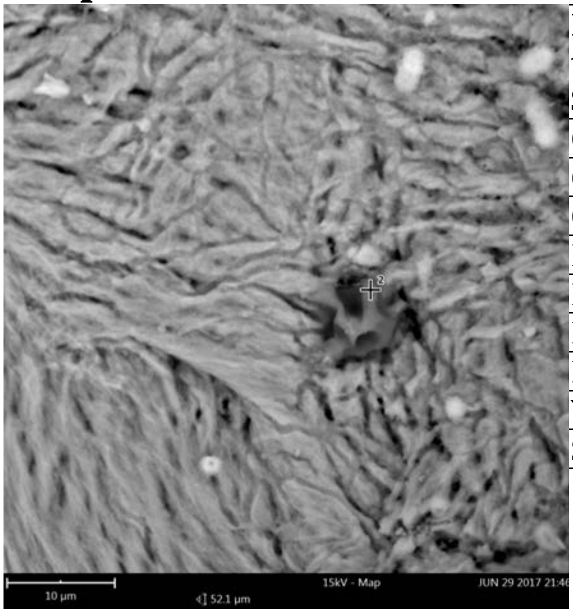

Element Symbol	Atomic Conc.	Weight Conc.	Oxide Symbol	Stoich. Conc.
O	38.44	31.90		
Ca	10.25	21.31	Ca	16.65
C	27.13	16.90	C	44.07
N	15.46	11.23	N	25.12
P	6.58	10.58	P	10.70
Fe	0.88	2.54	Fe	1.43
Br	0.49	2.05	Br	0.80
Y	0.39	1.78	Y	0.63
Sr	0.38	1.71	Sr	0.61

FOV: 52.1 µm, Mode: 15kV - Map, Detector: BSD Full, Time: JUN 29 2017 21:46

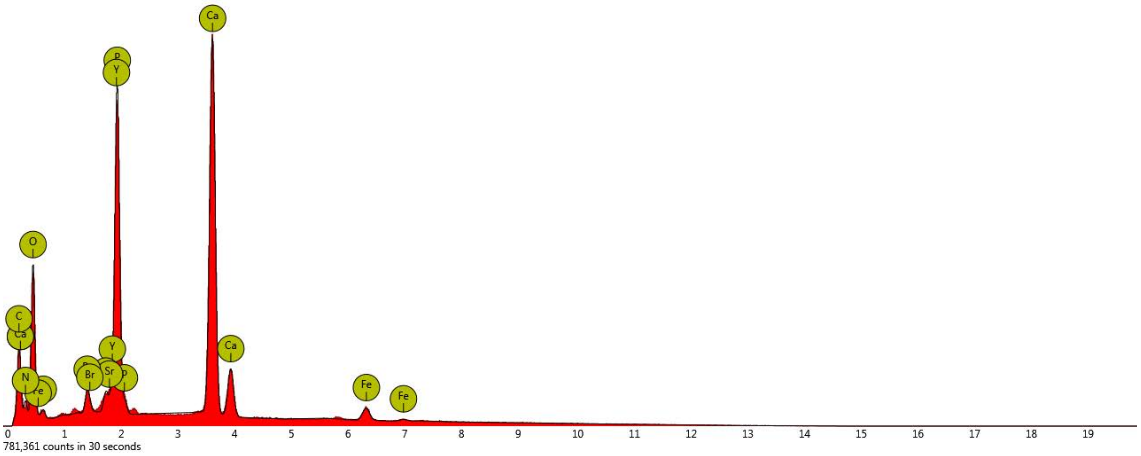

Disabled elements: B

3. spot

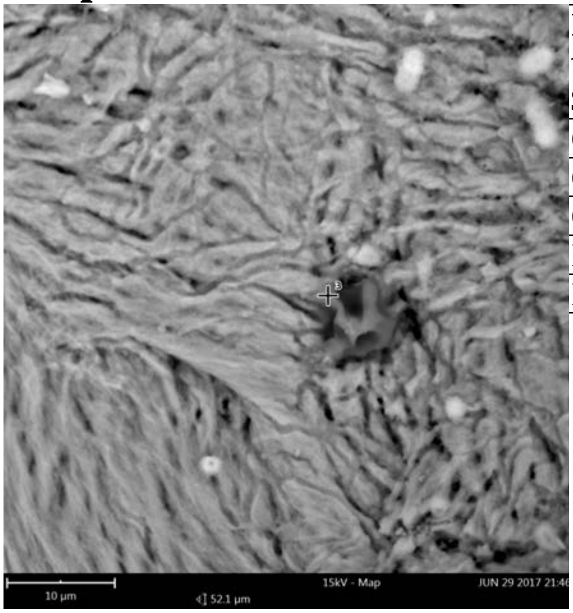

Element Symbol	Atomic Conc.	Weight Conc.	Oxide Symbol	Stoich. Conc.
C	55.20	43.94	C	90.36
O	38.90	41.25		
Ca	3.24	8.60	Ca	5.30
P	2.29	4.69	P	3.74
Fe	0.32	1.19	Fe	0.53

FOV: 52.1 µm, Mode: 15kV - Map, Detector: BSD Full, Time: JUN 29 2017 21:46

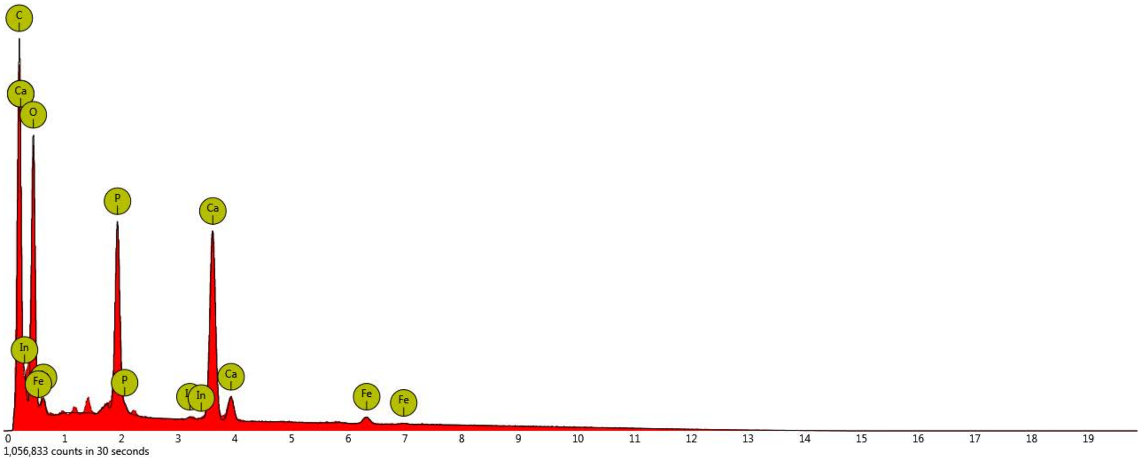

Disabled elements: B

4. spot

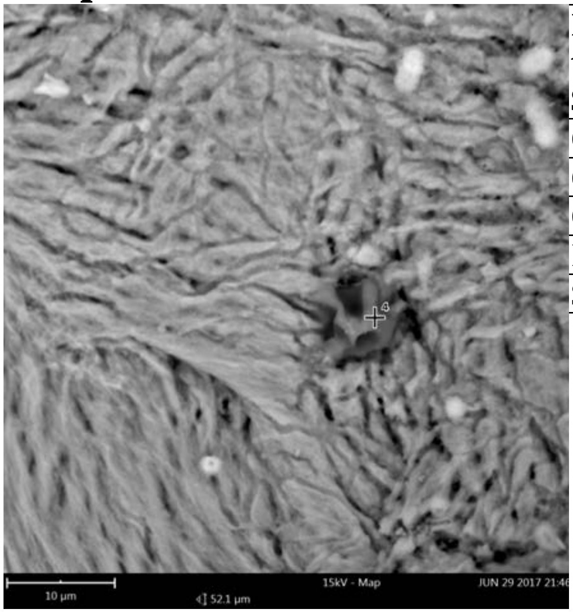

Element Symbol	Atomic Conc.	Weight Conc.	Oxide Symbol	Stoich. Conc.
O	39.93	40.45		
C	51.36	39.06	C	85.50
Ca	5.27	13.36	Ca	8.77
P	3.38	6.62	P	5.62
Sn	0.07	0.51	Sn	0.11

FOV: 52.1 µm, Mode: 15kV - Map, Detector: BSD Full, Time: JUN 29 2017 21:46

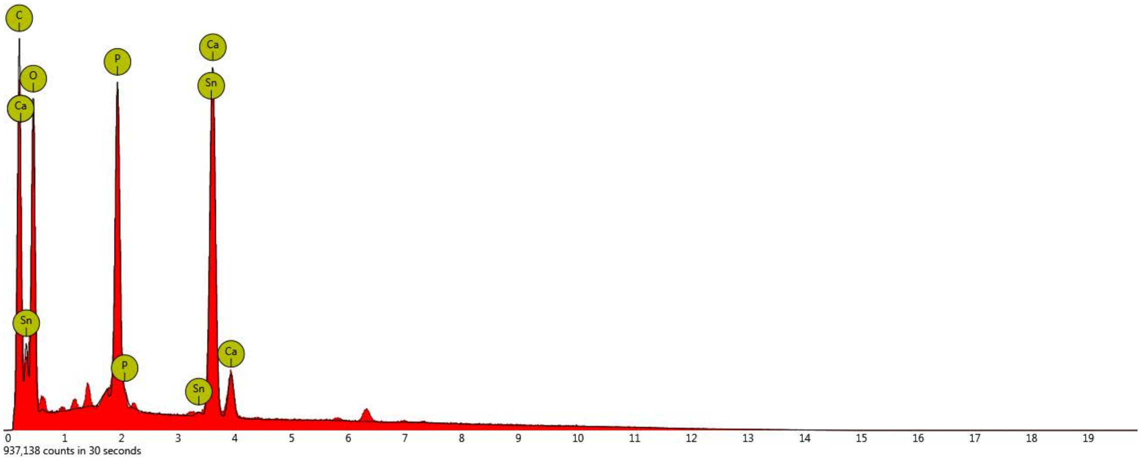

Disabled elements: B

5. spot

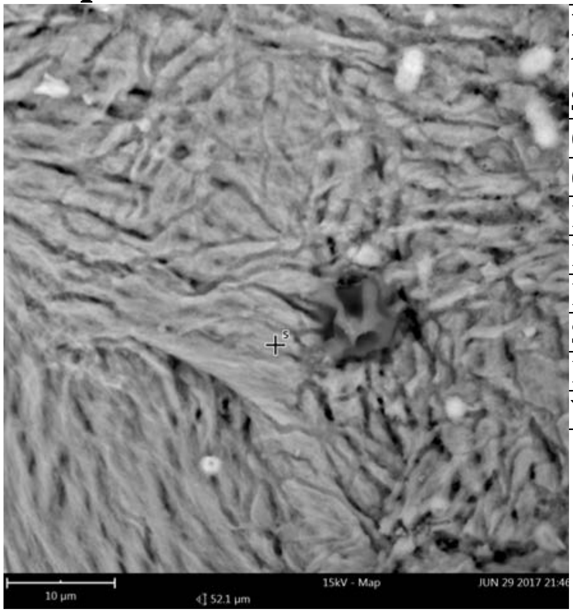

Element Symbol	Atomic Conc.	Weight Conc.	Oxide Symbol	Stoich. Conc.
O	58.07	41.69		
Ca	14.56	26.20	Ca	34.73
P	10.27	14.27	P	24.48
N	14.54	9.14	N	34.68
Br	0.76	2.72	Br	1.81
Sr	0.59	2.33	Sr	1.41
Fe	0.80	2.00	Fe	1.90
Y	0.41	1.65	Y	0.99

FOV: 52.1 µm, Mode: 15kV - Map, Detector: BSD Full, Time: JUN 29 2017 21:46

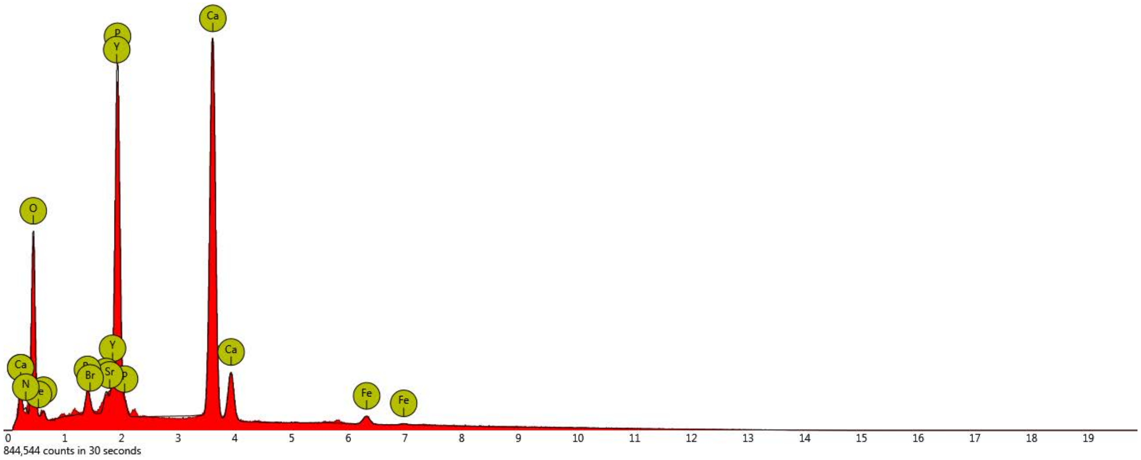

Disabled elements: B

Podocnemidid Indt. UR-CP-0043

Blood vessels embedded in bone 01

1. map

Combined map

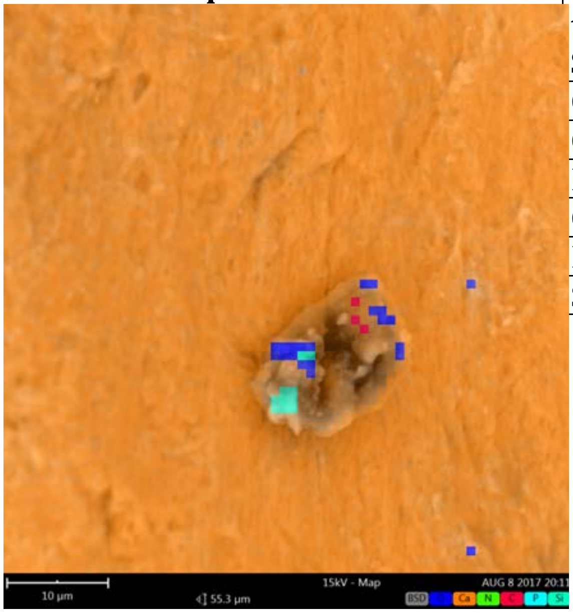

Element Symbol	Atomic Conc.	Weight Conc.	Oxide Symbol	Stoich. Conc.
O	54.11	47.60		
Ca	9.28	20.45	Ca	20.22
N	16.33	12.58	N	35.59
C	14.43	9.53	C	31.44
P	5.09	8.67	P	11.09
Si	0.76	1.17	Si	1.66

FOV: 55.3 µm, Mode: 15kV - Map, Detector: BSD Full, Time: AUG 8 2017 20:11

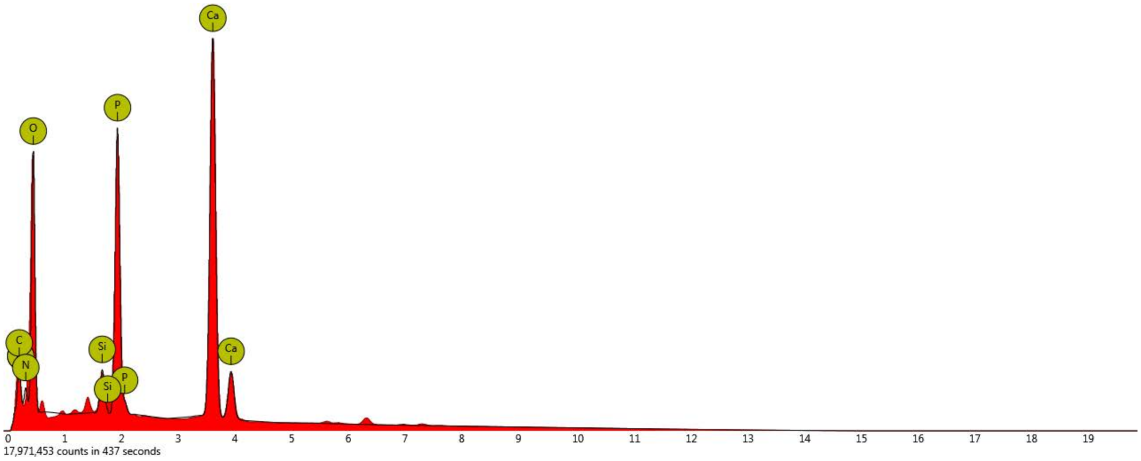

Disabled elements: B, Sr, Y

Cut out of map (resolution: 64x64 pixels)

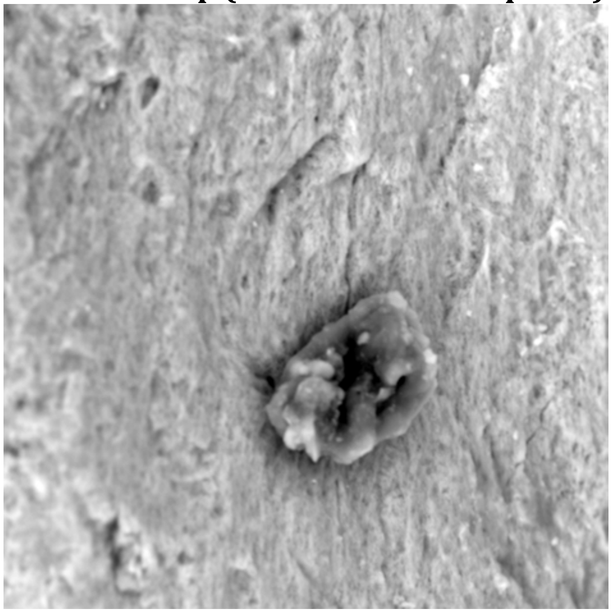

Oxygen

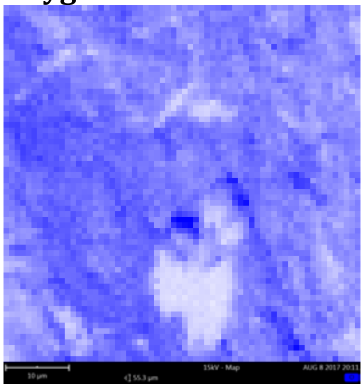

Calcium

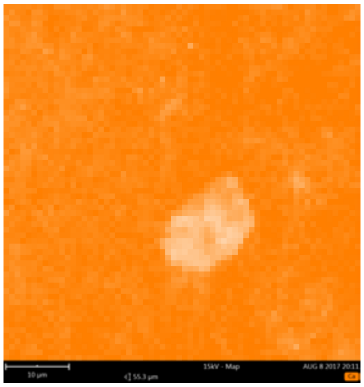

Nitrogen

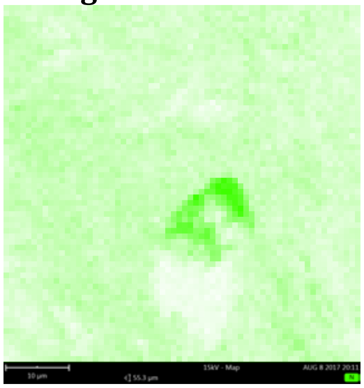

Carbon

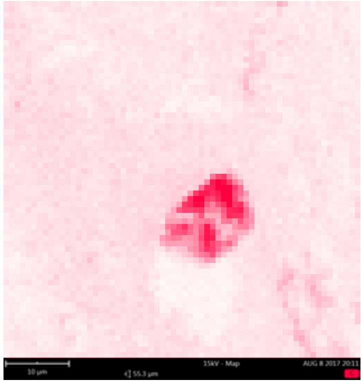

Phosphorus

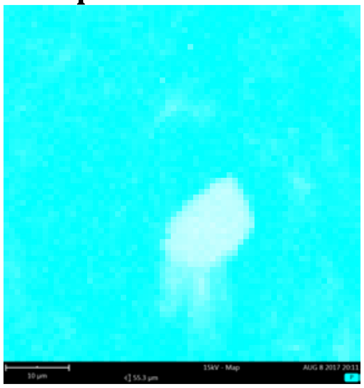

Silicon

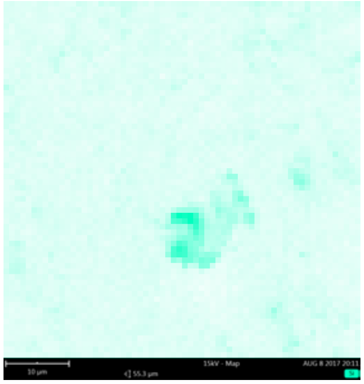

2. spot

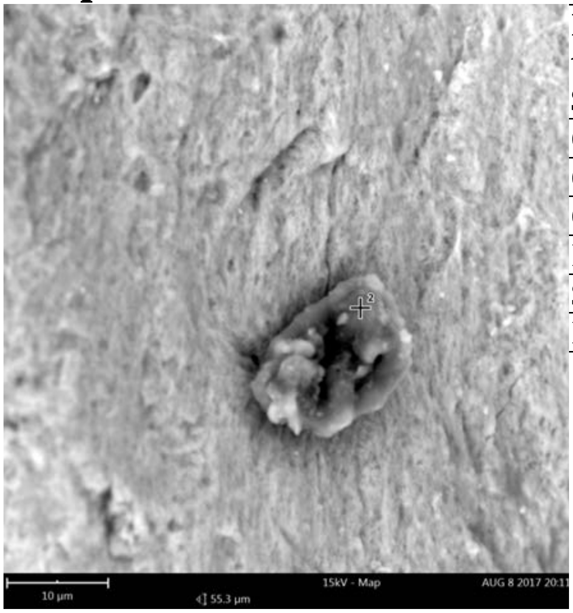

Element Symbol	Atomic Conc.	Weight Conc.	Oxide Symbol	Stoich. Conc.
C	65.85	55.52	C	94.49
O	30.31	34.04		
Ca	1.52	4.28	Ca	2.19
Br	0.34	1.92	Br	0.49
Si	0.90	1.78	Si	1.30
P	0.78	1.69	P	1.11

FOV: 55.3 µm, Mode: 15kV - Map, Detector: BSD Full, Time: AUG 8 2017 20:11

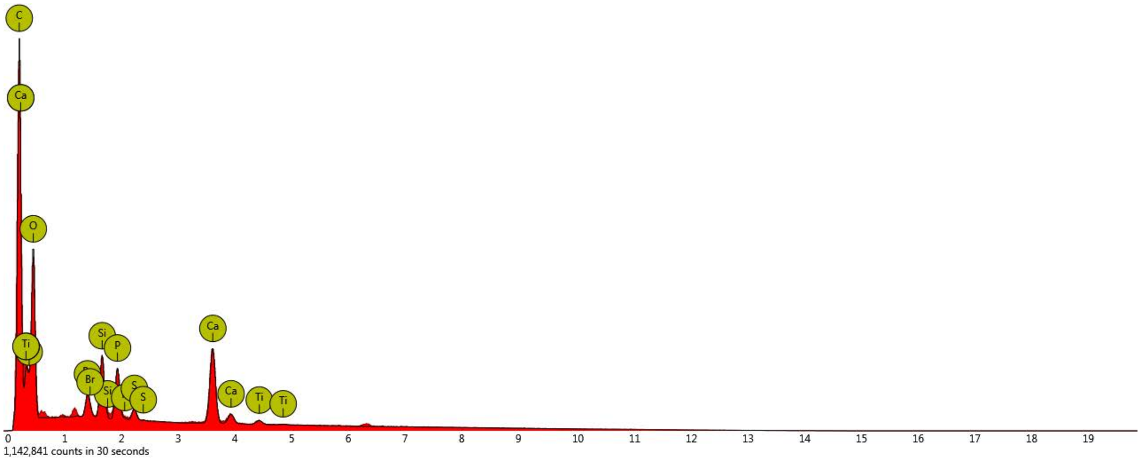

Disabled elements: B

3. spot

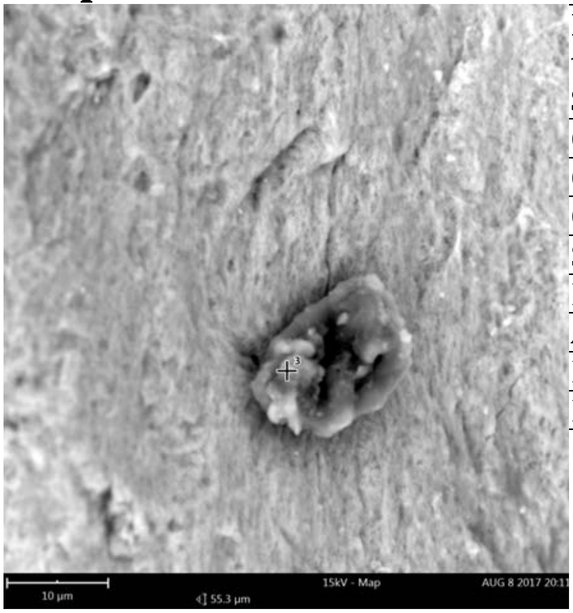

Element Symbol	Atomic Conc.	Weight Conc.	Oxide Symbol	Stoich. Conc.
C	55.89	45.36	C	90.79
O	38.44	41.56		
Ca	1.49	4.03	Ca	2.42
Si	1.99	3.77	Si	3.23
P	0.83	1.74	P	1.35
Al	0.83	1.50	Al	1.34
Fe	0.20	0.74	Fe	0.32
In	0.08	0.61	In	0.13

FOV: 55.3 µm, Mode: 15kV - Map, Detector: BSD Full, Time: AUG 8 2017 20:11

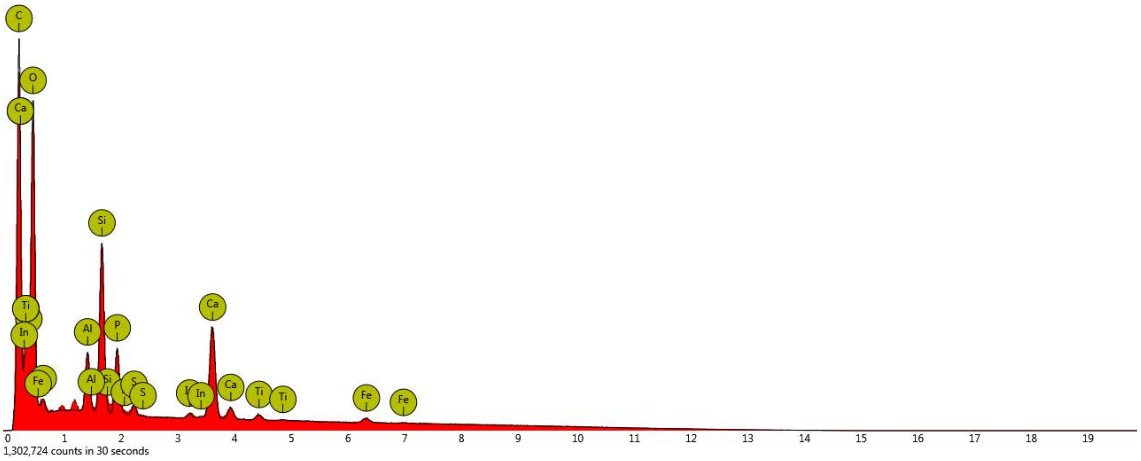

Disabled elements: B

4. spot

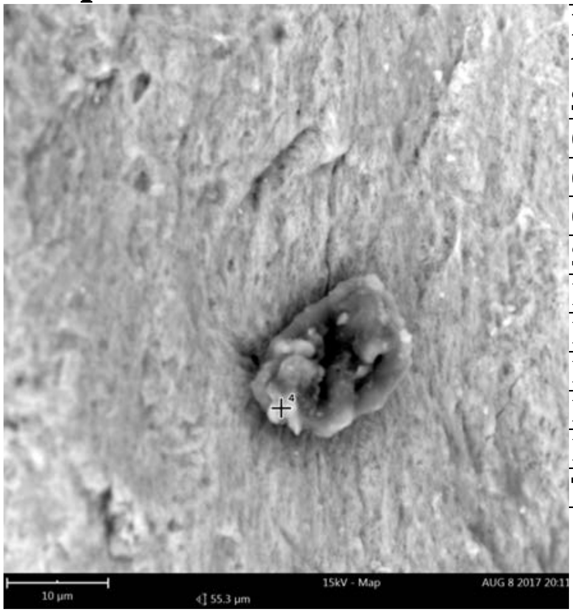

Element Symbol	Atomic Conc.	Weight Conc.	Oxide Symbol	Stoich. Conc.
O	44.63	36.71		
C	34.34	21.21	C	62.02
Ca	5.90	12.17	Ca	10.66
Si	7.25	10.46	Si	13.09
Br	2.17	8.91	Br	3.92
P	3.19	5.08	P	5.76
Fe	1.04	2.98	Fe	1.87
Mg	0.81	1.01	Mg	1.46
K	0.40	0.79	K	0.71
Ti	0.28	0.68	Ti	0.50

FOV: 55.3 µm, Mode: 15kV - Map, Detector: BSD Full, Time: AUG 8 2017 20:11

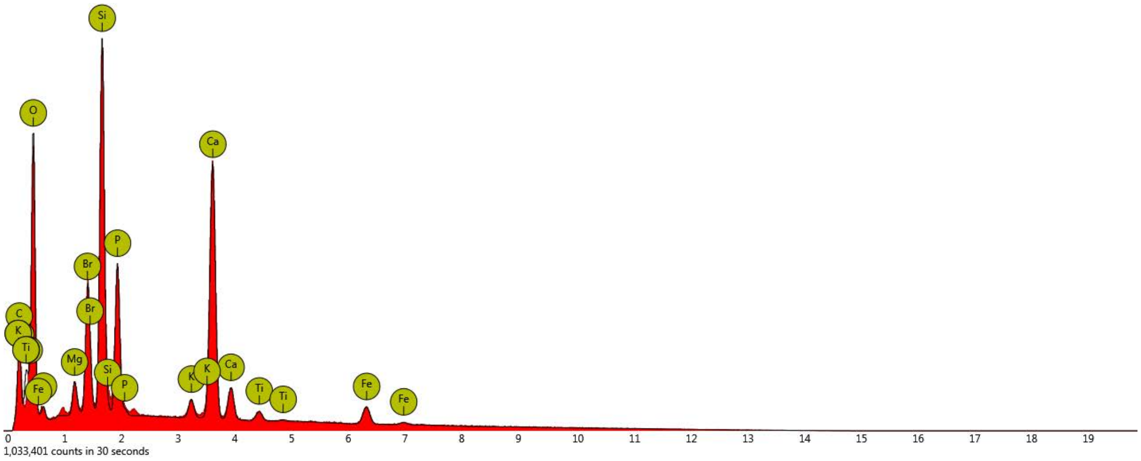

Disabled elements: B

5. spot

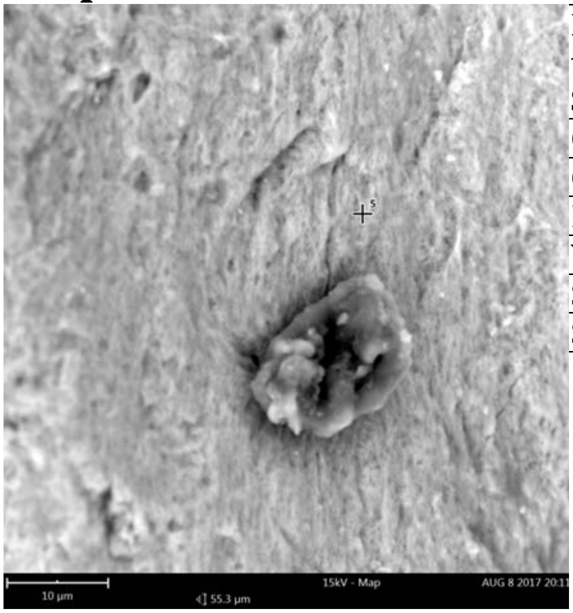

**Element Symbol**	**Atomic Conc.**	**Weight Conc.**	**Oxide Symbol**	**Stoich. Conc.**
O	69.85	49.00		
Ca	18.05	31.72	Ca	59.87
P	10.25	13.92	P	34.00
Y	0.69	2.71	Y	2.30
Sr	0.47	1.80	Sr	1.55
Si	0.69	0.84	Si	2.27

FOV: 55.3 µm, Mode: 15kV - Map, Detector: BSD Full, Time: AUG 8 2017 20:11

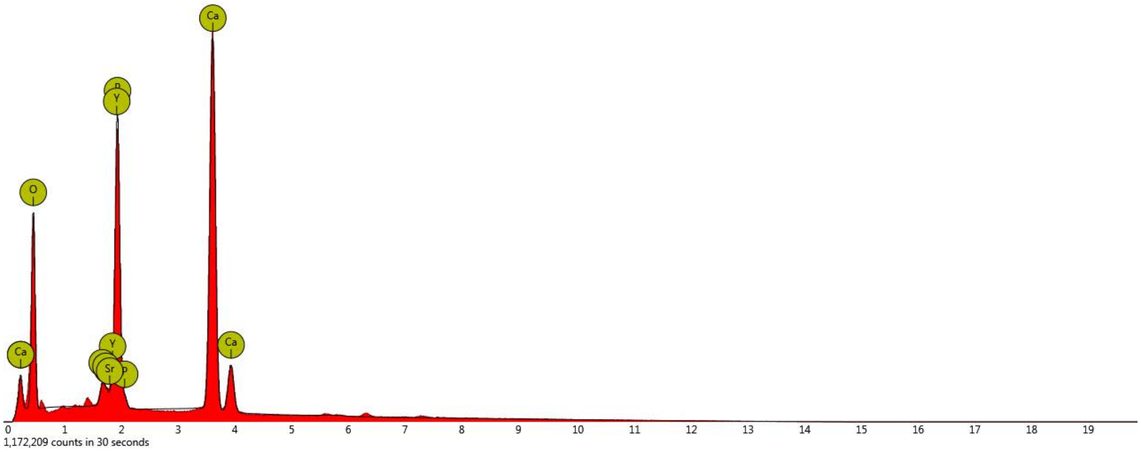

Disabled elements: B

6. spot

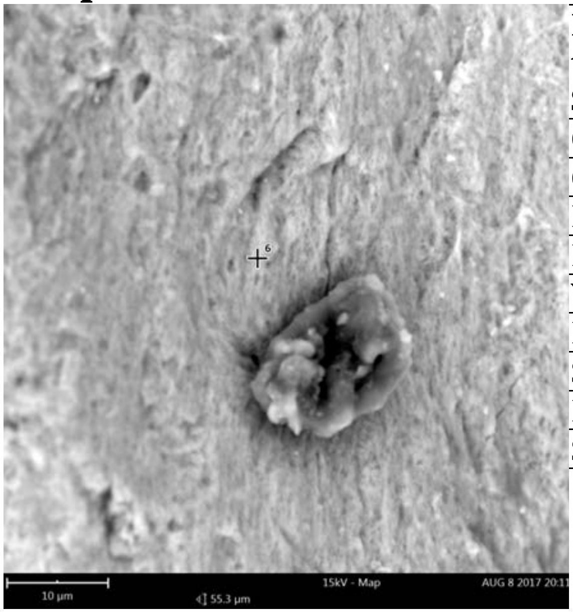

Element Symbol	Atomic Conc.	Weight Conc.	Oxide Symbol	Stoich. Conc.
O	58.99	44.36		
Ca	13.36	25.16	Ca	32.57
N	17.21	11.33	N	41.95
P	7.78	11.32	P	18.96
Y	0.54	2.24	Y	1.31
Fe	0.70	1.84	Fe	1.71
Sr	0.36	1.49	Sr	0.88
Br	0.35	1.30	Br	0.84
Si	0.73	0.96	Si	1.78

FOV: 55.3 µm, Mode: 15kV - Map, Detector: BSD Full, Time: AUG 8 2017 20:11

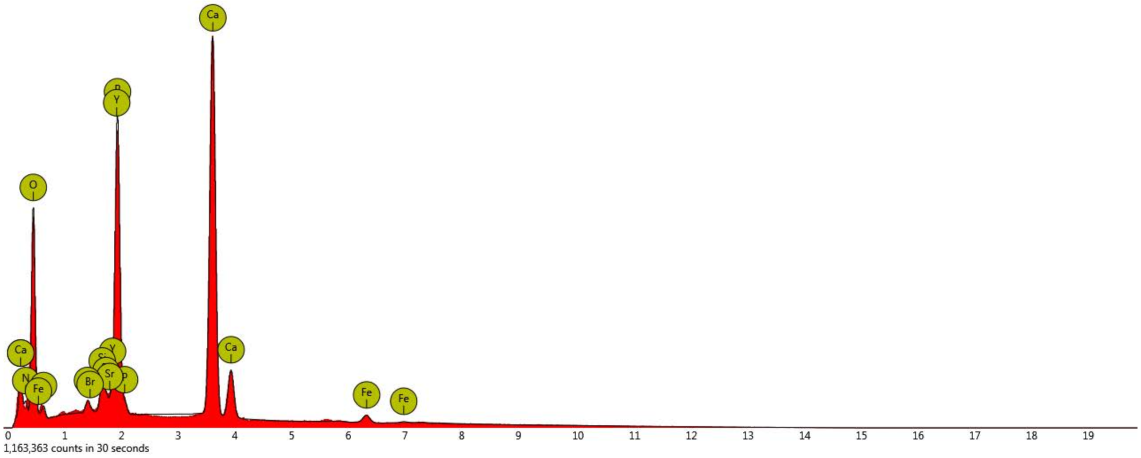

Disabled elements: B

7. spot

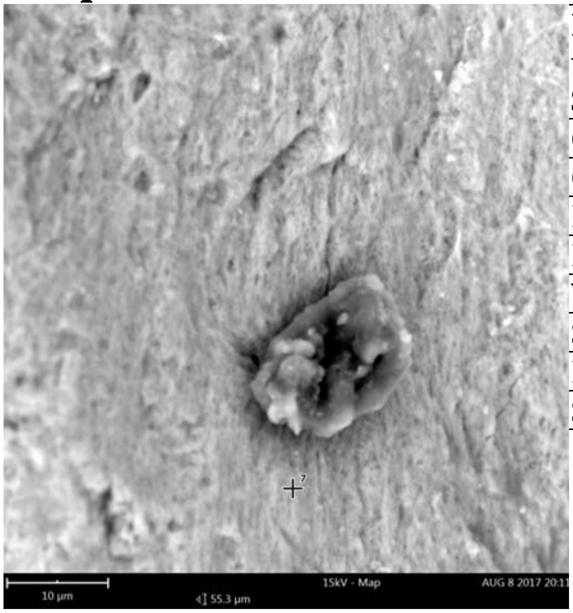

Element Symbol	Atomic Conc.	Weight Conc.	Oxide Symbol	Stoich. Conc.
Ca	28.14	41.39	Ca	60.86
O	53.76	31.57		
P	12.30	13.98	P	26.61
Fe	1.84	3.76	Fe	3.97
Y	1.06	3.47	Y	2.30
Sr	0.68	2.17	Sr	1.46
Br	0.71	2.09	Br	1.54
Si	1.51	1.55	Si	3.26

FOV: 55.3 µm, Mode: 15kV - Map, Detector: BSD Full, Time: AUG 8 2017 20:11

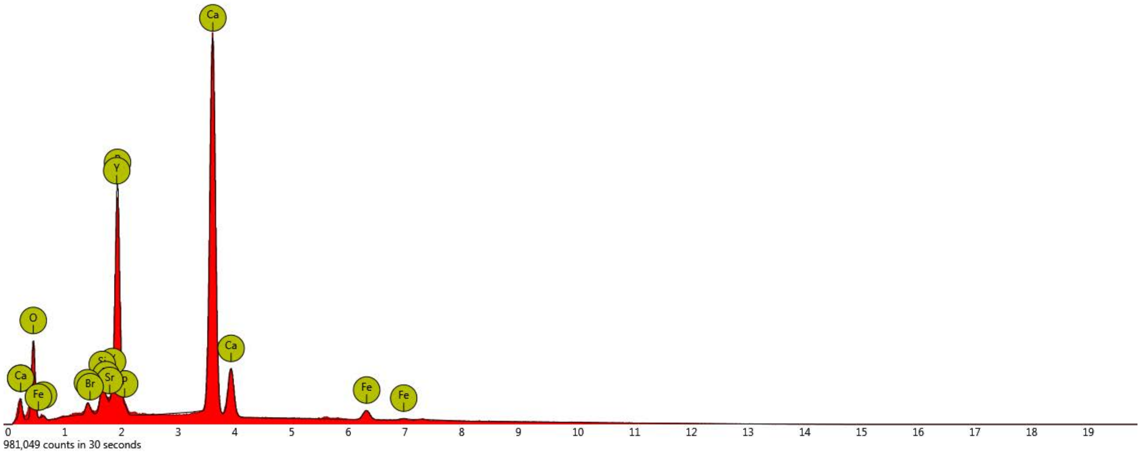

Disabled elements: B

8. spot

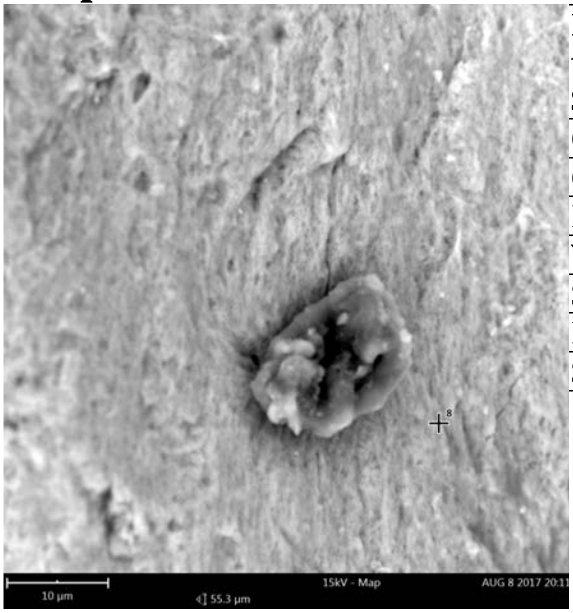

Element Symbol	Atomic Conc.	Weight Conc.	Oxide Symbol	Stoich. Conc.
O	69.96	48.80		
Ca	17.25	30.13	Ca	57.42
P	10.20	13.77	P	33.96
Y	0.70	2.70	Y	2.32
Sr	0.49	1.86	Sr	1.62
Br	0.45	1.56	Br	1.49
Si	0.96	1.17	Si	3.19

FOV: 55.3 µm, Mode: 15kV - Map, Detector: BSD Full, Time: AUG 8 2017 20:11

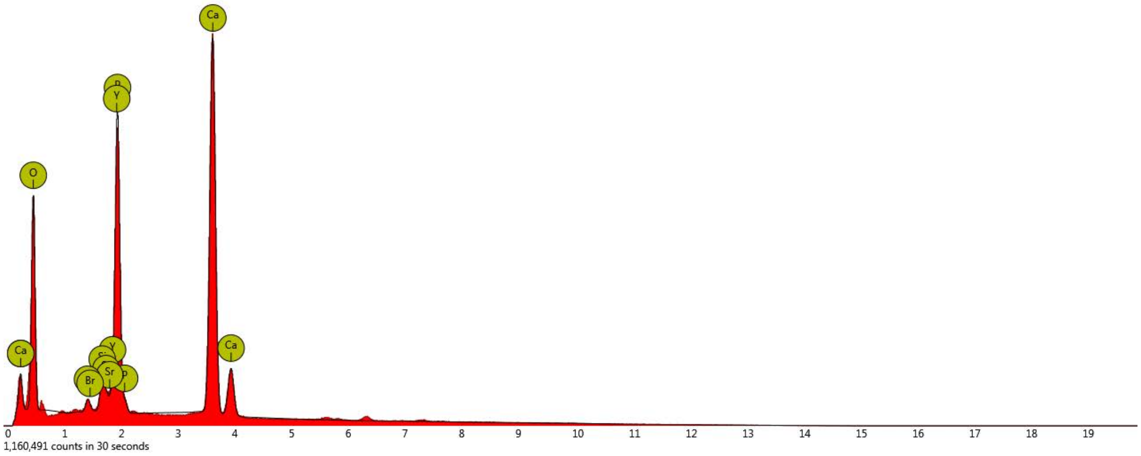

Disabled elements: B

9. spot

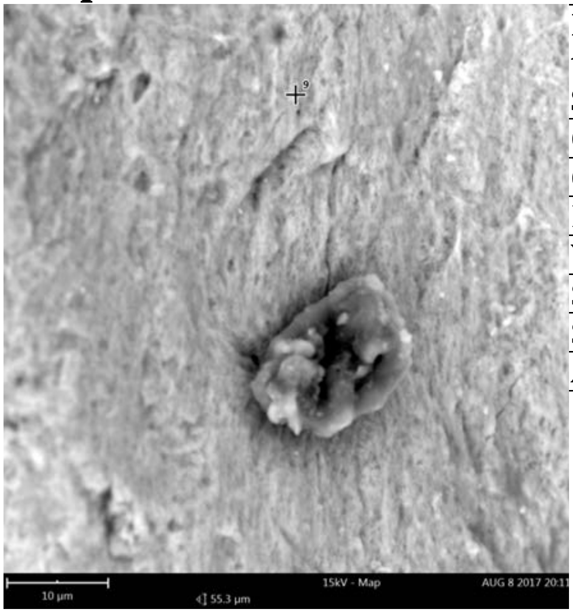

**Element Symbol**	**Atomic Conc.**	**Weight Conc.**	**Oxide Symbol**	**Stoich. Conc.**
O	70.28	49.75		
Ca	17.39	30.84	Ca	58.53
P	9.92	13.59	P	33.37
Y	0.65	2.55	Y	2.18
Sr	0.42	1.63	Sr	1.41
Si	0.87	1.09	Si	2.94
Al	0.47	0.56	Al	1.57

FOV: 55.3 µm, Mode: 15kV - Map, Detector: BSD Full, Time: AUG 8 2017 20:11

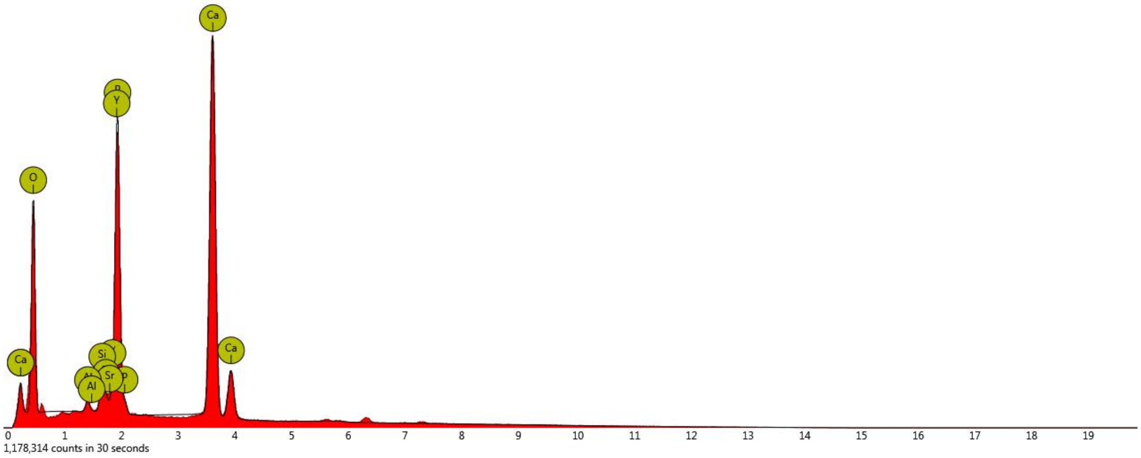

Disabled elements: B

10. spot

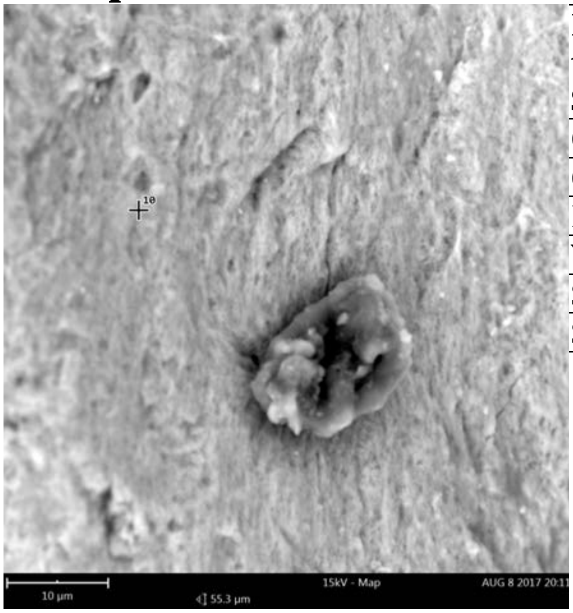

Element Symbol	Atomic Conc.	Weight Conc.	Oxide Symbol	Stoich. Conc.
O	72.99	52.92		
Ca	15.89	28.86	Ca	58.83
P	9.51	13.34	P	35.19
Y	0.62	2.49	Y	2.28
Sr	0.42	1.65	Sr	1.54
Si	0.58	0.74	Si	2.15

FOV: 55.3 µm, Mode: 15kV - Map, Detector: BSD Full, Time: AUG 8 2017 20:11

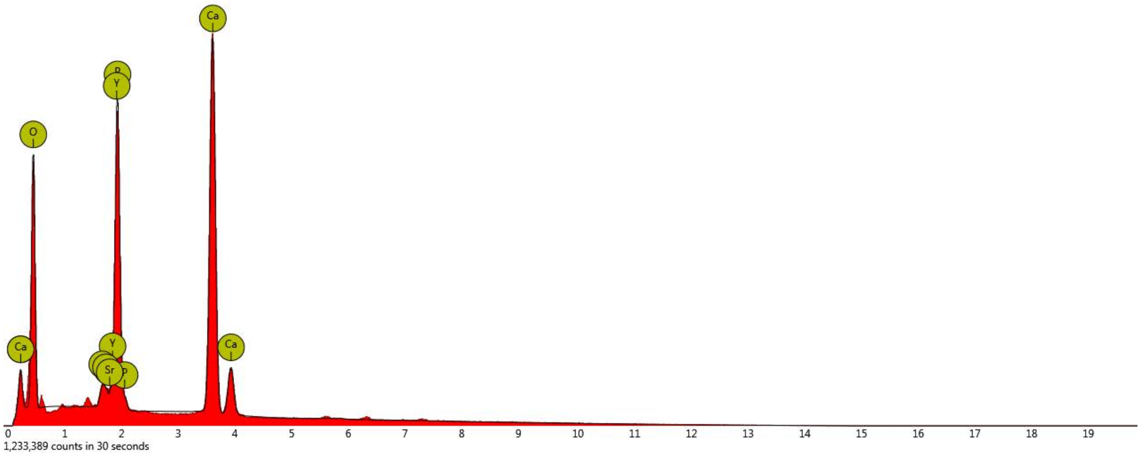

Disabled elements: B

11. spot

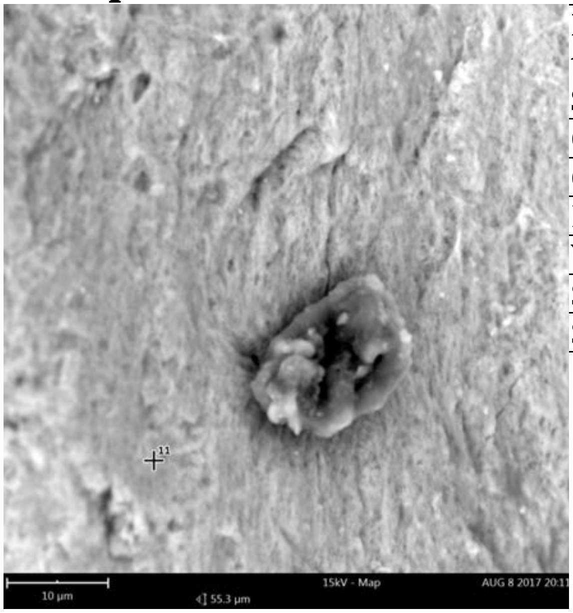

Element Symbol	Atomic Conc.	Weight Conc.	Oxide Symbol	Stoich. Conc.
O	76.98	58.22		
Ca	13.50	25.58	Ca	58.67
P	8.01	11.73	P	34.80
Y	0.54	2.29	Y	2.37
Sr	0.32	1.34	Sr	1.41
Si	0.63	0.84	Si	2.76

FOV: 55.3 µm, Mode: 15kV - Map, Detector: BSD Full, Time: AUG 8 2017 20:11

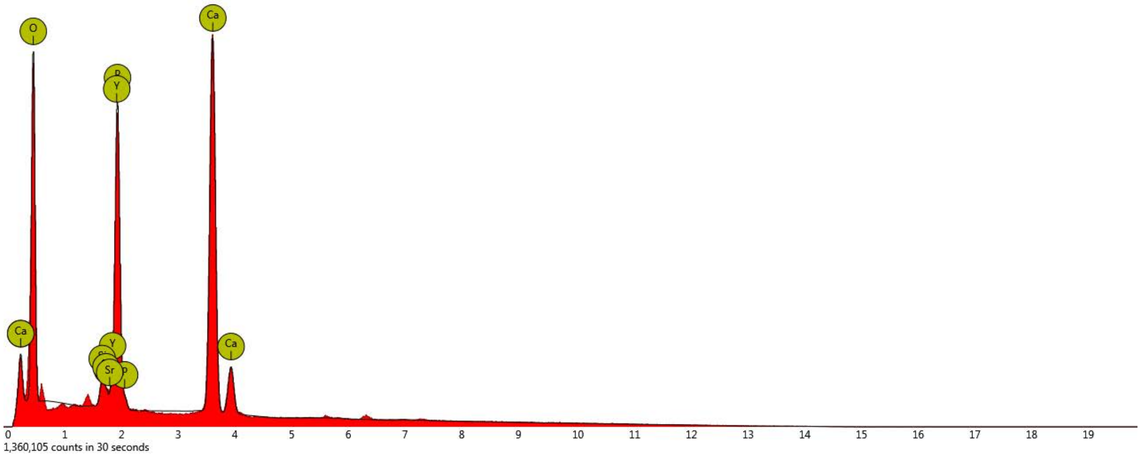

Disabled elements: B

12. spot

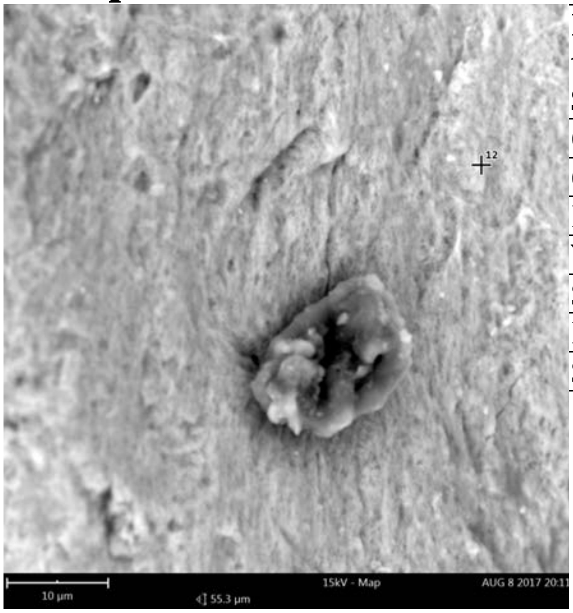

Element Symbol	Atomic Conc.	Weight Conc.	Oxide Symbol	Stoich. Conc.
O	66.55	44.92		
Ca	19.71	33.32	Ca	58.93
P	11.28	14.73	P	33.72
Y	0.74	2.77	Y	2.21
Sr	0.54	1.99	Sr	1.61
Br	0.40	1.33	Br	1.18
Si	0.79	0.93	Si	2.35

FOV: 55.3 µm, Mode: 15kV - Map, Detector: BSD Full, Time: AUG 8 2017 20:11

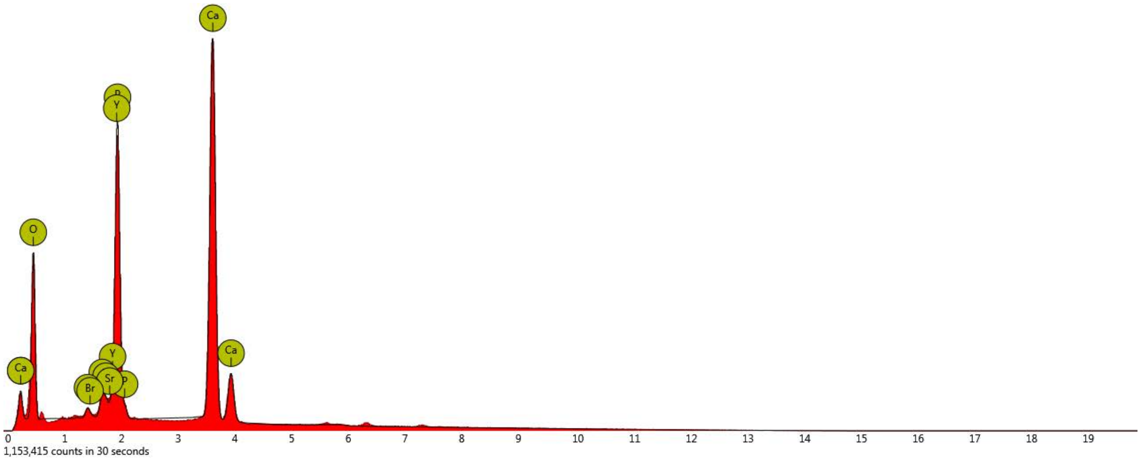

Disabled elements: B

Blood vessels embedded in bone 02

1. map

Combined map

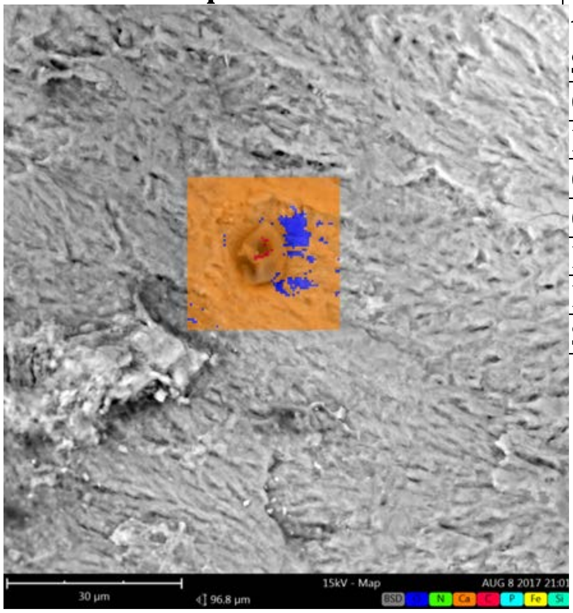

Element Symbol	Atomic Conc.	Weight Conc.	Oxide Symbol	Stoich. Conc.
O	53.95	49.53		
N	18.00	14.47	N	39.09
Ca	5.96	13.70	Ca	12.93
C	16.62	11.45	C	36.09
P	3.59	6.37	P	7.79
Fe	0.90	2.87	Fe	1.95
Si	1.00	1.61	Si	2.16

FOV: 96.8 µm, Mode: 15kV - Map, Detector: BSD Full, Time: AUG 8 2017 21:01

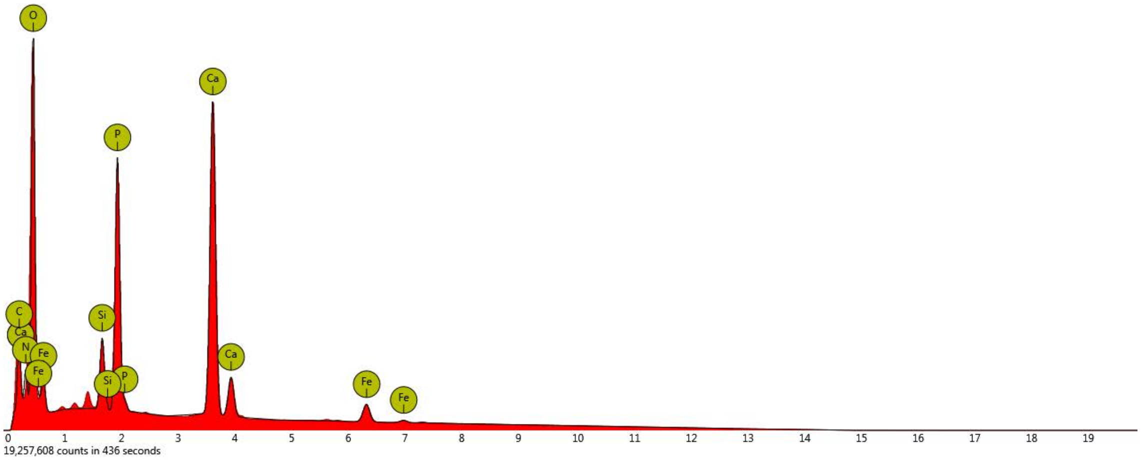

Disabled elements: B

Cut out of map (resolution: 64x64 pixels)

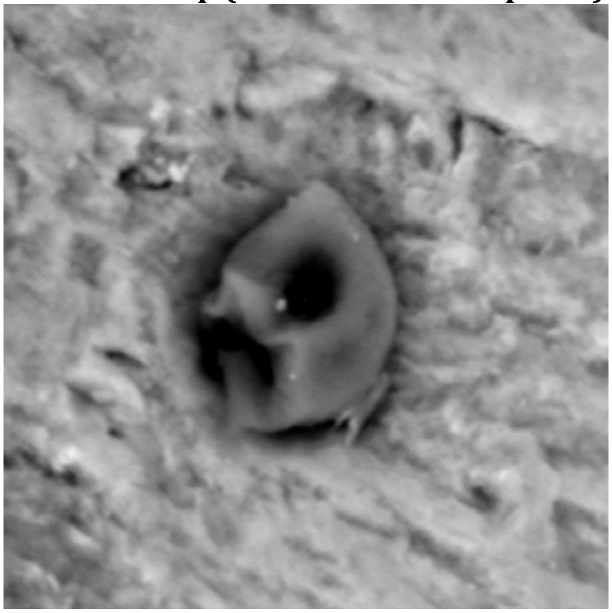

Oxygen

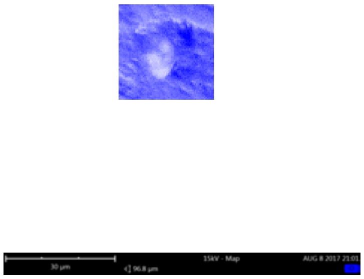

Nitrogen

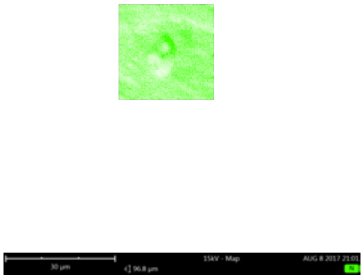

Calcium

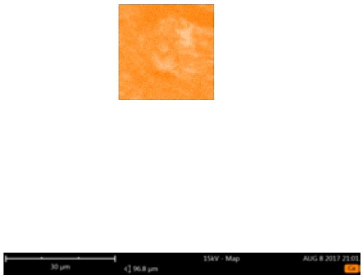

Carbon

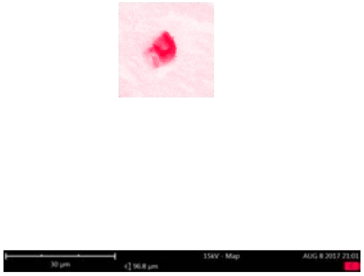

Phosphorus

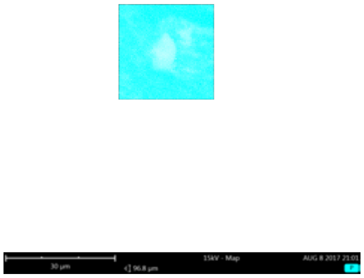

Iron

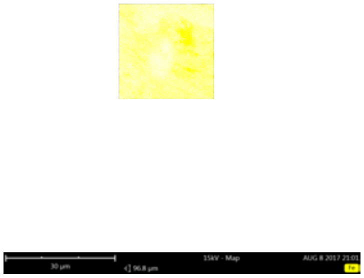

Silicon

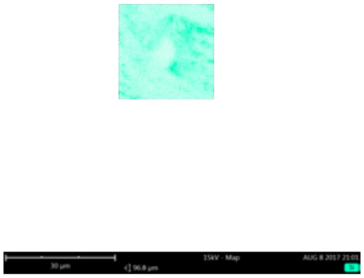

2. spot

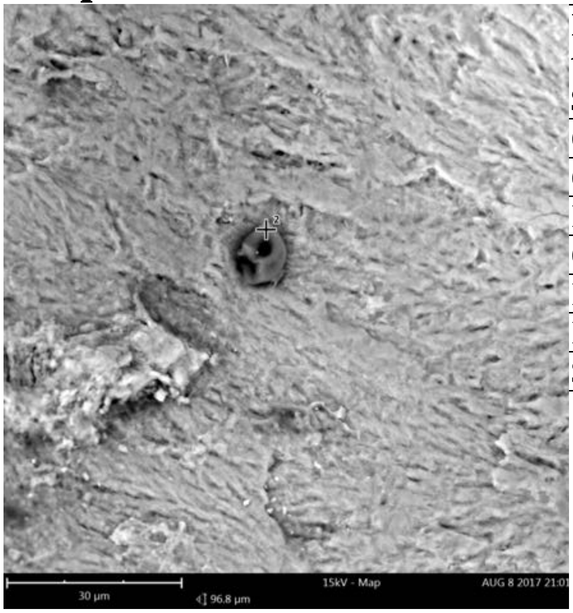

Element Symbol	Atomic Conc.	Weight Conc.	Oxide Symbol	Stoich. Conc.
O	45.62	45.85		
C	28.63	21.60	C	52.65
N	19.02	16.74	N	34.98
Ca	3.37	8.48	Ca	6.19
P	2.04	3.97	P	3.75
Fe	0.59	2.07	Fe	1.08
Si	0.74	1.30	Si	1.35

FOV: 96.8 µm, Mode: 15kV - Map, Detector: BSD Full, Time: AUG 8 2017 21:01

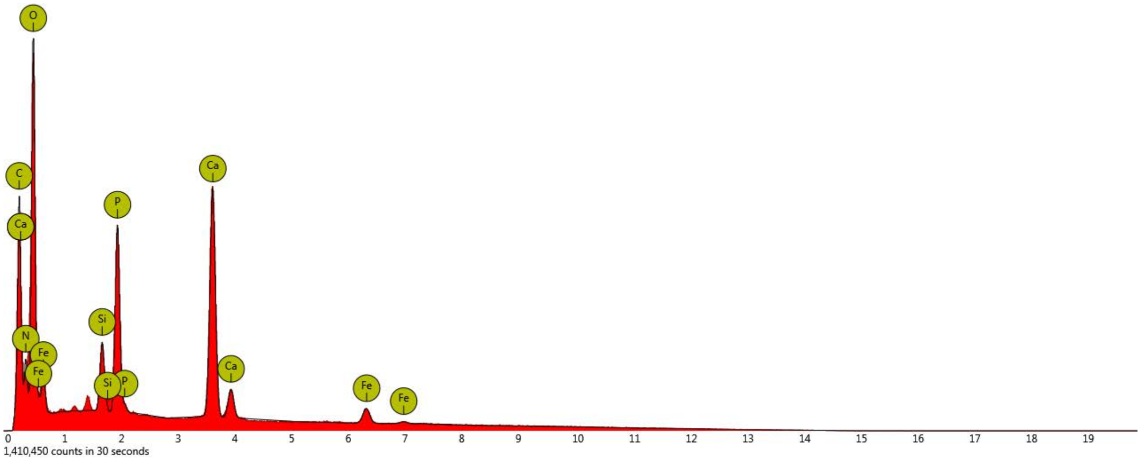

Disabled elements: B

3. spot

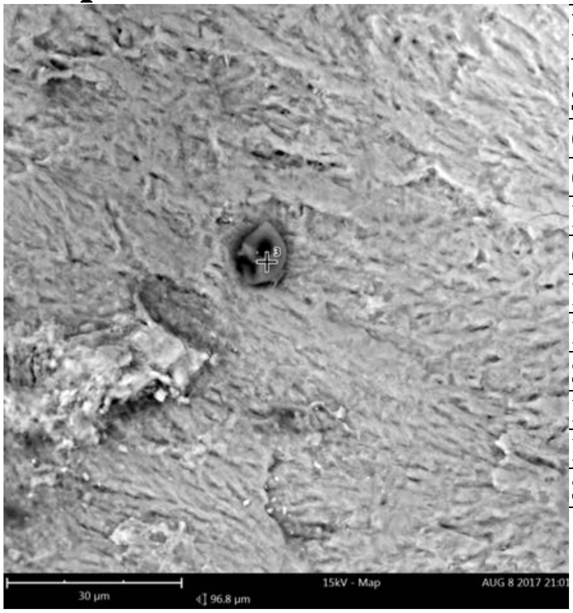

Element Symbol	Atomic Conc.	Weight Conc.	Oxide Symbol	Stoich. Conc.
C	49.70	39.35	C	71.60
O	30.59	32.27		
N	13.89	12.82	N	20.01
Ca	2.69	7.12	Ca	3.88
P	1.79	3.65	P	2.57
Fe	0.34	1.24	Fe	0.49
Si	0.58	1.07	Si	0.83
Mo	0.16	1.03	Mo	0.23
Br	0.15	0.81	Br	0.22
Sr	0.11	0.63	Sr	0.16

FOV: 96.8 μm, Mode: 15kV - Map, Detector: BSD Full, Time: AUG 8 2017 21:01

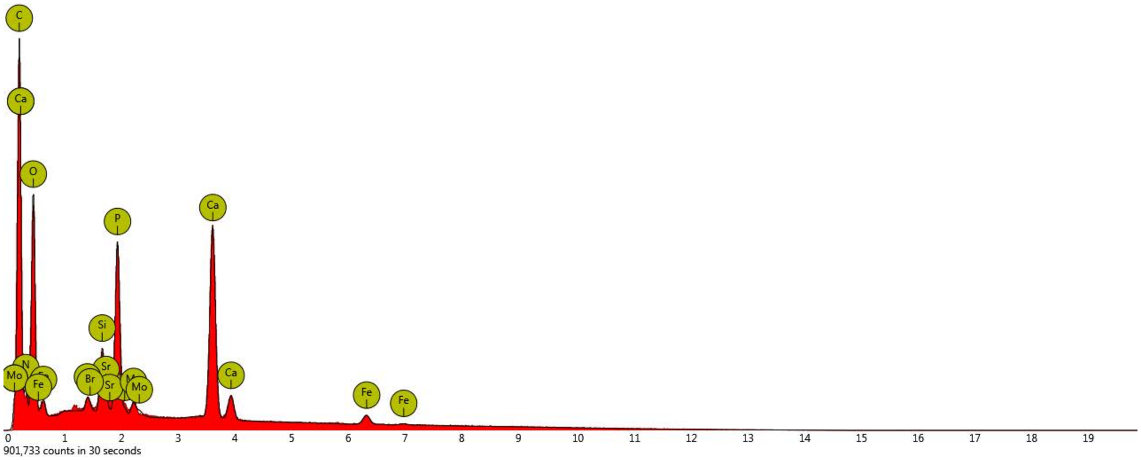

Disabled elements: B

4. spot

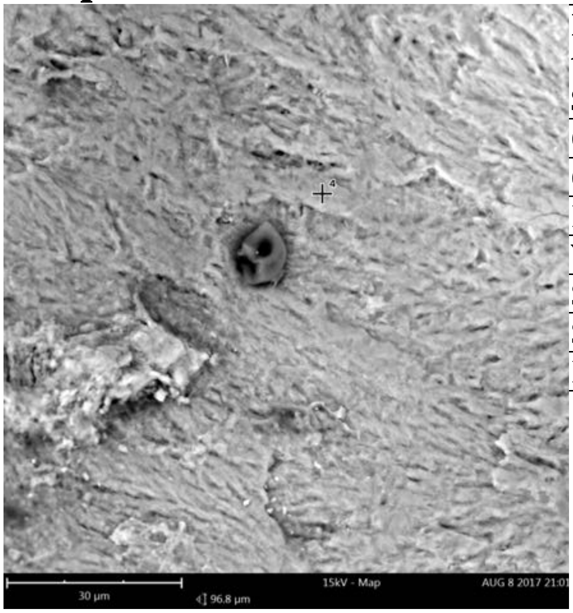

Element Symbol	Atomic Conc.	Weight Conc.	Oxide Symbol	Stoich. Conc.
O	69.32	48.19		
Ca	17.62	30.69	Ca	57.43
P	10.53	14.17	P	34.31
Y	0.71	2.75	Y	2.32
Sr	0.48	1.82	Sr	1.56
Si	1.02	1.24	Si	3.31
Br	0.33	1.14	Br	1.07

FOV: 96.8 µm, Mode: 15kV - Map, Detector: BSD Full, Time: AUG 8 2017 21:01

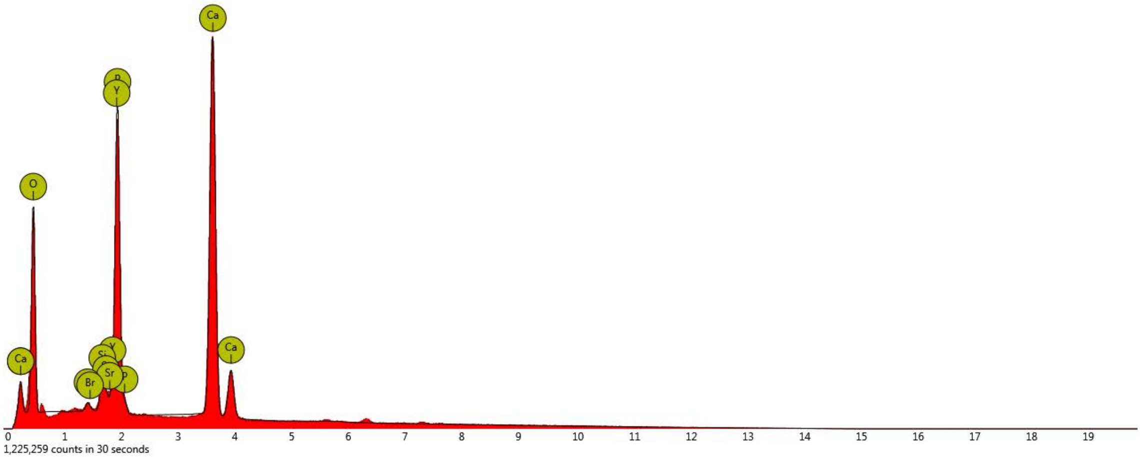

Disabled elements: B

5. spot

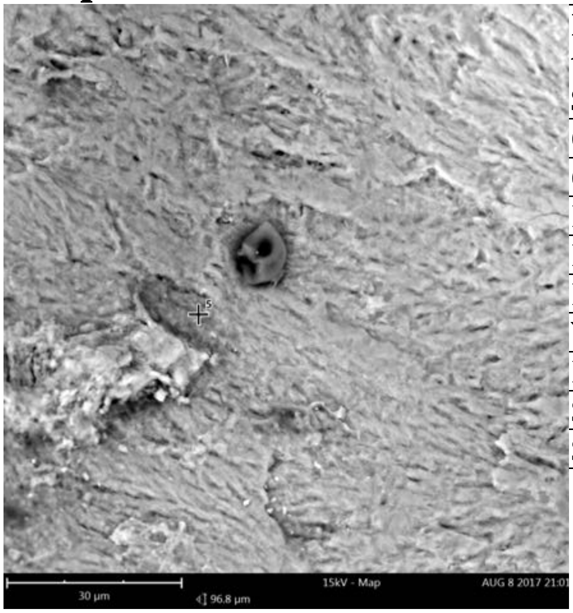

Elemen<br>t<br>Symbol	Atomi<br>c<br>Conc.	Weigh<br>t<br>Conc.	Oxide<br>Symbo<br>l	Stoich<br>.<br>Conc.
O	63.77	49.04		
Ca	11.11	21.41	Ca	30.68
P	7.03	10.46	P	19.40
N	14.83	9.98	N	40.92
Fe	1.10	2.95	Fe	3.03
Y	0.48	2.06	Y	1.33
Br	0.37	1.40	Br	1.01
Sr	0.32	1.36	Sr	0.89
Si	0.99	1.34	Si	2.74

FOV: 96.8 µm, Mode: 15kV - Map, Detector: BSD Full, Time: AUG 8 2017 21:01

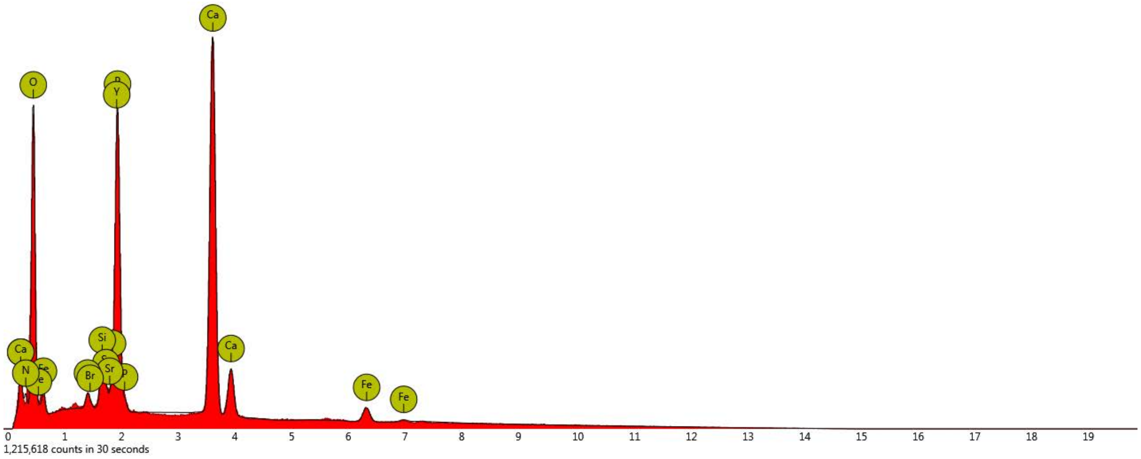

Disabled elements: B

6. spot

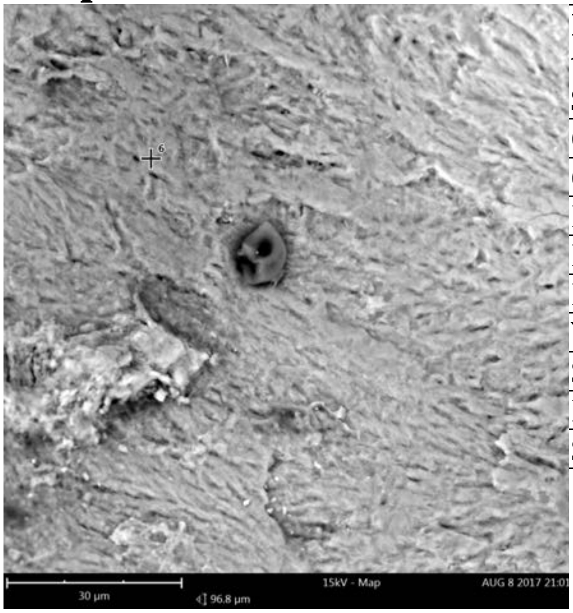

Element Symbol	Atomic Conc.	Weight Conc.	Oxide Symbol	Stoich. Conc.
O	63.63	48.74		
Ca	11.02	21.14	Ca	30.29
P	6.81	10.10	P	18.72
N	14.70	9.86	N	40.41
Fe	1.49	3.99	Fe	4.11
Y	0.45	1.91	Y	1.23
Si	1.27	1.70	Si	3.48
Br	0.35	1.34	Br	0.96
Sr	0.29	1.22	Sr	0.80

FOV: 96.8 µm, Mode: 15kV - Map, Detector: BSD Full, Time: AUG 8 2017 21:01

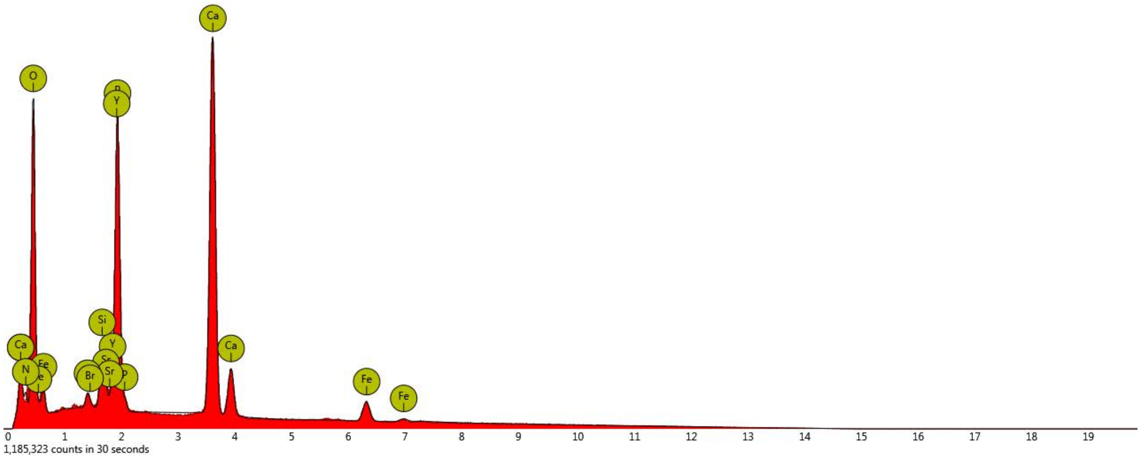

Disabled elements: B

7. spot

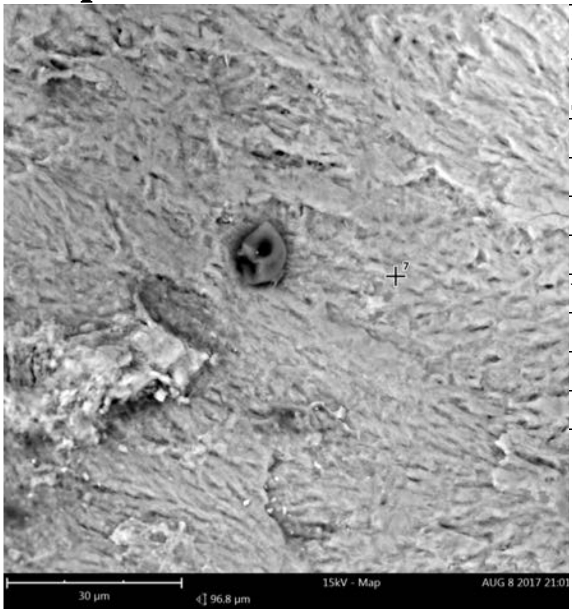

Element Symbol	Atomic Conc.	Weight Conc.	Oxide Symbol	Stoich. Conc.
O	62.61	48.49		
Ca	12.48	24.21	Ca	33.38
P	7.42	11.12	P	19.84
N	15.57	10.55	N	41.64
Y	0.49	2.13	Y	1.32
Sr	0.34	1.45	Sr	0.92
Fe	0.43	1.16	Fe	1.15
Si	0.66	0.89	Si	1.76

FOV: 96.8 µm, Mode: 15kV - Map, Detector: BSD Full, Time: AUG 8 2017 21:01

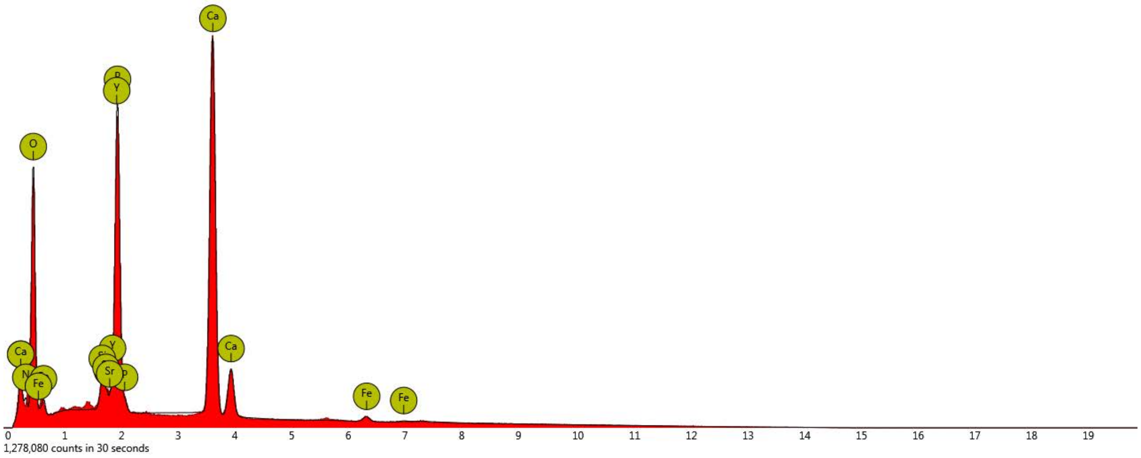

Disabled elements: B

Blood vessels and osteocytes embedded in bone 03

Combined map

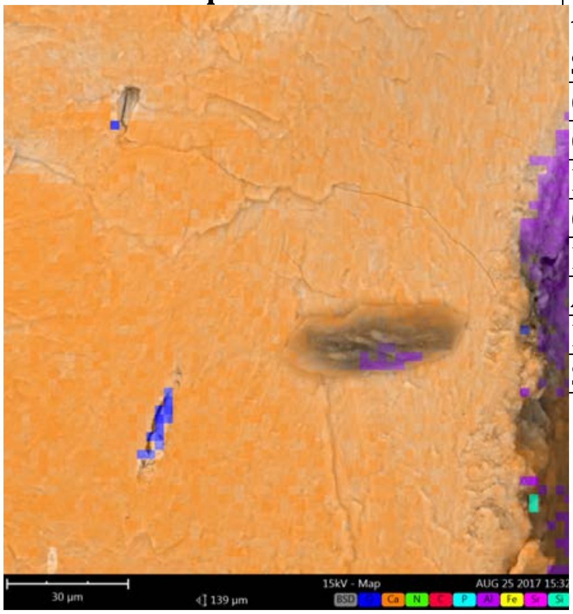

Element Symbol	Atomic Conc.	Weight Conc.	Oxide Symbol	Stoich. Conc.
O	39.14	32.69		
Ca	10.85	22.71	Ca	17.83
N	24.60	17.99	N	40.42
C	15.73	9.86	C	25.84
P	5.30	8.58	P	8.71
Al	3.08	4.34	Al	5.06
Fe	0.73	2.14	Fe	1.21
Sr	0.28	1.28	Sr	0.46

FOV: 139 µm, Mode: 15kV - Map, Detector: BSD Full, Time: AUG 25 2017 15:32

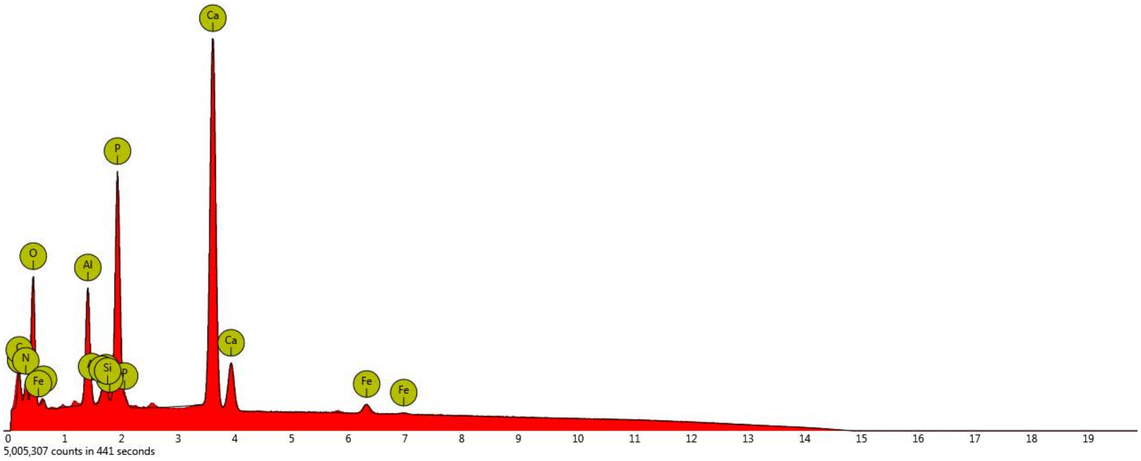

Disabled elements: B, Y

Cut out of map (resolution: 64x64 pixels)

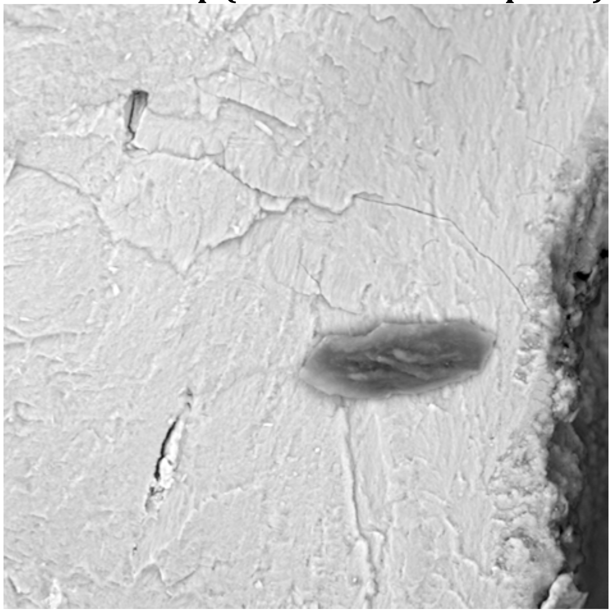

Oxygen

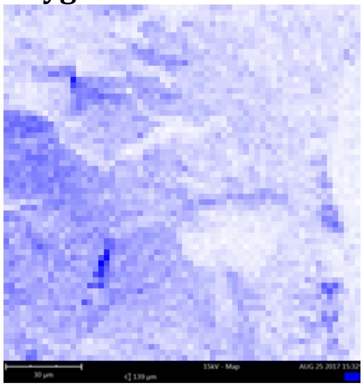

Calcium

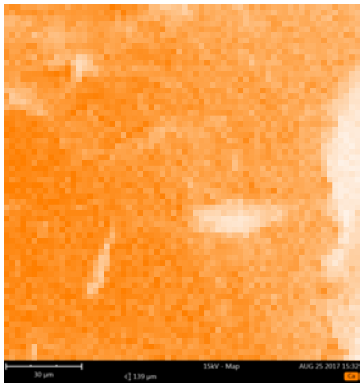

Nitrogen

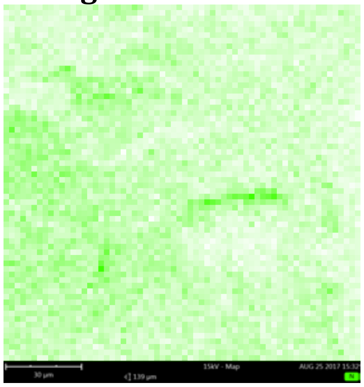

Carbon

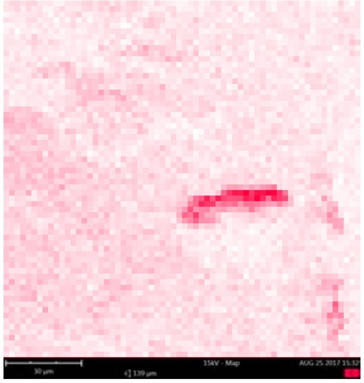

Phosphorus

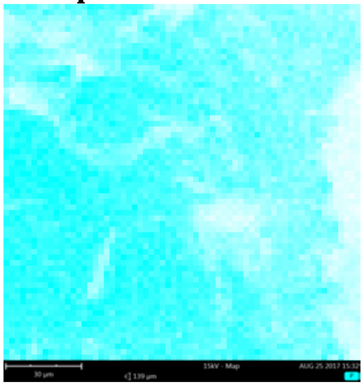

Aluminium

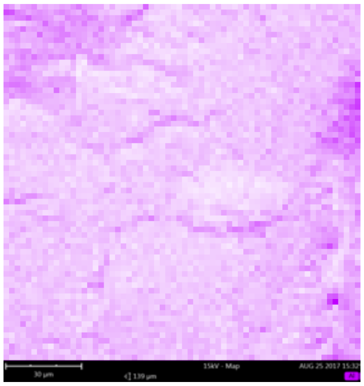

Iron

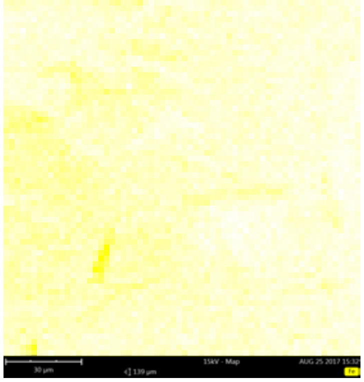

Strontium

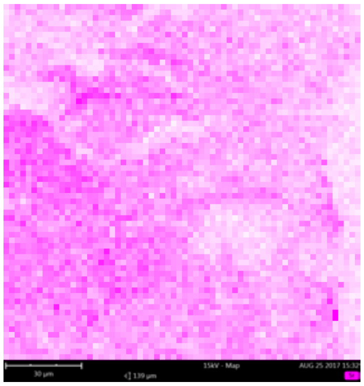

Silicon

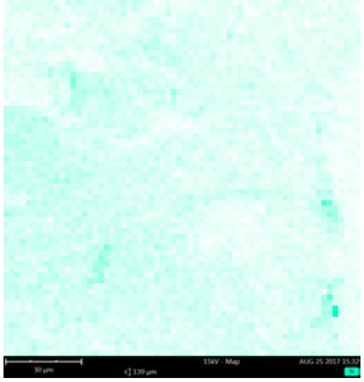

2. spot

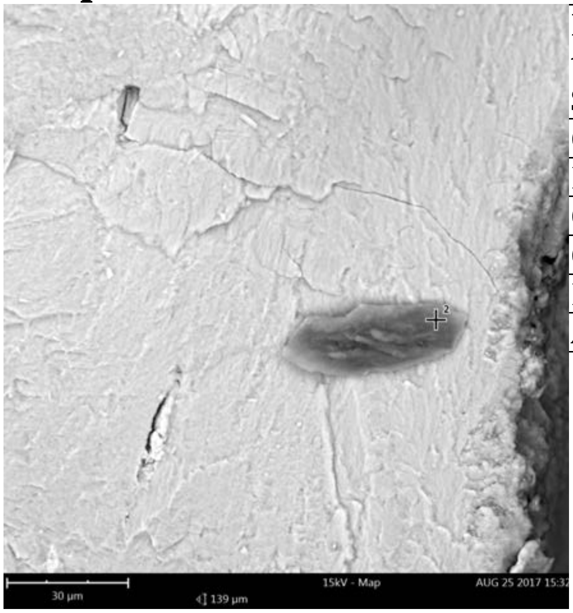

Element Symbol	Atomic Conc.	Weight Conc.	Oxide Symbol	Stoich. Conc.
C	40.53	31.91	C	51.49
N	30.89	28.36	N	39.23
O	21.27	22.31		
Ca	4.69	12.33	Ca	5.96
P	1.82	3.69	P	2.31
Al	0.80	1.41	Al	1.01

FOV: 139 μm, Mode: 15kV - Map, Detector: BSD Full, Time: AUG 25 2017 15:32

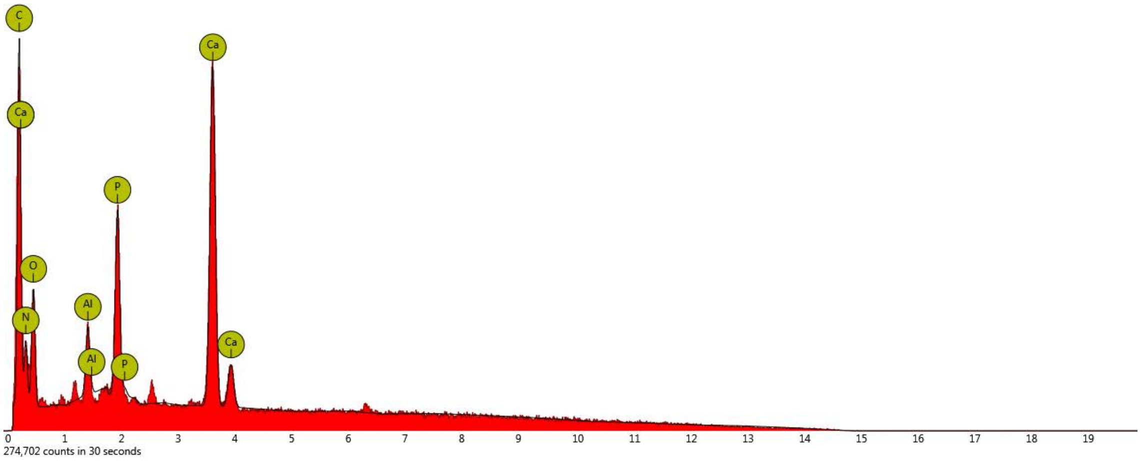

Disabled elements: B, Y

3. spot

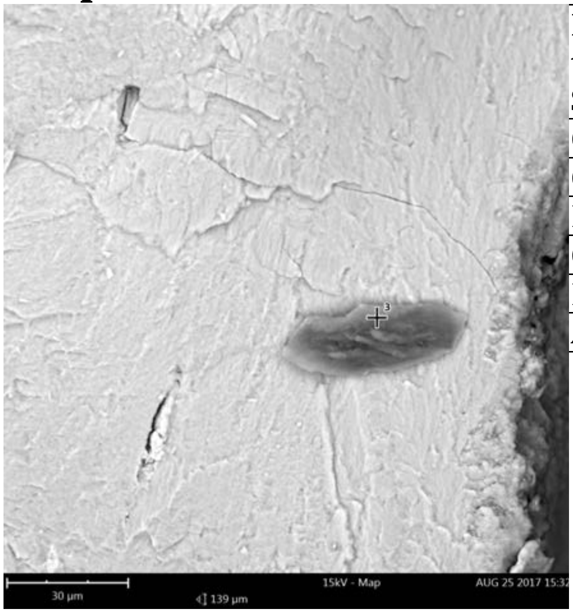

Element Symbol	Atomic Conc.	Weight Conc.	Oxide Symbol	Stoich. Conc.
C	43.61	35.38	C	58.14
O	25.00	27.01		
N	26.11	24.70	N	34.82
Ca	3.23	8.76	Ca	4.31
P	1.56	3.25	P	2.07
Al	0.49	0.90	Al	0.66

FOV: 139 μm, Mode: 15kV - Map, Detector: BSD Full, Time: AUG 25 2017 15:32

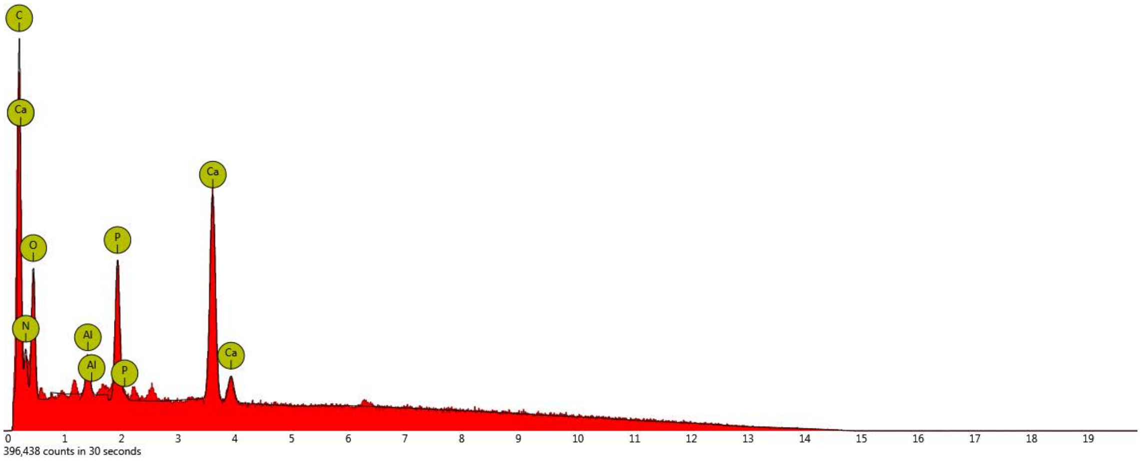

Disabled elements: B

4. spot

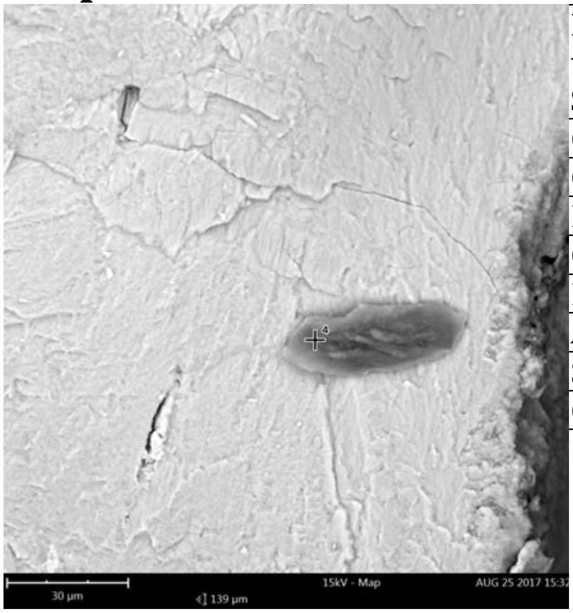

Element Symbol	Atomic Conc.	Weight Conc.	Oxide Symbol	Stoich. Conc.
C	39.93	30.08	C	55.62
O	28.21	28.31		
N	22.34	19.62	N	31.11
Ca	5.04	12.67	Ca	7.02
P	2.60	5.05	P	3.62
Al	1.18	2.01	Al	1.65
Sr	0.21	1.17	Sr	0.30
Cl	0.49	1.09	Cl	0.68

FOV: 139 μm, Mode: 15kV - Map, Detector: BSD Full, Time: AUG 25 2017 15:32

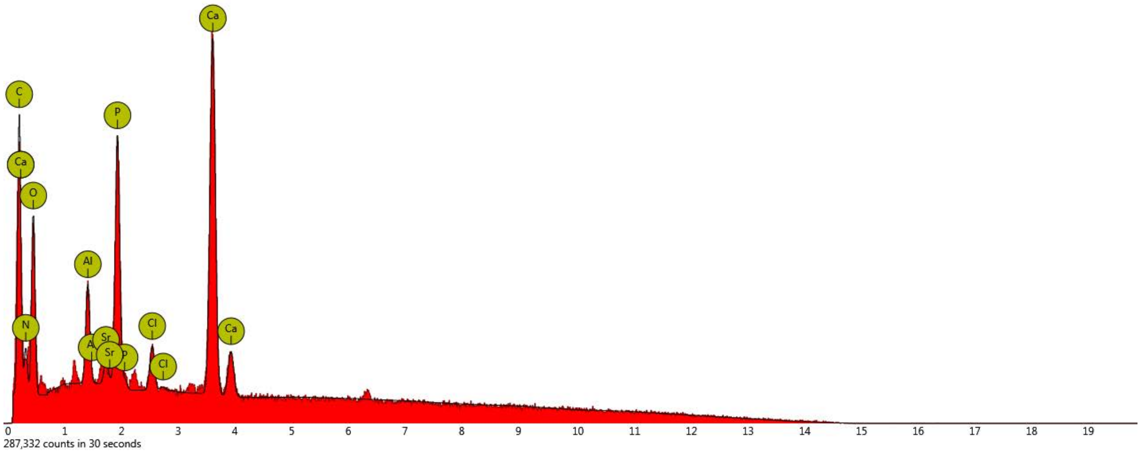

Disabled elements: B

5. spot

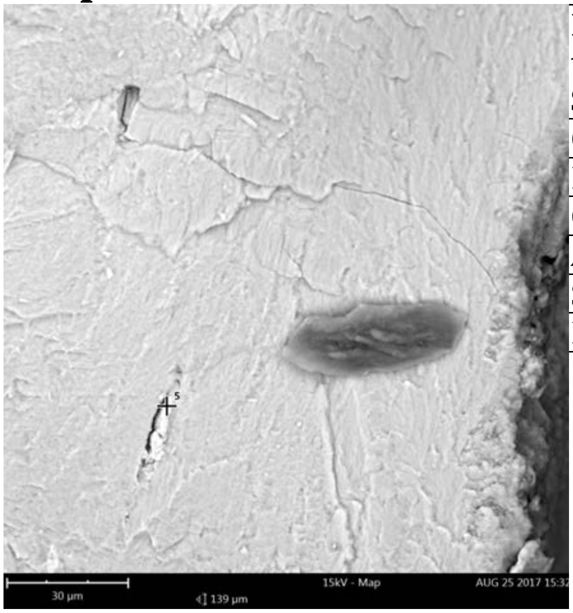

Element Symbol	Atomic Conc.	Weight Conc.	Oxide Symbol	Stoich. Conc.
O	73.21	51.67		
Fe	10.29	25.36	Fe	38.43
Ca	4.55	8.04	Ca	16.98
Al	5.25	6.25	Al	19.60
Si	3.61	4.48	Si	13.49
P	3.08	4.21	P	11.50

FOV: 139 µm, Mode: 15kV - Map, Detector: BSD Full, Time: AUG 25 2017 15:32

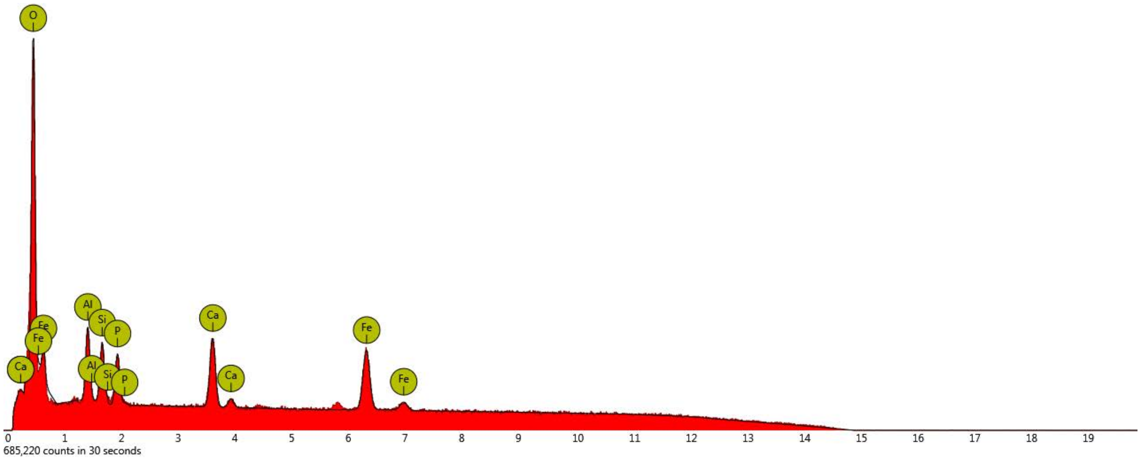

Disabled elements: B

6. spot

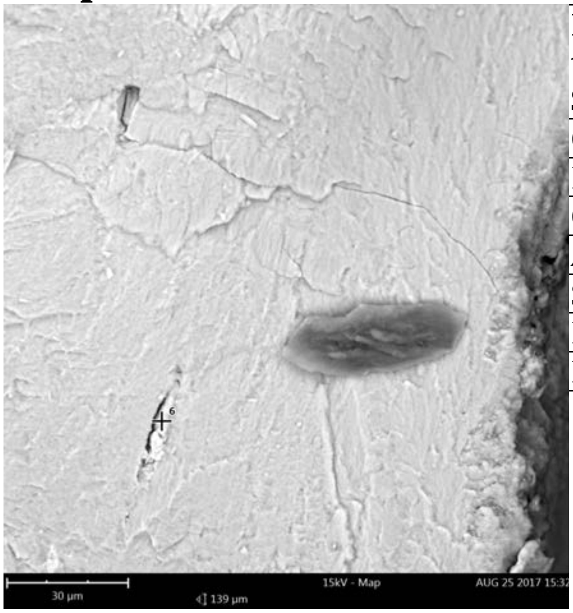

Element Symbol	Atomic Conc.	Weight Conc.	Oxide Symbol	Stoich. Conc.
O	71.99	49.63		
Fe	10.63	25.59	Fe	37.97
Ca	4.15	7.17	Ca	14.83
Al	5.08	5.91	Al	18.15
Si	4.02	4.86	Si	14.34
P	2.82	3.76	P	10.06
Mn	1.30	3.08	Mn	4.65

FOV: 139 µm, Mode: 15kV - Map, Detector: BSD Full, Time: AUG 25 2017 15:32

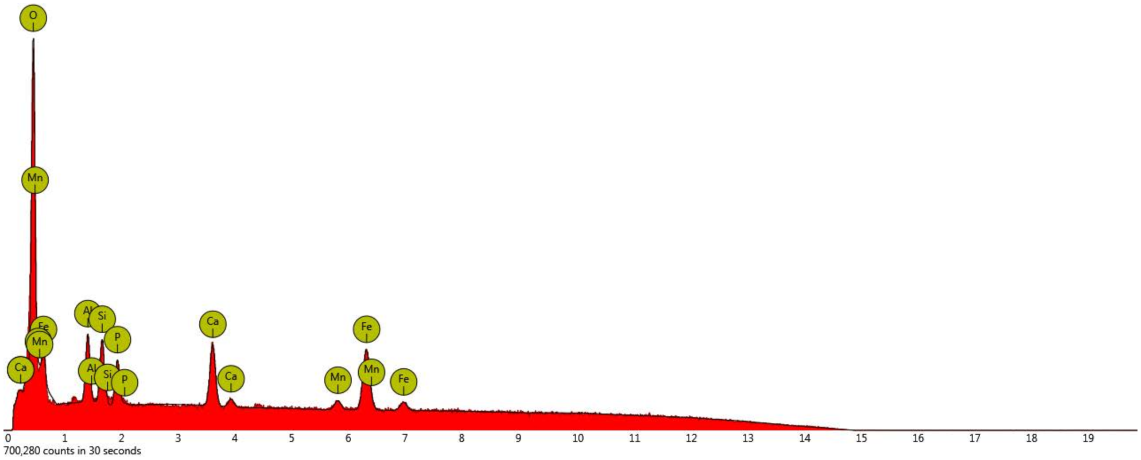

Disabled elements: B

7. spot

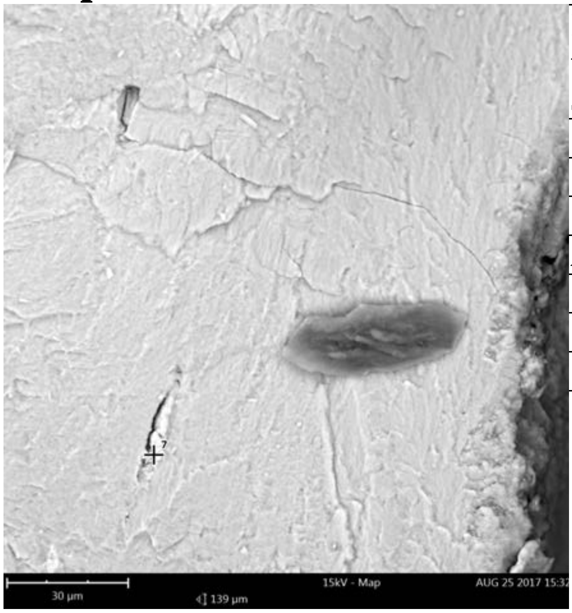

Element Symbol	Atomic Conc.	Weight Conc.	Oxide Symbol	Stoich. Conc.
O	63.45	40.83		
Fe	8.88	19.95	Fe	24.31
Ca	6.75	10.89	Ca	18.48
Al	9.36	10.15	Al	25.60
Mn	4.32	9.54	Mn	11.81
P	4.05	5.05	P	11.09
Si	3.18	3.59	Si	8.71

FOV: 139 µm, Mode: 15kV - Map, Detector: BSD Full, Time: AUG 25 2017 15:32

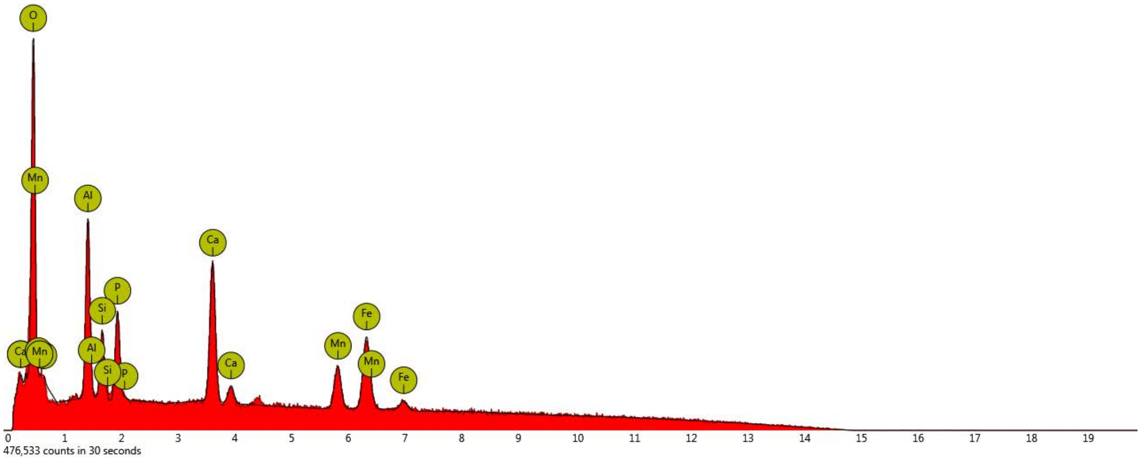

Disabled elements: B

8. spot

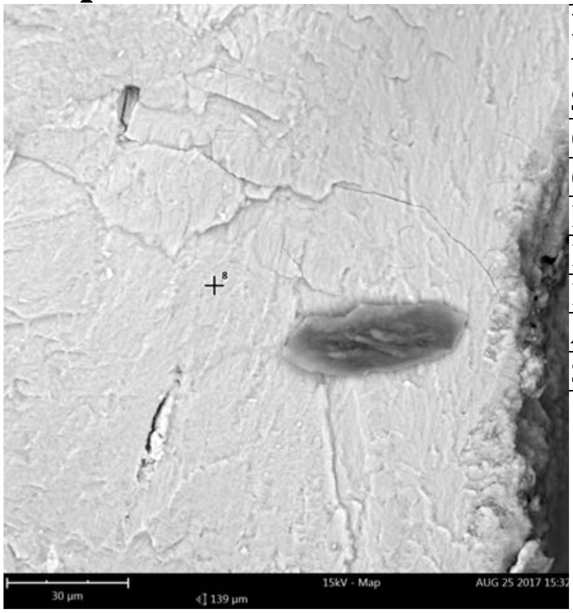

Element Symbol	Atomic Conc.	Weight Conc.	Oxide Symbol	Stoich. Conc.
O	45.78	34.92		
Ca	14.16	27.05	Ca	26.11
N	29.07	19.41	N	53.62
P	7.31	10.79	P	13.48
Fe	1.11	2.96	Fe	2.05
Al	1.75	2.25	Al	3.23
Sr	0.54	2.25	Sr	0.99

FOV: 139 µm, Mode: 15kV - Map, Detector: BSD Full, Time: AUG 25 2017 15:32

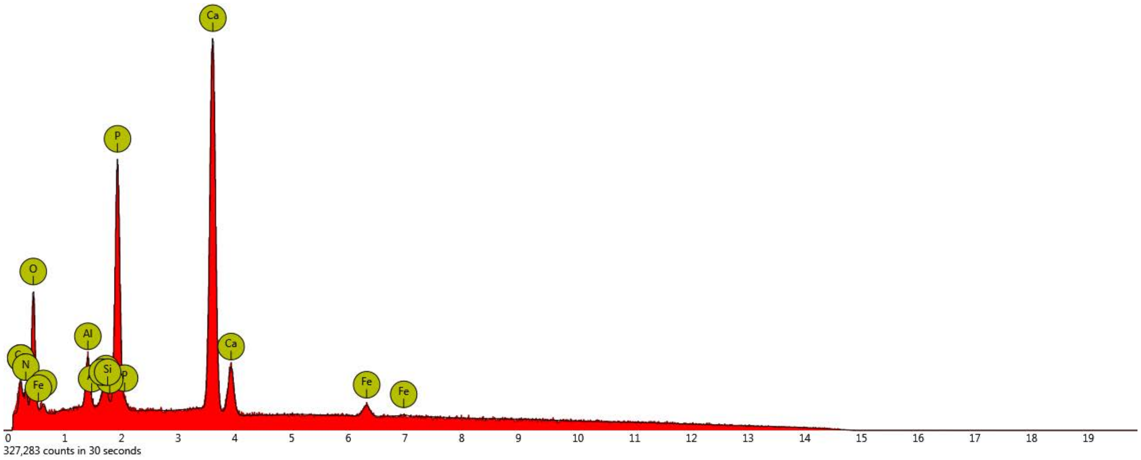

Disabled elements: B, Y

9. spot

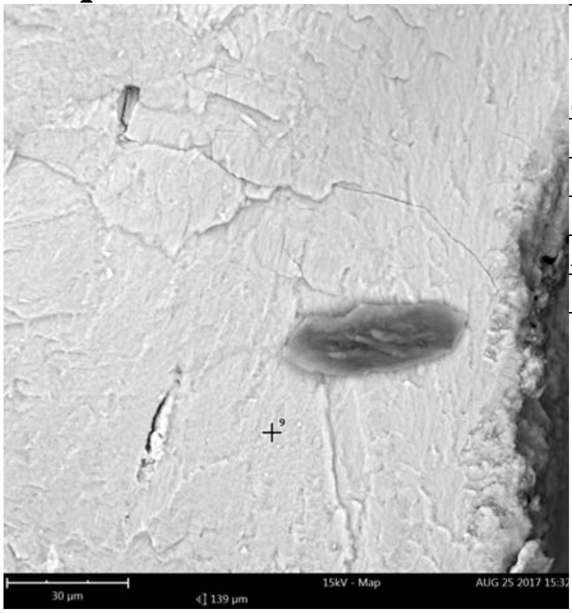

Element Symbol	Atomic Conc.	Weight Conc.	Oxide Symbol	Stoich. Conc.
O	71.62	52.27		
Ca	15.92	29.11	Ca	56.11
P	9.24	13.05	P	32.55
Al	2.64	3.25	Al	9.29
Sr	0.58	2.32	Sr	2.04

FOV: 139 µm, Mode: 15kV - Map, Detector: BSD Full, Time: AUG 25 2017 15:32

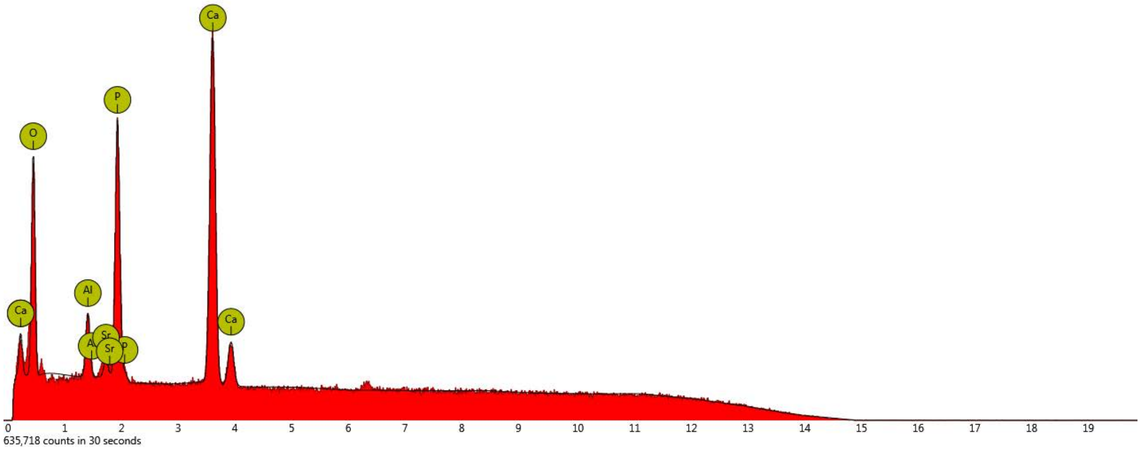

Disabled elements: B, Y

10. spot

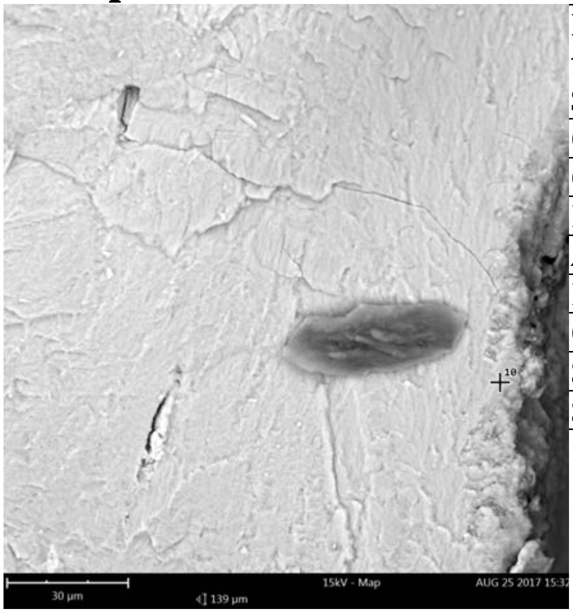

Element Symbol	Atomic Conc.	Weight Conc.	Oxide Symbol	Stoich. Conc.
Ca	26.14	38.94	Ca	49.18
O	46.84	27.85		
P	11.61	13.37	P	21.84
Al	9.82	9.84	Al	18.47
Fe	2.29	4.75	Fe	4.30
Cl	1.99	2.62	Cl	3.74
Sr	0.57	1.85	Sr	1.07
Si	0.75	0.78	Si	1.40

FOV: 139 µm, Mode: 15kV - Map, Detector: BSD Full, Time: AUG 25 2017 15:32

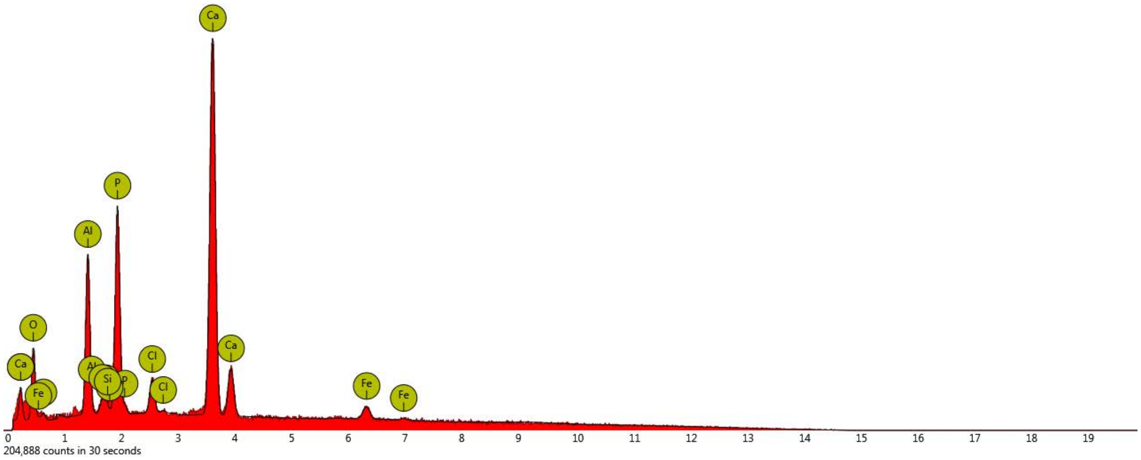

Disabled elements: Am, B, In, N, Sn, Y

Podocnemis lewyana (Uncatalogued specimen)

Osteocytes embedded in bone 01

1. map

Combined map

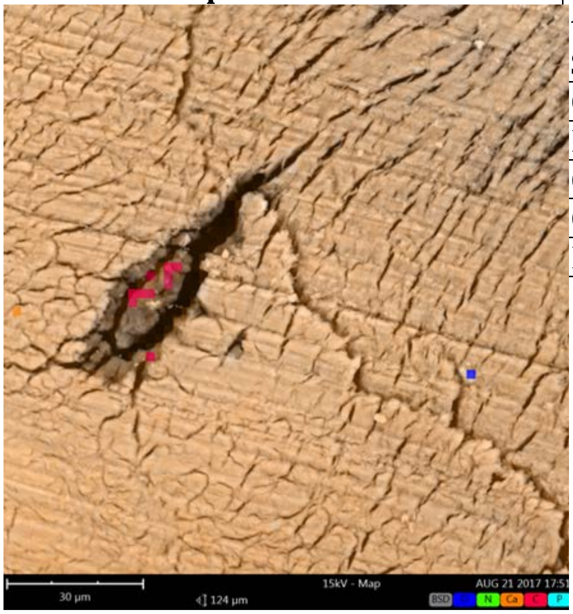

Element Symbol	Atomic Conc.	Weight Conc.	Oxide Symbol	Stoich. Conc.
O	38.50	35.96		
N	24.14	19.74	N	39.25
Ca	8.38	19.61	Ca	13.63
C	25.03	17.55	C	40.70
P	3.95	7.14	P	6.42

FOV: 124 µm, Mode: 15kV - Map, Detector: BSD Full, Time: AUG 21 2017 17:51

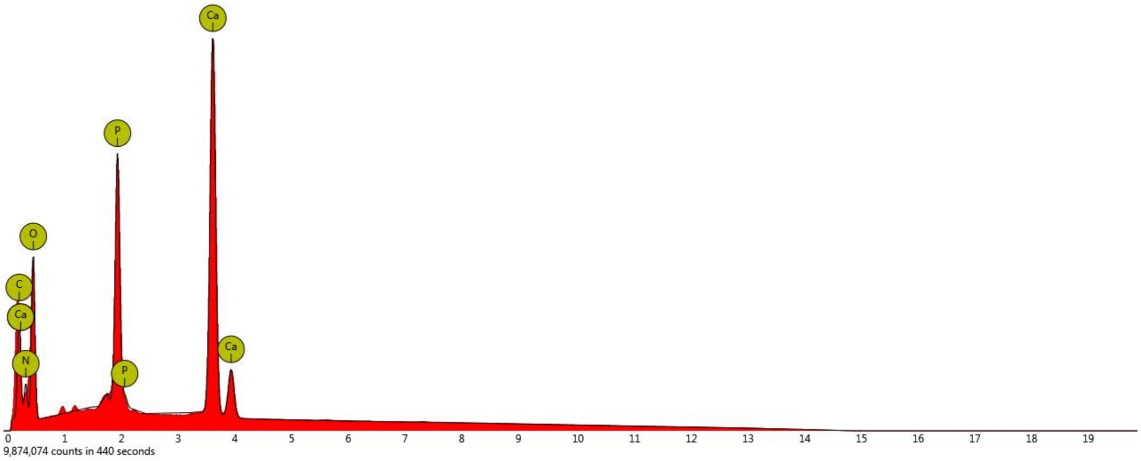

Disabled elements: B, Te

Cut out of map (resolution: 64x64 pixels)

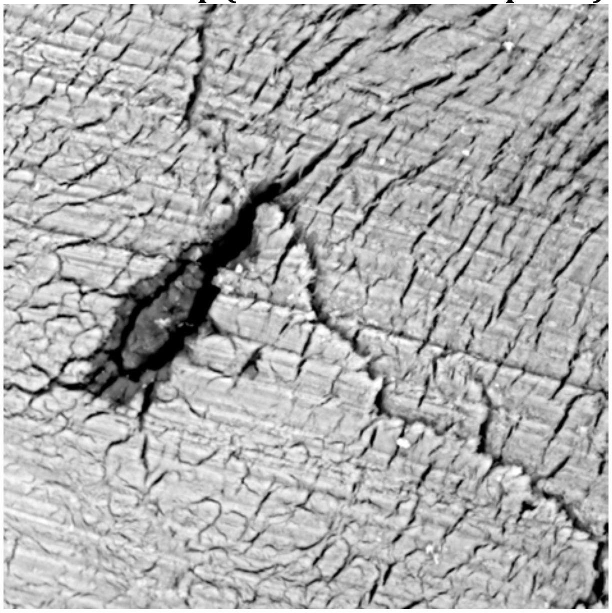

Oxygen

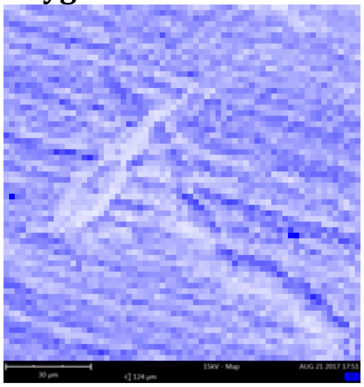

Nitrogen

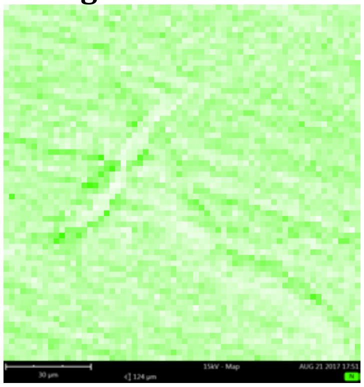

Calcium

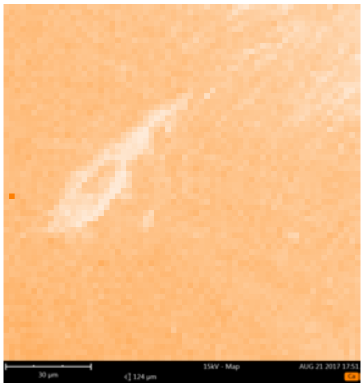

Carbon

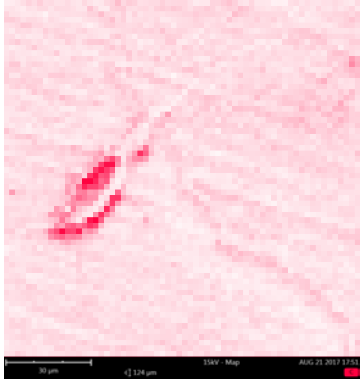

Phosphorus

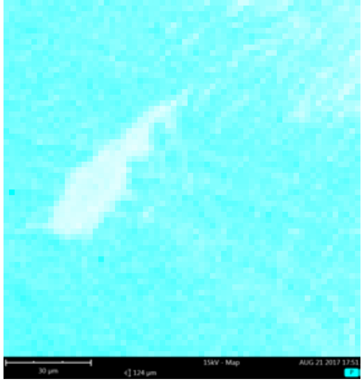

2. spot

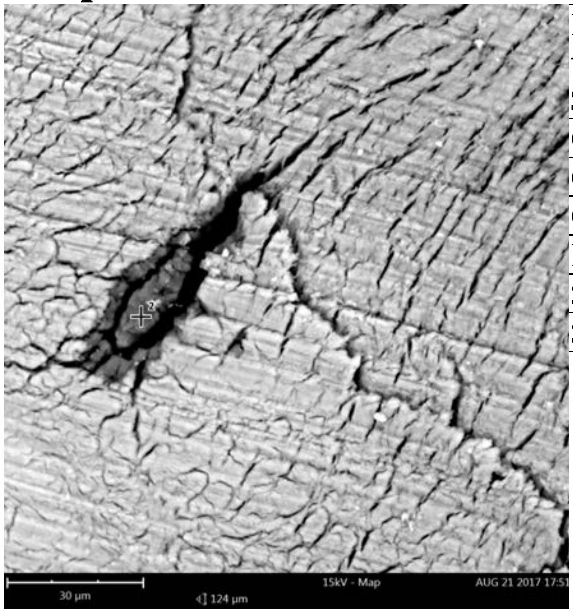

Element Symbol	Atomic Conc.	Weight Conc.	Oxide Symbol	Stoich. Conc.
C	63.91	48.22	C	85.13
O	24.93	25.06		
Ca	7.20	18.12	Ca	9.59
P	3.01	5.85	P	4.01
S	0.81	1.62	S	1.07
Sn	0.15	1.12	Sn	0.20

FOV: 124 µm, Mode: 15kV - Map, Detector: BSD Full, Time: AUG 21 2017 17:51

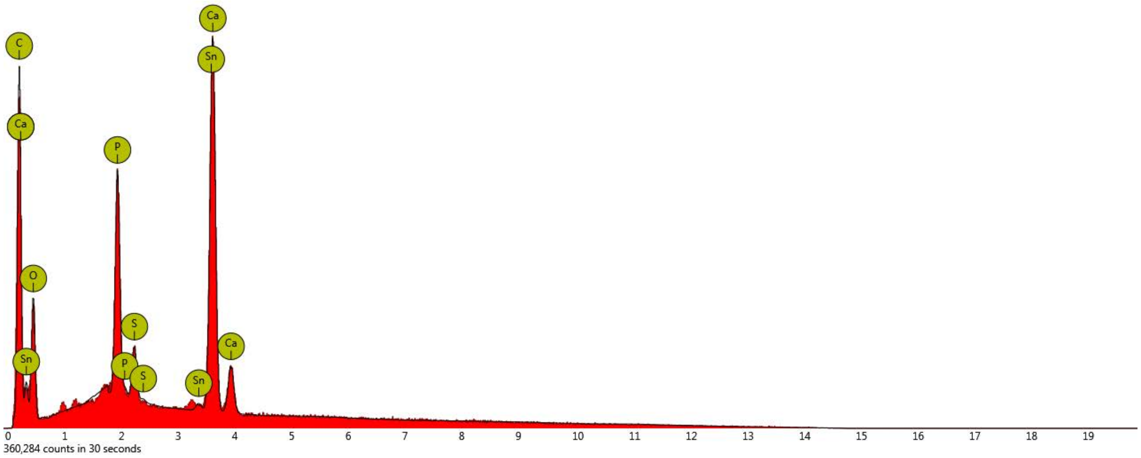

Disabled elements: B

3. spot

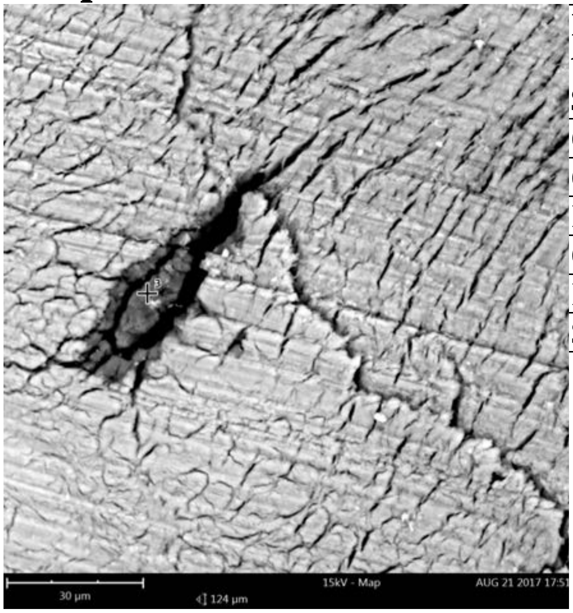

Element Symbol	Atomic Conc.	Weight Conc.	Oxide Symbol	Stoich. Conc.
C	52.99	45.20	C	66.71
O	20.57	23.37		
N	23.25	23.13	N	29.27
Ca	1.94	5.52	Ca	2.44
P	0.96	2.11	P	1.20
S	0.30	0.68	S	0.38

FOV: 124 μm, Mode: 15kV - Map, Detector: BSD Full, Time: AUG 21 2017 17:51

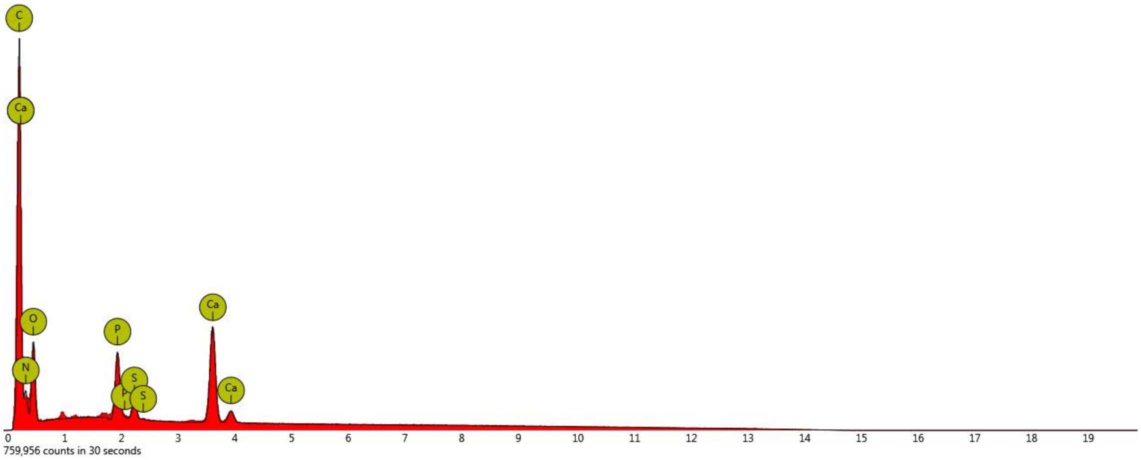

Disabled elements: B

4. spot

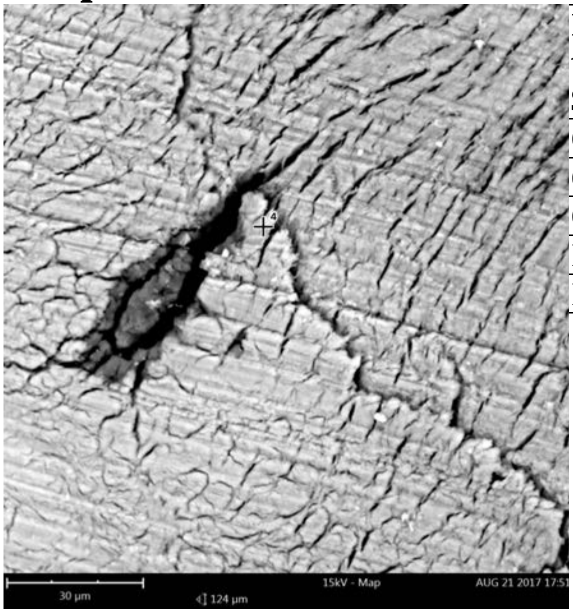

Element Symbol	Atomic Conc.	Weight Conc.	Oxide Symbol	Stoich. Conc.
O	39.49	37.21		
C	28.32	20.03	C	46.80
Ca	7.72	18.23	Ca	12.76
N	20.09	16.57	N	33.19
P	4.11	7.50	P	6.80

FOV: 124 µm, Mode: 15kV - Map, Detector: BSD Full, Time: AUG 21 2017 17:51

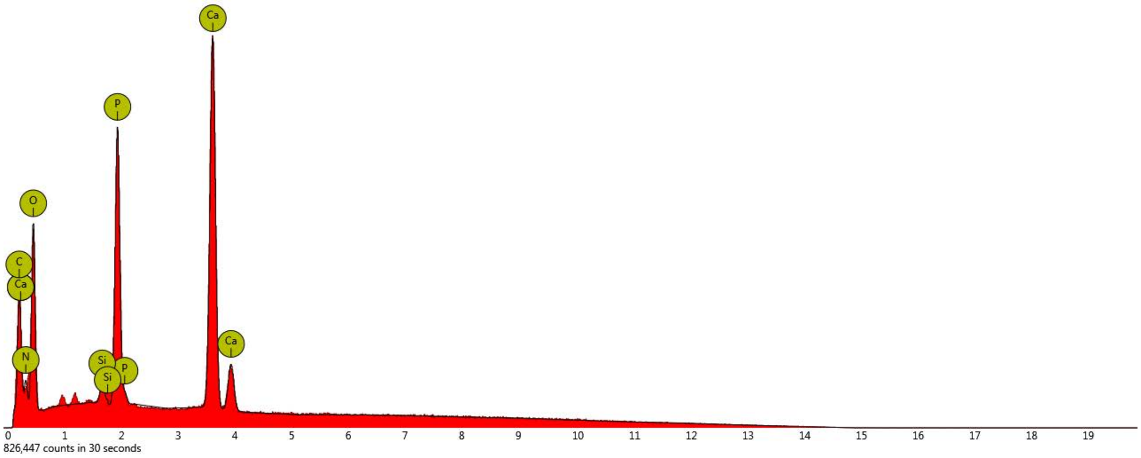

Disabled elements: B, Sr, Y

5. spot

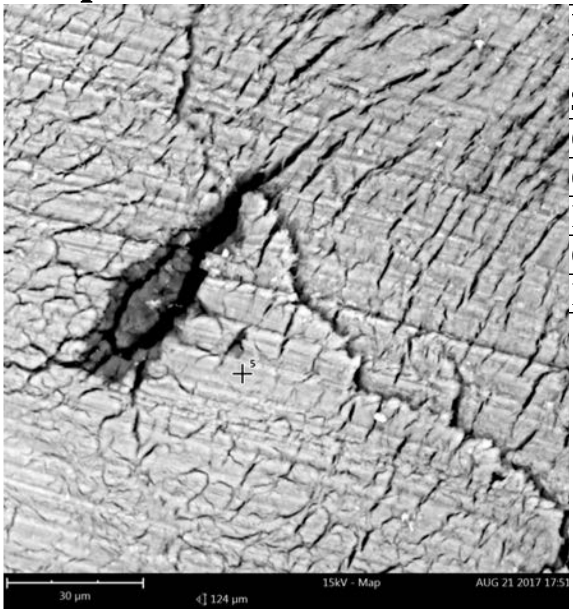

Element Symbol	Atomic Conc.	Weight Conc.	Oxide Symbol	Stoich. Conc.
Ca	14.73	30.87	Ca	22.06
O	33.23	27.80		
N	21.48	15.73	N	32.17
C	24.11	15.15	C	36.11
P	6.45	10.45	P	9.66

FOV: 124 µm, Mode: 15kV - Map, Detector: BSD Full, Time: AUG 21 2017 17:51

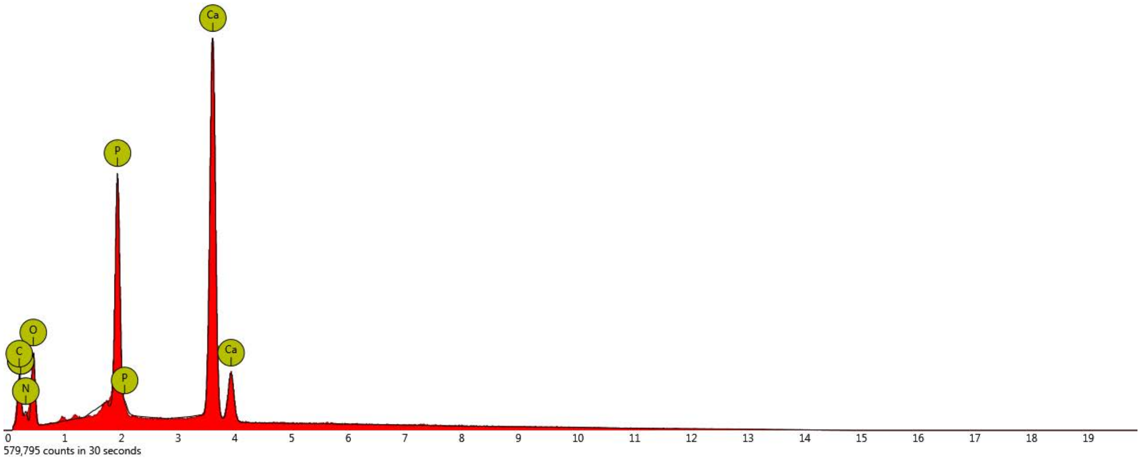

Disabled elements: B, Y

Osteocytes embedded in bone 02

1. map

Combined map

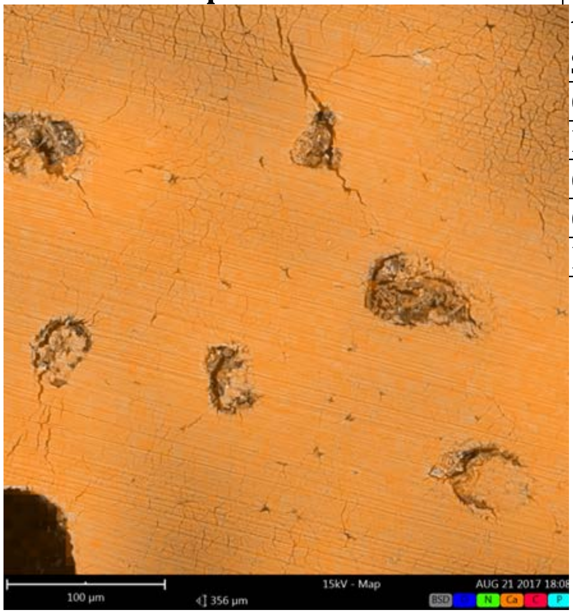

Element Symbol	Atomic Conc.	Weight Conc.	Oxide Symbol	Stoich. Conc.
O	40.64	37.54		
N	24.41	19.74	N	41.12
Ca	8.53	19.73	Ca	14.37
C	22.16	15.37	C	37.33
P	4.26	7.62	P	7.18

FOV: 356 μm, Mode: 15kV - Map, Detector: BSD Full, Time: AUG 21 2017 18:08

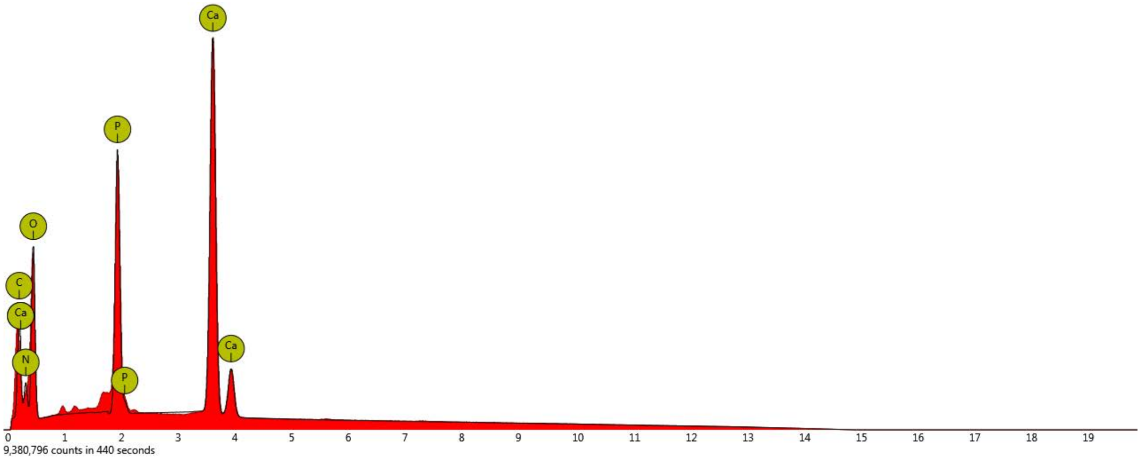

Disabled elements: B, Rb, Si, Sr, Te, Y

Cut out of map (resolution: 64x64 pixels)

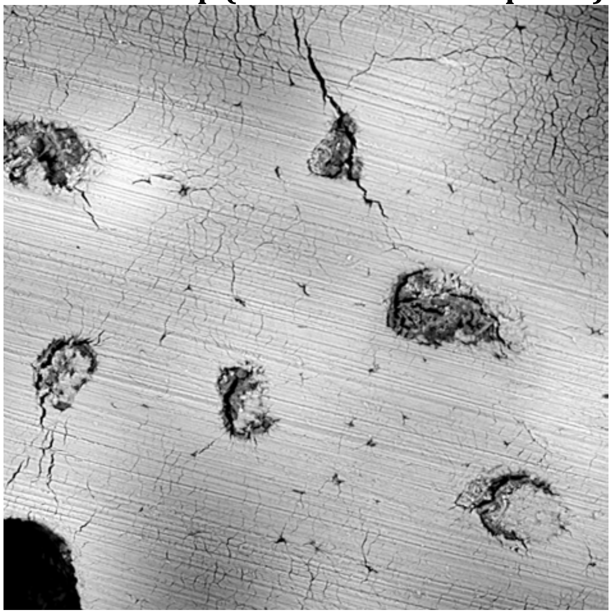

Oxygen

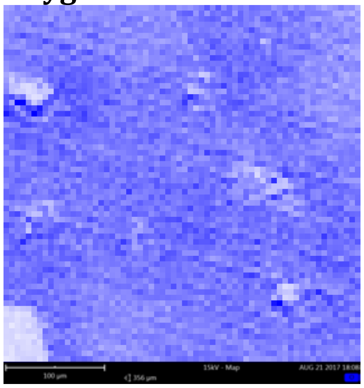

Nitrogen

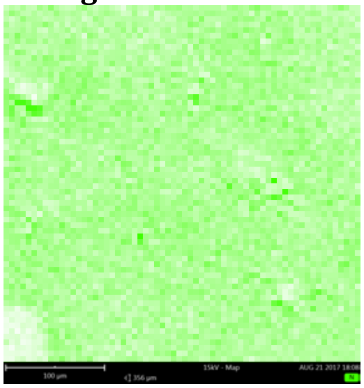

Calcium

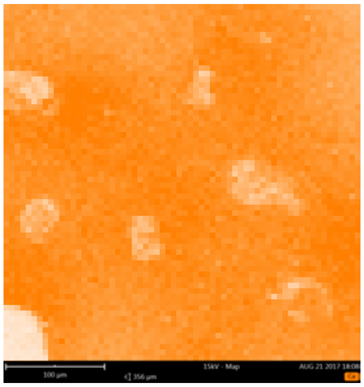

Carbon

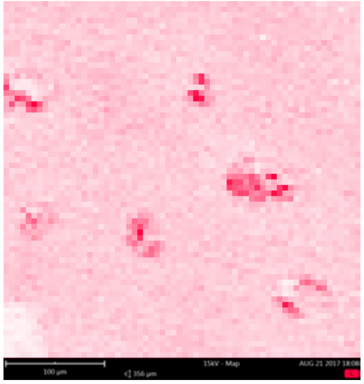

Phosphorus

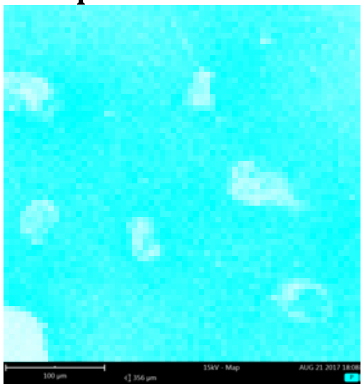

2. spot

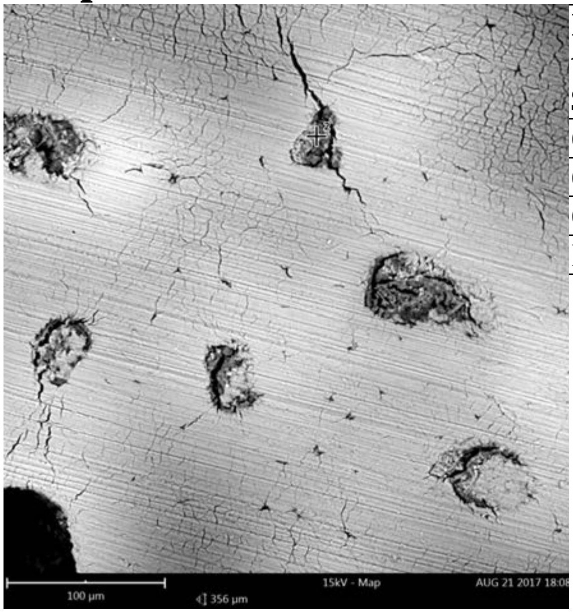

Element Symbol	Atomic Conc.	Weight Conc.	Oxide Symbol	Stoich. Conc.
C	60.17	47.07	C	87.85
O	31.51	32.85		
Ca	5.55	14.49	Ca	8.11
P	2.77	5.59	P	4.04

FOV: 356 µm, Mode: 15kV - Map, Detector: BSD Full, Time: AUG 21 2017 18:08

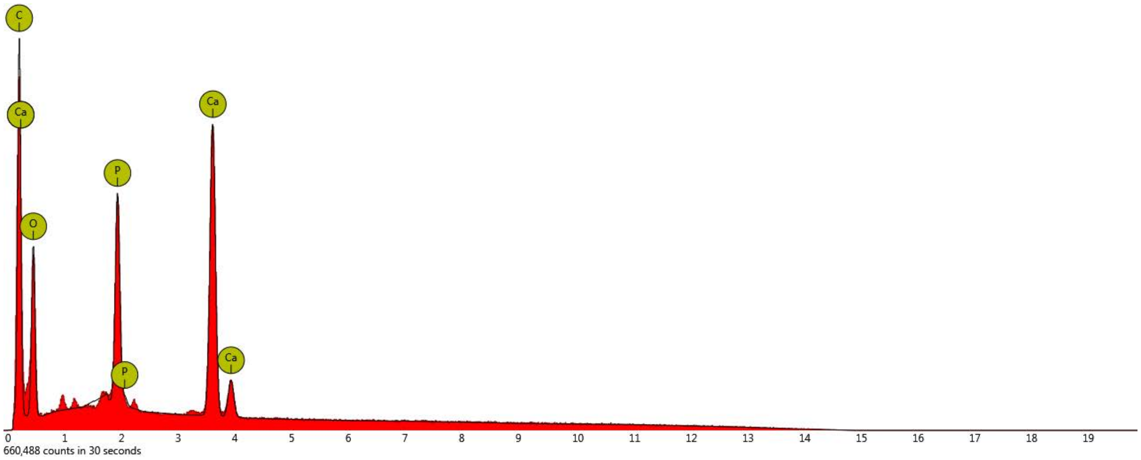

Disabled elements: B

3. spot

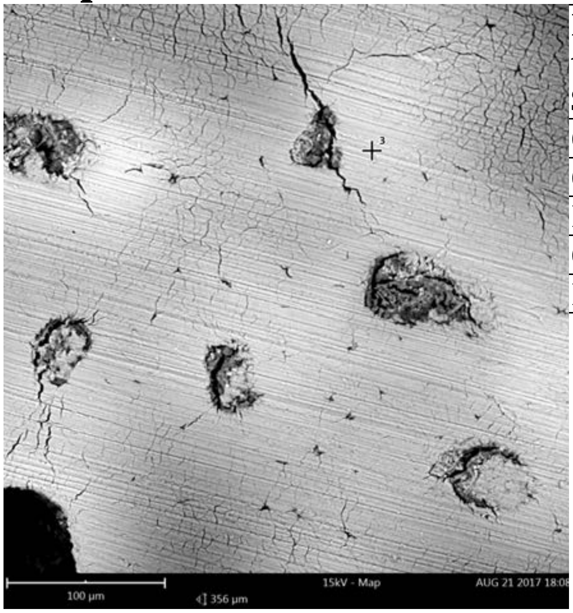

Element Symbol	Atomic Conc.	Weight Conc.	Oxide Symbol	Stoich. Conc.
Ca	16.21	33.21	Ca	24.20
O	33.02	27.01		
N	24.61	17.62	N	36.75
C	19.87	12.20	C	29.67
P	6.29	9.95	P	9.38

FOV: 356 µm, Mode: 15kV - Map, Detector: BSD Full, Time: AUG 21 2017 18:08

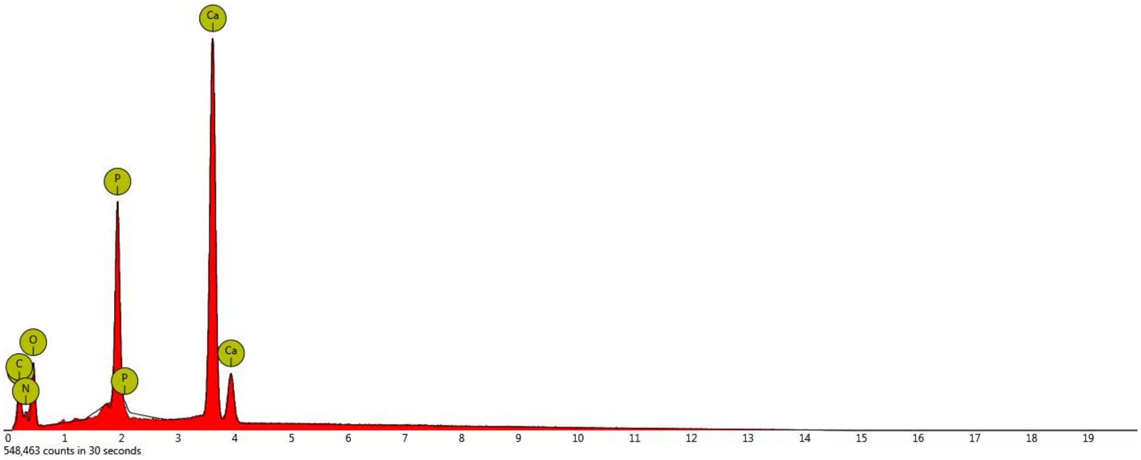

Disabled elements: B

4. spot

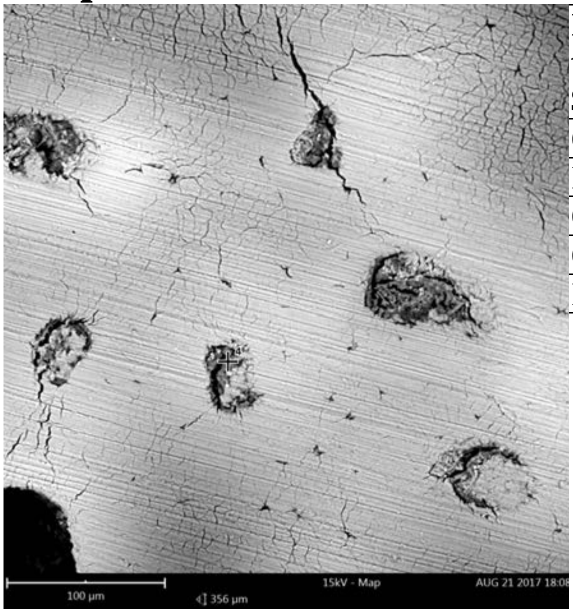

Element Symbol	Atomic Conc.	Weight Conc.	Oxide Symbol	Stoich. Conc.
C	49.83	42.39	C	63.52
N	25.63	25.43	N	32.67
O	21.55	24.42		
Ca	1.86	5.28	Ca	2.37
P	0.92	2.01	P	1.17

FOV: 356 µm, Mode: 15kV - Map, Detector: BSD Full, Time: AUG 21 2017 18:08

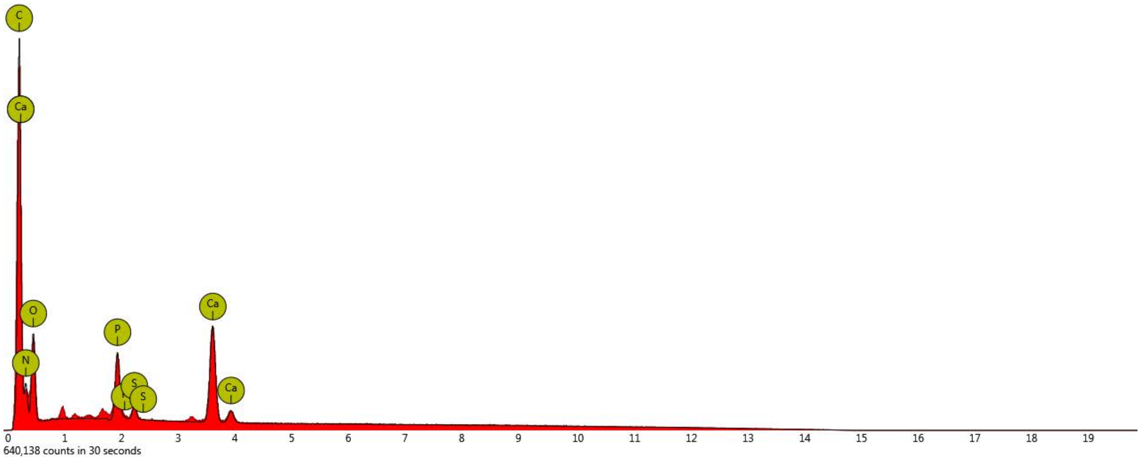

Disabled elements: B, In

5. spot

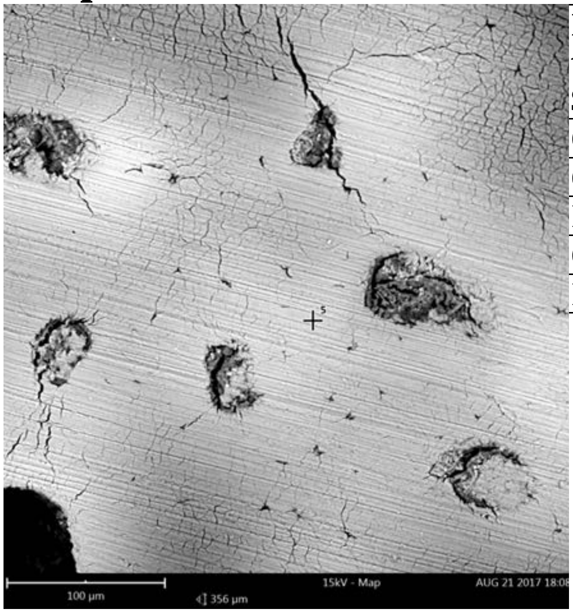

Element Symbol	Atomic Conc.	Weight Conc.	Oxide Symbol	Stoich. Conc.
O	37.07	31.00		
Ca	14.47	30.32	Ca	23.00
N	22.58	16.53	N	35.88
C	19.92	12.51	C	31.66
P	5.96	9.65	P	9.47

FOV: 356 μm, Mode: 15kV - Map, Detector: BSD Full, Time: AUG 21 2017 18:08

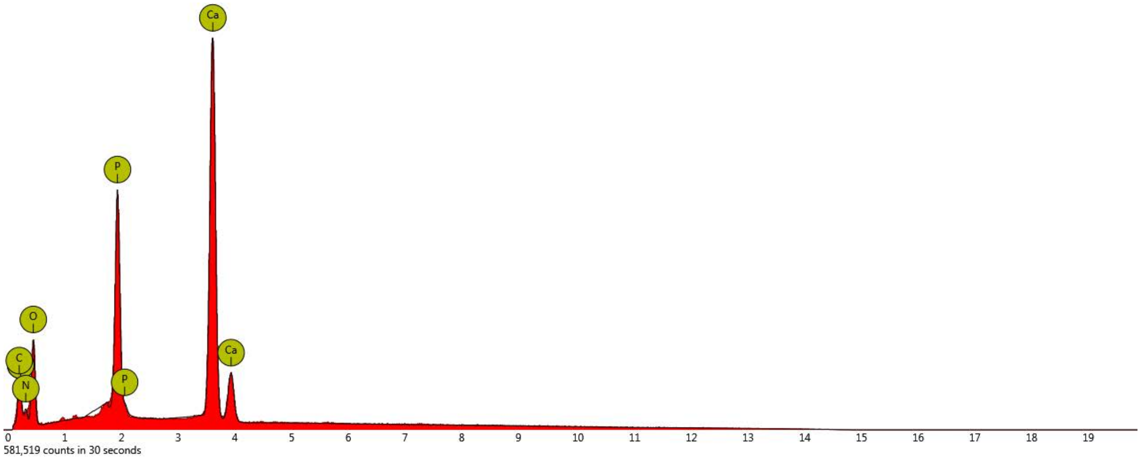

Disabled elements: B

Osteocytes embedded in bone 03

Combined map

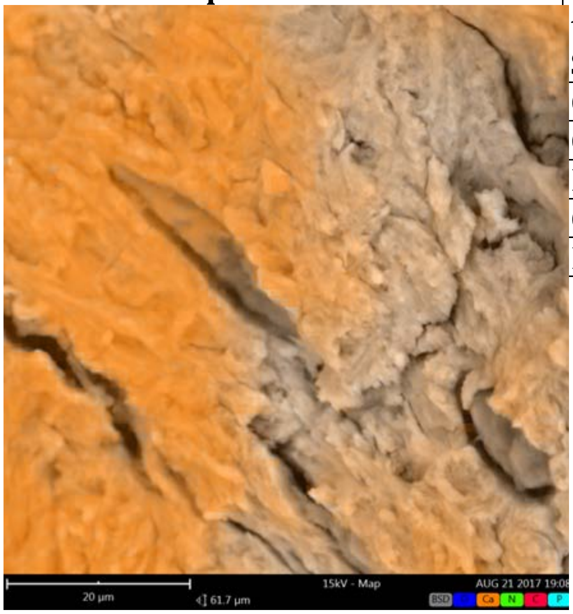

Element Symbol	Atomic Conc.	Weight Conc.	Oxide Symbol	Stoich. Conc.
O	40.93	35.15		
Ca	13.50	29.04	Ca	22.85
N	20.73	15.59	N	35.09
C	20.72	13.36	C	35.07
P	4.13	6.86	P	6.99

FOV: 61.7 µm, Mode: 15kV - Map, Detector: BSD Full, Time: AUG 21 2017 19:08

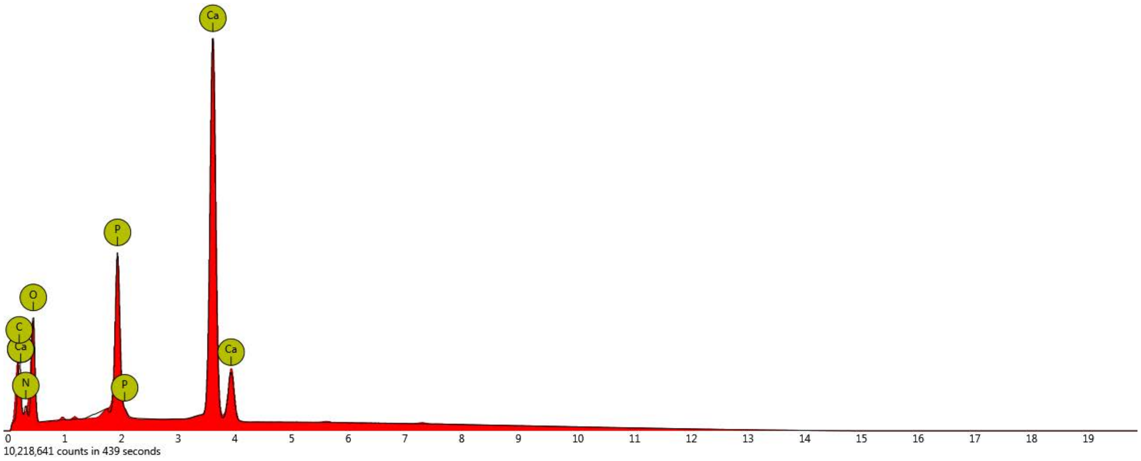

Disabled elements: B

Cut out of map (resolution: 64x64 pixels)

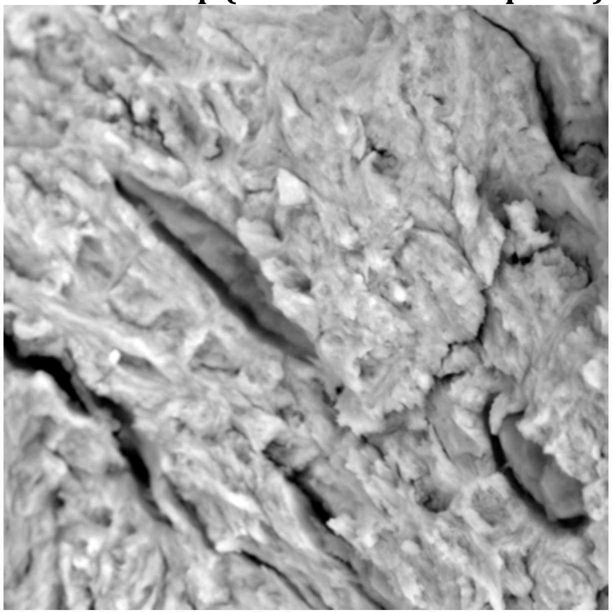

Oxygen

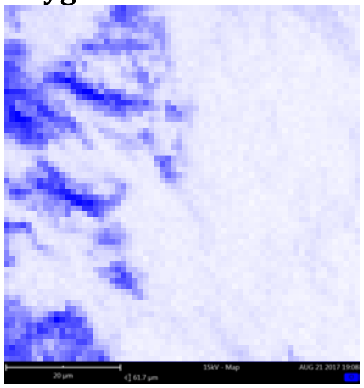

Calcium

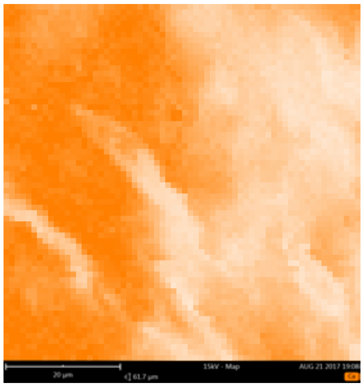

Nitrogen

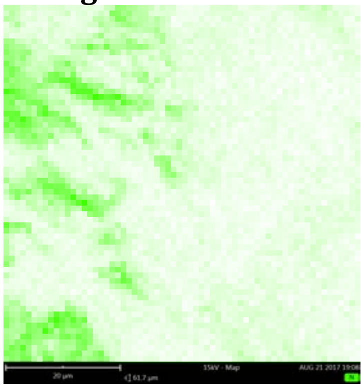

Carbon

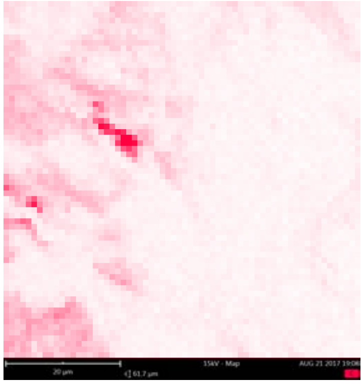

Phosphorus

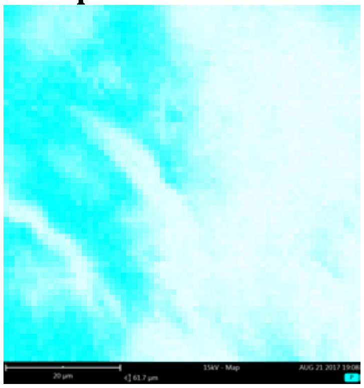

2. spot

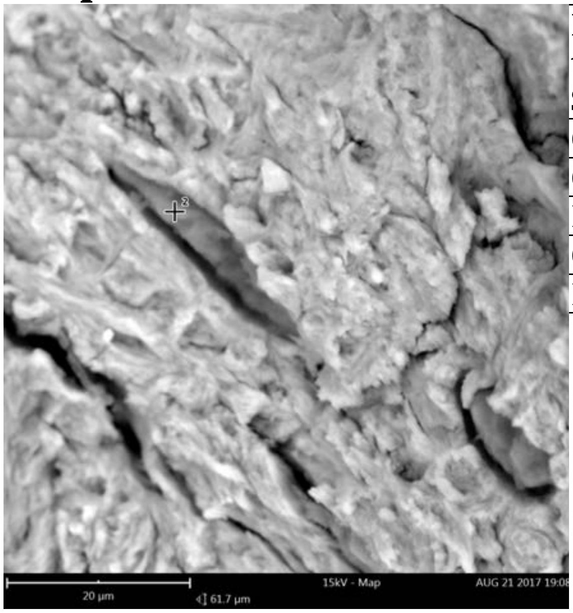

Element Symbol	Atomic Conc.	Weight Conc.	Oxide Symbol	Stoich. Conc.
C	57.02	46.79	C	75.77
O	24.74	27.05		
N	13.01	12.45	N	17.29
Ca	4.26	11.66	Ca	5.66
P	0.97	2.05	P	1.29

FOV: 61.7 µm, Mode: 15kV - Map, Detector: BSD Full, Time: AUG 21 2017 19:08

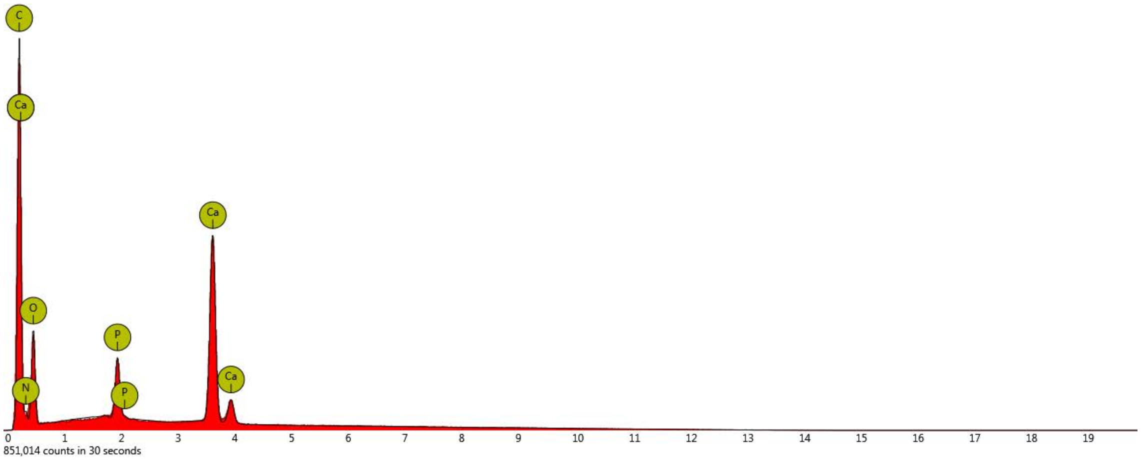

Disabled elements: B

3. spot

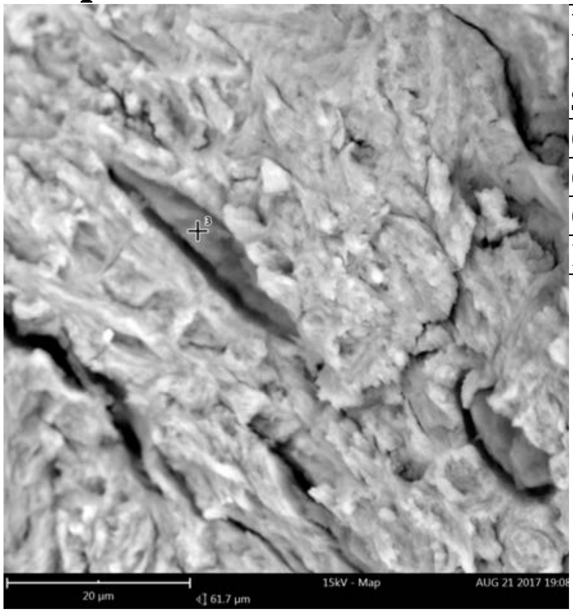

**Element Symbol**	**Atomic Conc.**	**Weight Conc.**	**Oxide Symbol**	**Stoich. Conc.**
C	55.48	39.00	C	79.16
Ca	12.22	28.65	Ca	17.43
O	29.92	28.02		
P	2.39	4.33	P	3.41

FOV: 61.7 µm, Mode: 15kV - Map, Detector: BSD Full, Time: AUG 21 2017 19:08

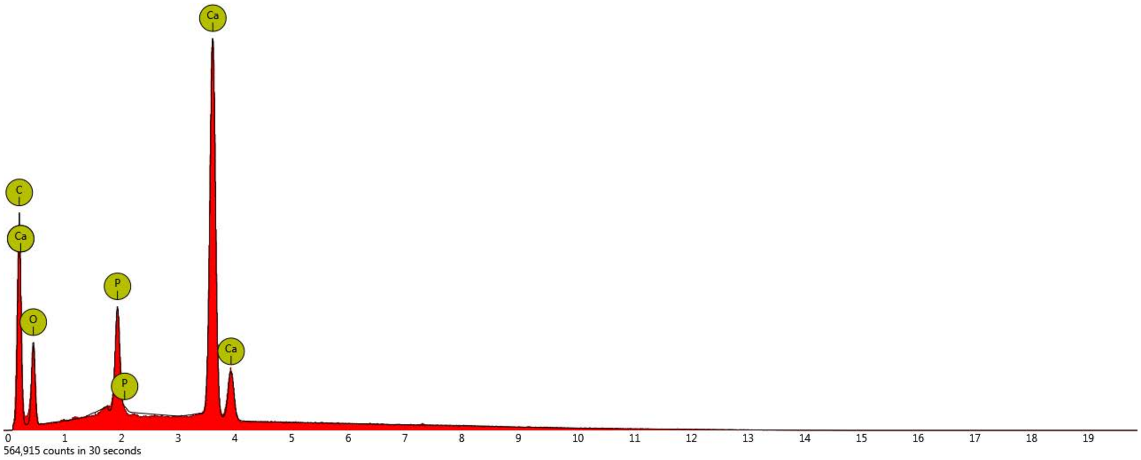

Disabled elements: B

4. spot

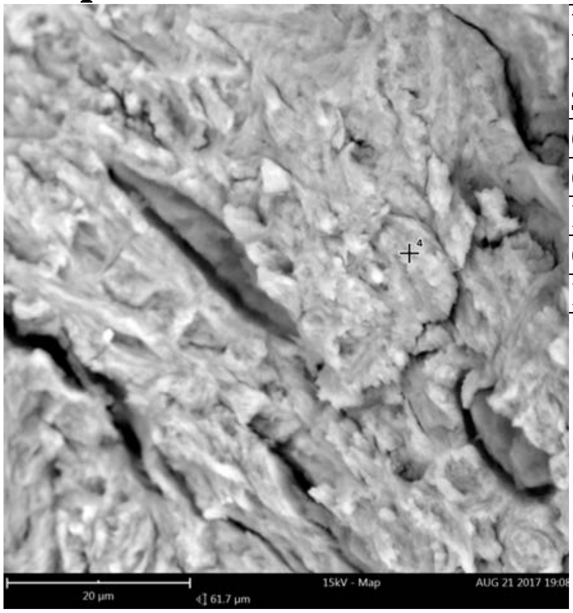

Element Symbol	Atomic Conc.	Weight Conc.	Oxide Symbol	Stoich. Conc.
Ca	19.56	39.47	Ca	30.72
O	36.34	29.28		
N	22.41	15.80	N	35.20
C	19.26	11.65	C	30.25
P	2.44	3.80	P	3.83

FOV: 61.7 µm, Mode: 15kV - Map, Detector: BSD Full, Time: AUG 21 2017 19:08

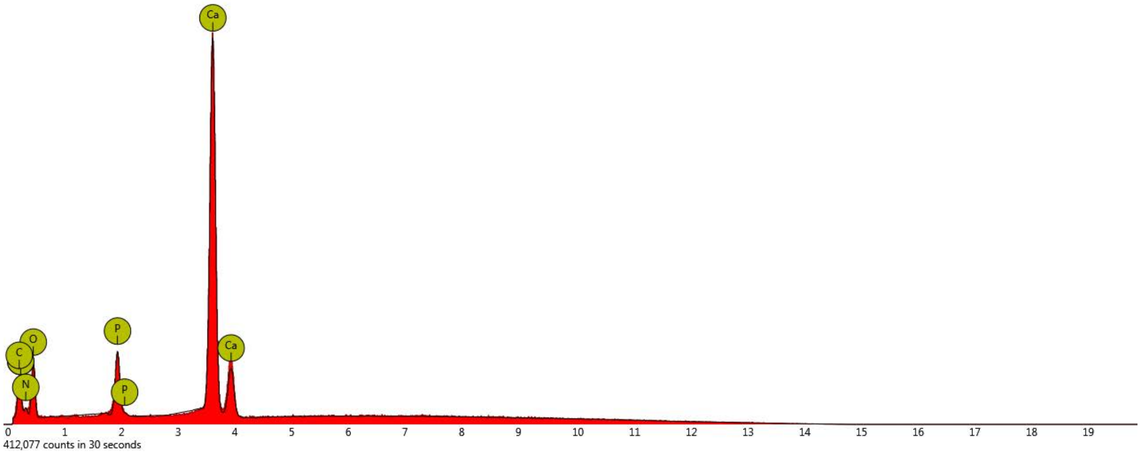

Disabled elements: B, I, Sn, Y

Blood vessel embedded in bone 01

1. spot

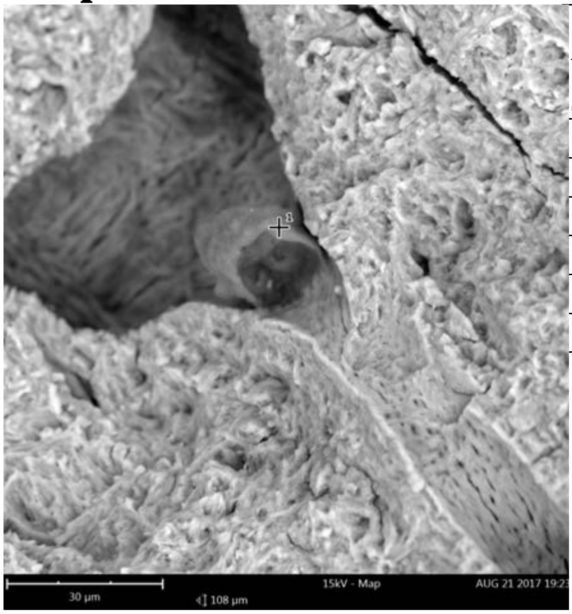

Element Symbol	Atomic Conc.	Weight Conc.	Oxide Symbol	Stoich. Conc.
C	44.06	38.03	C	56.77
N	32.29	32.50	N	41.60
O	22.38	25.73		
Ca	0.73	2.10	Ca	0.94
P	0.47	1.04	P	0.60
I	0.07	0.60	I	0.08

FOV: 108 µm, Mode: 15kV - Map, Detector: BSD Full, Time: AUG 21 2017 19:23

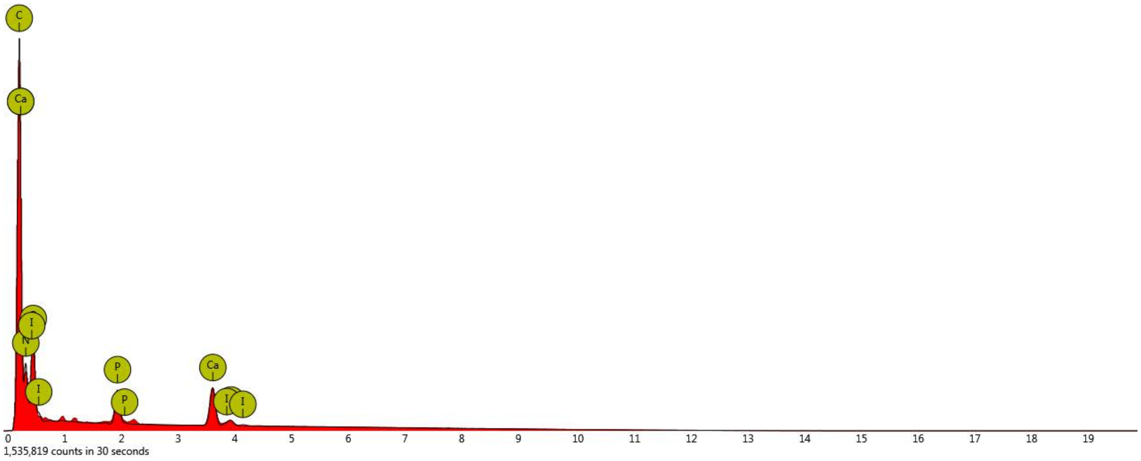

Disabled elements: B

2. spot

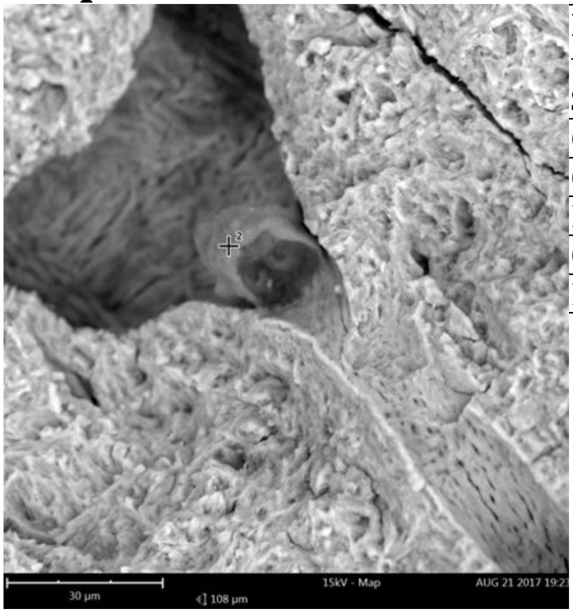

Element Symbol	Atomic Conc.	Weight Conc.	Oxide Symbol	Stoich. Conc.
C	52.00	45.06	C	67.02
O	22.41	25.86		
N	23.61	23.86	N	30.43
Ca	1.22	3.54	Ca	1.58
P	0.75	1.68	P	0.97

FOV: 108 µm, Mode: 15kV - Map, Detector: BSD Full, Time: AUG 21 2017 19:23

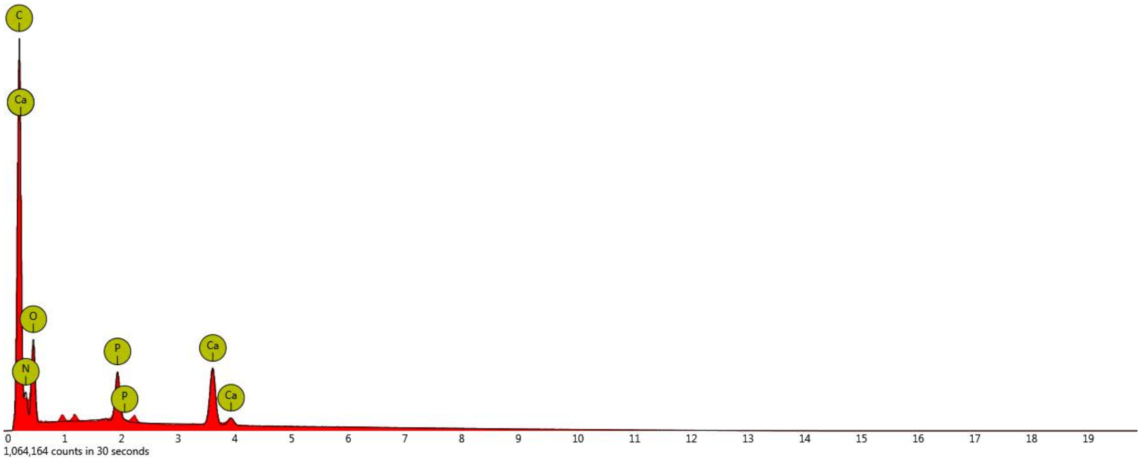

Disabled elements: B

3. spot

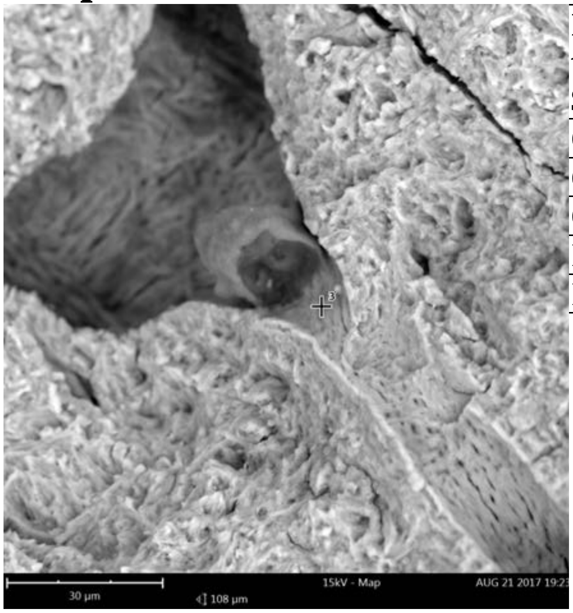

Element Symbol	Atomic Conc.	Weight Conc.	Oxide Symbol	Stoich. Conc.
O	39.77	33.99		
Ca	14.05	30.09	Ca	23.33
C	22.92	14.71	C	38.06
N	19.06	14.26	N	31.64
P	4.20	6.94	P	6.97

FOV: 108 µm, Mode: 15kV - Map, Detector: BSD Full, Time: AUG 21 2017 19:23

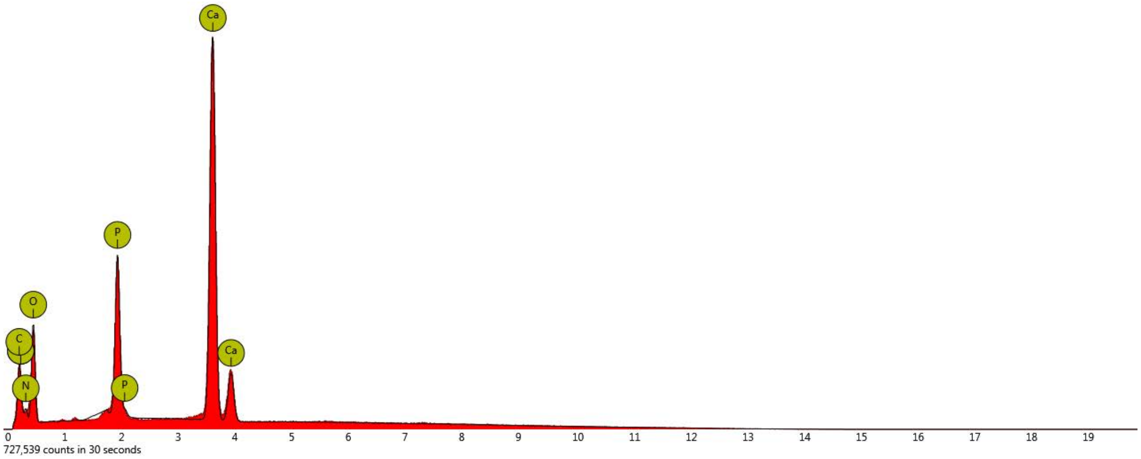

Disabled elements: Am, B, Sn, Y

4. spot

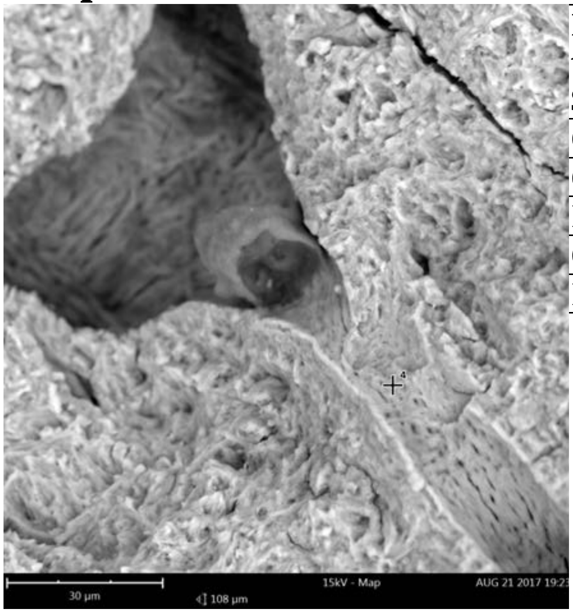

Element Symbol	Atomic Conc.	Weight Conc.	Oxide Symbol	Stoich. Conc.
O	55.31	51.47		
Ca	6.77	15.77	Ca	15.14
N	16.37	13.34	N	36.64
C	17.60	12.29	C	39.38
P	3.95	7.12	P	8.84

FOV: 108 µm, Mode: 15kV - Map, Detector: BSD Full, Time: AUG 21 2017 19:23

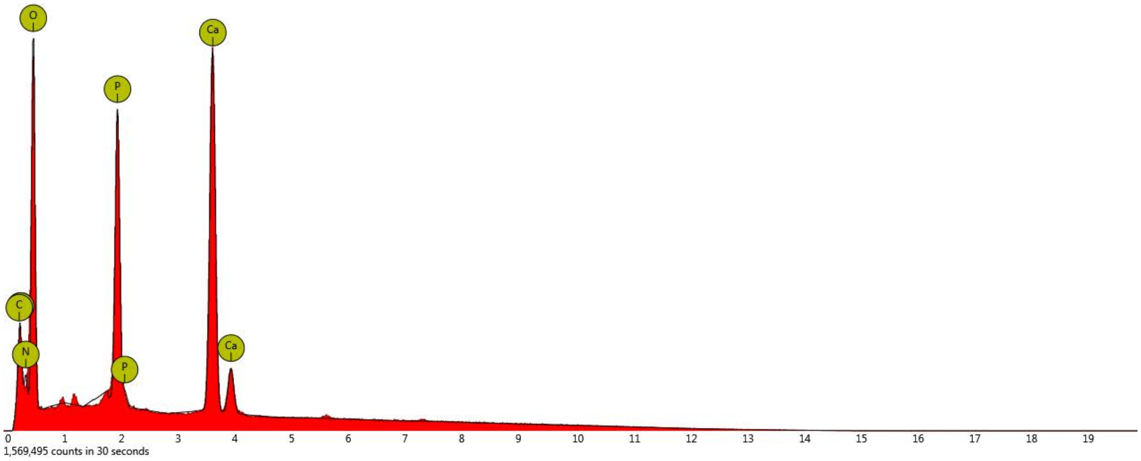

Disabled elements: B, Y

5. spot

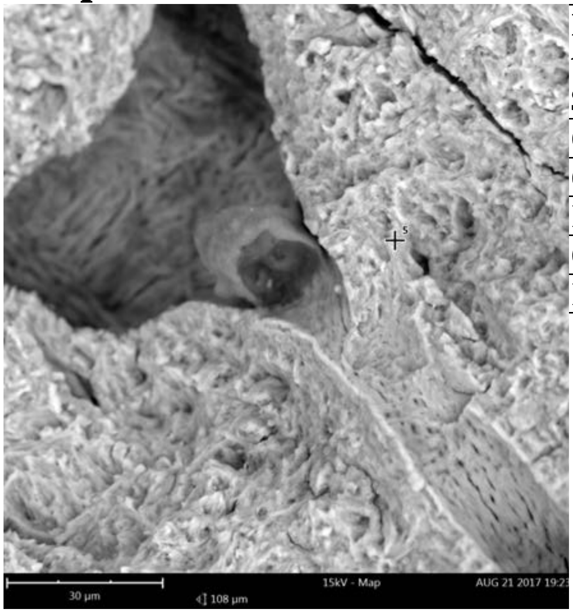

Element Symbol	Atomic Conc.	Weight Conc.	Oxide Symbol	Stoich. Conc.
O	52.68	47.08		
Ca	8.76	19.62	Ca	18.52
N	15.56	12.17	N	32.88
C	17.51	11.75	C	37.00
P	5.15	8.91	P	10.88

FOV: 108 µm, Mode: 15kV - Map, Detector: BSD Full, Time: AUG 21 2017 19:23

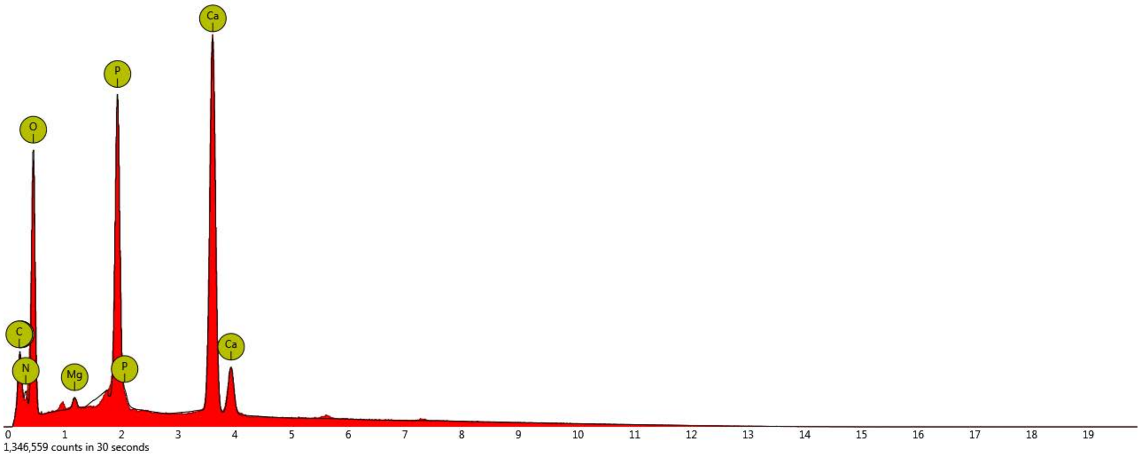

Disabled elements: B, Y

Blood vessel embedded in bone 02

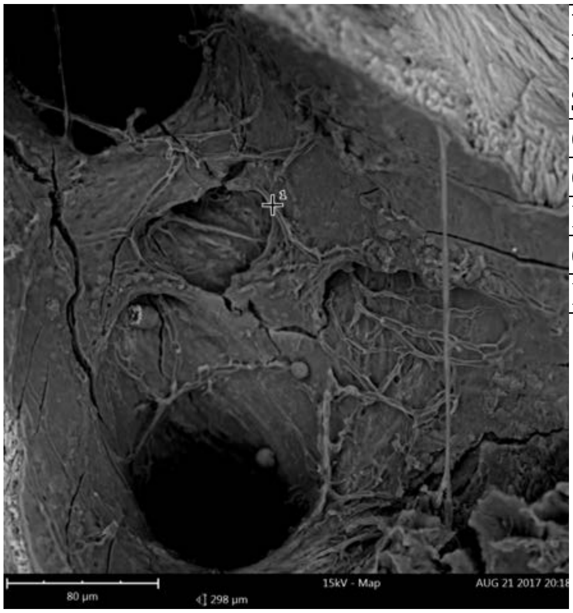

Element Symbol	Atomic Conc.	Weight Conc.	Oxide Symbol	Stoich. Conc.
O	35.44	38.50		
C	35.75	29.16	C	55.38
N	25.67	24.42	N	39.77
Ca	2.15	5.85	Ca	3.33
P	0.98	2.07	P	1.53

FOV: 298 µm, Mode: 15kV - Map, Detector: BSD Full, Time: AUG 21 2017 20:18

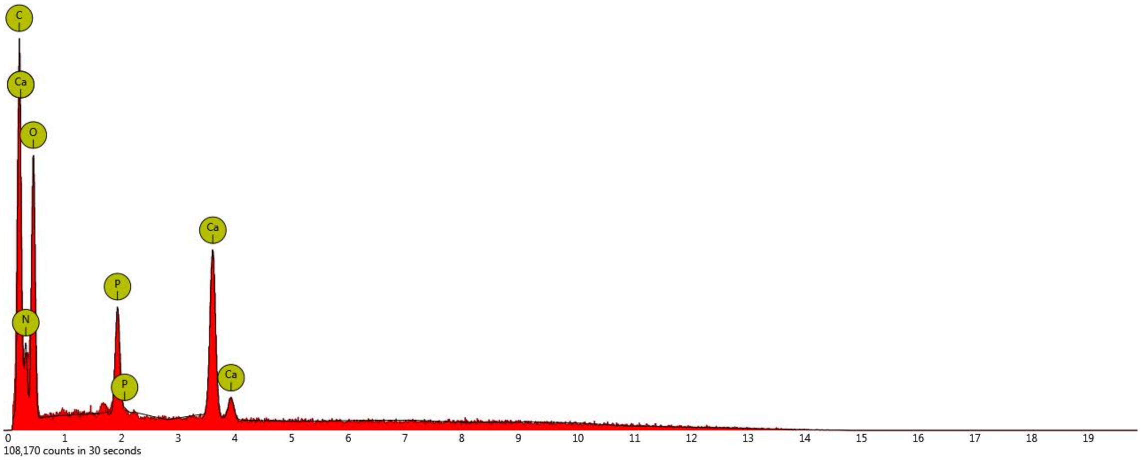

Disabled elements: B

2. spot

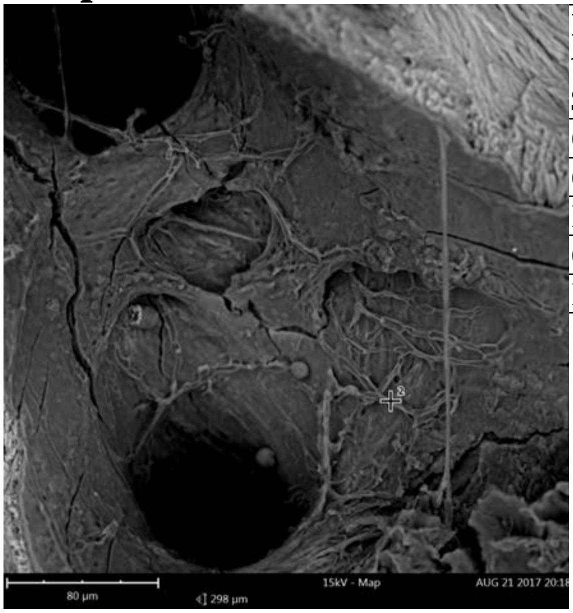

Element Symbol	Atomic Conc.	Weight Conc.	Oxide Symbol	Stoich. Conc.
O	32.90	36.08		
C	35.69	29.39	C	53.19
N	28.68	27.54	N	42.74
Ca	1.91	5.25	Ca	2.85
P	0.82	1.74	P	1.22

FOV: 298 µm, Mode: 15kV - Map, Detector: BSD Full, Time: AUG 21 2017 20:18

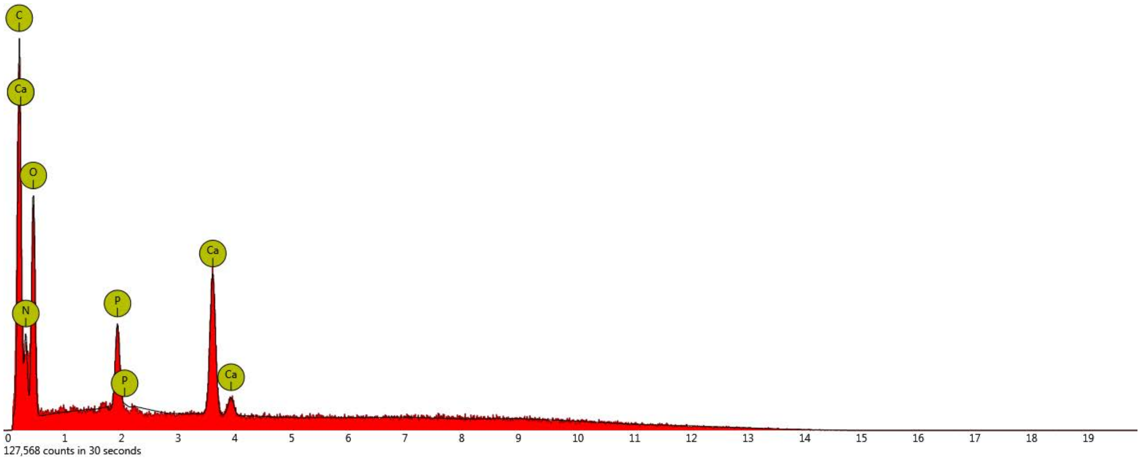

Disabled elements: B

3. spot

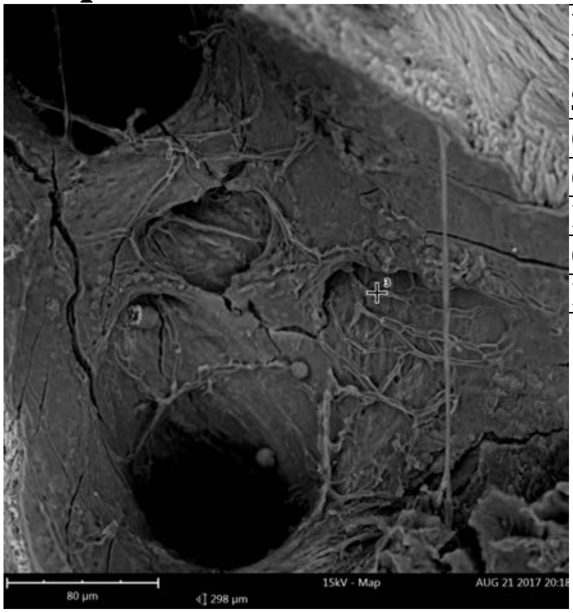

**Element Symbol**	**Atomic Conc.**	**Weight Conc.**	**Oxide Symbol**	**Stoich. Conc.**
O	33.93	37.21		
C	36.64	30.16	C	55.45
N	26.70	25.63	N	40.41
Ca	1.90	5.23	Ca	2.88
P	0.84	1.78	P	1.27

FOV: 298 µm, Mode: 15kV - Map, Detector: BSD Full, Time: AUG 21 2017 20:18

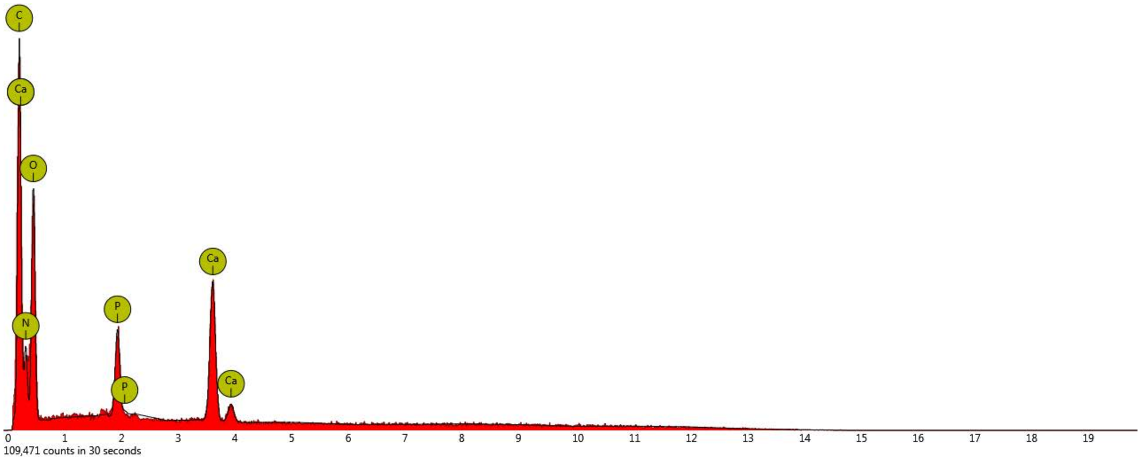

Disabled elements: B

4. spot

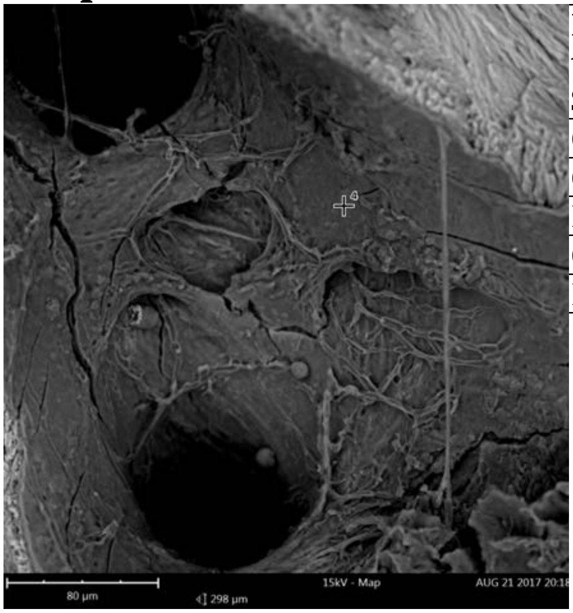

Element Symbol	Atomic Conc.	Weight Conc.	Oxide Symbol	Stoich. Conc.
O	35.66	38.36		
C	33.80	27.30	C	52.54
N	26.98	25.41	N	41.92
Ca	2.45	6.60	Ca	3.81
P	1.12	2.32	P	1.73

FOV: 298 μm, Mode: 15kV - Map, Detector: BSD Full, Time: AUG 21 2017 20:18

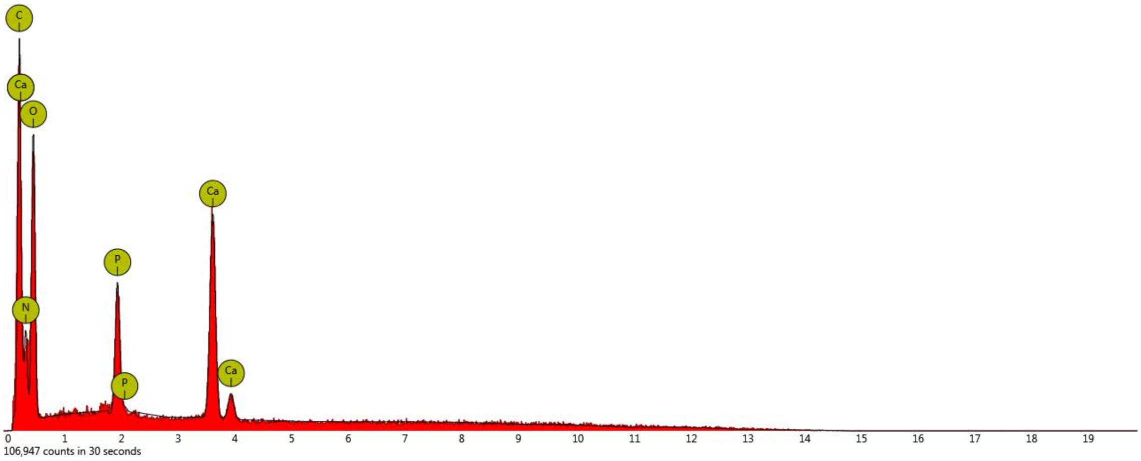

Disabled elements: B

5. spot

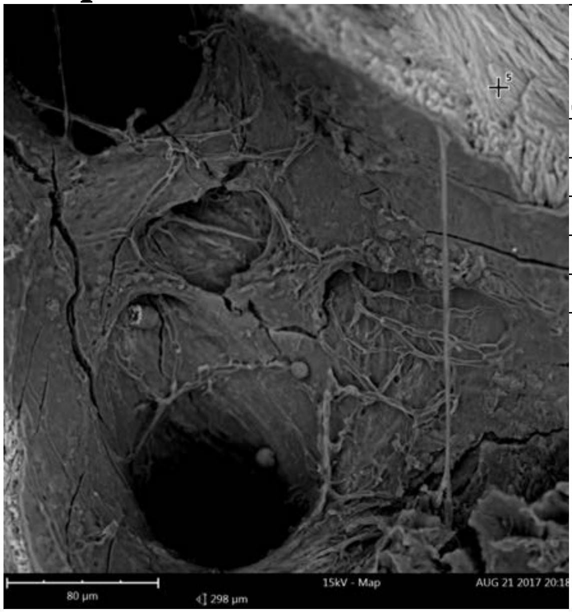

Element Symbol	Atomic Conc.	Weight Conc.	Oxide Symbol	Stoich. Conc.
O	45.49	44.41		
C	24.80	18.17	C	45.50
N	21.09	18.03	N	38.70
Ca	5.62	13.73	Ca	10.30
P	2.99	5.66	P	5.49

FOV: 298 µm, Mode: 15kV - Map, Detector: BSD Full, Time: AUG 21 2017 20:18

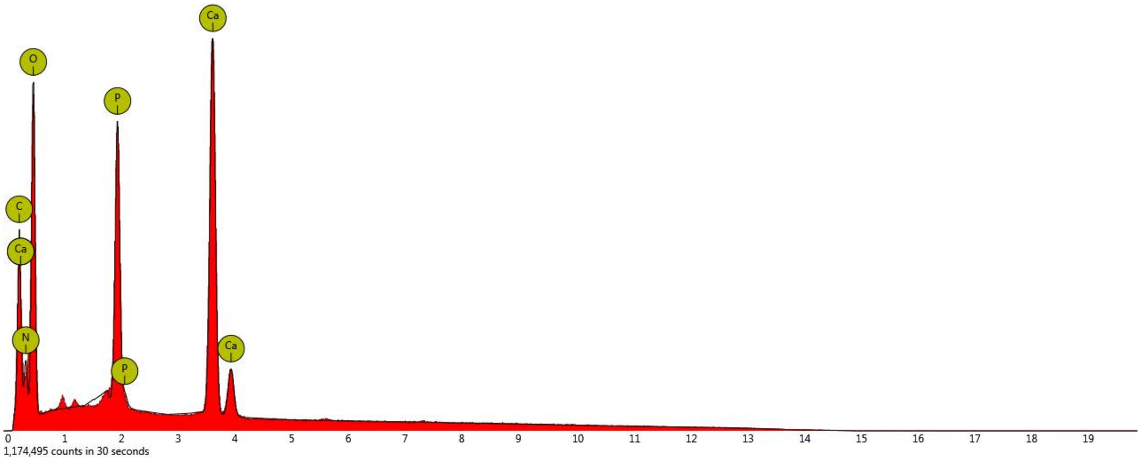

Disabled elements: B

Blood vessel embedded in bone 03

1. map

Combined map

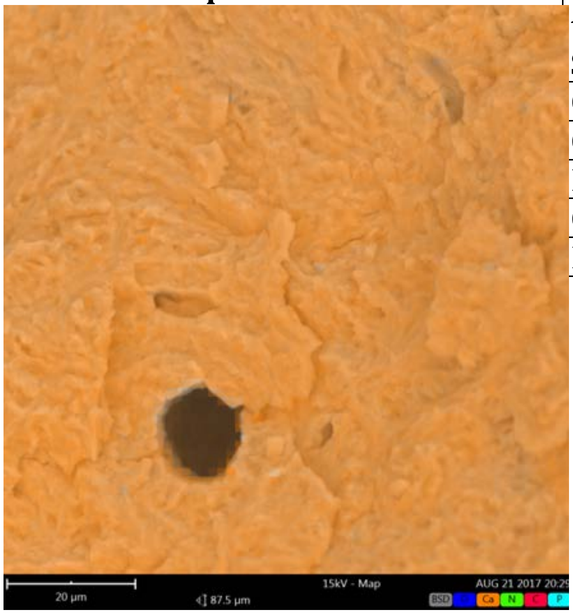

FOV: 87.5 µm, Mode: 15kV - Map, Detector: BSD Full, Time: AUG 21 2017 20:29

Element Symbol	Atomic Conc.	Weight Conc.	Oxide Symbol	Stoich. Conc.
O	51.55	45.97		
Ca	9.24	20.64	Ca	19.08
N	19.35	15.11	N	39.94
C	15.13	10.13	C	31.24
P	4.72	8.15	P	9.75

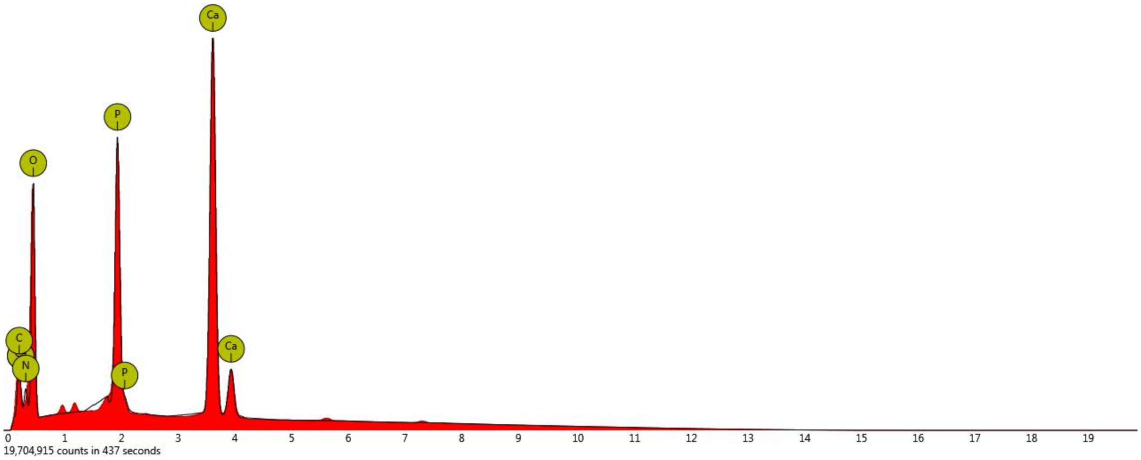

Disabled elements: B

Cut out of map (resolution: 64x64 pixels)

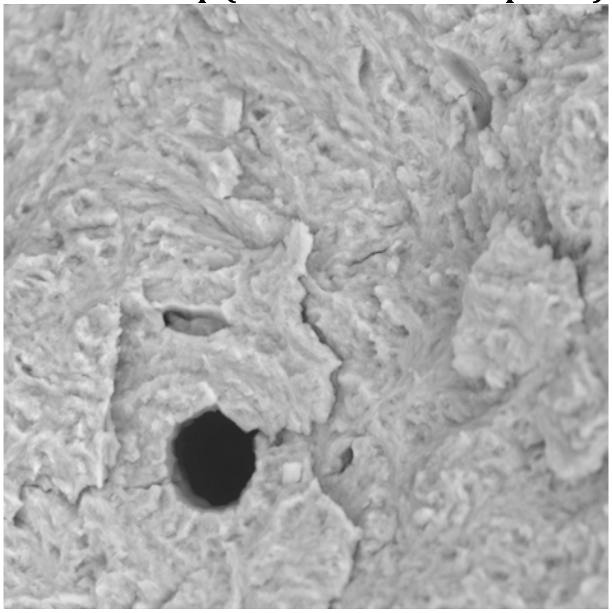

Oxygen

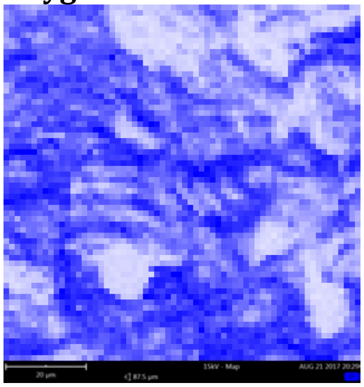

Calcium

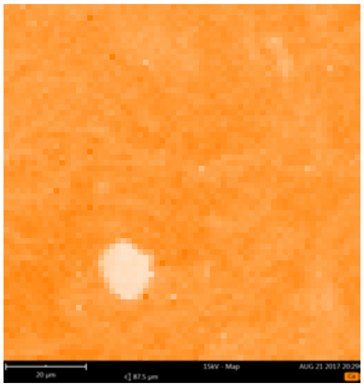

Nitrogen

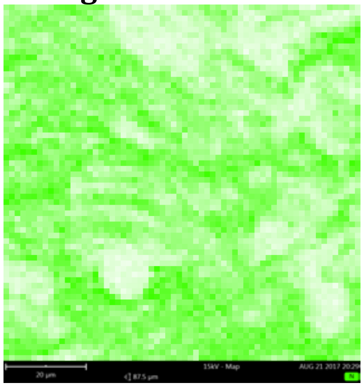

Carbon

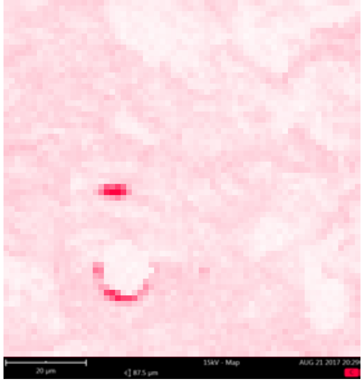

Phosphorus

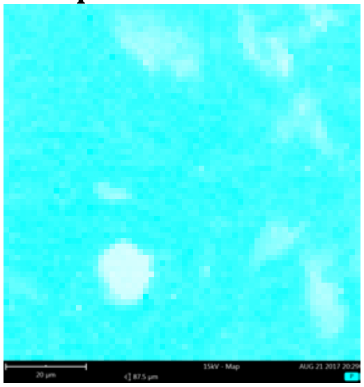

2. spot

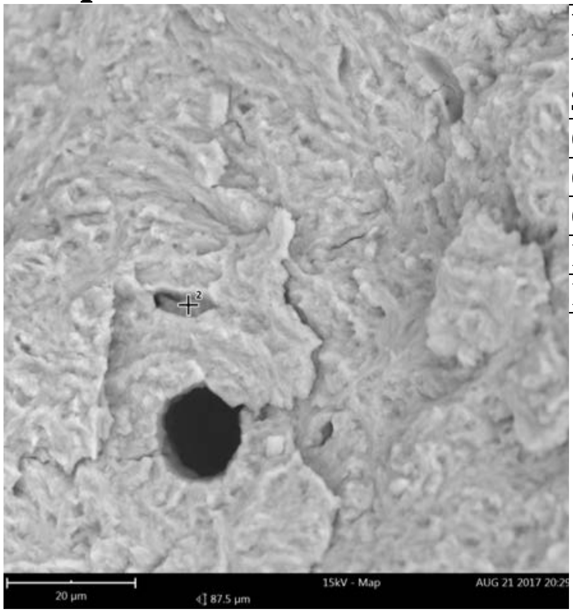

Element Symbol	Atomic Conc.	Weight Conc.	Oxide Symbol	Stoich. Conc.
C	51.83	41.19	C	72.88
O	28.88	30.58		
Ca	4.80	12.73	Ca	6.75
N	12.64	11.71	N	17.77
P	1.85	3.78	P	2.59

FOV: 87.5 µm, Mode: 15kV - Map, Detector: BSD Full, Time: AUG 21 2017 20:29

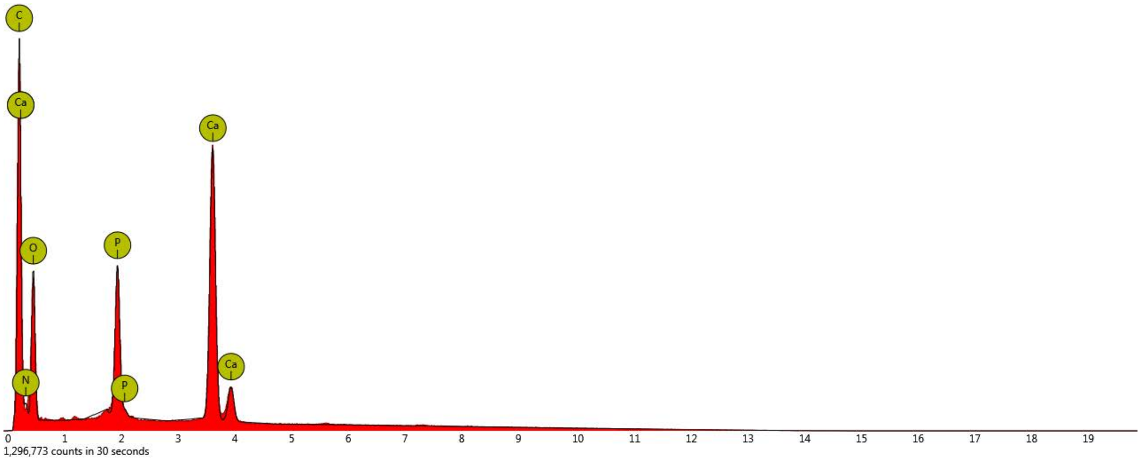

Disabled elements: B, I, Sn

3. spot

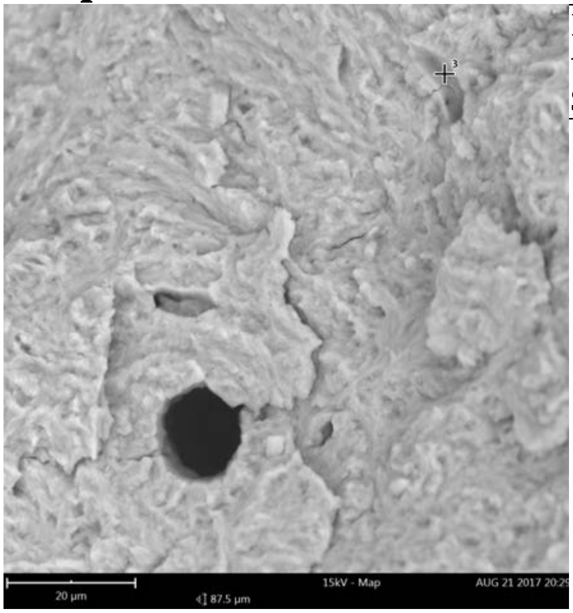

Element Symbol	Atomic Conc.	Weight Conc.	Oxide Symbol	Stoich. Conc.

FOV: 87.5 µm, Mode: 15kV - Map, Detector: BSD Full, Time: AUG 21 2017 20:29

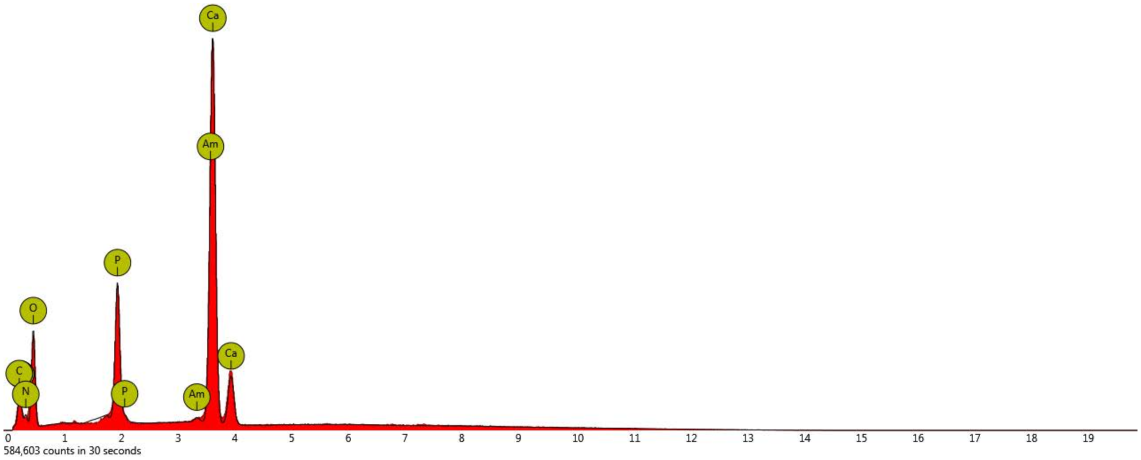

Disabled elements: B, Sn, Y

4. spot

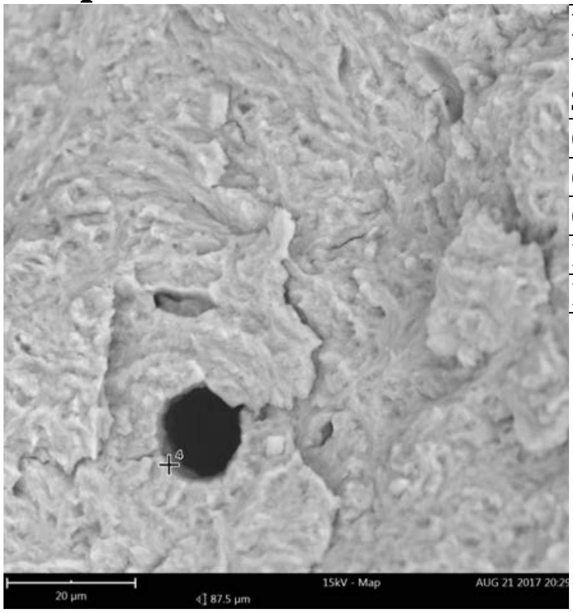

Element Symbol	Atomic Conc.	Weight Conc.	Oxide Symbol	Stoich. Conc.
O	49.62	45.77		
Ca	8.09	18.69	Ca	16.06
C	21.53	14.91	C	42.73
N	16.82	13.58	N	33.39
P	3.94	7.04	P	7.83

FOV: 87.5 µm, Mode: 15kV - Map, Detector: BSD Full, Time: AUG 21 2017 20:29

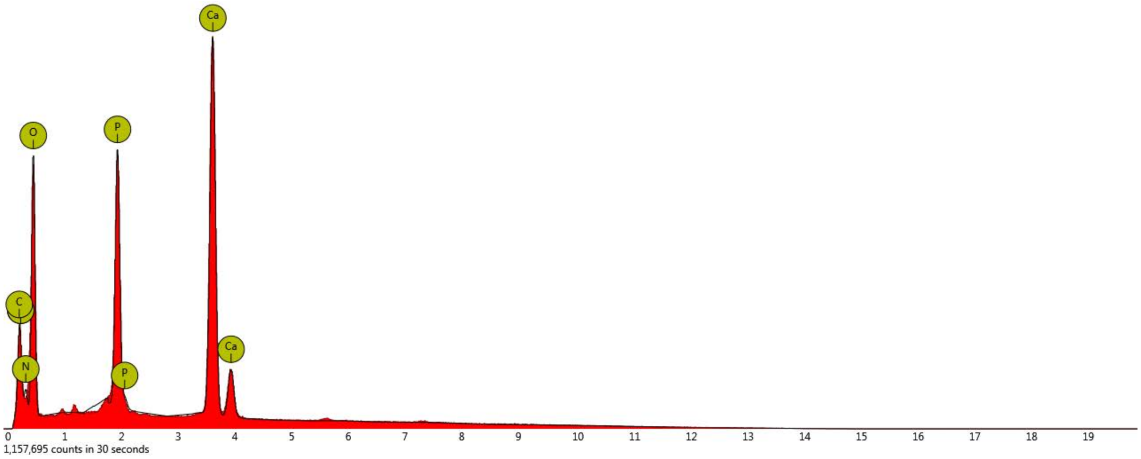

Disabled elements: B

5. spot

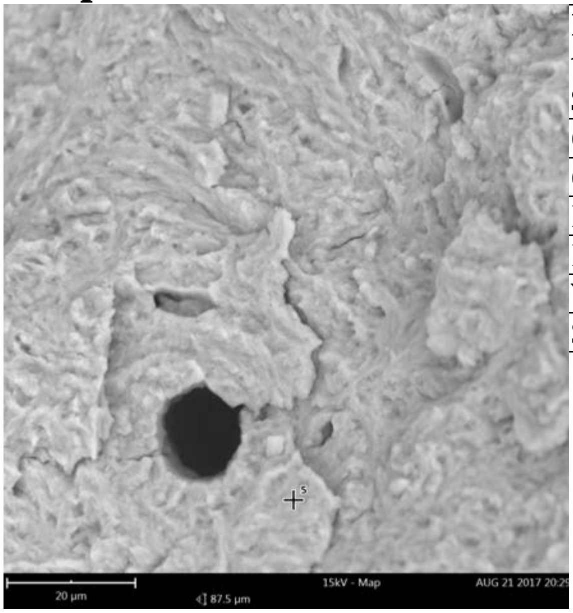

Element Symbol	Atomic Conc.	Weight Conc.	Oxide Symbol	Stoich. Conc.
O	66.54	54.01		
Ca	10.65	21.65	Ca	31.82
N	15.74	11.19	N	47.04
P	6.37	10.01	P	19.04
Y	0.46	2.08	Y	1.38
Sr	0.24	1.06	Sr	0.71

FOV: 87.5 µm, Mode: 15kV - Map, Detector: BSD Full, Time: AUG 21 2017 20:29

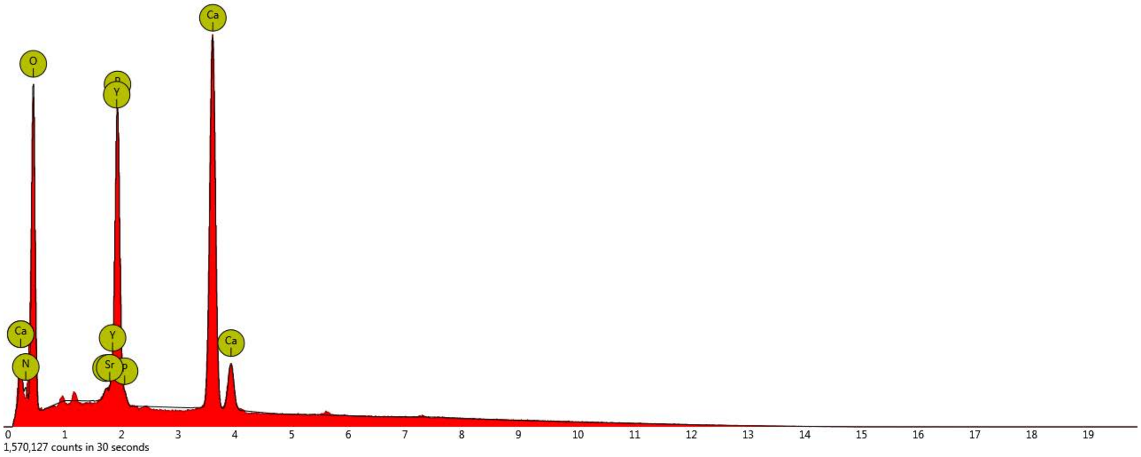

Disabled elements: B

Lepidochelys olivacea (uncatalogued specimen)

Lacuna after osteocyte is released 01

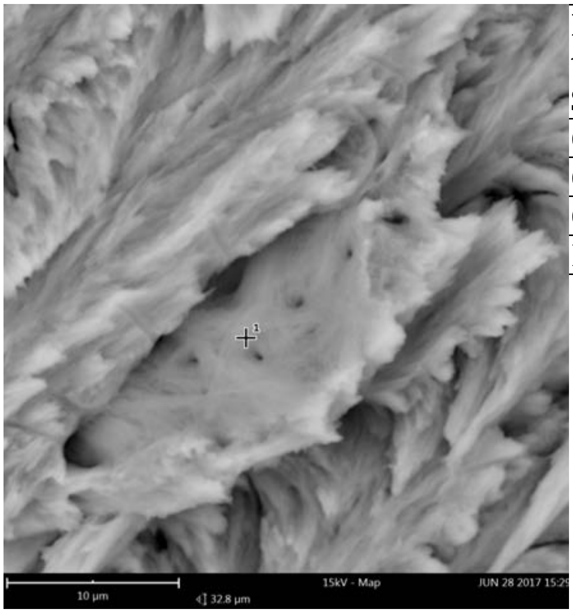

Element Symbol	Atomic Conc.	Weight Conc.	Oxide Symbol	Stoich. Conc.
Ca	25.86	45.98	Ca	40.68
O	36.43	25.86		
C	28.12	14.98	C	44.23
P	9.59	13.18	P	15.09

FOV: 32.8 µm, Mode: 15kV - Map, Detector: BSD Full, Time: JUN 28 2017 15:29

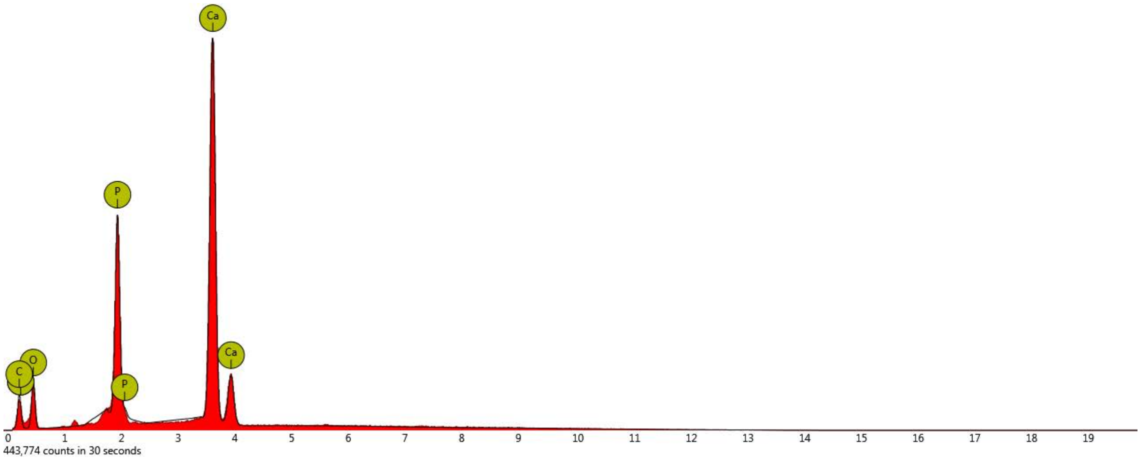

Disabled elements: B

2. spot

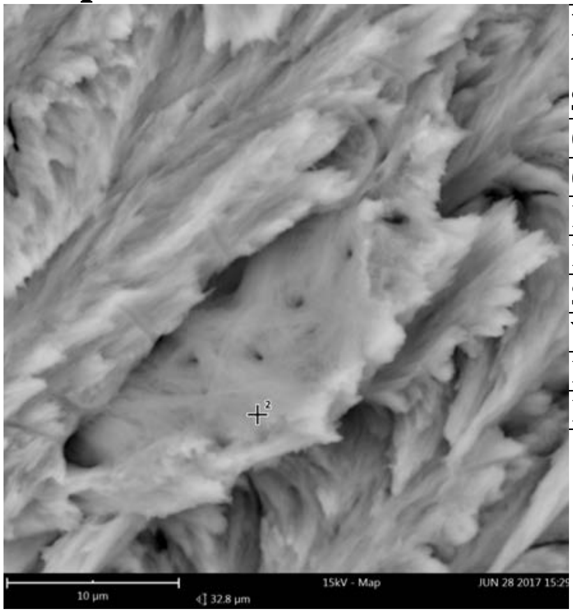

Element Symbol	Atomic Conc.	Weight Conc.	Oxide Symbol	Stoich. Conc.
O	56.31	41.17		
Ca	14.66	26.85	Ca	33.55
P	10.34	14.63	P	23.66
N	15.99	10.24	N	36.60
Sr	0.68	2.72	Sr	1.55
Y	0.52	2.12	Y	1.19
Mg	1.29	1.43	Mg	2.94
Rb	0.22	0.86	Rb	0.50

FOV: 32.8 µm, Mode: 15kV - Map, Detector: BSD Full, Time: JUN 28 2017 15:29

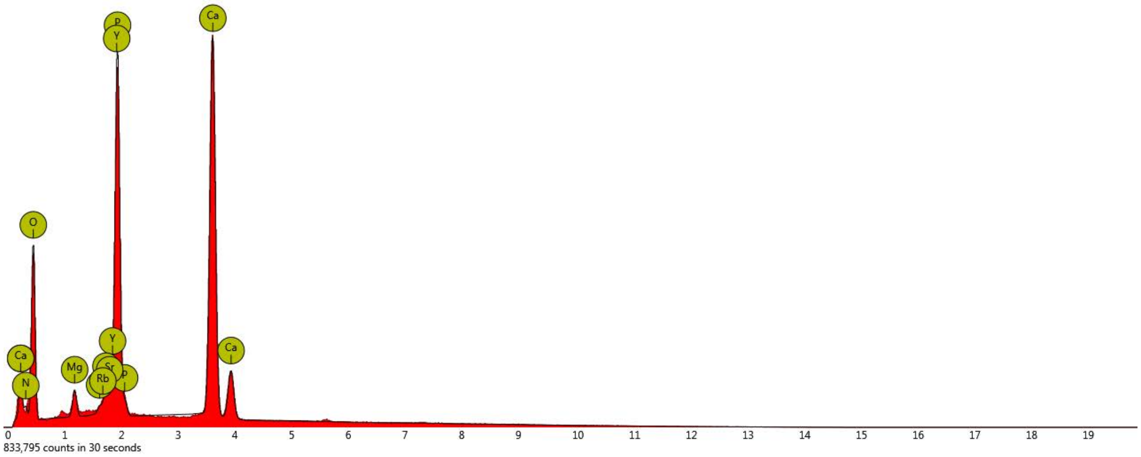

Disabled elements: B

3. spot

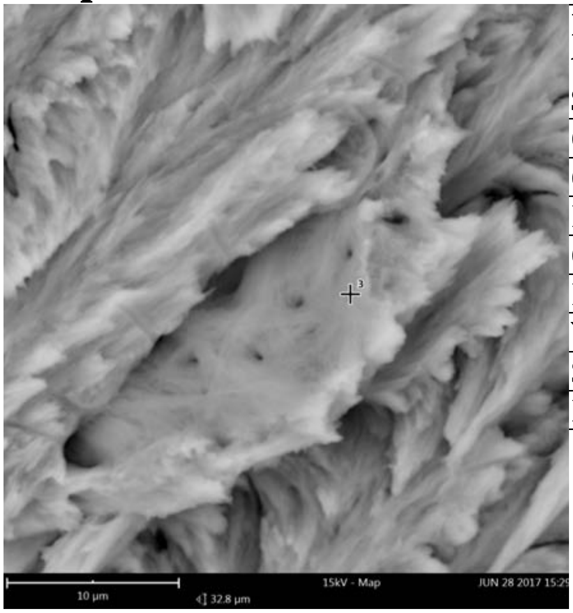

Element Symbol	Atomic Conc.	Weight Conc.	Oxide Symbol	Stoich. Conc.
O	52.34	44.01		
Ca	10.14	21.35	Ca	21.26
N	13.96	10.28	N	29.29
C	16.15	10.20	C	33.88
P	6.03	9.82	P	12.66
Y	0.40	1.87	Y	0.84
Sr	0.36	1.68	Sr	0.76
Mg	0.62	0.79	Mg	1.30

FOV: 32.8 µm, Mode: 15kV - Map, Detector: BSD Full, Time: JUN 28 2017 15:29

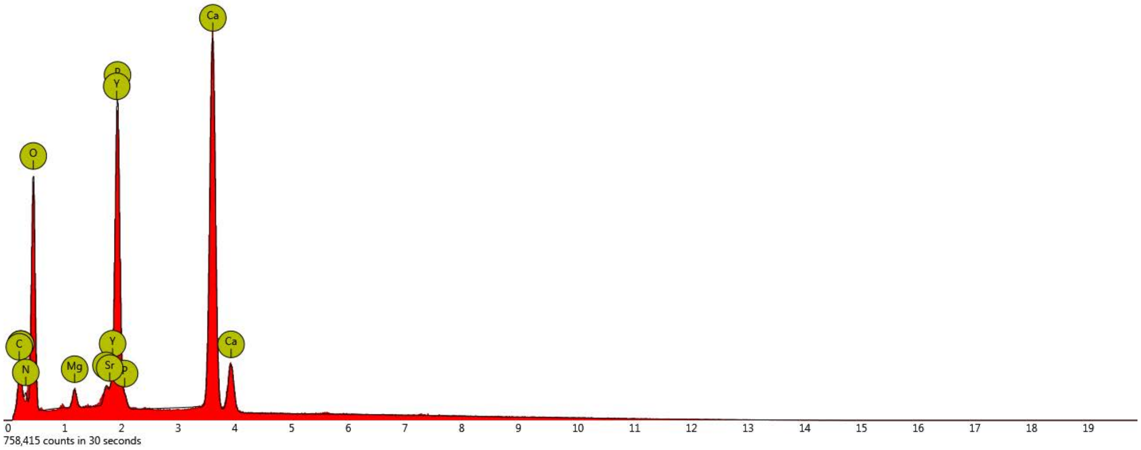

Disabled elements: B

4. spot

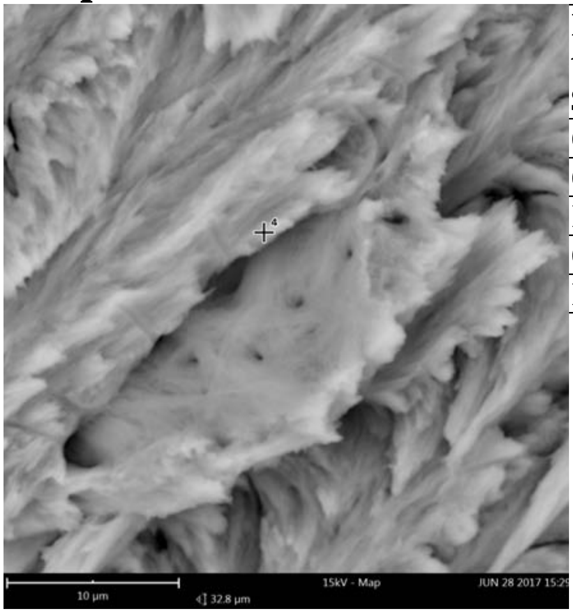

Element Symbol	Atomic Conc.	Weight Conc.	Oxide Symbol	Stoich. Conc.
Ca	24.55	43.45	Ca	38.81
O	36.73	25.95		
P	11.62	15.89	P	18.37
C	26.48	14.04	C	41.84
Mg	0.62	0.67	Mg	0.98

FOV: 32.8 µm, Mode: 15kV - Map, Detector: BSD Full, Time: JUN 28 2017 15:29

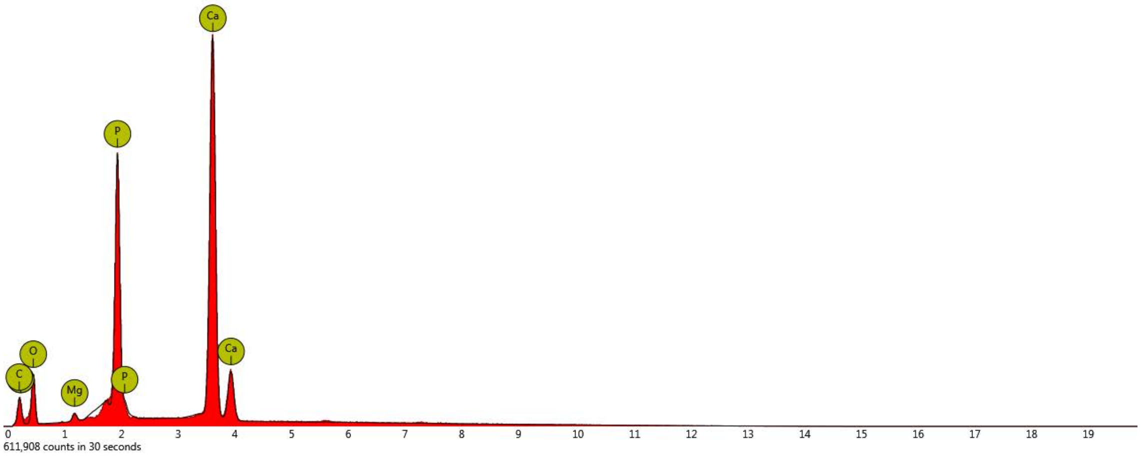

Disabled elements: B

5. map

Combined map

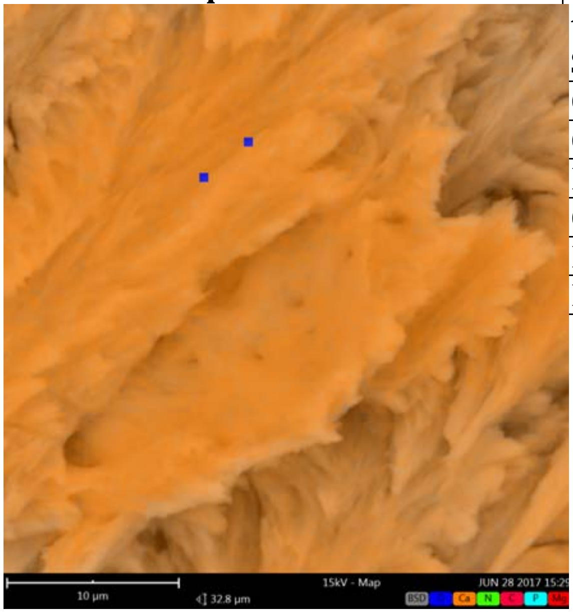

Element Symbol	Atomic Conc.	Weight Conc.	Oxide Symbol	Stoich. Conc.
O	48.12	40.80		
Ca	12.16	25.82	Ca	23.43
N	18.74	13.91	N	36.12
C	14.69	9.35	C	28.31
P	5.68	9.32	P	10.95
Mg	0.62	0.80	Mg	1.19

FOV: 32.8 µm, Mode: 15kV - Map, Detector: BSD Full, Time: JUN 28 2017 15:29

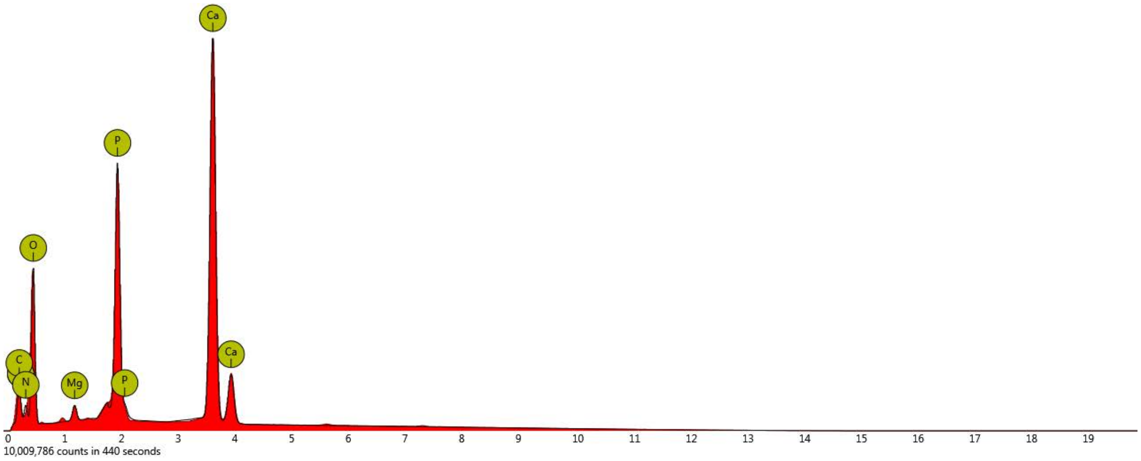

Disabled elements: B

Cut out of map (resolution: 64x64 pixels)

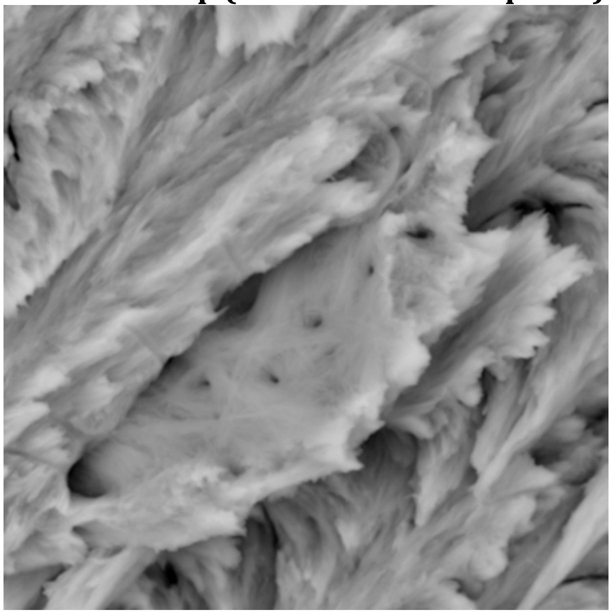

Oxygen

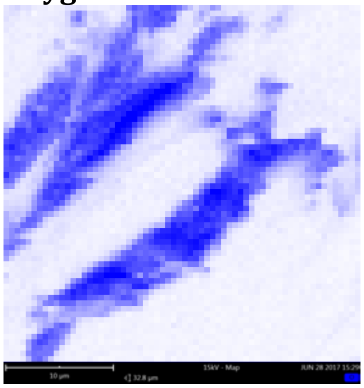

Calcium

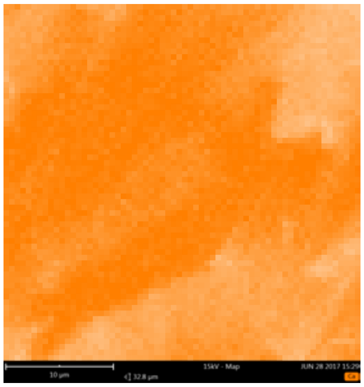

Nitrogen

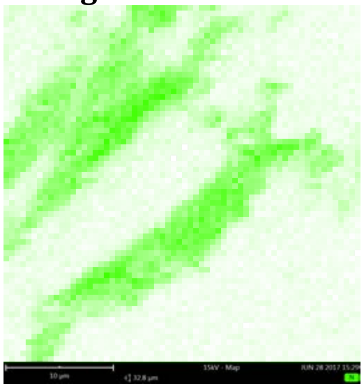

Carbon

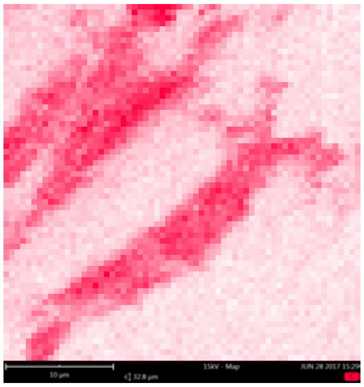

Phosphorus

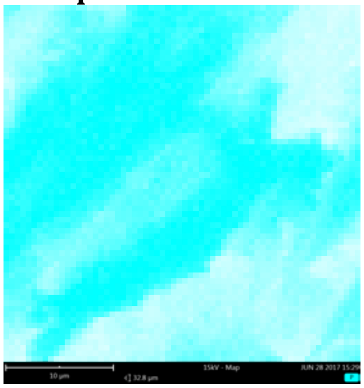

Magnesium

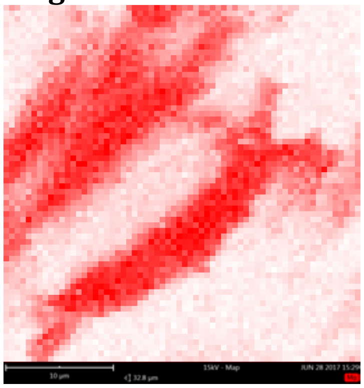

Blood vessels system inside bone 01

Combined map

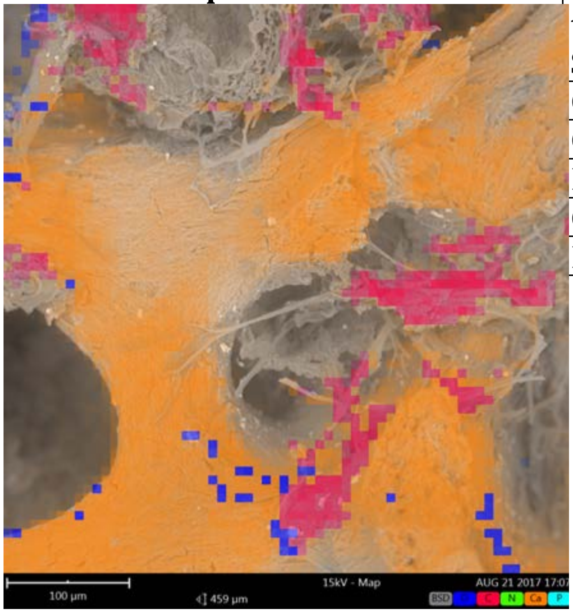

**Element Symbol**	**Atomic Conc.**	**Weight Conc.**	**Oxide Symbol**	**Stoich. Conc.**
O	39.28	41.04		
C	33.03	25.91	C	54.40
N	22.59	20.67	N	37.21
Ca	3.49	9.14	Ca	5.75
P	1.60	3.24	P	2.64

FOV: 459 µm, Mode: 15kV - Map, Detector: BSD Full, Time: AUG 21 2017 17:07

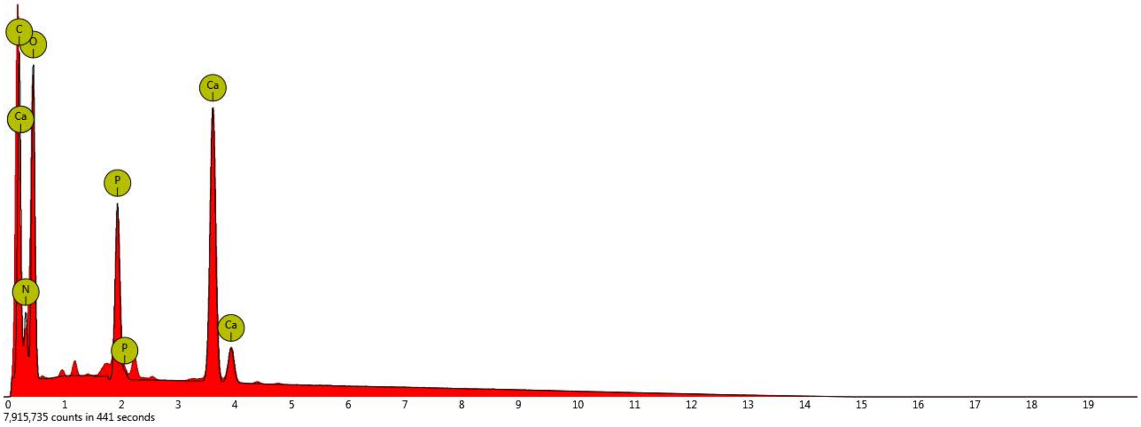

Disabled elements: B, Te

Cut out of map (resolution: 64x64 pixels)

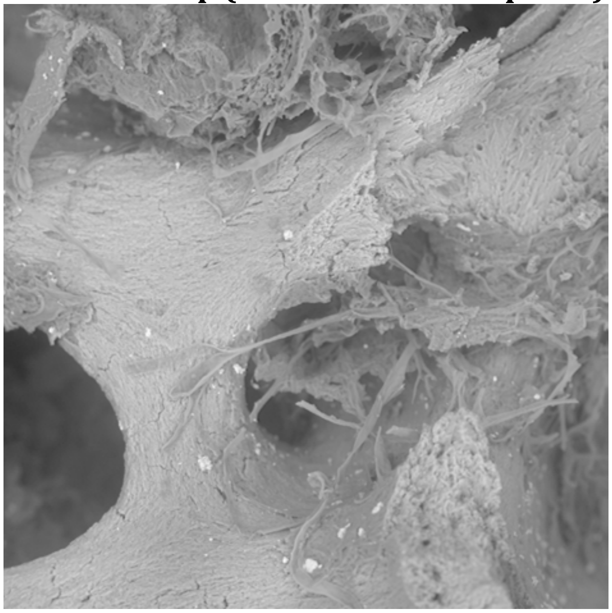

Oxygen

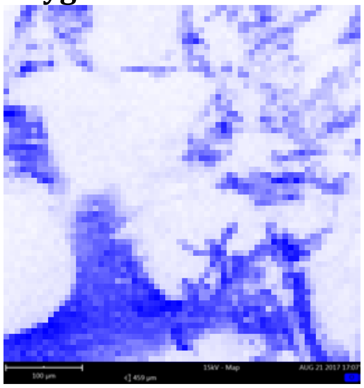

Carbon

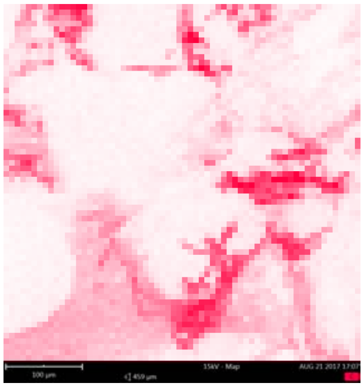

Nitrogen

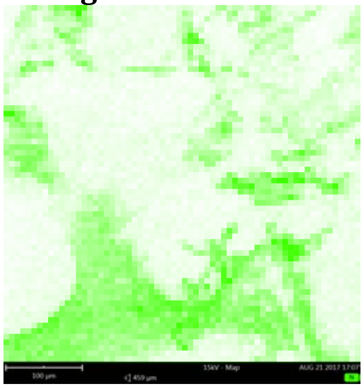

Calcium

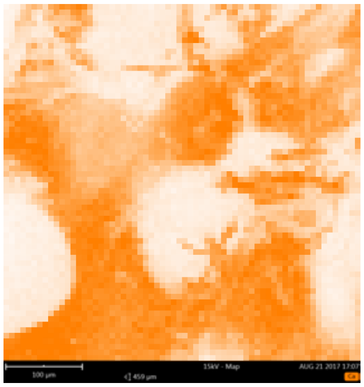

Phosphorus

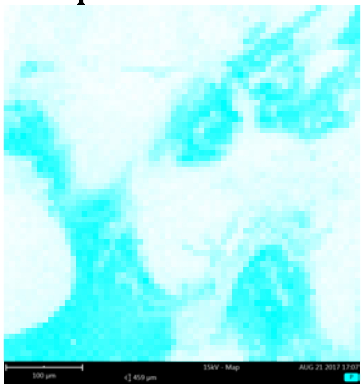

2. spot

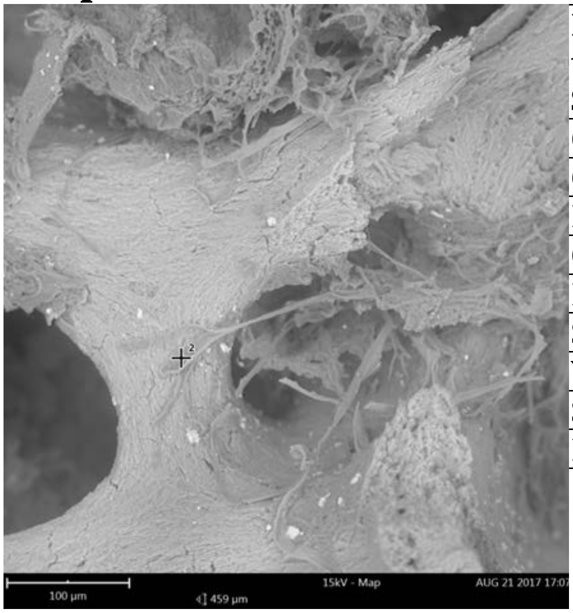

Element Symbol	Atomic Conc.	Weight Conc.	Oxide Symbol	Stoich. Conc.
C	53.67	43.52	C	69.25
O	22.49	24.29		
N	17.88	16.91	N	23.07
Ca	2.97	8.03	Ca	3.83
P	1.85	3.87	P	2.39
Sr	0.17	1.03	Sr	0.22
Y	0.13	0.80	Y	0.17
S	0.36	0.77	S	0.46
Mg	0.47	0.77	Mg	0.61

FOV: 459 µm, Mode: 15kV - Map, Detector: BSD Full, Time: AUG 21 2017 17:07

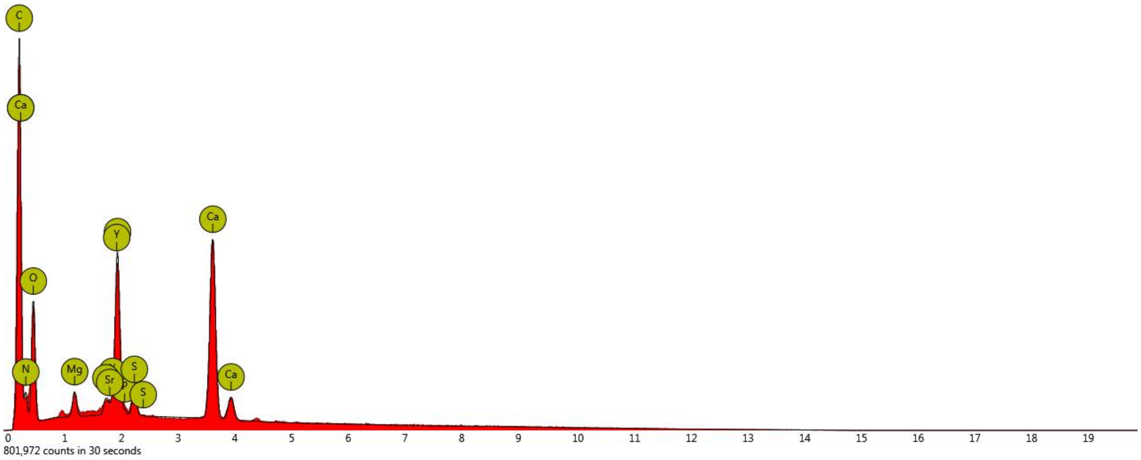

Disabled elements: B

3. spot

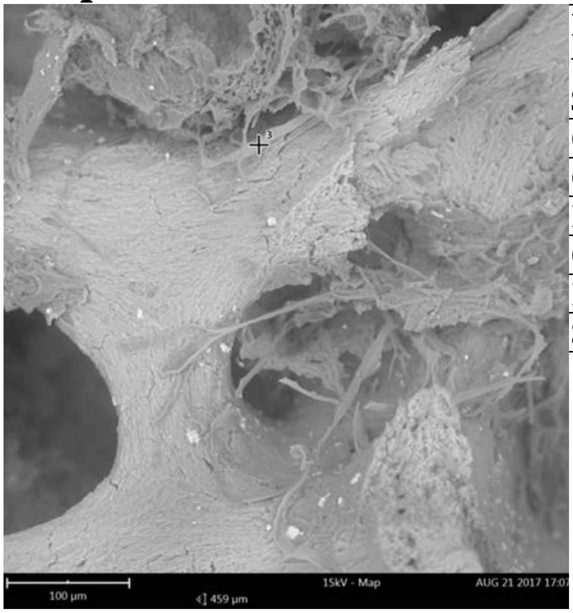

Element Symbol	Atomic Conc.	Weight Conc.	Oxide Symbol	Stoich. Conc.
O	32.15	32.15		
C	34.02	25.54	C	50.14
N	25.54	22.36	N	37.64
Ca	6.81	17.05	Ca	10.03
P	1.20	2.32	P	1.76
S	0.29	0.58	S	0.43

FOV: 459 µm, Mode: 15kV - Map, Detector: BSD Full, Time: AUG 21 2017 17:07

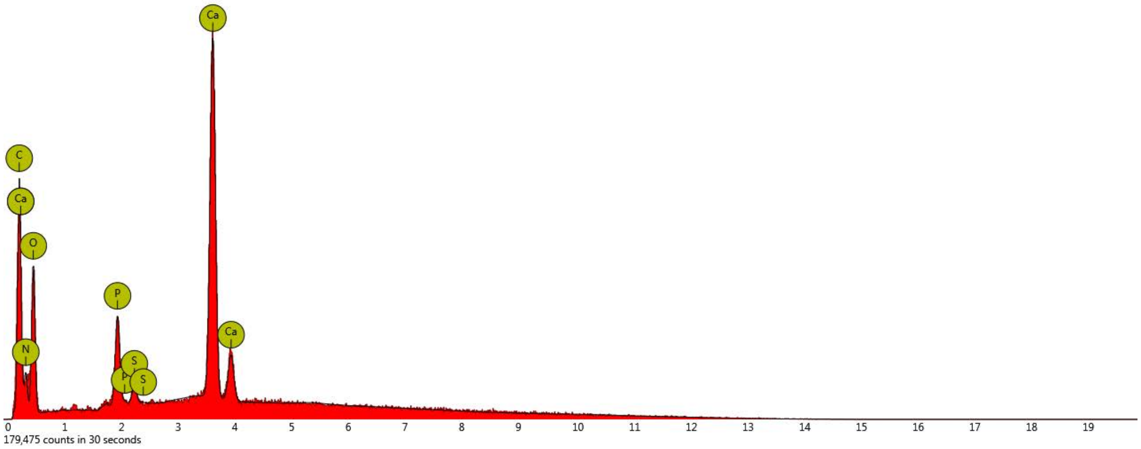

Disabled elements: B

4. spot

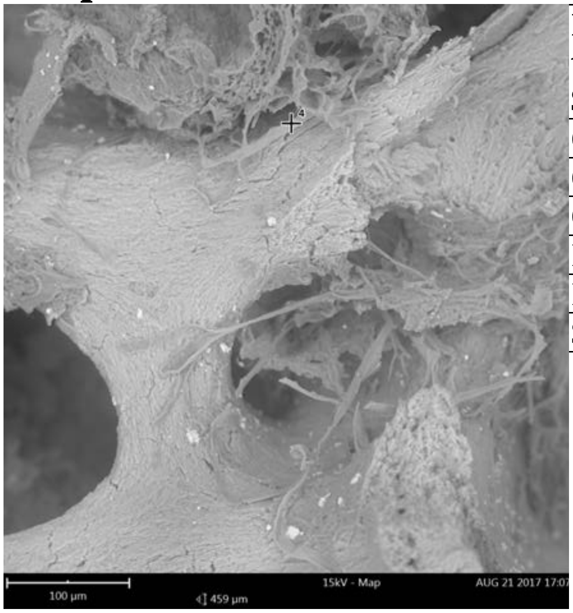

**Element Symbol**	**Atomic Conc.**	**Weight Conc.**	**Oxide Symbol**	**Stoich. Conc.**
Ca	16.30	35.11	Ca	21.97
O	25.79	22.18		
C	31.01	20.02	C	41.79
N	24.24	18.25	N	32.67
P	2.30	3.83	P	3.10
S	0.35	0.60	S	0.47

FOV: 459 µm, Mode: 15kV - Map, Detector: BSD Full, Time: AUG 21 2017 17:07

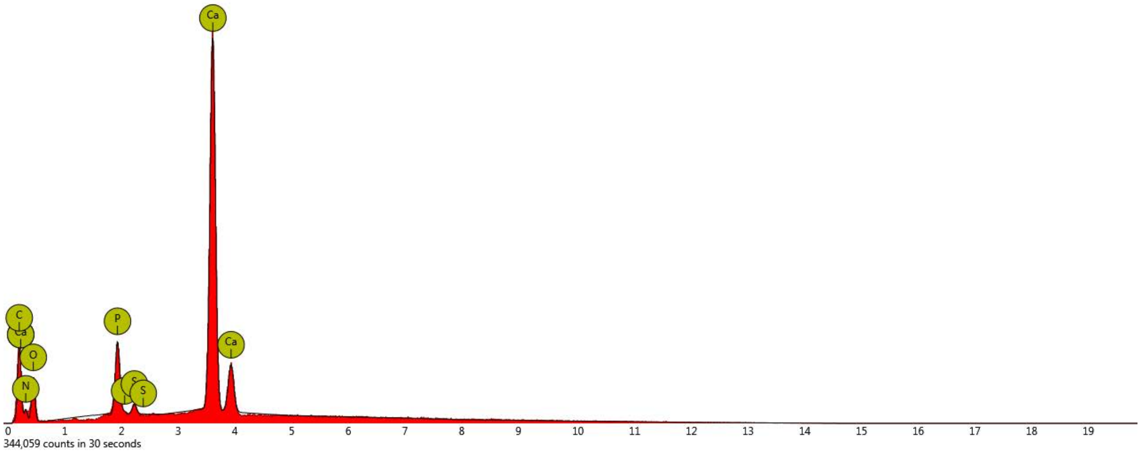

Disabled elements: B

5. spot

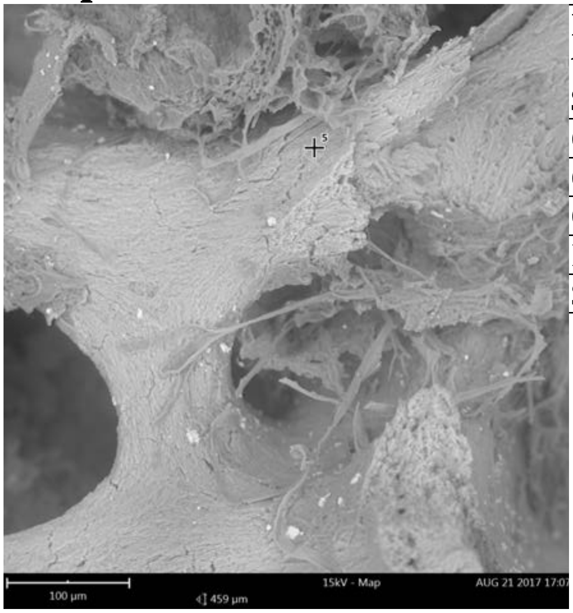

Element Symbol	Atomic Conc.	Weight Conc.	Oxide Symbol	Stoich. Conc.
Ca	24.58	46.58	Ca	34.36
C	41.17	23.38	C	57.56
O	28.47	21.54		
P	5.41	7.93	P	7.57
S	0.37	0.56	S	0.52

FOV: 459 μm, Mode: 15kV - Map, Detector: BSD Full, Time: AUG 21 2017 17:07

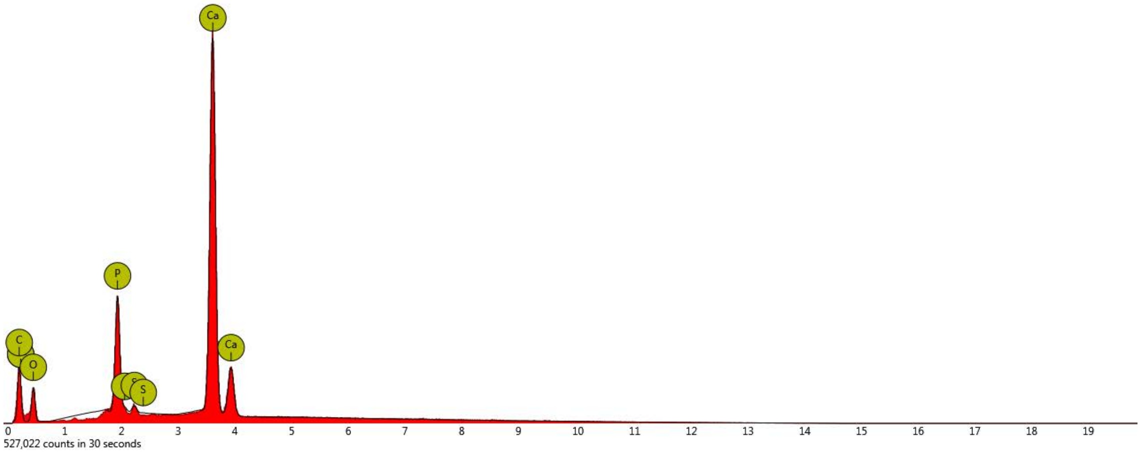

Disabled elements: B

6. spot

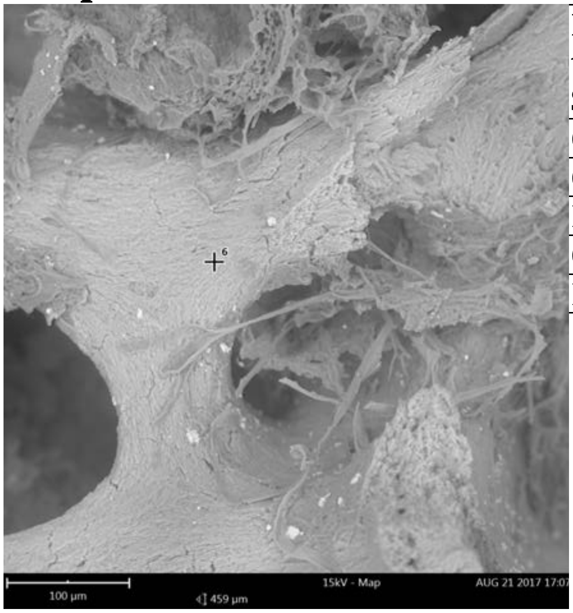

Element Symbol	Atomic Conc.	Weight Conc.	Oxide Symbol	Stoich. Conc.
Ca	16.00	34.39	Ca	23.15
O	30.90	26.52		
N	25.33	19.03	N	36.65
C	25.66	16.53	C	37.13
P	2.12	3.53	P	3.07

FOV: 459 µm, Mode: 15kV - Map, Detector: BSD Full, Time: AUG 21 2017 17:07

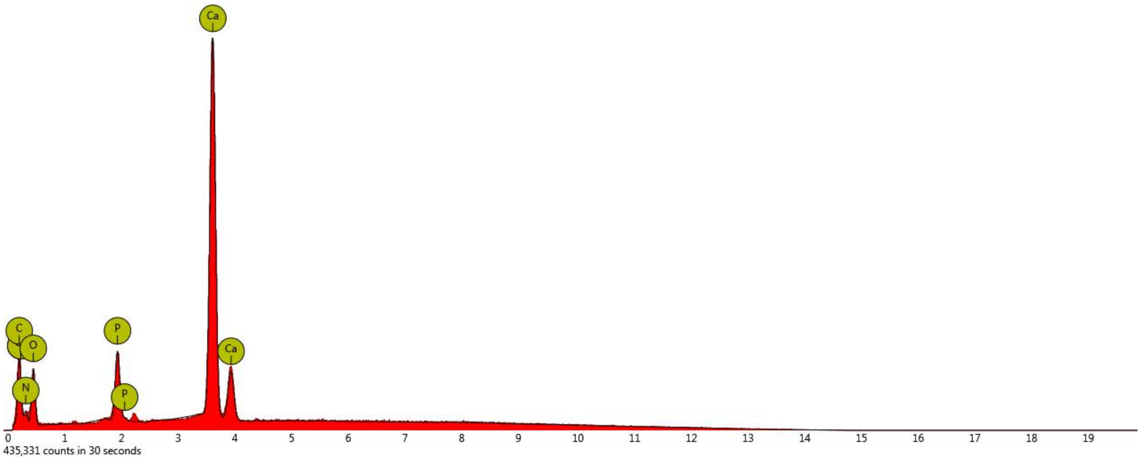

Disabled elements: B

Blood vessels system inside bone 02

1. spot

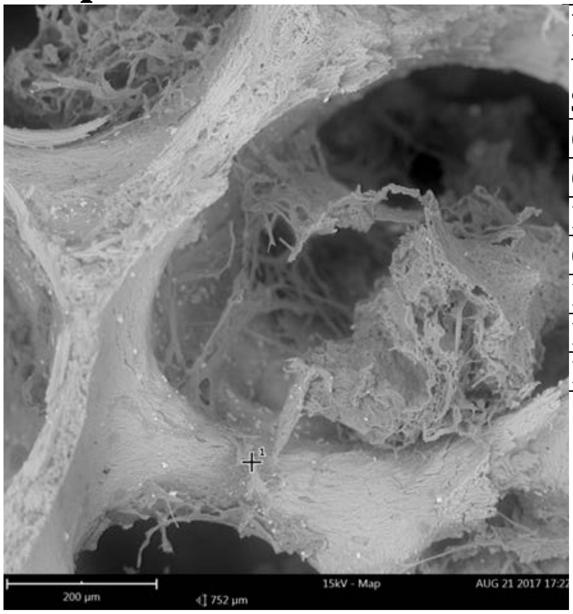

Element Symbol	Atomic Conc.	Weight Conc.	Oxide Symbol	Stoich. Conc.
C	46.37	39.07	C	63.05
O	26.45	29.69		
N	24.32	23.90	N	33.07
Ca	1.26	3.54	Ca	1.71
P	0.71	1.54	P	0.96
Na	0.43	0.69	Na	0.58
In	0.09	0.69	In	0.12

FOV: 752 µm, Mode: 15kV - Map, Detector: BSD Full, Time: AUG 21 2017 17:22

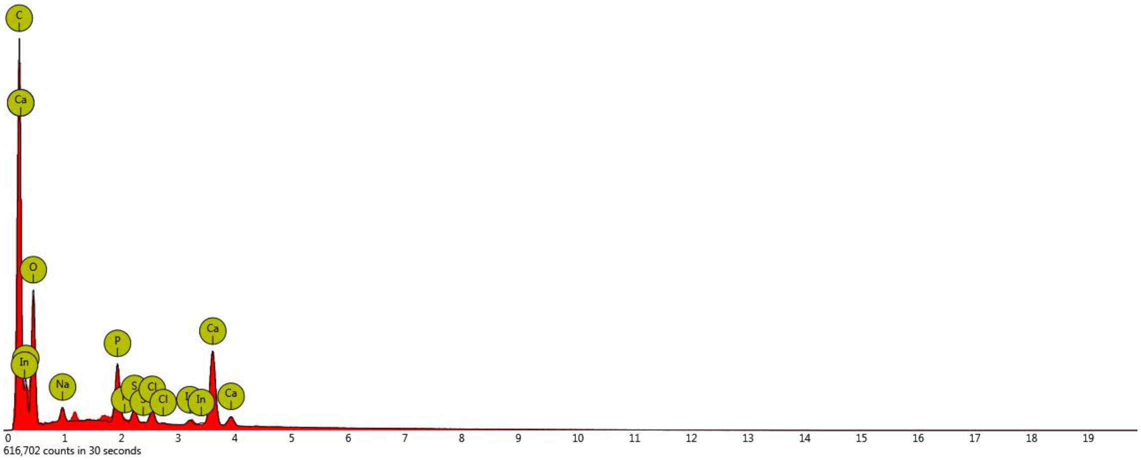

Disabled elements: B

2. spot

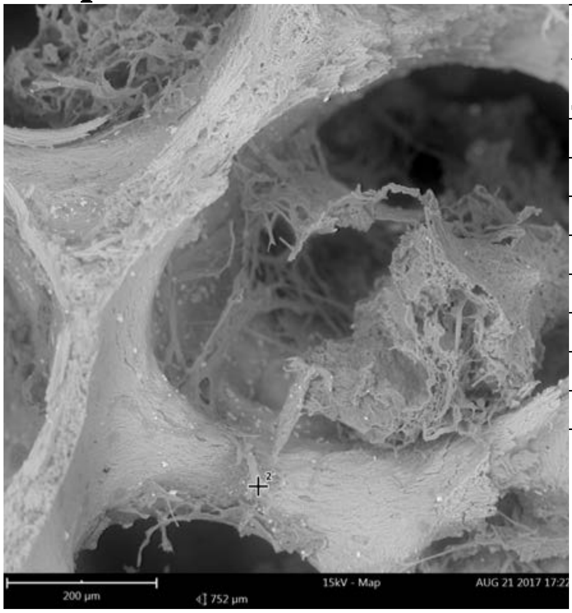

Element Symbol	Atomic Conc.	Weight Conc.	Oxide Symbol	Stoich. Conc.
C	44.41	31.34	C	65.68
O	32.38	30.43		
Ba	1.83	14.79	Ba	2.71
N	17.40	14.32	N	25.73
S	1.47	2.77	S	2.18
Ca	1.11	2.62	Ca	1.65
Sr	0.40	2.03	Sr	0.58
P	0.69	1.25	P	1.02

FOV: 752 µm, Mode: 15kV - Map, Detector: BSD Full, Time: AUG 21 2017 17:22

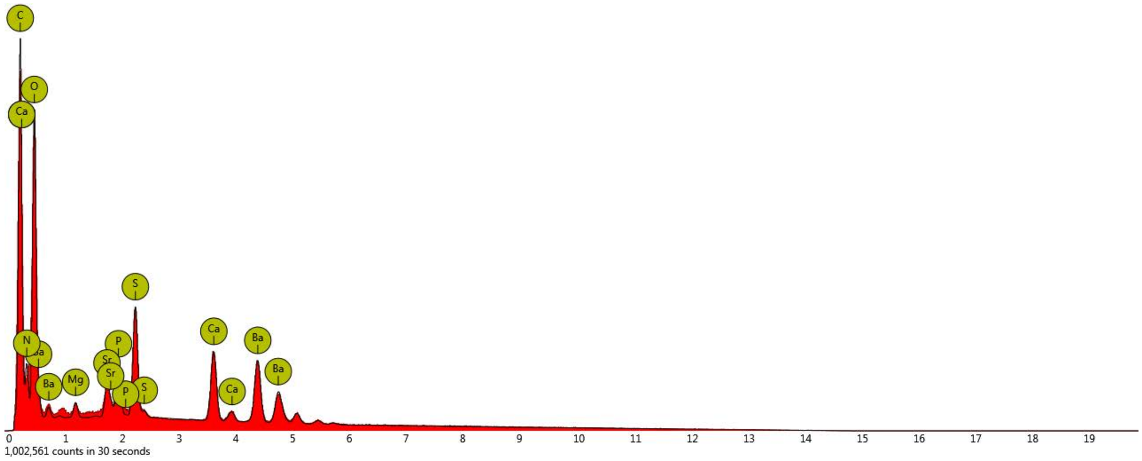

Disabled elements: B

3. spot

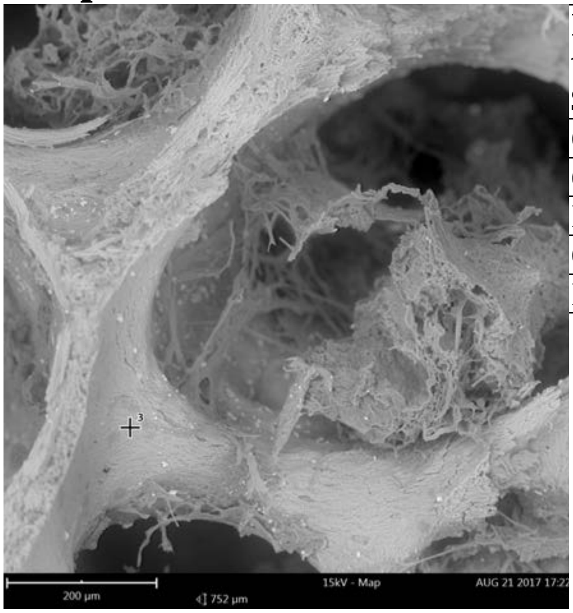

Element Symbol	Atomic Conc.	Weight Conc.	Oxide Symbol	Stoich. Conc.
O	37.21	38.98		
C	33.84	26.61	C	53.89
N	23.90	21.92	N	38.07
Ca	3.78	9.92	Ca	6.02
P	1.27	2.57	P	2.02

FOV: 752 µm, Mode: 15kV - Map, Detector: BSD Full, Time: AUG 21 2017 17:22

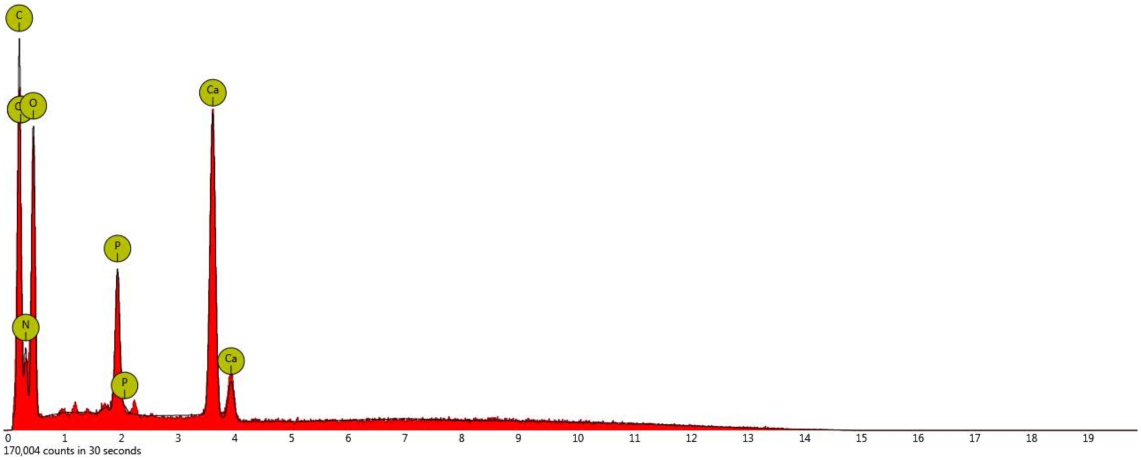

Disabled elements: B

4. spot

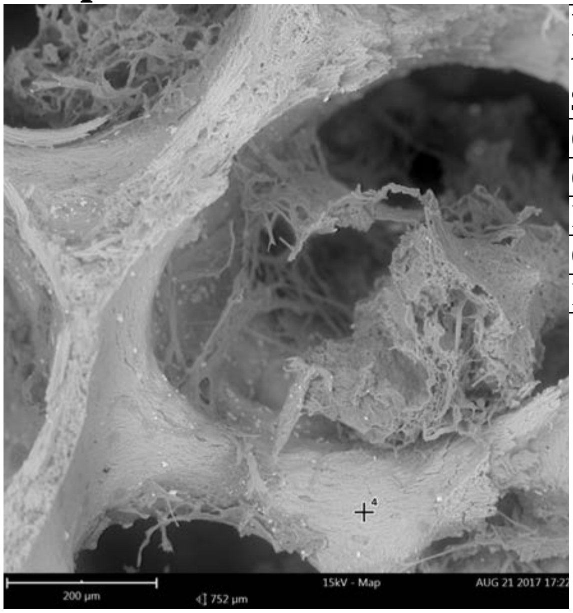

**Element Symbol**	**Atomic Conc.**	**Weight Conc.**	**Oxide Symbol**	**Stoich. Conc.**
Ca	18.75	39.02	Ca	26.97
O	30.49	25.34		
N	24.48	17.81	N	35.22
C	24.80	15.47	C	35.69
P	1.48	2.37	P	2.12

FOV: 752 µm, Mode: 15kV - Map, Detector: BSD Full, Time: AUG 21 2017 17:22

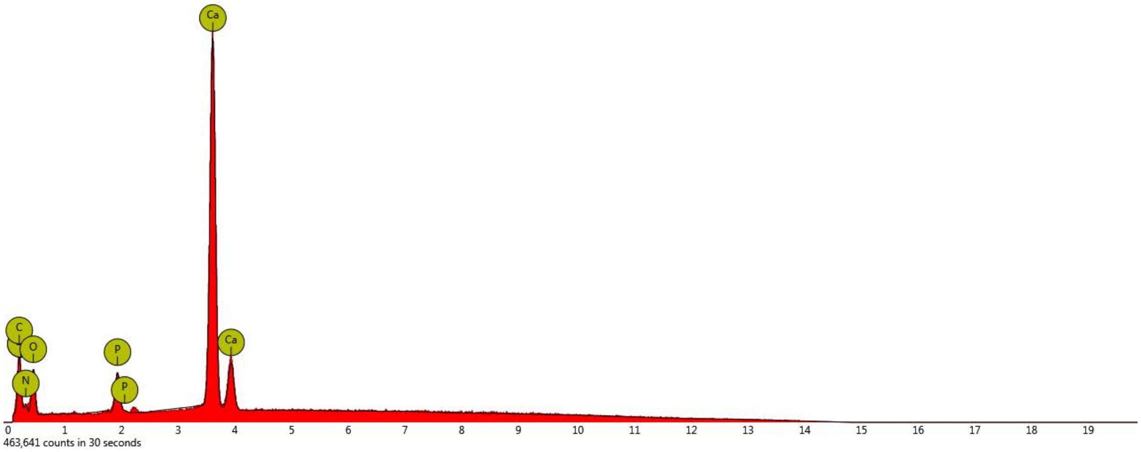

Disabled elements: B

Image 17

1. spot

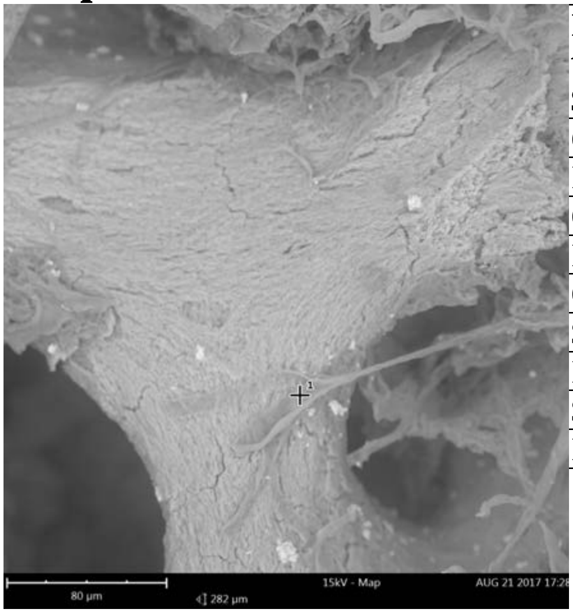

Element Symbol	Atomic Conc.	Weight Conc.	Oxide Symbol	Stoich. Conc.
C	53.33	31.10	C	65.29
Ba	4.19	27.93	Ba	5.13
O	18.32	14.23		
N	15.24	10.37	N	18.66
Ca	3.37	6.55	Ca	4.12
S	2.65	4.13	S	3.25
P	1.87	2.82	P	2.29
Sr	0.54	2.31	Sr	0.66
Mg	0.48	0.57	Mg	0.59

FOV: 282 µm, Mode: 15kV - Map, Detector: BSD Full, Time: AUG 21 2017 17:28

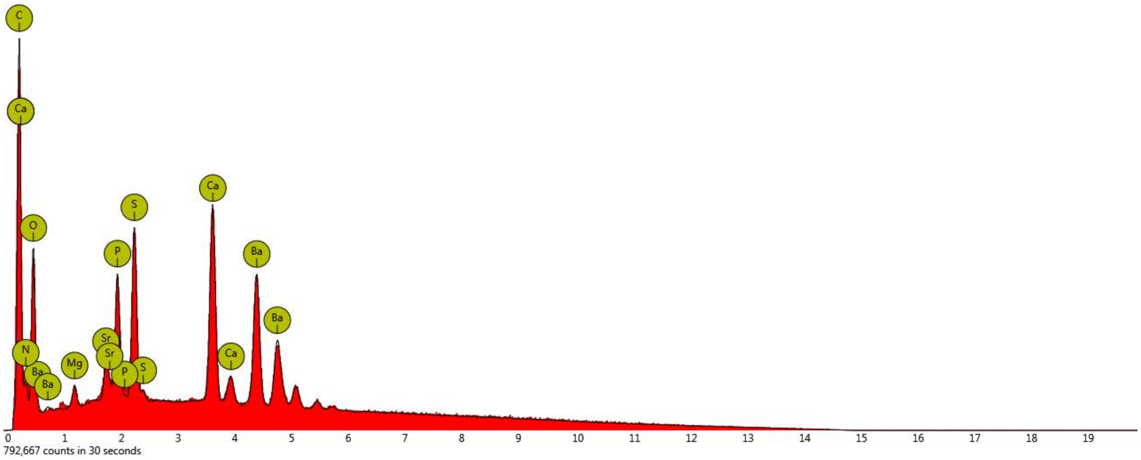

Disabled elements: B

2. spot

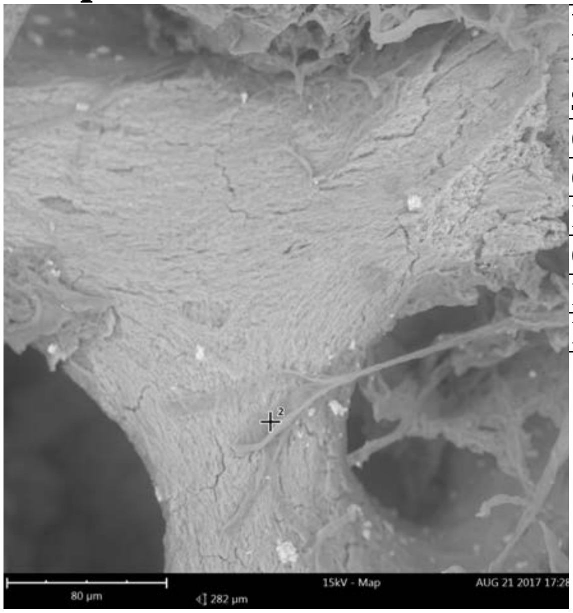

Element Symbol	Atomic Conc.	Weight Conc.	Oxide Symbol	Stoich. Conc.
C	53.42	42.95	C	69.14
O	22.74	24.35		
N	16.75	15.71	N	21.69
Ca	4.09	10.97	Ca	5.29
P	2.29	4.75	P	2.96
Mg	0.49	0.79	Mg	0.63

FOV: 282 µm, Mode: 15kV - Map, Detector: BSD Full, Time: AUG 21 2017 17:28

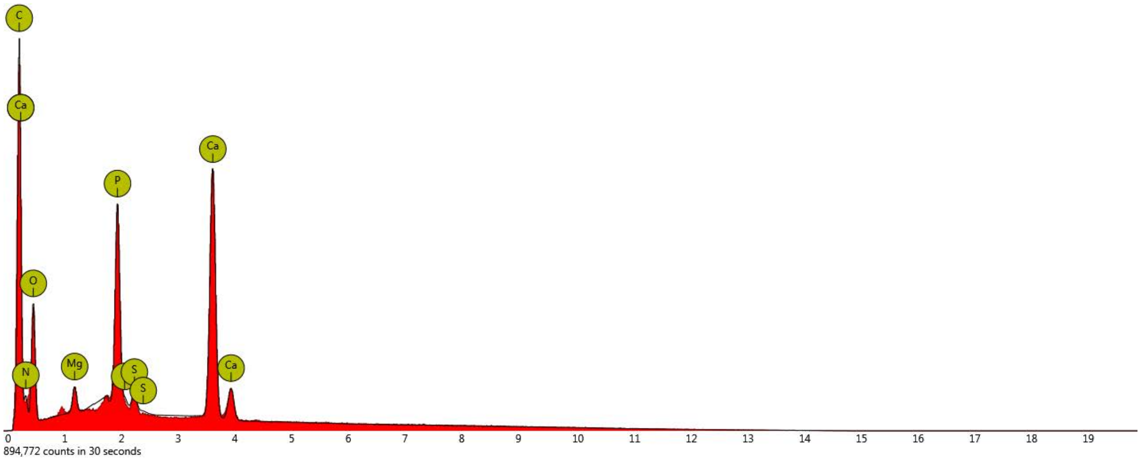

Disabled elements: B

3. spot

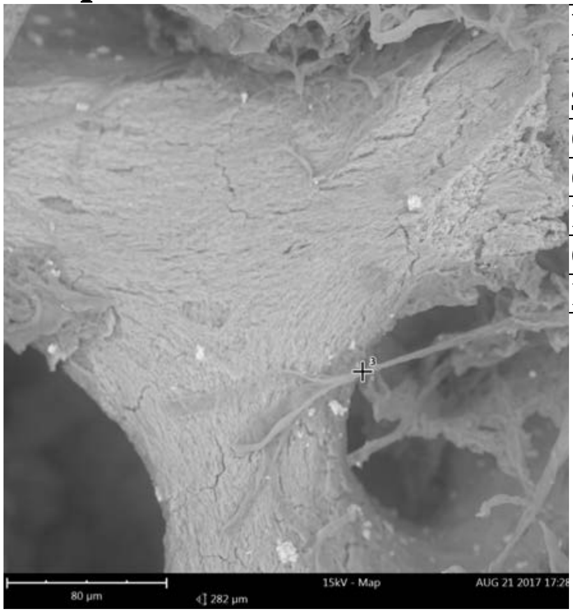

Element Symbol	Atomic Conc.	Weight Conc.	Oxide Symbol	Stoich. Conc.
O	34.16	36.69		
C	38.88	31.35	C	59.05
N	22.76	21.41	N	34.57
Ca	2.94	7.90	Ca	4.46
P	1.11	2.30	P	1.68

FOV: 282 µm, Mode: 15kV - Map, Detector: BSD Full, Time: AUG 21 2017 17:28

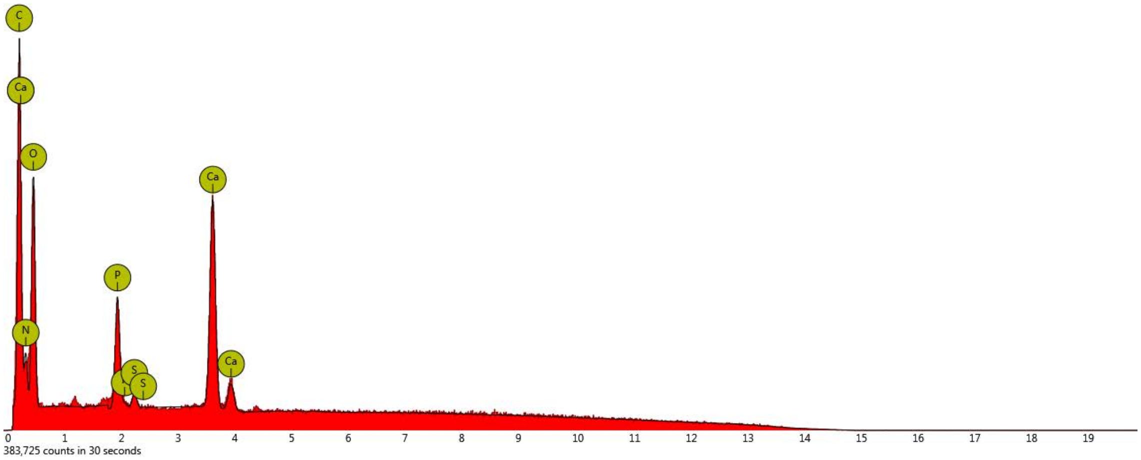

Disabled elements: B

4. spot

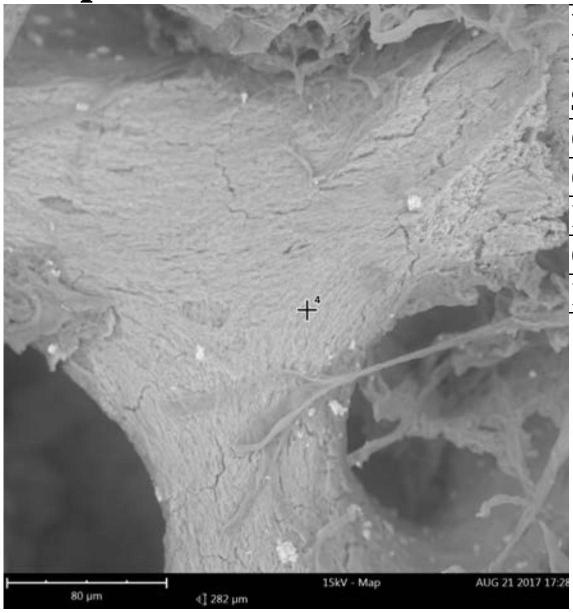

Element Symbol	Atomic Conc.	Weight Conc.	Oxide Symbol	Stoich. Conc.
Ca	16.66	35.44	Ca	23.97
O	30.53	25.93		
N	23.29	17.31	N	33.52
C	27.05	17.25	C	38.94
P	2.48	4.08	P	3.57

FOV: 282 µm, Mode: 15kV - Map, Detector: BSD Full, Time: AUG 21 2017 17:28

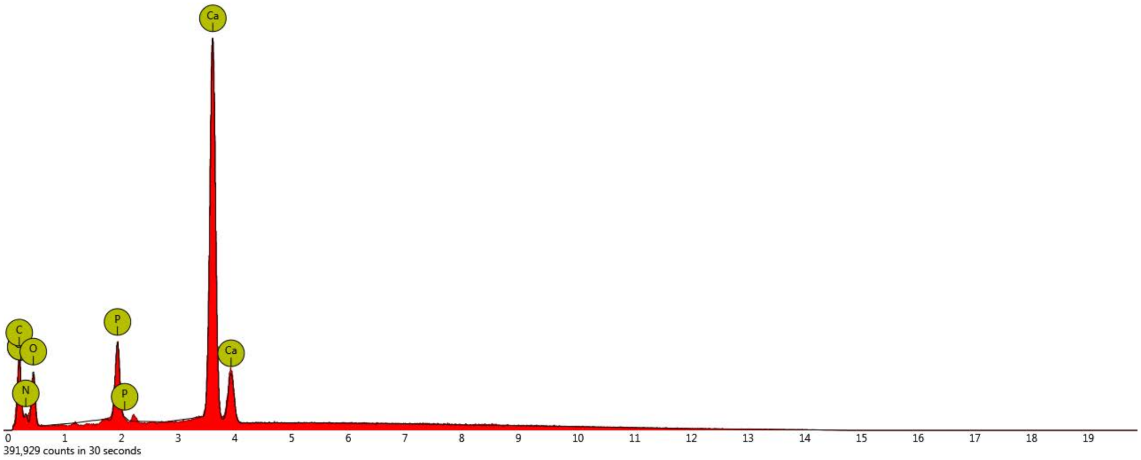

Disabled elements: B

5. spot

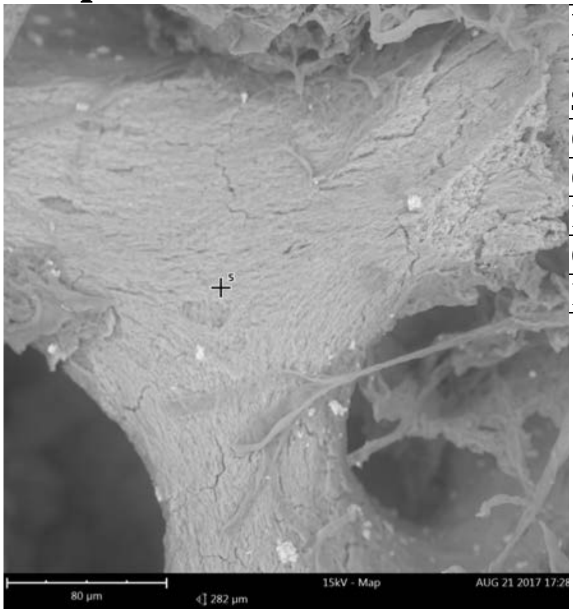

Element Symbol	Atomic Conc.	Weight Conc.	Oxide Symbol	Stoich. Conc.
Ca	17.84	37.50	Ca	25.80
O	30.84	25.88		
N	25.04	18.39	N	36.21
C	24.57	15.47	C	35.53
P	1.70	2.77	P	2.47

FOV: 282 µm, Mode: 15kV - Map, Detector: BSD Full, Time: AUG 21 2017 17:28

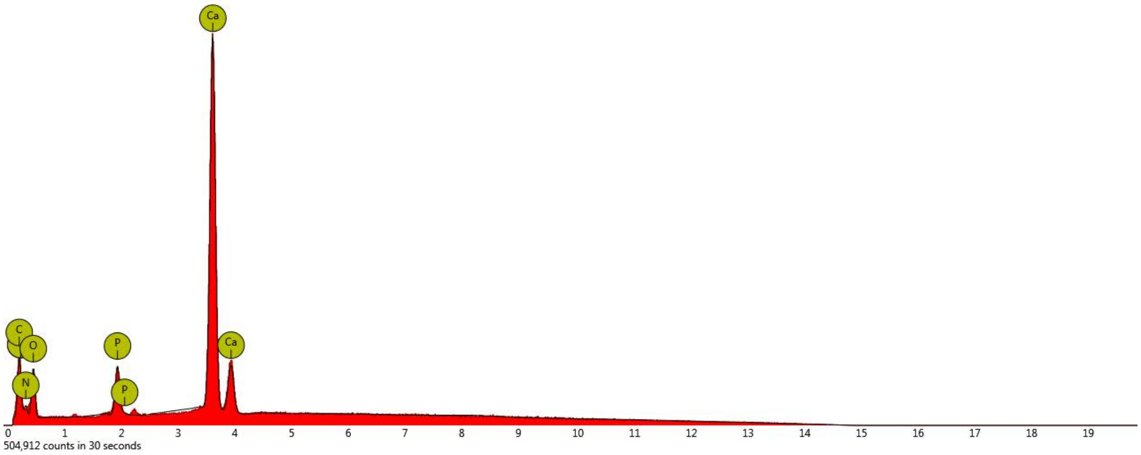

Disabled elements: B

Gallus gallus (uncatalogued specimen)

Osteocytes embedded in bone 01

1. map

Combined map

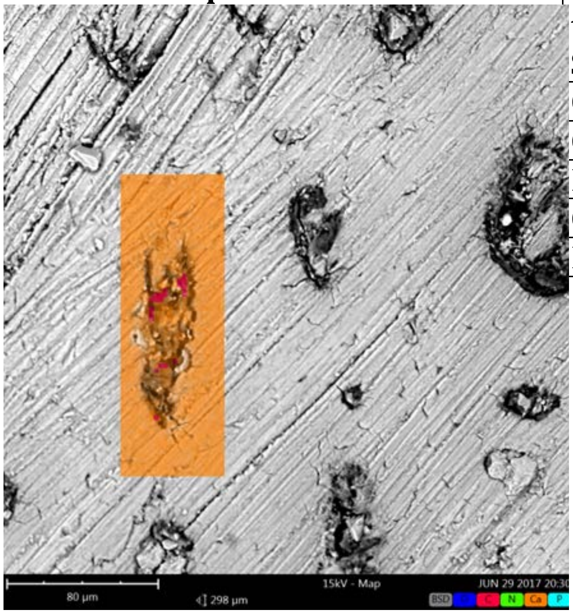

Element Symbol	Atomic Conc.	Weight Conc.	Oxide Symbol	Stoich. Conc.
O	42.23	42.31		
C	28.56	21.48	C	49.45
N	21.67	19.01	N	37.51
Ca	4.53	11.37	Ca	7.84
P	3.00	5.82	P	5.19

FOV: 298 µm, Mode: 15kV - Map, Detector: BSD Full, Time: JUN 29 2017 20:30

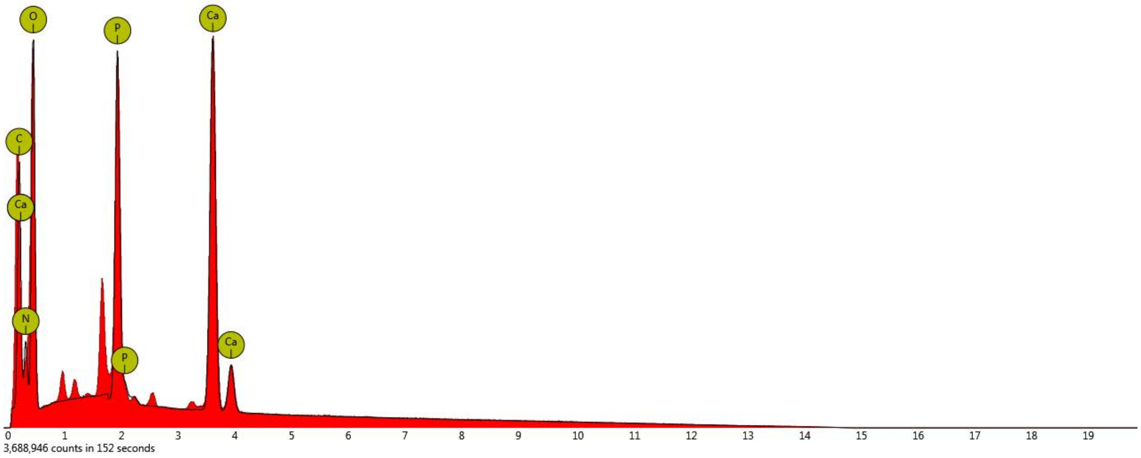

Disabled elements: B, Rb, Si, Sr, Te

Cut out of map (resolution: 22x64 pixels)

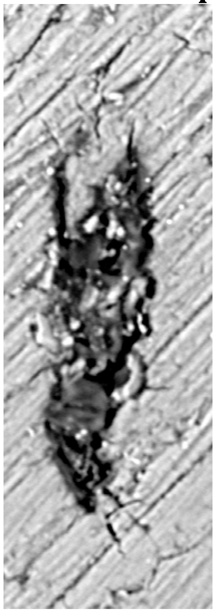

Oxygen

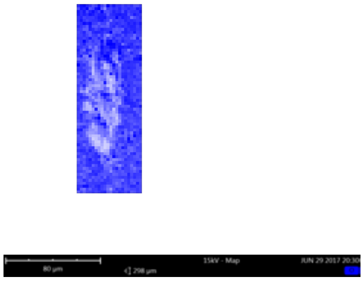

Carbon

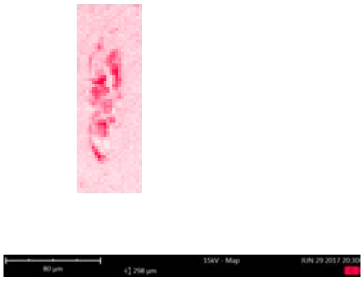

Nitrogen

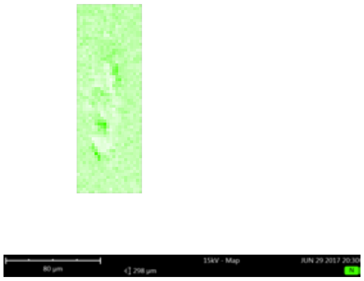

Calcium

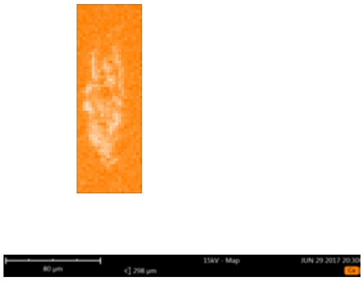

Phosphorus

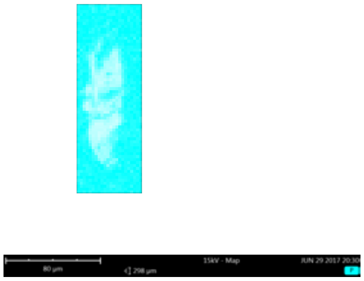

1. map

Combined map

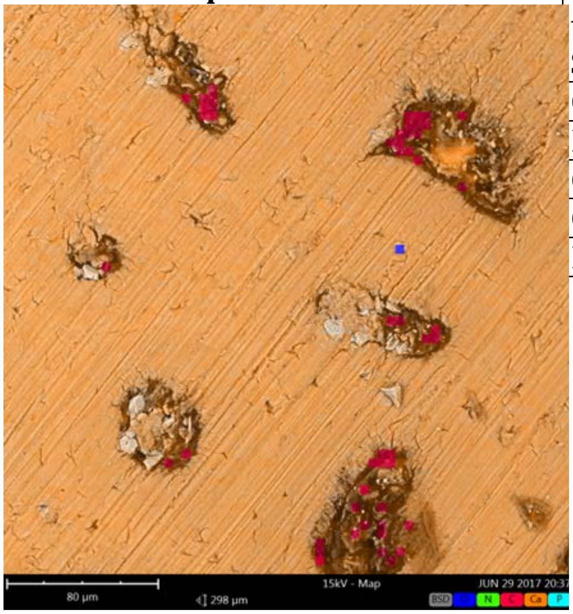

Element Symbol	Atomic Conc.	Weight Conc.	Oxide Symbol	Stoich. Conc.
O	44.82	44.18		
N	22.20	19.16	N	40.22
C	24.82	18.37	C	44.97
Ca	4.82	11.91	Ca	8.74
P	3.35	6.39	P	6.07

FOV: 298 µm, Mode: 15kV - Map, Detector: BSD Full, Time: JUN 29 2017 20:37

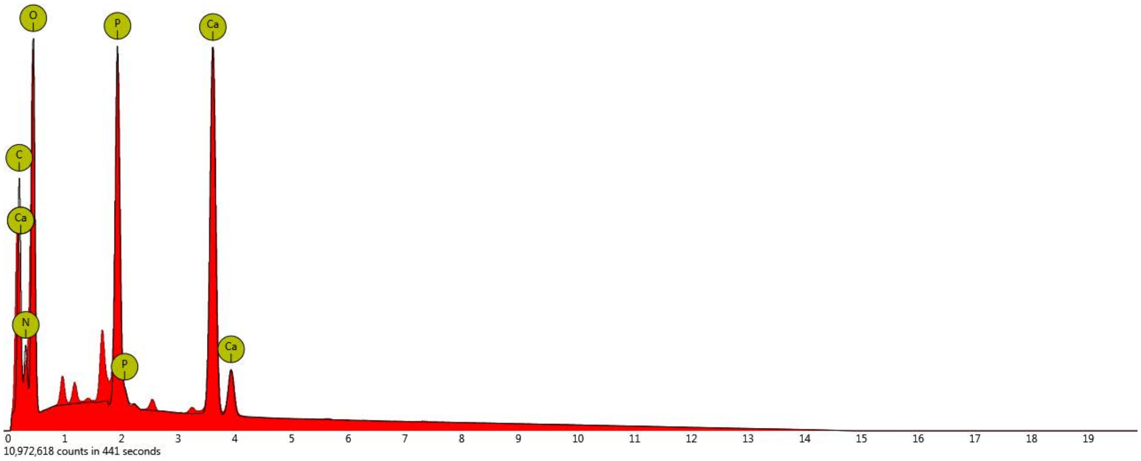

Disabled elements: B, Rb, Si, Sr

Cut out of map (resolution: 64x64 pixels)

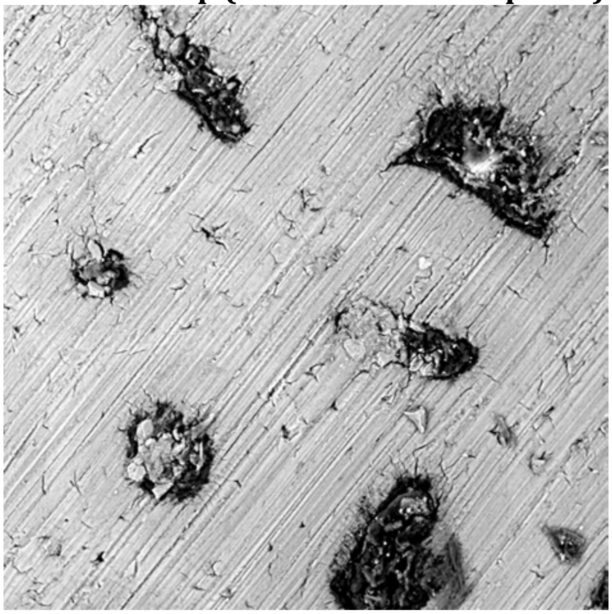

Oxygen

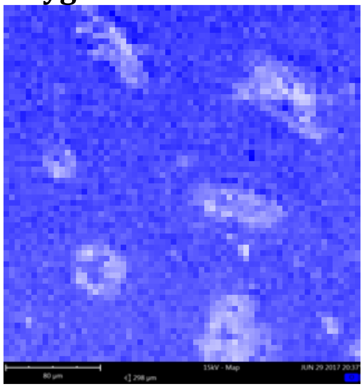

Nitrogen

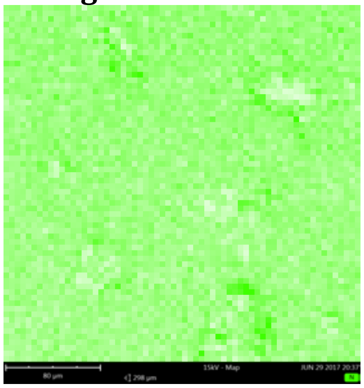

Carbon

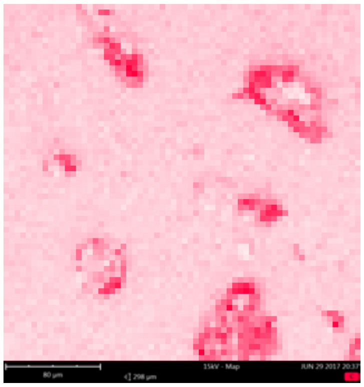

Calcium

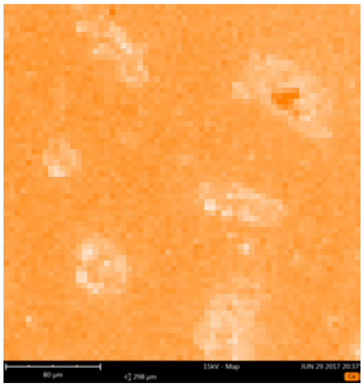

Phosphorus

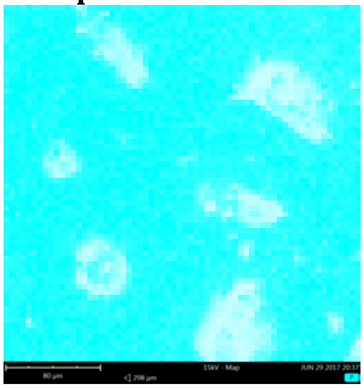

Supplement: Supplemental Information 1 [file peerj-08-9833-s001.pdf]
