# Supplementary material for: In situ SEM/EDS compositional characterization of osteocytes and blood vessels in fossil and extant turtles on untreated bone surfaces; different preservational pathways microns away
Source: PeerJ. 2020 Aug 27;8:e9833. doi: 10.7717/peerj.9833 (PMC7456530; doi:10.7717/peerj.9833)
Supplement: Supplemental Information 3 — Additional figures of fossil and extant samples studied here. [file peerj-08-9833-s003.pdf]

In situ SEM/EDS compositional characterization of osteocytes- and blood vessels-like in fossil and extant turtles on untreated bone surfaces; different preservational pathways microns away

Edwin-Alberto Cadena

Supplemental Figures S1–S4

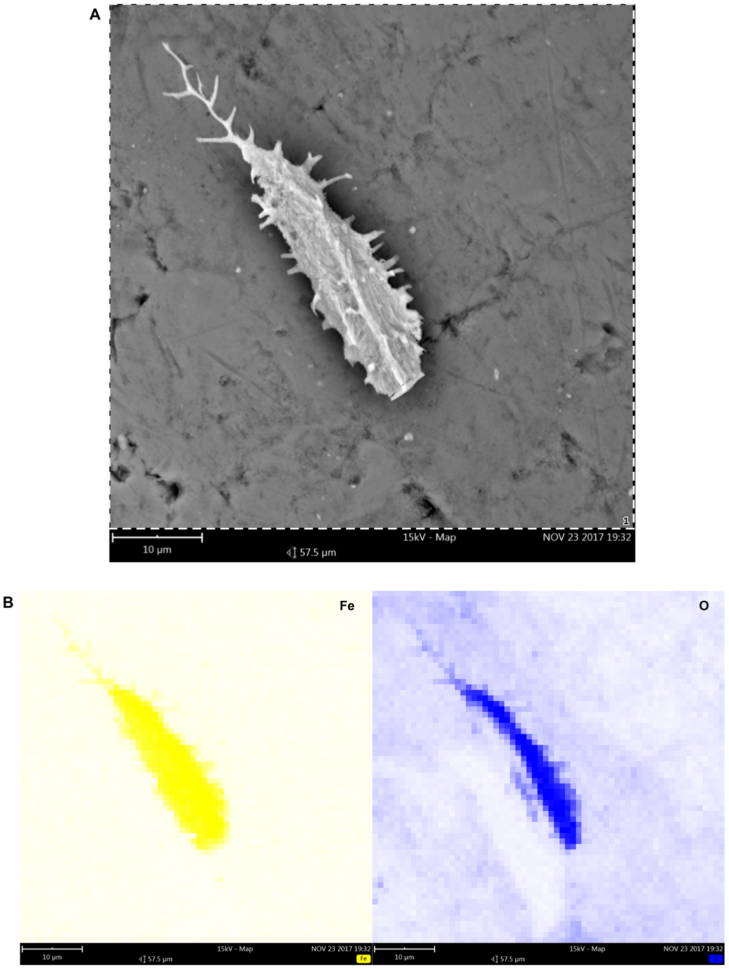

Figure S1. SEM/EDS analysis of _Mongolemys elegans_ (IGM-90/42) isolated osteocyte-like. (A) Micrograph of the osteocyte measuring 38 µm. (B) EDS map of the isolated osteocyte-like showing a high amount of iron on its entire surface.

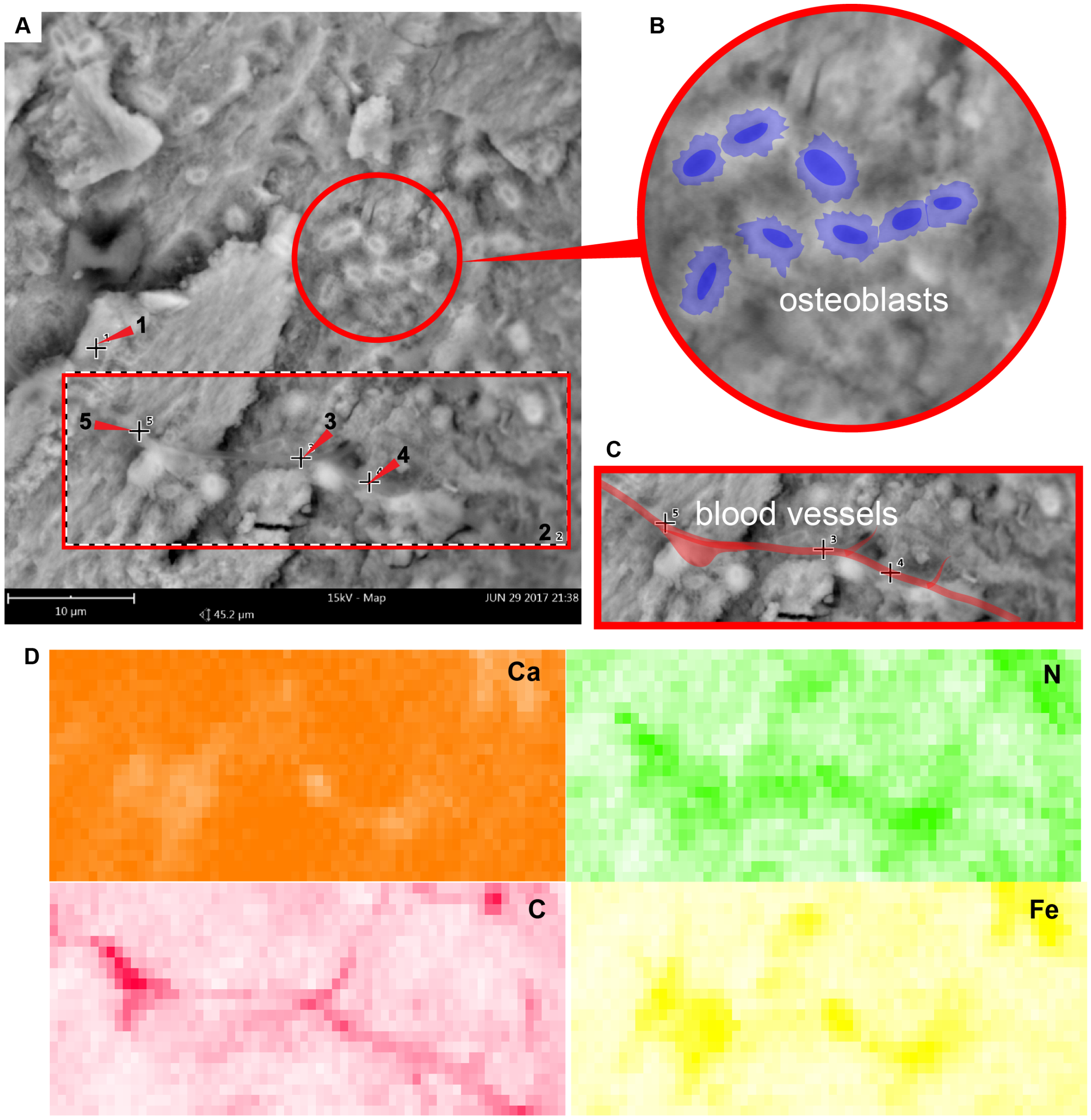

Figure S2. SEM/EDS analysis of *Allaeochelys crassesculpta* (SMF ME 2449) bone. (A) Micrograph indicating the two zones shown in (B) and (D), as well as the points for which EDS data was obtained (see Fig. 2). (B) Close-up showing some of the osteoblasts-like. (C) Close-up of the blood vessels-like system. (D) EDS map of the blood vessels-like system showing a rich amount of carbon, nitrogen, and iron, which are absent in the surrounding bone matrix dominated by calcium.

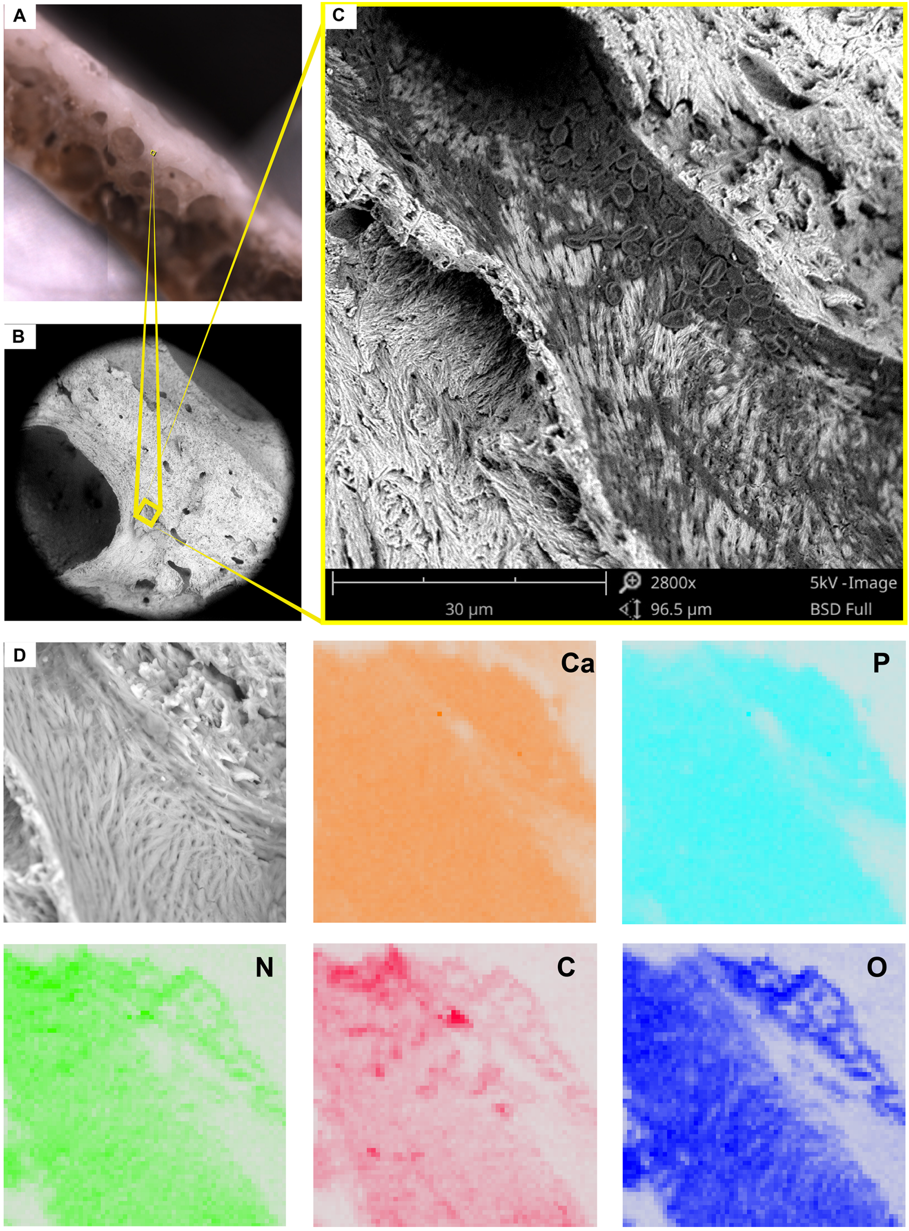

Figure S3. SEM/EDS analysis of the extant *Podocnemis lewyana* bone. (A) Bone in the SEM holder. (B) Micrograph of bone at the base of the external cortex. (C) Close-up of one of the Haversian canals showing red blood cells. (D) EDS map of the Haversian canal shown in (C), the walls of the canal are rich in carbon and nitrogen, especially where the red blood cells are located.

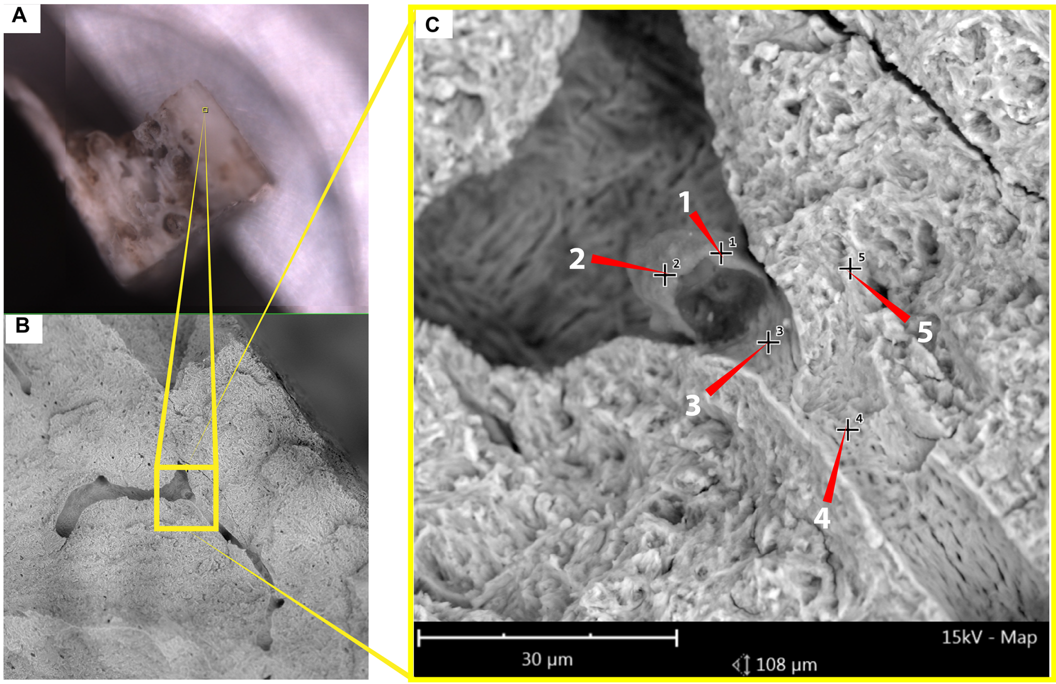

D

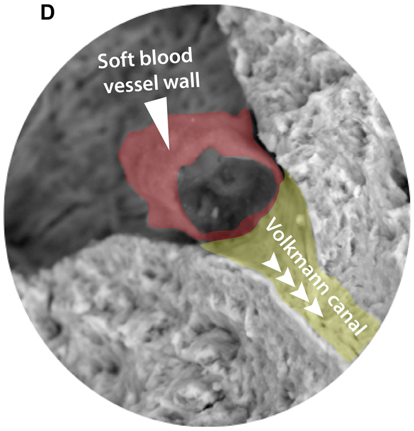

E **Point 2**

Element Symbol	Atomic Conc.	Weight Conc.	Oxide Symbol	Stoich. Conc.
C	52.00	45.06	C	67.02
O	22.41	25.86		
N	23.61	23.86	N	30.43
Ca	1.22	3.54	Ca	1.58
P	0.75	1.68	P	0.97

F **Point 5**

Element Symbol	Atomic Conc.	Weight Conc.	Oxide Symbol	Stoich. Conc.
O	52.68	47.08		
Ca	8.76	19.62	Ca	18.52
N	15.56	12.17	N	32.88
C	17.51	11.75	C	37.00
P	5.15	8.91	P	10.88

Figure S4. SEM/EDS analysis of the extant *Podocnemis lewyana* bone. (A) Bone in the SEM holder. (B) Micrograph of bone at its external cortex region. (C–D) Close-up of one of the Volkmann canals, and its soft blood wall. (E) EDS elemental analysis of point 2, indicated in (C) showing a high amount of carbon and nitrogen in the wall forms the Volkmann canal. (D) EDS elemental analysis of point 5, indicated in (C) showing the composition of the bone matrix (calcium, phosphorus, carbon and nitrogen).
